# Isomerization of Olefins Triggered by Rhodium-Catalyzed C–H Bond Activation: Control of Endocyclic β-Hydrogen Elimination**

**DOI:** 10.1002/anie.201500596

**Published:** 2015-04-23

**Authors:** Stephanie Y Y Yip, Christophe Aïssa

**Affiliations:** Department of Chemistry, University of Liverpool Crown Street, L69 7ZD (UK)

**Keywords:** C–H activation, isomerization, metallacycles, rhodium, β-H elimination

## Abstract

Five-membered metallacycles are typically reluctant to undergo endocyclic β-hydrogen elimination. The rhodium-catalyzed isomerization of 4-pentenals into 3-pentenals occurs through this elementary step and cleavage of two C–H bonds, as supported by deuterium-labeling studies. The reaction proceeds without decarbonylation, leads to *trans* olefins exclusively, and tolerates other olefins normally prone to isomerization. Endocyclic β-hydrogen elimination can also be controlled in an enantiodivergent reaction on a racemic mixture.

Five-membered metallacycles are important intermediates of numerous catalytic processes, both in academic laboratories and in large-scale industrial chemistry.[1] As shown in experimental[2] and theoretical[3] studies, geometric constraints make these intermediates reluctant to undergo endocyclic β-hydrogen (β-H) elimination, especially in the case of square-planar complexes. However, and although thorough experimental studies are still lacking, theoretical studies suggest that five-membered metallacycles that are not square-planar could undergo β-H elimination more easily.[4] For example, recent calculations indicate that the rhodium-catalyzed decarbonylation of 4-pentenals could occur by reversible endocyclic β-H elimination of intermediate **A** (R=H) (Figure 1).[5] Importantly, substrate decarbonylation is a notorious problem during the hydroacylation of 4-pentenals, especially in the case of α,α-disubstituted aldehydes (R≠H).[6–9] In contrast, we have found that the rhodium-catalyzed isomerization of 4-pentenal **1** (R=Ph) into 3-pentenal **2** occurs without decarbonylation in 86 % yield and in a highly stereoselective fashion. The most efficient catalyst was prepared with ligand **L1**,[10] whereas those prepared with **L2**–**L5** led to incomplete conversion and decarbonylation. Hence, we assumed this isomerization to be triggered by C–H bond activation (a), and the catalytic cycle would be completed by migratory insertion of the terminal olefin into the rhodium-hydrogen bond thus engendered (b), followed by endocyclic β-H elimination of **A** (R=Ph) (c), and final reductive elimination (d).

**Figure 1:**
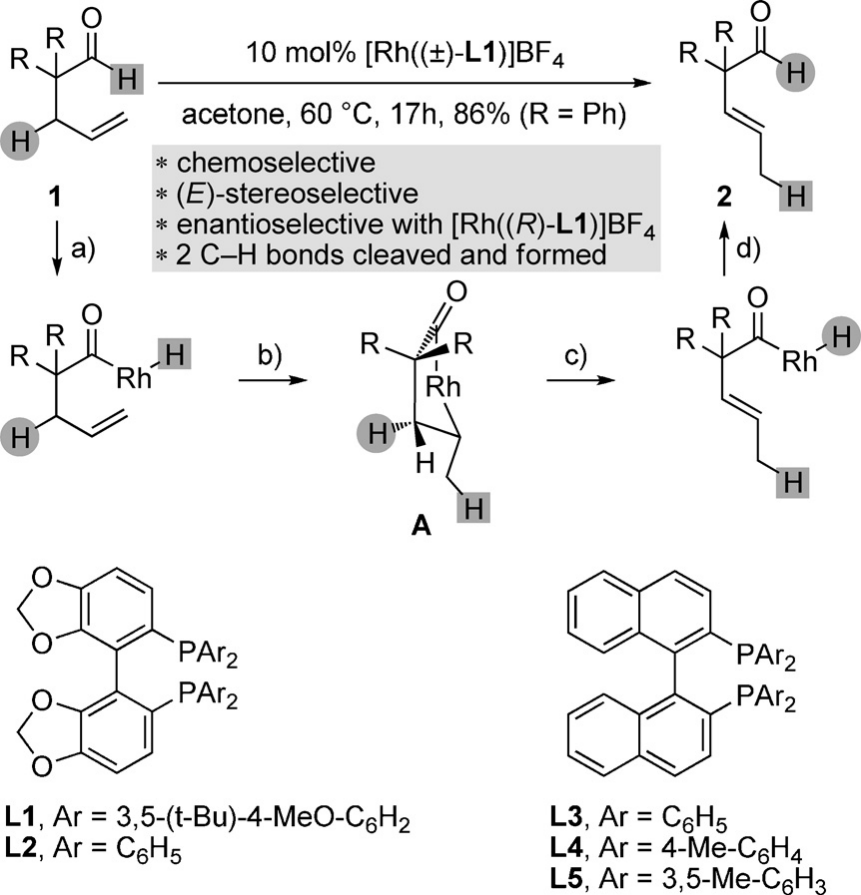
Rhodium-catalyzed isomerization of 4-pentenals into 3-pentenals by endocyclic β-H elimination. Ligands and charges are omitted for clarity: Rh=[Rh((±)-L1)]BF_4_. Yield of isolated product 2.

Herein, we report a thorough study of the reaction depicted in Figure 1, including deuterium-labeling experiments that support the postulated mechanism and the endocyclic β-H elimination of rhodacyclopentanone **A**. Moreover, we also show that the isomerization is chemoselective for olefins that enable the formation of **A**, and that olefins located elsewhere on the substrate remain intact under the reaction conditions, even in the challenging case of sensitive olefins normally prone to facile isomerization in the presence of transition-metal catalysts,[11] including rhodium catalysts.[12] Finally, we describe how the endocyclic β-H elimination of rhodacyclopentanones can be prevented, whereby each enantiomer of the racemic 4-pentenal undergoes a distinct and enantioselective rearrangement when treated with an enantiopure catalyst.

We found that the rhodium-catalyzed isomerization of deuterated substrates **3** and **4** into compounds **5** and **6**, respectively, occurred smoothly with complete transfer of the deuterium atom at the positions indicated in Scheme 1. Transient intermediate **3-int** was observed in the isomerization of **3** into **5**, indicating that step (b) in Figure 1 is reversible.[13] No intermolecular transfer of the deuterium atom was observed when **3** and **7** were treated with the rhodium catalyst. Instead, **5** and **8** were obtained in 75 % and 93 % yield, respectively. The results of these experiments are in good agreement with the intramolecular addition of an acylhydridorhodium intermediate and the endocyclic β-H elimination envisioned in Figure 1. In contrast to many precedents, neither the reversible intermolecular addition of a metal–hydride species[14] nor allylic C–H activation[15] can account for the olefin isomerization examined herein.

**Scheme 1:**
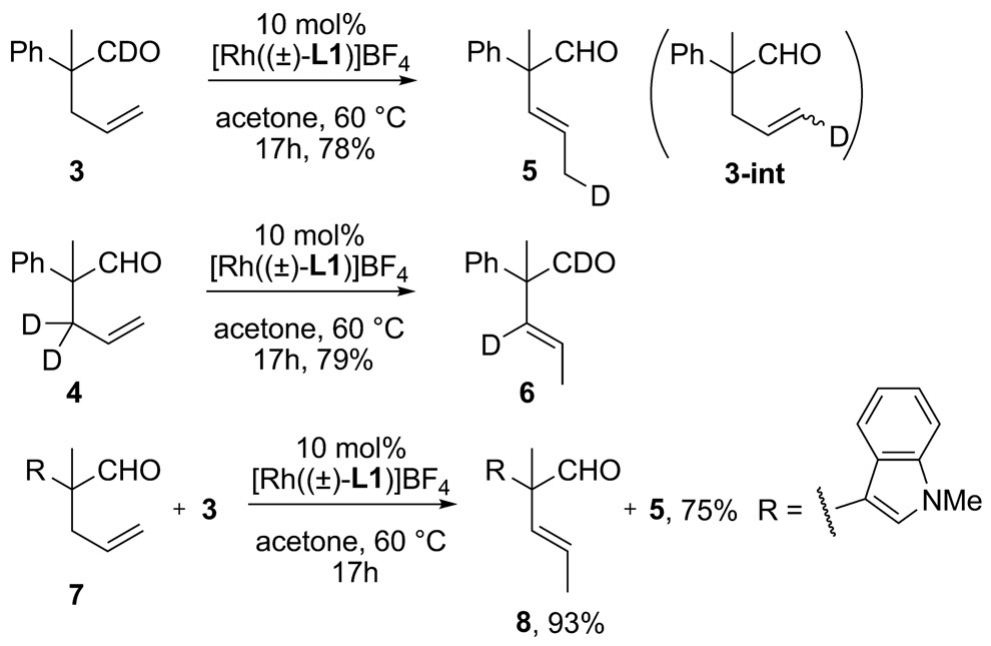
Deuterium-labeling experiments.

We then explored the generality of the isomerization of 4-pentenals into 3-pentenals with substrates **9 a**–**9 o** and observed in all cases the stereoselective formation of **10 a**–**10 o** as *trans* isomer only (Scheme 2). This exquisite *trans*-selectivity could be explained by the fact that the hydrogen atom highlighted by a gray disc in **A** (Figure 1) is the only one in this conformer of the five-membered metallacycle which is correctly positioned to develop an agostic interaction with the metal prior to β-H elimination.[5], [16] Hence, placing the *cis* isomer of **10 a** under the reaction conditions led to only limited isomerization into its *trans* isomer (*Z*/*E*=4.7:1). Monosubstituted **9 i** could be converted into **10 i**, albeit with decomposition owing to the instability of both **9 i** and **10 i**, even in the absence of catalyst.[17] Remarkably, other olefins, such as the remote terminal olefin in **10 k**, an allylbenzene (**10 l**), an allylic ether (**10 m**), an allylic amide (**10 n**), and a 1,4-enyne (**10 o**), remained unaffected by the active rhodium catalyst, although they are all susceptible to undergo metal-catalyzed isomerization.[11], [12]

**Scheme 2:**
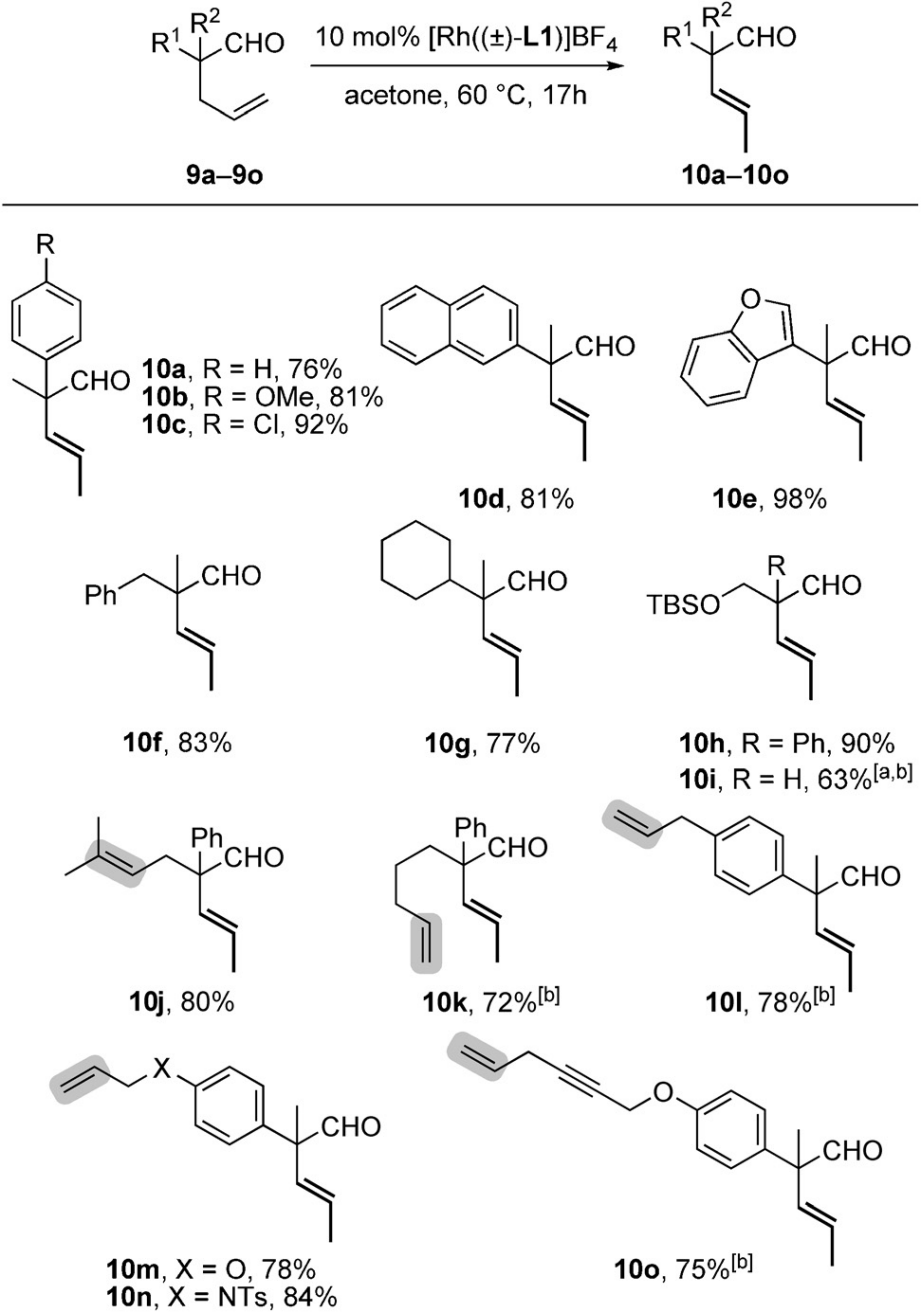
Scope of the chemo- and stereoselective isomerization of 4-pentenals into (*E*)-3-pentenals. All of the yields are given for isolated products as the average of two experiments; see the Supporting Information. [a] Yield determined by ^1^H NMR spectroscopy in [D_6_]acetone in the presence of an internal standard. [b] Room temperature. TBS=*tert*-butyldimethylsilyl. Ts=*p*-tolylsulfonyl.

The 1,1-disubstituted olefin in **11** does not undergo isomerization but sluggish intramolecular hydroacylation, and **12** was isolated in low yield besides the recovered starting material [Eq. (1)]. The isomerization of 1,2-bisubstituted olefin **13** into **14** is reversible and placing either **13** or **14** under the reaction condition leads to the same **13**/**14** ratio [Eq. (2)], although the reaction of **14** led to traces of decarbonylated olefins as well. In view of the inertness of **11** and the notoriously low yields of formation of α,α-disubstituted cyclopentanones in hydroacylation reactions,[7], [8] we were surprised to observe that the treatment of **15** with the active rhodium catalyst led to the isolation of **17** in 97 % yield as a single diastereomer, as confirmed by NOESY (Scheme 3). Importantly, compound **16** could be isolated as transient intermediate in the formation of racemic **17** at room temperature: at 50 % conversion, **16** accounts for 45 % of the mass balance. The strikingly different reactivity of **11** and **16** suggests that the 1,2-disubsituted olefin of **16** facilitates the observed intramolecular hydroacylation. This tandem reaction could also be observed with other substrates (see the Supporting Information).


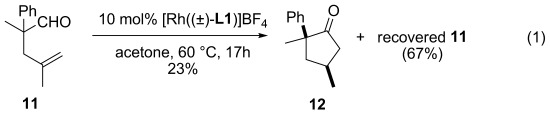
(1)


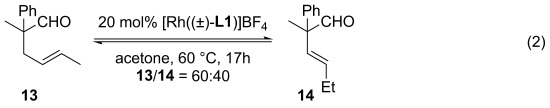
(2)

**Scheme 3:**
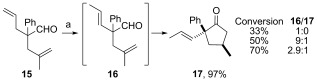
Olefin isomerization followed by intramolecular hydroacylation. a) [Rh((±)-L1)]BF_4_ (10 mol %), acetone, 60 °C, 17 h. The 16/17 ratios were obtained for the reaction performed at room temperature.

Further exploration of the scope of the reaction revealed that the isomerization of 4-pentenals into 3-pentenals can be prevented in favor of intramolecular hydroacylation. Thus, placing (±)-**18** under our optimized conditions led to cyclopentanone (±)-**19** and trace amounts of cyclobutanone (±)-**20** as single diastereomer [Eq. (3)]. Presumably, coordination by the methoxy group can prevent endocyclic β-H elimination by blocking a coordination site on the metal.


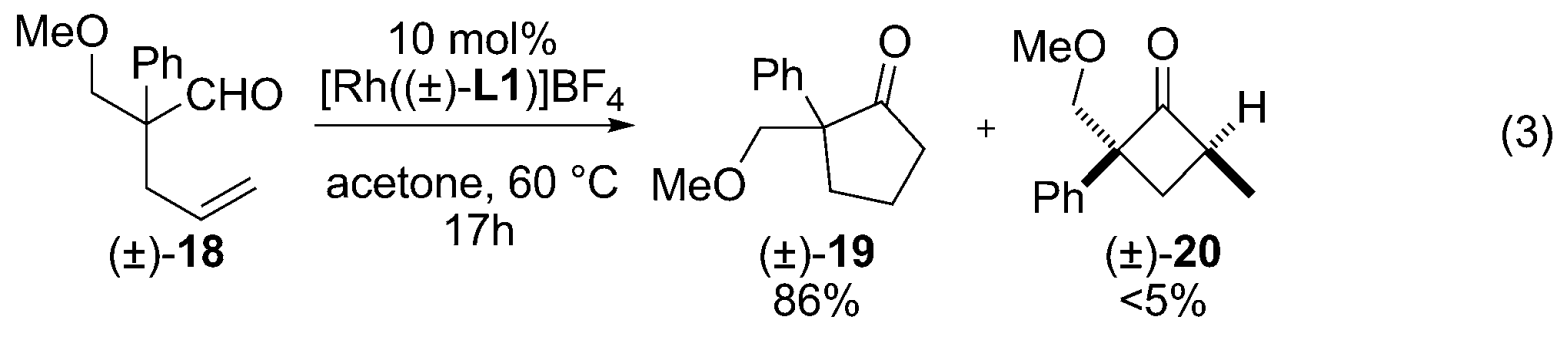
(3)

Considering the rare examples of simple kinetic resolution in the rhodium-catalyzed hydroacylation of 4-pentenals into cyclopentanones,[7b,c, 18] and parallel kinetic resolution of 4-alkynals,[19] we were curious to evaluate the effect of an enantiopure catalyst on the reaction of (±)-**18**. Using (*R*)-**L1** as ligand, we obtained (*R*)-**19** besides a mixture of (*S*,*S*)-**20** and (*S*)-**21** (Figure 2). Reduction of the latter compounds enabled the separation of (*R*)-**22** and the determination of its enantiomeric purity, which is assumed to be the same for (*S*)-**21**. The absolute stereochemistry of (*R*)-**19** was assigned by comparison with similar compounds,[20] and X-ray crystallography of ester (*S*)-**23** confirmed the configuration of (*S*)-**21**.[21] The absolute stereochemistry and enantiomeric purity of (*S*,*S*)-**20** were deduced by considering the mass balance and the enantiomeric ratios of the other products obtained in this reaction after full conversion, using the mathematical treatment proposed by Horeau.[22] Significantly, we observed that **19** is formed more quickly than **20** and **21**: at 10 % conversion, **19** accounts for more than 8 % of the mass balance whereas **20** and **21** account less than 2 % together. Accordingly, the behavior of (±)-**18** in the presence of an enantiopure catalyst is best described as a divergent reaction on a racemic mixture (RRM),[23] whereby each enantiomer follows predominantly a distinct reaction pathway, (*R*)-**18** leading to (*R*)-**19**, and (*S*)-**18** leading to (*S*,*S*)-**20** and (*S*)-**21**. In **B**, coordination of the metal by the oxygen atom lone pair of the methoxy group would prevent endocyclic β-H elimination, and **B** would instead undergo exocyclic β-H elimination and thereby revert to an acylhydridorhodium intermediate, which eventually would lead to (*R*)-**19** by intramolecular hydroacylation. In **C**, similar coordination by the methoxy group and the release of the steric repulsion between the pseudo-axial phenyl and methyl groups after reductive elimination would promote the formation of (*S*,*S*)-**20**. In **D**, the coordination site on the metal necessary to the endocyclic β-H elimination would remain available and this intermediate would eventually lead to (*S*)-**21**. To the best of our knowledge, the enantioselective reaction depicted in Figure 2 is the first example of a divergent RRM of 4-pentenals. Although the details of the interaction of (*R*)-**L1** with the substrates are not known, a preliminary investigation indicated that a simple kinetic resolution of (±)-**16** with [Rh((*R*)-**L1**)]BF_4_ (10 mol %) at room temperature led to the isolation of (−)-**17** in 50 % yield (e.r.=98:2) whilst (+)-**16** was recovered in 40 % yield (e.r.=99:1).

**Figure 2:**
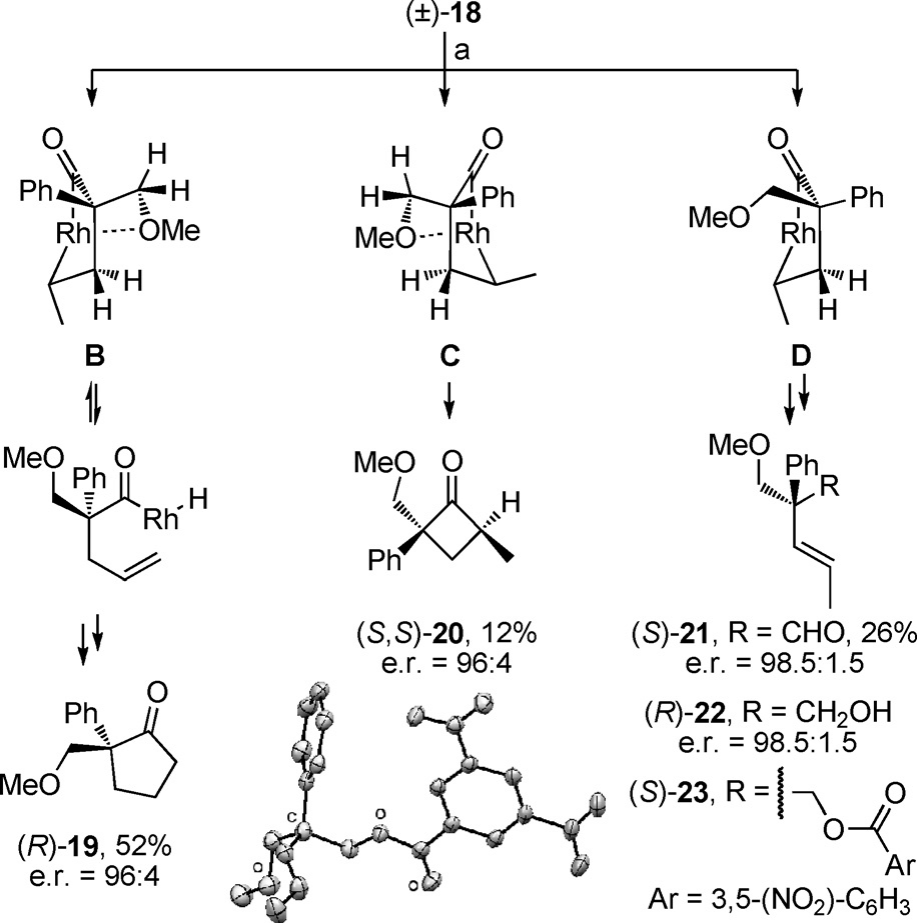
Divergent reaction on a racemic mixture. a) [Rh((*R*)-L1)]BF_4_ (10 mol %), acetone, room temperature, 17 h. Enantiomeric ratios (e.r.) of (*R*)-19 and (*R*)-22 were determined by chiral-phase HPLC. Ligands and charges are omitted for clarity: Rh=[Rh(*R*)-L1)]BF_4_. Ellipsoids are set at 50 % probability in the ORTEP of (*S*)-23.

In conclusion, we have identified several factors which control the behavior of a key five-membered metallacycle intermediate in the isomerization of 4-pentenals into 3-pentenals in terms of chemo- and stereoselectivity. Endocyclic β-H elimination of this intermediate enables the stereoselective formation of a *trans* olefin with exquisite control. Alternatively, this elementary step can be prevented by a coordinating group, in which case it is possible to observe a specific reaction for each enantiomer of the racemic 4-pentenal in the presence of an enantiopure catalyst.

## Supporting Information

As a service to our authors and readers, this journal provides supporting information supplied by the authors. Such materials are peer reviewed and may be re-organized for online delivery, but are not copy-edited or typeset. Technical support issues arising from supporting information (other than missing files) should be addressed to the authors.

miscellaneous_information
