# Supplementary material for: Isomerization of Olefins Triggered by Rhodium-Catalyzed C–H Bond Activation: Control of Endocyclic β-Hydrogen Elimination
Source: Angew Chem Int Ed Engl. 2015 Apr 23;54(23):6870–3. doi: 10.1002/anie.201500596 (PMC4497606; doi:10.1002/anie.201500596)

Angewandte Chemie
Eine Zeitschrift der Gesellschaft Deutscher Chemiker

Supporting Information

German Edition: DOI:

Isomerization of Olefins Triggered by Rhodium-Catalyzed C–H Bond Activation: Control of Endocyclic β-Hydrogen Elimination**

*Stephanie Y. Y. Yip and Christophe Aïssa**

anie_201500596_sm_miscellaneous_information.pdf

General. Otherwise noted, all reactions were carried out in flame-dried glassware under dry nitrogen atmosphere. THF and Et_2O were purchased dried from Acros. CH_2Cl_2 was dried over 4Å molecular sieves. Dry acetone was purchased from VWR. Flash chromatography: Merck silica gel 60 (230-400 mesh). NMR: Spectra were recorded on a Bruker DRX 500 in $CDCl_3$; chemical shifts (δ) are given in ppm. The solvent signals were used as references and the chemical shifts converted to the TMS scale ($CDCl_3$: δ_C = 77.0 ppm; residual $CHCl_3$ in $CDCl_3$: δ_H = 7.26 ppm); apparent splitting patterns are designated using the following abbreviations: s (singlet), d (doublet), t (triplet), q (quartet), quint. (quintuplet), m (multiplet), br (broad), and the appropriate combinations. In ^{13}C NMR, an APT sequence was used to separate methylene groups and quaternary carbons (e, even) from methine and methyl groups (o, odd). IR: PerkinElmer Spectrum 100 FT-IR spectrometer, wavenumbers ($\tilde{\nu}$) in cm^{-1}. HRMS determined at the University of Liverpool on micromass LCT mass spectrometer (ES+) and Trio-1000 or Agilent QTOF 7200 mass spectrometers (CI). Melting points: Griffin melting point apparatus (not corrected). Elemental analyses: University of Liverpool. X-Ray crystallography: Bruker D8 Venture Photon 100 Dual Microsource diffractometer. Optical rotations were measured on a PerkinElmer Model 343 plus polarimeter with a sodium lamp (D line, 589 nm) at ambient temperature (indicated in ºC as superscript) using a 1 mL quartz cell of 100 mm length; solution concentration (c) are given in g/100 mL. All commercially available compounds were used as received.

Preparation of compounds **1**, **3**, **4**, **7**, and **9a**–**9o**	S2
Preparation of compounds **11**, **13**, **15** and **18**	S25
Preparation of compound ***cis*-10a**	S28
Preparation of [Rh(nbd)(DTBM-Segphos)]	S31
Representative procedure for Rh-catalysed isomerization	S32
Analytical data for compounds **2**, **5**, **6**, **8**, and **10a**–**10o**	S33
Analytical data for compounds **12** and **14**	S37
Preparation of compound **17** and isolation of transient intermediate **16**	S38
Supplementary examples of isomerization/hydroacylation tandem	S39
Preparation of compounds **(±)-19**, **(*R*)-19**, **(±)-20**, **(*S*,*S*)-20**, **(*S*)-21**, **(*R*)-22**, and **(*S*)-23**	S41
Calculation of e.e. of **(*S*,*S*)-20** and determination of its absolute configuration	S43
Kinetic resolution of **(±)-16**	S44
NMR spectra for compounds **1**, **3**, **4**, **7**, and **9a**–**9o**	S45
NMR spectra for compounds **11**, **13**, **15** and **18**	S83
NMR spectra for compounds **2**, **5**, **6**, **8**, and **10a**–**10o**	S93
NMR spectra for compounds **12**, **14**, **16**, **17**, **19**–**23**	S135
HPLC traces for compounds **19** and **22**	S166
HPLC traces for compounds **17** and **SI-3**	S170
Crystal data and structure refinement for (*S*)-**23**	S178

Preparation of compounds 1, 3, 4, 7, and 9a–9o[1]

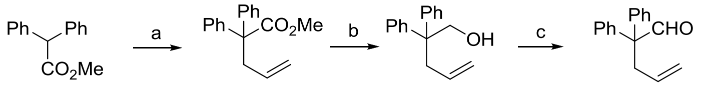

1a 1b 1

[(a)] i) LDA, THF, -78 °C; ii) allyl bromide, nBu_4NI, -78 °C to rt, 99%. [(b)] $LiAlH_4$, Et_2O, 90%. [(c)] $(COCl)_2$, DMSO, Et_3N, CH_2Cl_2, 81%.

Compound 1a. Under N_2 atmosphere, di-isopropylamine (7.29 mmol, 1.03 mL) was dissolved in THF (22 mL) and cooled to 0 °C. nButyllithium (7.29 mmol, 2.92 mL, 2.5M in hexanes) was added and stirred for 15 minutes at 0 °C before cooling to −78 °C and adding diphenyl methyl acetate (6.63 mmol, 1.5 g), stirring for 30 minutes at −78 °C. Then, allyl bromide (7.29 mmol, 631 µL) and tetrabutylammonium iodide (1.33 mmol, 490 mg) were added and the reaction mixture was warmed to room temperature and stirred for 16 hours. The reaction mixture was cooled to 0 °C, quenched carefully with a saturated aqueous solution of ammonium chloride and the aqueous layer was extracted with Et_2O. The organic phase was dried over $MgSO_4$ and the solvent removed under reduced pressure. Purification by flash column chromatography (petroleum ether/Et_2O = 50:1) afforded **1a** (1.74 g, 99%) as a colourless oil. ^1H NMR (500 MHz, $CDCl_3$): δ = 7.33-7.22 (m, 10H), 5.59 (ddt, *J* = 17.6, 9.7, 7.0 Hz, 1H), 4.97-4.90 (m, 2H), 3.70 (s, 3H), 3.17 (dt, *J* = 1.2 Hz, 2H), in agreement with previously reported data.[2]

Compound 1b. A solution of **1a** (5.63 mmol, 1.5 g) in Et_2O (12 mL) was added under N_2 to a suspension of $LiAlH_4$ (3.10 mmol, 133 mg) in Et_2O (26 mL) at 0 °C. After stirring at room temperature for 30 minutes, another portion of $LiAlH_4$ (3.10 mmol, 133 mg) was added. After stirring for 30 minutes, the reaction mixture was quenched carefully at 0 °C with a saturated aqueous solution of sodium sulfate. The white precipitate was filtered over celite pad, the filtrate was evaporated under reduced pressure and the crude material was purified by flash chromatography (petroleum ether/Et_2O = 10:1) to afford **1b** as colourless oil (1.20 g, 90%). ^1H NMR (500 MHz, $CDCl_3$): δ = 7.33-7.28 (m, 4H), 7.25-7.17 (m, 6H), 5.43 (ddt, *J* = 17.2, 10.1, 7.1 Hz, 1H), 5.13-5.07 (m, 1H), 5.02-4.97 (m, 1H), 4.16 (d, *J* = 6.8 Hz, 2H), 2.97 (d, *J* = 7.2 Hz, 2H), 1.14 (t, *J* = 6.9 Hz, 1H (OH)), in agreement with previously reported data.[3]

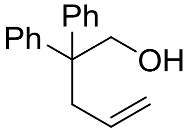

1b

Compound 1. Under N_2, DMSO (6.05 mmol, 429 µL) in CH_2Cl_2 (1 mL) was added to a solution of oxalyl chloride (3.02 mmol, 259 µL) in CH_2Cl_2 (20 mL) at –78 °C. After 10 minutes stirring at –78 °C, a solution of the **1b** (2.52 mmol) in CH_2Cl_2 (4 mL) was added. After 20 minutes stirring at –78 °C, triethylamine (12.6 mmol, 1.76 mL) was added and the mixture was stirred at room temperature during 20 minutes. A saturated solution of ammonium chloride was added to the reaction mixture which was then extracted three times with diethyl ether. The organic layer was washed with water and brine, dried over Na_2SO_4, filtered, and concentrated. The crude mixture was purified by flash chromatography (petroleum ether/Et_2O = 50:1) to afford **1** as colourless oil (481 mg, 81%). ^1H NMR (500 MHz, $CDCl_3$): δ = 9.83 (s, 1H), 7.39-7.34 (m, 4H), 7.33-7.27 (m, 2H), 7.21-7.17 (m, 4H), 5.58 (ddt, *J* = 17.2, 10.1, 7.0 Hz, 1H), 5.01-4.92 (m, 2H), 3.09 (d, *J* = 7.1 Hz, 1H); ^{13}C NMR (125 MHz, $CDCl_3$): δ = 198.3 (o), 139.6 (e, 2C), 133.5 (o), 129.1 (o, 4C), 128.6 (o, 4C), 127.3 (o, 2C), 118.4 (e), 63.4 (e), 38.8 (e); IR (neat): $\tilde{\nu}$ = 3060 (w), 3025 (w), 2917 (w), 2816 (w), 2716 (w), 1721 (s), 1640 (w), 1599 (w), 1493 (m), 1445 (m), 1389 (w), 1282 (w), 1189 (w), 1159 (w), 1097 (w), 1084 (w), 1033 (w), 998 (w), 953 (w), 916 (m), 870 (w),

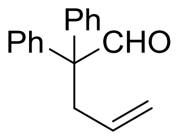

1

---

[1] **9j** is a known compound, see: C. Aïssa, K. Y-T. Ho, D. J. Tetlow, M. Pin-Nó, *Angew. Chem. Int. Ed* **2014**, 53, 4209
[2] M. Mitsuno, M. Kadokena, Y. Watanabe, *J. Org. Chem.* **1987**, *52*, 1695
[3] Y.-K. Jeong, D.-Y. Kim, Y.-S. Choi, J.-S. Ryu, *Org. Biomol. Chem.* **2011**, 374

846 (w), 755 (m), 697 (vs), 659 (w) cm^{-1}; elemental analysis (%) calcd for $C_{17}H_{16}O$: C 86.40, H 6.82; found: C 85.95, H 6.74.

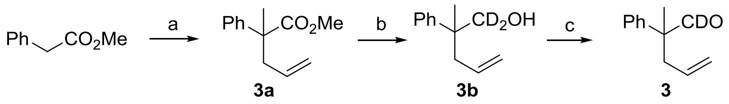

[(a)] i) LDA, THF, -78 °C; ii) allyl bromide, nBu_4NI, -78 °C to rt, iii) LDA, THF, -78 °C; iv) MeI, 96%. [(b)] $LiAlD_4$, Et_2O, 89%. [(c)] $(COCl)_2$, DMSO, Et_3N, CH_2Cl_2, 78%.

Compound 3a. Under N_2 atmosphere, diisopropylamine (11.62 mmol, 1.6 mL) was dissolved in THF (32 mL) and cooled to 0 °C. *n*Butyllithium (11.62 mmol, 4.5 mL, 2.5M in hexanes) was added and the mixture was stirred for 15 minutes. The mixture was cooled to –78 °C before adding the intermediate (9.68 mmol, 1.84 g) obtained from methyl phenyl acetate (9.99 mmol, 1.44 mL) using the allylation procedure described for the preparation of **1a**. After stirring for 30 minutes at –78 °C, methyl iodide (15.48 mmol, 943 µL) was added and the reaction was left to stir over 48 hours at room temperature before quenching with a saturated aqueous solution of ammonium chloride and the aqueous layer was extracted with Et_2O. The organic phase was dried over $MgSO_4$ and the solvent removed under reduced pressure. Purification by flash column chromatography (petroleum ether/Et_2O = 50:1) afforded **3a** as a colourless oil (1.97 g, 96% over two steps); ^{1}H NMR (500 MHz, $CDCl_3$): δ = 7.36-7.28 (m, 4H), 7.26-7.21 (m, 1H), 5.61 (ddt, *J* = 17.1, 10.0, 7.2 Hz, 1H), 5.11-5.01 (m, 2H), 3.66 (s, 3H), 2.83 (dd, *J* = 13.7, 7.4 Hz, 1H), 2.66 (dd, *J* = 13.7, 7.1 Hz, 1H), 1.53 (s, 3H); ^{13}C NMR (125 MHz, $CDCl_3$): δ = 176.2 (e), 143.3 (e), 134.0 (o), 128.3 (o, 2C), 126.7 (o), 125.9 (o, 2C), 118.3 (e), 52.0 (o), 49.9 (e), 43.7 (e), 22.6 (o); IR (neat) $\tilde{\nu}$ = 3062 (w), 2979 (w), 2950 (w), 1728 (vs), 1640 (w), 1600 (w), 1583 (w), 1496 (m), 1459 (m), 1446 (m), 1433 (m), 1378 (w), 1317 (w), 1286 (w), 1272 (w), 1231 (m), 1142 (s), 1103 (m), 1072 (w), 1030 (w), 995 (m), 956 (w), 916 (m), 851 (w), 787 (w), 767 (m), 735 (m), 697 (s), 659 (w) cm^{-1}; HRMS (CI(NH_4)) calcd for ($C_{13}H_{16}O_2$ + H): 205.1123; found: 205.1128.

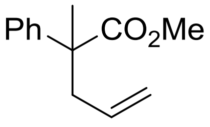

3a

Compound 3b. This compound was obtained from **3a** (1.96 mmol, 400 mg) following the same procedure as for the preparation of **1b** but using $LiAlD_4$ (2.15 mmol, 90mg). Colourless oil (314 mg, 89%). ^{1}H NMR (500 MHz, $CDCl_3$): δ = 7.39-7.33 (m, 4H), 7.25-7.21 (m, 1H), 5.60 (ddt, *J* = 17.0, 10.1, 7.26 Hz, 1H), 5.08-5.02 (m, 1H), 5.01-4.97 (m, 1H), 2.56 (dd, *J* = 13.9, 6.6 Hz, 1H), 2.36 (dd, *J* = 13.9, 7.9 Hz, 1H), 1.34 (s, 3H), 1.20 (br s, 1H (OH)); ^{13}C NMR (125 MHz, $CDCl_3$): δ = 144.6 (e), 134.4 (o), 128.2 (o, 2C), 126.6 (o, 2C), 126.0 (o), 117.3 (e), 70.7 (e, *J* = 22.1 Hz, quint.), 42.8 (e), 31.5 (e), 21.6 (o); IR (neat): $\tilde{\nu}$ = 3377 (br), 3059 (w), 3023 (w), 2975 (w), 2913 (w), 2203 (w), 2086 (w), 1638 (w), 1601 (w), 1580 (w), 1496 (m), 1444 (m), 1415 (w), 1375 (w), 1289 (w), 1155 (w), 1098 (m), 1078 (w), 1048 (w), 1028 (m), 996 (w), 976 (m), 911 (s), 844 (w), 788 (w), 758 (m), 697 (vs), 664 (w) cm^{-1}; HRMS (CI(NH_4)) calcd for ($C_{12}H_{14}D_2O$ + NH_4): 196.1665; found: 196.1668.

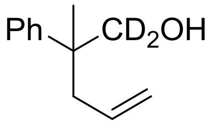

3b

Compound 3. This compound was obtained from **3b** (1.69 mmol, 300 mg) following the same procedure as for the preparation of **1**. Colourless oil (231 mg, 78%). ^{1}H NMR (500 MHz, $CDCl_3$): δ = 7.42-7.36 (m, 2H), 7.32-7.23 (m, 3H), 5.55 (dddd, *J* = 17.2, 10.1, 7.3, 7.1 Hz, 1H), 5.06 (ddt, *J* = 16.8, 1.9, 1.4 Hz, 1H), 5.03 (ddt, *J* = 10.1, 2.0, 1.0 Hz, 1H), 2.70 (ddt, *J* = 14.1, 6.8, 1.2 Hz, 1H), 2.63 (ddt, *J* = 14.1, 7.7, 1.1 Hz, 1H), 1.45 (s, 3H); ^{13}C NMR (125 MHz, $CDCl_3$): δ = 201.4 (e, *J* = 26.7 Hz, t), 139.4 (e), 133.1 (o), 128.9 (o, 2C), 127.2 (o), 127.0 (o, 2C), 118.4 (e), 53.3 (e, *J* = 3.3 Hz, t), 40.4 (e), 18.7 (o); IR (neat): $\tilde{\nu}$ = 3077 (w), 3026 (w), 2978 (w), 2934 (w), 2103 (w), 2052 (w), 1713 (s), 1640 (m), 1599 (w), 1581 (w), 1494 (m), 1445 (m), 1417 (w), 1376 (w), 1271 (w), 1158 (w), 1077 (w), 1046 (w), 1028 (w), 995 (m), 955 (w), 917 (m), 848 (w), 803 (w), 755 (m), 729 (w), 697 (vs) cm^{-1}; HRMS (CI(NH_4)) calcd for ($C_{12}H_{13}DO$ + NH_4): 193.1446; found: 193.1448.

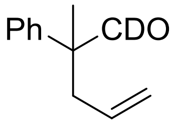

3

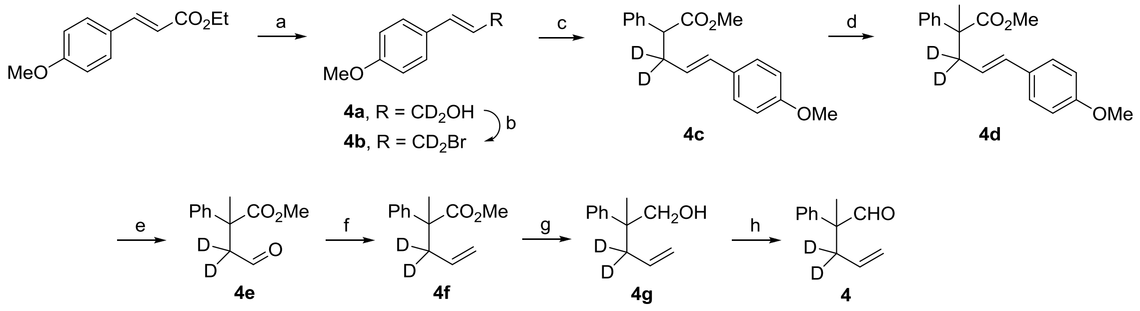

[(a)] $LiAlD_4$, Et_2O, 76%. [(b)] PBr_3, Et_2O. [(c)] i) LDA, THF, -78 °C; ii) **4b**, -78 °C to rt, 48% over two steps. [(d)] i) LDA, THF, -78 °C; ii) MeI, 94%. [(e)] i) O_3, CH_2Cl_2 ; ii) PPh_3. [(f)] i) Methyl-triphenylphosphonium bromide, nBuLi; ii) **4e**, 29% over two steps. [(g)] $LiAlH_4$, Et_2O, 85%. [(h)] $(COCl)_2$, DMSO, Et_3N, CH_2Cl_2, 82%.

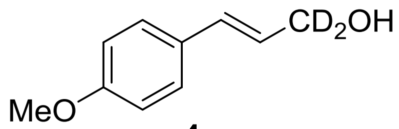

4a

Compound 4a. This compound was obtained from ethyl 4-methoxycinnamate (9.7 mmol, 2.0 g) following the same procedure as for the preparation of **1b** but using $LiAlD_4$ (10.7 mmol, 450 mg). White solid (1.22 g, 76%). m.p.: 70–71 °C; ^{1}H NMR (500 MHz, $CDCl_3$): δ = 7.36-7.30 (m, 2H), 6.89-6.83 (m, 2H), 6.56 (d, J = 15.9 Hz, 1H), 6.24 (d, J = 15.9 Hz, 1H), 3.81 (s, 3H), 1.35 (br s, 1H (OH)); IR (neat): $\tilde{\nu}$ = 3356 (br), 3033 (w), 2970 (w), 2917 (w), 2843 (w), 2165 (w), 2194 (w), 2080 (w), 1651 (w), 1605 (m), 1510 (s), 1458 (m), 1443 (m), 1421 (m), 1306 (m), 1272 (s), 1242 (s), 1210 (m), 1190 (w), 1174 (s), 1144 (m), 1108 (w), 1075 (m), 1024 (s), 991 (w), 969 (s), 953 (s), 935 (m), 917 (w), 908 (w), 870 (w), 830 (s), 822 (vs), 772 (m), 743 (w), 734 (w), 693 (w) cm^{-1}; elemental analysis (%) calcd for $C_{10}H_{10}D_2O_2$: C 72.26, H 7.28; found: C 71.94, H 7.28.

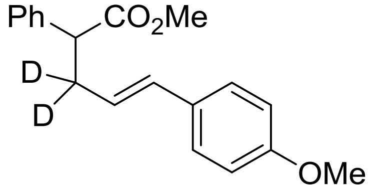

4c

Compound 4c. Under N_2 atmosphere, **4a** (0.482 mmol, 80 mg) was dissolved in Et_2O (4.8 mL) and the solution cooled to 0 °C. Phosphorus tribromide (0.241 mmol, 23 μL) was added and stirred to room temperature for 1 hour. The reaction was quenched with brine, extracted with Et_2O (2 × 20 mL), and the organic layer dried over $MgSO_4$, filtered and concentrated afforded **4b** as a white solid (88 mg, 80%), which was used directly in the next step without further purification. Under a N_2 atmosphere, diisopropylamine (0.383 mmol, 54 μL) was dissolved in THF (1 mL) and cooled to 0 °C. *n*Butyllithium (0.383 mmol, 153 μL, 2.5M in hexanes) was added and stirred for 15 minutes at 0 °C before cooling to –78 °C and adding methyl phenyl acetate (0.348 mmol, 50 μL), stirring for 30 minutes at –78 °C. At –78 °C, **4b** (0.383 mmol, 88 mg) was added and the reaction mixture was warmed to room temperature and stirred for 16 hours. The reaction mixture was cooled to 0 °C, diluted with Et_2O and quenched carefully with brine. The organic phase was dried over $MgSO_4$ and the solvent removed under reduced pressure. Purification by flash column chromatography (petroleum ether/Et_2O = 30:1) afforded **4c** as a white solid (61 mg, 59%). m.p.: 60–62 °C; ^{1}H NMR (500 MHz, $CDCl_3$): δ = 7.38-7.19 (m, 7H), 6.84-6.78 (m, 2H), 6.38 (d, J = 15.8 Hz, 1H), 5.95 (d, J = 15.8 Hz, 1H), 3.79 (s, 3H), 3.68 (s, 1H), 3.66 (s, 3H); ^{13}C NMR (125 MHz, $CDCl_3$): δ = 173.9 (e), 158.9 (e), 138.6 (e), 131.6 (o), 130.2 (e), 128.7 (o, 2C), 127.9 (o, 2C), 127.3 (o), 127.2 (o, 2C), 124.6 (o), 113.9 (o, 2C), 55.3 (o), 52.0 (o), 51.8 (o), 36.3 (e, J = 19.5 Hz, quint.); IR (neat): $\tilde{\nu}$ = 2952 (w), 2840 (w), 1727 (vs), 1608 (m), 1578 (w), 1509 (s), 1454 (m), 1435 (m), 1335 (m), 1309 (w), 1277 (m), 1242 (vs), 1200 (s), 1172 (vs), 1152 (m), 1127 (m), 1108 (w), 1075 (w), 1032 (s), 987 (m), 966 (s), 867 (w), 826 (m), 808 (m), 766 (m), 740 (m), 729 (m), 699 (s) cm^{-1}; HRMS (ESI) calcd for ($C_{19}H_{18}D_2O_3$ + Na): 321.1436; found: 321.1429; elemental analysis (%) calcd for $C_{19}H_{18}D_2O_3$: C 76.48, H 6.76; found: C 75.99, H 6.78.

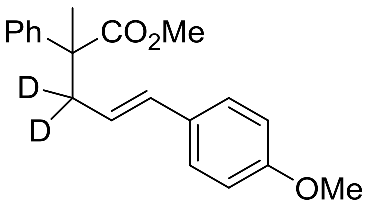

4d

Compound 4d. Under N_2 atmosphere, diisopropylamine (1.24 mmol, 176 µL) was dissolved in THF (3.5 mL) and cooled to 0 °C. *n*Butyllithium (1.24 mmol, 498 µL, 2.5M in hexanes) was added and stirred for 15 minutes. Reaction was cooled to –78 °C before adding methyl **4c** (1.04 mmol, 300 mg) stirring for 30 minutes at –78 °C. Methyl iodide (1.66 mmol, 103 µL) was added at –78 °C and the reaction was left to stir for 90 minutes before quenching with a saturated aqueous solution of ammonium chloride. The aqueous layer was extracted twice with Et_2O. The combined organic layers were dried over $MgSO_4$ and the solvent removed under reduced pressure. Purification by flash column chromatography (petroleum ether/Et_2O = 19:1) afforded **4d** as a white solid (295 mg, 94%). m.p.: 73–75 °C; ^{1}H NMR (500 MHz, $CDCl_3$): δ = 7.38-7.31 (m, 4H), 7.29-7.19 (m, 3H), 6.84-6.79 (m, 2H), 6.35 (d, *J* = 15.8 Hz, 1H), 5.85 (d, *J* = 15.7 Hz, 1H), 3.79 (s, 3H), 3.67 (s, 3H), 1.57 (s, 3H); ^{13}C NMR (125 MHz, $CDCl_3$): δ = 176.3 (e), 158.8 (e), 143.3 (e), 132.8 (o), 130.2 (e), 128.4 (o, 2C), 127.2 (o, 2C), 126.8 (o), 125.9 (o, 2C), 123.3 (o), 113.8 (o, 2C), 55.2 (o), 52.1 (o), 50.3 (e), 42.3 (e, *J* = 20.6 Hz, quint), 22.6 (o); IR (neat): $\tilde{\nu}$ = 3008 (w), 2953 (w), 2836 (w), 1723 (s), 1608 (m), 1579 (w), 1510 (m), 1494 (m), 1457 (w), 1442 (m), 1457 (w), 1380 (w), 1311 (w), 1230 (m), 1248 (vs), 1189 (m), 1177 (m), 1129 (s), 1110 (w), 1074 (w), 1038 (m), 976 (m), 896 (w), 867 (w), 837 (m), 809 (m), 775 (m), 763 (m), 729 (m), 698 (m) cm^{-1}; HRMS (ESI) calcd for ($C_{20}H_{20}D_2O_3$ + Na): 335.1592; found: 335.1590; elemental analysis (%) calcd for $C_{20}H_{20}D_2O_3$: C 76.89, H 7.10; found: C 76.76, H 7.20.

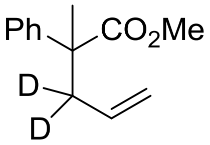

4f

Compound 4f. Intermediate **4d** (3.2 mmol, 1.0 g) was dissolved in CH_2Cl_2 (32 mL) and cooled to –78 °C. Ozone was bubbled through the solution using a pipette for 20 minutes (light blue colour change) until reaction was complete by TLC. Triphenyl phosphine (4.8 mmol, 1.26 g) was added to the solution and compressed air was bubbled through the solution for 15 minutes whilst the reaction is allowed to warm to room temperature. The solution was concentrated and purified by flash column chromatography (petroleum ether/Et_2O = 19:1) to afford a colourless oil (678 mg, 68%) as a 2:1 ratio mixture of **4e** and 4-methoxybenzaldehyde which was used as this purity for the next step. Under N_2 atmosphere, nbutyllithium (4.22 mmol, 1.7 mL, 2.5 M in hexanes) was added to methyltriphenylphosphonium bromide (4.22 mmol, 1.5 g) in THF (25 mL) at –78 °C. the reaction mixture was warmed to 40 °C for 15 minutes until white solid was dissolved, before cooling to –78 °C and adding a solution of **4e** (3.84 mmol, 678 mg) in THF (13 mL) *via* cannula. The mixture was heated to 40 °C for 18 hours before being quenched at room temperature with a saturated aqueous solution of ammonium chloride and extracted with Et_2O (2 × 30 mL). The combined organic layers were dried over $MgSO_4$, filtered and concentrated. The crude mixture was purified by flash column chromatography (petroleum ether/Et_2O = 99:1) afforded **4f** as a colourless oil (190 mg, 43%). ^{1}H NMR (500 MHz, $CDCl_3$): δ = 7.37-7.28 (m, 4H), 7.26-7.22 (m, 1H), 5.60 (dd, *J* = 17.0, 10.2 Hz, 1H), 5.11-5.04 (m, 2H), 3.66 (s, 3H), 1.53 (s, 3H); ^{13}C NMR (125 MHz, $CDCl_3$): δ = 176.3 (e), 143.3 (e), 133.9 (o), 128.4 (o, 2C), 126.8 (o), 125.9 (o, 2C), 118 (e), 52.1 (o), 49.8 (e), 43.0 (e, *J* = 19.8 Hz, quint.), 22.6 (o); IR (neat): $\tilde{\nu}$ = 2978 (w), 2951 (w), 1731 (vs), 1637 (w), 1600 (w), 1497 (w), 1446 (w), 1378 (w), 1249 (m), 1166 (w), 1126 (m), 1079 (w), 1055 (w), 1031 (w), 1001 (w), 920 (w), 767 (w), 733 (w), 699 (m); cm^{-1}; HRMS (CI(NH_4)) calcd for ($C_{13}H_{14}D_2O_2$ + H): 207.1333; found: 207.1347; elemental analysis (%) calcd for $C_{13}H_{14}D_2O_2$: C 75.75, H 7.82; found: C 75.93, H 7.84.

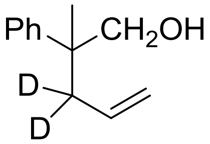

4g

Compound 4g. This compound was obtained from **4f** (0.922 mmol, 190 mg) following the same procedure as for the preparation of **1b**. Colourless oil (140 mg, 85%). ^{1}H NMR (500 MHz, $CDCl_3$): δ = 7.40-7.33 (m, 4H), 7.26-7.21 (m, 1H), 5.59 (dd, *J* = 17.1, 10.2 Hz, 1H), 5.05 (dd, *J* = 17.1, 2.2 Hz, 1H), 5.00 (dd, *J* = 10.1, 2.2 Hz, 1H), 3.75 (dd, *J* = 11.0, 5.6 Hz, 1H), 3.60 (dd, *J* = 10.9, 7.4 Hz, 1H), 1.34 (s, 3H), 1.32-1.22 (m, 1H (OH)); ^{13}C NMR (125 MHz, $CDCl_3$): δ = 144.5, 134.4, 128.4 (2C), 126.6 (2C), 126.2, 117.6, 71.7, 43.0, 42.1 (*J* = 19.7 Hz, quint.), 21.7; IR (neat): $\tilde{\nu}$ = 3358 (br), 3059 (w), 3025 (w), 2973 (w), 2934 (w), 2875 (w), 1635 (w), 1601 (w), 1581 (w), 1497 (m), 1445 (m), 1413 (w), 1374 (w),

1277 (w), 1193 (w), 1133 (w), 1025 (s), 1001 (m), 950 (wq), 914 (m), 872 (w), 842 (w), 759 (m), 738 (w), 698 (vs) cm^{-1}; elemental analysis (%) calcd for $C_{12}H_{14}D_2O$: C 80.85, H 9.04; found: C 81.17, H 9.27.

Compound 4. This compound was obtained from **4g** (0.56 mmol, 100mg) following the same procedure as for the preparation of **1**. Colourless oil (80 mg, 82%).

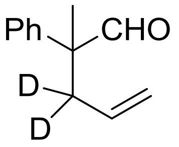

4

^{1}H NMR (500 MHz, $CDCl_3$): δ = 9.52 (s, 1H), 7.42-7.36 (m, 2H), 7.32-7.27 (m, 1H), 7.26-7.23 (m, 2H), 5.53 (dd, *J* = 17.0, 10.2 Hz, 1H), 5.06 (dd, *J* = 16.8, 2.0 Hz, 1H), 5.03 (dd, *J* = 9.8, 2.0 Hz, 1H), 1.44 (s, 3H); ^{13}C NMR (125 MHz, $CDCl_3$): δ = 202.0, 139.3, 133.1, 128.8 (2C), 127.3, 127.2 (2C), 118.7, 53.5, 39.8 (*J* = 20.1 Hz, quint.), 18.7; IR (neat): $\tilde{\nu}$ = 3061 (w), 2977 (w), 2935 (w), 2805 (w), 2706 (w), 1723 (vs), 1637 (w), 1599 (w), 1582 (w), 1494 (m), 1445 (m), 1414 (w), 1389 (w), 1372 (w), 1229 (w), 1192 (w), 1129 (w), 1060 (w), 1029 (m), 1000 (m), 918 (m), 759 (m), 699 (s) cm^{-1}; HRMS (CI(NH_4)) calcd for ($C_{12}H_{12}D_2O$ + NH_4): 194.1503; found: 194.1508; elemental analysis (%) calcd for $C_{12}H_{12}D_2O$: C 81.77, H 8.01; found: C 81.95, H 7.81.

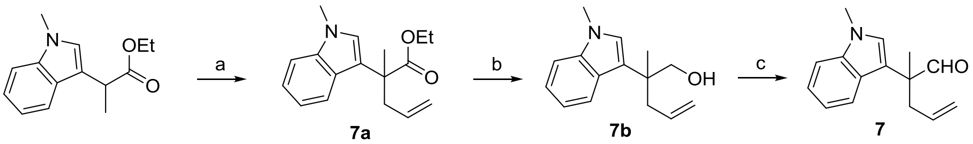

[(a)] i) NaHMDS, DMF, 0 °C; ii) allyl bromide, 0 °C to rt, 50%. [(b)] $LiAlH_4$, Et_2O, 91%. [(c)] $(COCl)_2$, DMSO, Et_3N, CH_2Cl_2, 96%.

Compound 7a. Under N_2 atmosphere, Ethyl 2-(1-methylindol-3-yl)propionate (0.49 mmol, 113 mg) and Sodium bis(trimethylsilyl)amide (0.54 mmol, 99 mg) were dissolved in DMF (2.5 mL) and cooled to 0 °C and stirred for 30 minutes. Allyl bromide (0.54 mmol, 47 µL) was added and the reaction mixture was warmed to room temperature and stirred for 48 hours. The reaction mixture was cooled to 0 °C, diluted with Et_2O and quenched carefully with a saturated aqueous solution of ammonium chloride. The organic layer was washed with water (2 × 10 mL), dried over $MgSO_4$ and the solvent was removed under reduced pressure. Purification by flash column chromatography (petroleum ether/Et_2O = 15:1) afforded **7a** as a colourless oil (67 mg, 50%).

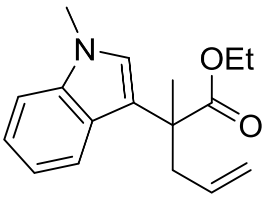

7a

^{1}H NMR (500 MHz, $CDCl_3$): δ = 7.68 (d, *J* = 8.1 Hz, 1H), 7.30-7.26 (m, 1H), 7.23-7.18 (m, 1H), 7.09-7.05 (m, 1H), 6.92 (s, 1H), 5.67 (ddt, *J* = 17.1, 10.0, 7.2 Hz, 1H), 5.11-5.05 (m, 1H), 5.05-5.01 (m, 1H), 4.18–4.07 (m, 2H), 3.76 (s, 3H), 2.95 (dd, *J* = 13.7, 7.5 Hz, 1H), 2.83 (dd, *J* = 13.7, 6.9 Hz, 1H), 1.61 (s, 3H), 1.16 (t, *J* = 7.1 Hz, 3H); ^{13}C NMR (125 MHz, $CDCl_3$): δ = 176.0 (e), 137.4 (e), 134.4 (o), 126.0 (e), 125.9 (o), 121.5 (o), 120.5 (o), 118.8 (o), 117.9 (e), 117.7 (e), 109.3 (o), 60.7 (e), 45.5 (e), 42.6 (e), 32.7 (o), 22.8 (o), 14.2 (o); IR (neat): $\tilde{\nu}$ = 3051 (w), 2978 (w), 2935 (w), 1720 (s), 1640 (w), 1615 (w), 1545 (w), 1484 (m), 1465 (m), 1425 (w), 1372 (m), 1330 (m), 1279 (w), 1233 (m), 1207 (m), 1172 (w), 1137 (m), 1096 (m), 1065 (w), 1018 (m), 993 (w), 955 (w), 916 (m), 858 (w), 809 (w), 766 (w), 737 (vs), 681 (w), 657 (w) cm^{-1}; HRMS (CI(CH_4)) calcd for ($C_{17}H_{21}NO_2$ + H): 272.1645; found: 272.1646.

Compound 7b. This compound was obtained from **7a** (0.383 mmol, 104 mg) following the same procedure as for the preparation of **1b**. Colourless oil (80 mg, 91%).

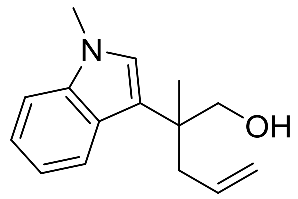

7b

^{1}H NMR (500 MHz, $CDCl_3$): δ = 7.78 (dt, *J* = 8.1, 0.9 Hz, 1H), 7.32 (dt, *J* = 8.2, 0.9 Hz, 1H), 7.23 (ddd, *J* = 8.2, 7.1, 1.1 Hz, 1H), 7.09 (ddd, *J* = 8.1, 7.0, 1.1 Hz, 1H), 6.89 (s, 1H), 5.67 (dddd, *J* = 17.0, 10.1, 8.1, 6.5 Hz, 1H), 5.05 (ddt, *J* = 17.1, 2.4, 1.4 Hz, 1H), 4.97 (ddt, *J* = 10.1, 2.1, 1.1 Hz, 1H), 3.93 (dd, *J* = 10.7, 5.6 Hz, 1H), 3.76 (s, 3H), 3.72 (dd, *J* = 10.7, 7.9 Hz, 1H), 2.76 (ddt, *J* = 13.7, 6.5, 1.3 Hz, 1H), 2.47 (ddt, *J* = 13.7, 8.0, 1.0 Hz, 1H), 1.40 (s, 3H), 1.23 (dd, *J* = 7.8, 5.6 Hz, 1H (OH)); ^{13}C NMR (125 MHz, $CDCl_3$): δ = 137.9 (e), 135.1 (o), 127.8 (o), 126.1 (e), 121.5 (o), 121.0 (o), 118.8 (o), 117.7 (e), 117.2 (e), 109.6 (o), 70.4 (e), 42.1 (e), 41.0 (e), 32.8 (o), 22.4 (o); IR (neat): $\tilde{\nu}$ = 3396 (br), 3064 (w), 2931 (m), 2877 (w), 1638 (w), 1615 (w), 1544 (w), 1484 (m), 1464 (m), 1424 (w), 1374 (m), 1330 (m), 1243 (m), 1137 (w), 1111 (w), 1034 (m), 996 (w), 913 (m), 810 (w), 765 (w), 738 (vs) cm^{-1}; HRMS (CI(CH_4)) calcd for ($C_{15}H_{19}NO$ + H): 230.1539; found: 230.1539.

Compound 7. This compound was obtained from **7b** (0.35 mmol, 79 mg) following the same procedure as for the preparation of **1**. Colourless oil (75 mg, 96%). ^{1}H NMR (500 MHz, $CDCl_3$): δ = 9.49 (s, 1H), 7.57 (dt, *J* = 8.1, 0.9 Hz, 1H), 7.32 (dt, *J* = 8.3, 0.9 Hz, 1H), 7.26-7.22 (m. 1H), 7.10 (ddd, *J* = 8.1, 7.0, 1.1 Hz, 1H), 6.96 (s, 1H), 5.61 (dddd, *J* = 17.1, 10.1, 7.7, 6.7 Hz, 1H), 5.07 (ddt, *J* = 17.0, 2.1, 1.4 Hz, 1H), 5.01 (ddt, *J* = 10.1, 2.1, 1.0 Hz, 1H), 3.80 (s, 3H), 2.91 (ddt, *J* = 14.1, 6.7, 1.3 Hz, 1H), 2.72 (ddt, *J* = 14.0, 7.8, 1.1 Hz, 1H), 1.49 (s, 3H); ^{13}C NMR (125 MHz, $CDCl_3$): δ = 201.6 (o), 137.6 (e), 133.7 (o), 127.4 (o), 126.1 (e), 121.8 (o), 120.1 (o), 119.4 (o), 118.0 (e), 112.9 (e), 109.5 (o), 49.8 (e), 38.9 (e), 32.8 (o), 18.8 (o); IR (neat): $\tilde{\nu}$ = 3064 (w), 2976 (w), 2932 (w), 2800 (w), 2702 (w), 1717 (s), 1640 (w), 1615 (w), 1541 (w), 1483 (m), 1465 (m), 1424 (w), 1367 (m), 1330 (m), 1243 (m), 1215 (w), 1153 (w), 1136 (w), 1111 (w), 1099 (w), 1071 (w), 1018 (w), 994 (m), 910 (s), 845 (w), 818 (w), 736 (vs), 667 (w) cm^{-1}; HRMS (CI(NH_4)) calcd for ($C_{15}H_{17}NO$ + H): 228.1383; found: 228.1380.

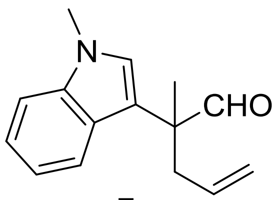

7

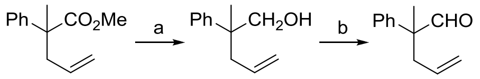

3a **9aa** **9a**

[(a)] $LiAlH_4$, Et_2O, 93%. [(b)] $(COCl)_2$, DMSO, Et_3N, CH_2Cl_2, 89%.

Compound 9aa. This compound was obtained from **3a** (4.90 mmol, 1.0 g) following the same procedure as for the preparation of **1b**. Colourless oil (800 mg, 93%). ^{1}H NMR (500 MHz, $CDCl_3$): δ = 7.39-7.33 (m, 4H), 7.25-7.21 (m, 1H), 5.60 (dddd, *J* = 17.0, 10.2, 7.9, 6.7 Hz, 1H), 5.05 (ddt, *J* = 17.0, 2.2, 1.4 Hz, 1H), 4.99 (ddt, *J* = 10.2, 2.2, 1.1 Hz, 1H), 3.76 (dd, *J* = 11.0, 5.8 Hz, 1H), 3.62 (ddt, *J* = 11.0, 7.5 Hz, 1H), 2.56 (ddt, *J* = 14.0, 6.6, 1.2 Hz, 1H), 2.37 (ddt, *J* = 13.9, 7.9, 1.1 Hz, 1H), 1.35 (s, 3H), 1.22 (dd, *J* = 7.5, 5.8 Hz, 1H); ^{13}C NMR (125 MHz, $CDCl_3$): δ = 144.6 (e), 134.5 (o), 128.4 (o, 2C), 126.7 (o, 2C), 126.2 (o), 117.5 (e), 71.7 (e), 43.1 (e), 42.9 (e), 21.8 (o); IR (neat): $\tilde{\nu}$ = 3362 (br), 3060 (w), 2973 (m), 2920 (m), 2875 (m), 1638 (m), 1600 (w), 1497 (m), 1445 (m), 1415 (w), 1375 (w), 1312 (w), 1156 (w), 1025 (s), 997 (m), 950 (w), 913 (s), 763 (s), 699 (vs), 670 (w) cm^{-1}; HRMS (CI(NH_4)) calcd for ($C_{12}H_{16}O$ + NH_4): 194.1539; found: 194.1548.

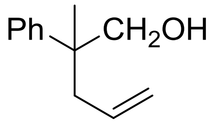

9aa

Compound 9a. This compound was obtained from **9aa** (284 mmol, 500 mg) following the same procedure as for the preparation of **1**. Colourless oil (440 mg, 89%). ^{1}H NMR (500 MHz, $CDCl_3$): δ = 9.53 (s, 1H), 7.41-7.36 (m, 2H), 7.31-7.27 (m, 1H), 7.26-7.24 (m, 2H), 5.55 (dddd, *J* = 17.0, 10.1, 7.7, 6.9 Hz, 1H), 5.06 (ddt, *J* = 16.8, 2.0, 1.4 Hz, 1H), 5.03 (ddt, *J* = 10.2, 2.1, 1.0 Hz, 1H), 2.70 (ddt, *J* = 14.1, 6.9, 1.2 Hz, 1H), 2.63 (ddt, *J* = 14.1, 7.7, 1.1 Hz, 1H), 1.45 (s, 3H), ^{13}C NMR (125 MHz, $CDCl_3$): δ = 201.9 (o), 139.4 (e), 133.1 (o), 128.8 (o, 2C), 127.3 (o), 127.1 (o, 2C), 118.6 (e), 53.6 (e), 40.6 (e), 18.8 (o); IR (neat): $\tilde{\nu}$ = 3077 (w), 3025 (w), 2978 (w), 2934 (w), 2806 (w), 2710 (w), 1722 (s), 1640 (w), 1599 (w), 1581 (w), 1494 (m), 1445 (m), 1417 (w), 1389 (w), 1372 (w), 1272 (w), 1158 (w), 1077 (w), 1029 (m), 996 (m), 917 (m), 876 (w), 857 (w), 833 (w), 760 (m), 698 (vs) cm^{-1}; HRMS (CI(NH_4)) calcd for ($C_{12}H_{14}O$ + NH_4): 192.1383; found: 192.1390.

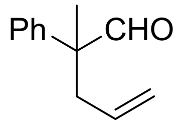

9a

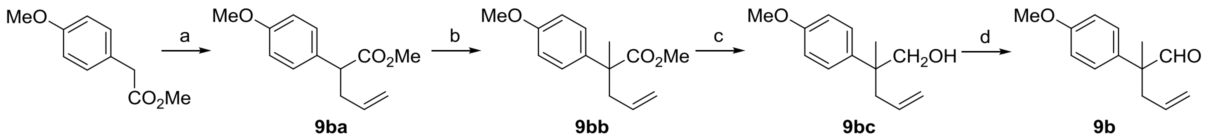

[(a)] i) LDA, THF, -78 °C; ii) allyl bromide, nBu_4NI, -78 °C to rt, 74%. [(b)] i) LDA, THF, -78 °C; ii) MeI, 66%. [(c)] $LiAlH_4$, Et_2O, 85%. [(d)] $(COCl)_2$, DMSO, Et_3N, CH_2Cl_2, 84%.

Compound 9bb. Known intermediate **9ba**[4] was first obtained as colourless oil (897 mg, 74%) from methyl (4-methoxyphenyl)acetate (5.55 mmol, 895 µL) following the same procedure as for the preparation of **1a**. Under N_2 atmosphere, diisopropylamine (2.50 mmol, 353 µL) was dissolved in THF (8 mL) and cooled to 0 °C. *n*Butyllithium (2.50 mmol, 1 mL, 2.5M in hexanes) was added and stirred for 15 minutes at 0 °C before cooling to –78 °C and adding Methyl 2-allyl-2-(*p*-methoxy)phenylacetate (2.27 mmol, 500 mg), stirring for 30 minutes at –78 °C. Methyl iodide (2.50 mmol, 156 µL) was added and the reaction mixture was warmed to room temperature and stirred for 16 hours. The reaction mixture was cooled to 0 °C, diluted with Et_2O and quenched carefully with saturated aqueous solution of ammonium chloride. After extraction, the organic phase was dried over $MgSO_4$ and the solvent removed under reduced pressure. Purification by flash column chromatography (petroleum ether/Et_2O = 99:1) afforded **9bb** as a colourless oil (350 mg, 66%). ^{1}H NMR (500 MHz, $CDCl_3$): δ = 7.26-7.21 (m, 2H), 6.88-6.84 (m, 2H), 5.60 (ddt, *J* = 17.1, 10.0, 7.2 Hz, 1H), 5.10-5.01 (m, 2H), 3.80 (s, 3H), 3.65 (s, 3H), 2.80 (dd, *J* = 13.7, 7.4 Hz, 1H), 2.64 (dd, *J* = 13.7, 7.0 Hz, 1H), 1.51 (s, 3H); ^{13}C NMR (125 MHz, $CDCl_3$): δ = 176.3 (e), 158.2 (e), 135.2 (e), 134.0 (o), 127.0 (o, 2C), 118.2 (e), 113.6 (o, 2C), 55.1 (o), 52.0 (o), 49.1 (e), 43.7 (e), 22.5 (o); IR (neat): $\tilde{\nu}$ = 3076 (w), 2979 (w), 2951 (w), 2838 (w), 1727 (vs), 1640 (w), 1610 (w), 1582 (w), 1513 (vs), 1461 (m), 1442 (m), 1417 (w), 1377 (w), 1278 (m), 1249 (vs), 1184 (s), 1143 (s), 1096 (m), 1032 (s), 1010 (w), 995 (m), 957 (w), 916 (m), 829 (s), 804 (m), 790 (w), 774 (w), 753 (w), 738 (w), 666 (w) cm^{-1}; HRMS (CI(NH_4)) calcd for ($C_{14}H_{18}O_3$ + H): 235.1328; found: 235.1336.

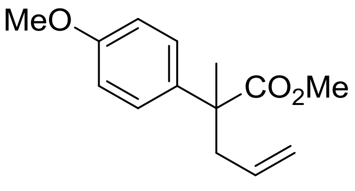

9bb

Compound 9bc. This compound was obtained from **9bb** (1.50 mmol, 350 mg) following the same procedure as for the preparation of **1b**. Colourless oil (262 mg, 85%). ^{1}H NMR (500 MHz, $CDCl_3$): δ = 7.31-7.26 (m, 2H), 6.91-6.87 (m, 2H), 5.60 (dddd, *J* = 17.0, 10.1, 7.8, 6.8 Hz, 1H), 5.07-5.01 (m, 1H), 5.01-4.97 (m, 1H), 3.80 (s, 3H), 3.71 (dd, *J* = 10.9, 5.7 Hz, 1H), 3.58 (dd, *J* = 10.9, 7.4 Hz, 1H), 2.53 (dd, *J* = 13.9, 6.6 Hz, 1H), 2.34 (dd, *J* = 13.9, 7.9 Hz, 1H), 1.32 (s, 3H), 1.21 (dd, *J* = 7.1, 6.1 Hz, 1H (OH)); ^{13}C NMR (125 MHz, $CDCl_3$): δ = 157.9 (e), 136.4 (e), 134.6 (o), 127.7 (o, 2C), 117.7 (e), 113.8 (o, 2C), 71.8 (e), 55.2 (o), 43.0 (e), 42.6 (e), 21.9 (o); IR (neat): $\tilde{\nu}$ = 3402 (br), 3074 (w), 2962 (w), 2933 (w), 2836 (w), 1638 (w), 1610 (m), 1580 (w), 1513 (vs), 1464 (m), 1441 (m), 1415 (w), 1373 (w), 1298 (m), 1247 (vs), 1185 (s), 1115 (w), 1028 (vs), 1010 (m), 951 (w), 912 (m), 827 (s), 798 (m), 735 (w), 680 (w) cm^{-1}; HRMS (CI(NH_4)) calcd for ($C_{13}H_{18}O_2$ + NH_4): 224.1645; found: 224.1652.

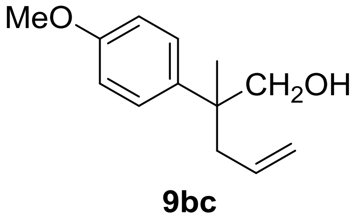

9bc

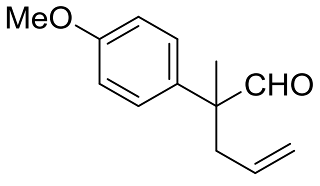

9b

Compound 9b. This compound was obtained from **9bc** (0.49 mmol, 100 mg) following the same procedure as for the preparation of **1**. Colourless oil (83 mg, 84%). ^{1}H NMR (500 MHz, $CDCl_3$): δ = 9.47 (s, 1H), 7.19-7.15 (m, 2H), 6.94-6.89 (m, 2H), 5.56 (dddd, *J* = 17.0, 10.2, 7.7, 6.9 Hz, 1H), 5.08-5.01 (m, 2H), 3.81 (s, 3H), 2.67 (ddt, *J* = 14.0, 6.9, 1.3 Hz, 1H), 2.60 (ddt, *J* = 14.1, 7.8. 1.1 Hz, 1H), 1.42 (s, 3H); ^{13}C NMR (125 MHz, $CDCl_3$): δ = 201.9 (o), 158.8

[4] P.-S. Lai, J. A. Dubland, M. G. Sawar, M. G. Chudzinski, M. S. Taylor, *Tetrahedron* **2011**, *67*, 7586

(e), 133.3 (o), 131.2 (e), 128.3 (o, 2C), 118.4 (e), 114.2 (o, 2C), 55.3 (o), 52.9 (e), 40.5 (e), 18.9 (o); IR (neat): $\tilde{\nu}$ = 3077 (w), 2977 (w), 2935 (w), 2837 (w), 2709 (w), 1720 (s), 1639 (w), 1609 (m), 1580 (w), 1512 (s), 1463 (m), 1442 (w), 1417 (w), 1389 (w), 1372 (w), 1299 (m), 1250 (vs), 1184 (s), 1116 (w), 1032 (s), 997 (w), 914 (m), 827 (s), 799 (m), 729 (w), 705 (w) cm^{-1}; HRMS (CI(NH_4)) calcd for ($C_{13}H_{16}O_2$ + NH_4): 222.1489; found: 222.1486; elemental analysis (%) calcd for $C_{13}H_{16}O_2$: C 76.44, H 7.90; found: C 76.85, H 8.00.

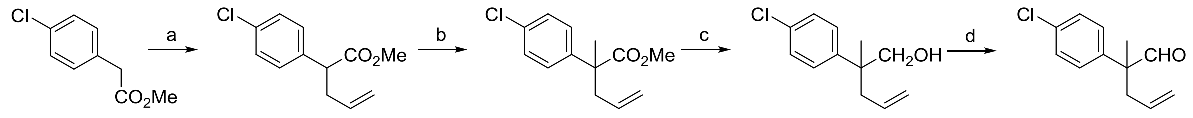

[a] i) LDA, THF, -78 °C; ii) allyl bromide, nBu_4NI, -78 °C to rt, 78%. [b] i) LDA, THF, -78 °C; ii) MeI, 58%. [c] $LiAlH_4$, Et_2O, 98%. [d] $(COCl)_2$, DMSO, Et_3N, CH_2Cl_2, 90%.

Compound 9cb. Known intermediate **9ca**[5] was first obtained as colourless oil (995 mg, 78%) from methyl 2-(4-chlorophenyl)acetate (5.42 mmol, 1.0 g) following the same procedure as for the preparation of **1a**. Then **9cb** was obtained from **9ca** (2.23 mmol, 500 mg) following the same procedure as for the preparation of **9bb**. Colourless oil (309 mg, 58%). ^{1}H NMR (500 MHz, $CDCl_3$): δ = 7.32-7.28 (m, 2H), 7.26-7.22 (m, 2H), 5.57 (ddt, *J* = 17.1, 10.0, 7.1 Hz, 1H), 5.09-5.02 (m, 2H), 3.66 (s, 3H), 2.79 (dd, *J* = 13.7, 7.4 Hz, 1H), 2.63 (dd, *J* = 13.7, 7.1 Hz, 1H), 1.52 (s, 3H); ^{13}C NMR (125 MHz, $CDCl_3$): δ = 175.8 (e), 141.7 (e), 133.5 (o), 132.7 (e), 128.5 (o, 2C), 127.5 (o, 2C), 118.7 (e), 52.2 (o), 49.6 (e), 43.6 (e), 22.5 (o); IR (neat): $\tilde{\nu}$ = 3078 (w), 2981 (w), 2951 (w), 1732 (vs), 1641 (w), 1494 (m), 1460 (w), 1434 (w), 1402 (w), 1378 (w), 1291 (w), 1235 (m), 1145 (m), 1096 (m), 1013 (m), 995 (w), 919 (m), 827 (m), 760 (w), 735 (w) cm^{-1}; HRMS (CI(CH_4)) calcd for ($C_{13}H_{15}{}^{35}ClO_2$ + H): 239.0833; found: 239.0842.

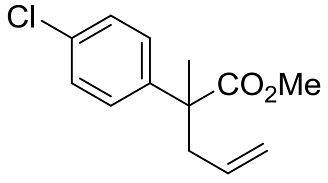

9cb

Compound 9cc. This compound was obtained from **9cb** (1.26 mmol, 300 mg) following the same procedure as for the preparation of **1b**. Colourless oil (261 mg, 98%). ^{1}H NMR (500 MHz, $CDCl_3$): δ = 7.34-7.28 (m, 4H), 5.57 (dddd, *J* = 17.0, 10.1, 7.7, 6.8 Hz, 1H), 5.07-4.98 (m, 2H), 3.73 (dd, *J* = 11.0, 5.8 Hz, 1H), 3.60 (dd, *J* = 11.0, 7.2 Hz, 1H), 2.52 (dd, *J* = 13.9, 6.7 Hz, 1H), 2.35 (dd, *J* = 13.9, 7.8 Hz, 1H), 1.32 (s, 3H), 1.24 (t, *J* = 6.5 Hz, 1H (OH)); ^{13}C NMR (125 MHz, $CDCl_3$): δ = 143.3 (e), 134.0 (o), 132.1 (e), 128.5 (o, 2C), 128.2 (o, 2C), 117.9 (e), 71.5 (e), 43.0 (e), 42.9 (e), 21.9 (o); IR (neat): $\tilde{\nu}$ = 3375 (br), 3076 (w), 2976 (w), 2923 (w), 2879 (w), 1639 (w), 1595 (w), 1494 (m), 1440 (w), 1400 (w), 1314 (w), 1154 (w), 1097 (m), 1031 (s), 1011 (vs), 997 (m), 951 (w), 914 (s), 823 (s), 749 (m), 722 (m), 672 (w) cm^{-1}; HRMS (CI(NH_4)) calcd for ($C_{12}H_{15}{}^{35}ClO$ + NH_4): 228.1150; found: 228.1148.

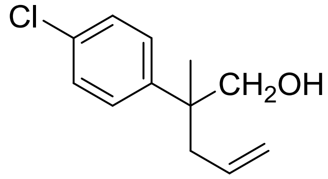

9cc

Compound 9c. This compound was obtained from **9cc** (0.48 mmol, 100 mg) following the same procedure as for the preparation of **1**. Colourless oil (89 mg, 90%). ^{1}H NMR (500 MHz, $CDCl_3$): δ = 9.50 (s, 1H), 7.38-7.33 (m, 2H), 7.21-7.16 (m, 2H), 5.52 (ddt, *J* = 17.0, 10.1, 7.2 Hz, 1H), 5.09-5.02 (m, 2H), 2.66 (dd, *J* = 14.2, 6.9 Hz, 1H), 2.60 (dd, *J* = 14.2, 7.7 Hz, 1H), 1.44 (s, 3H); ^{13}C NMR (125 MHz, $CDCl_3$): δ = 201.4 (o), 138.0 (e), 133.4 (e), 132.7 (o), 129.0 (o, 2C), 128.6 (o, 2C), 119.0 (e), 53.3 (e), 40.6 (e), 18.9 (o); IR (neat): $\tilde{\nu}$ = 3078 (w), 2980 (w), 2935 (w), 2809 (w), 2711 (w), 1724 (vs), 1641 (w), 1595 (w), 1494 (m), 1460 (w), 1402 (w), 1372 (w), 1274 (w), 1097 (m), 1055 (w), 1033 (w), 1013 (m), 996 (m), 917 (m), 877 (w), 822 (m), 755 (m), 720 (m), 688 (w) cm^{-1}; elemental analysis (%) calcd for $C_{12}H_{13}ClO$: C 69.07, H 6.28; found: C 69.92, H 6.40.

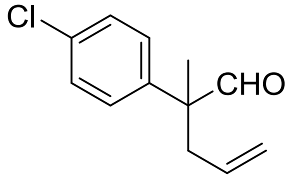

9c

[5] M. Pour, M. Špulák, V. Balšánek, J. Kuneš, P. Kubanová, V. Buchta, *Bioorg. Med. Chem.* **2003**, *11*, 2843

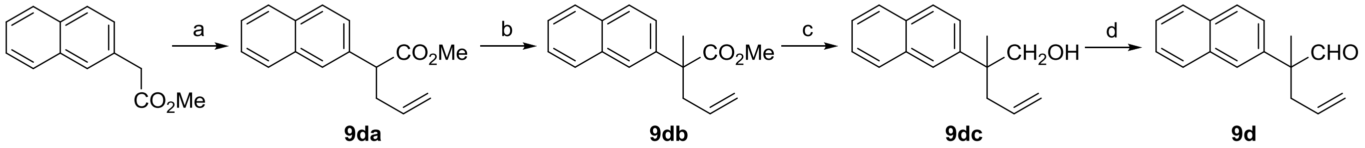

[(a)] i) LDA, THF, -78 °C; ii) allyl bromide, nBu_4NI, -78 °C to rt, 55%. [(b)] i) LDA, THF, -78 °C; ii) MeI, 55%. [(c)] $LiAlH_4$, Et_2O, quantitative. [(d)] $(COCl)_2$, DMSO, Et_3N, CH_2Cl_2, 84%.

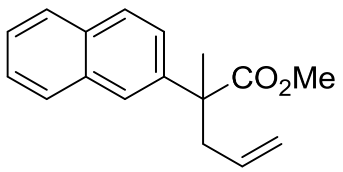

9db

Compound 9db. Intermediate **9da** was first obtained as colourless oil (659 mg, 55%) from methyl 2-(naphthalen-2-yl)acetate (4.99 mmol, 1.0 g) following the same procedure as for the preparation of **1a**. Then **9db** was obtained from **9da** (1.25 mmol, 300 mg) following the same procedure as for the preparation of **9bb**. Colourless oil (174 mg, 55%). ^{1}H NMR (500 MHz, $CDCl_3$): δ = 7.85-7.78 (m, 3H), 7.76-7.73 (m, 1H), 7.51-7.41 (m, 3H), 5.62 (ddt, *J* = 17.1, 9.8, 7.2 Hz, 1H), 5.13-5.07 (m, 1H), 5.07-5.02 (m, 1H), 3.67 (s, 3H), 2.92 (dd, *J* = 13.7, 7.5 Hz, 1H), 2.80 (dd, *J* = 13.7, 7.0 Hz, 1H), 1.64 (s, 3H); ^{13}C NMR (125 MHz, $CDCl_3$): δ = 176.3 (e), 140.6 (e), 133.9 (o), 133.2 (e), 132.2 (e), 128.1 (o, 2C), 127.4 (o), 126.1 (o), 125.9 (o), 124.6 (o), 124.4 (o), 118.5 (e), 52.2 (o), 50.1 (e), 43.5 (e), 22.6 (o); IR (neat): $\tilde{\nu}$ = 3059 (w), 2979 (w), 2950 (w), 1730 (vs), 1639 (w), 1600 (w), 1507 (w), 1458 (w), 1434 (w), 1379 (w), 1282 (w), 1233 (m), 1143 (m), 1108 (w), 995 (w), 918 (m), 856 (w), 818 (m), 778 (w), 750 (m); cm^{-1}; HRMS (CI(CH_4)) calcd for ($C_{17}H_{18}O_2$ + H): 255.1380; found: 255.1388.

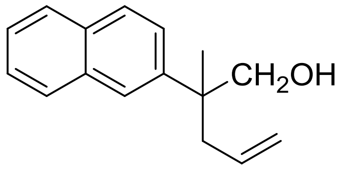

9dc

Compound 9dc. This compound was obtained from **9db** (0.68 mmol, 174 mg) following the same procedure as for the preparation of **1b**. Colourless oil (155 mg, quantitative). ^{1}H NMR (500 MHz, $CDCl_3$): δ = 7.86-7.80 (m, 3H), 7.79-7.76 (m, 1H), 7.56-7.52 (m, 1H), 7.50-7.43 (m, 2H), 5.61 (dddd, *J* = 16.9, 10.2, 7.8, 6.7 Hz, 1H), 5.10-5.04 (m, 1H), 5.01-4.96 (m, 1H), 3.86 (dd, *J* = 11.0, 5.7 Hz, 1H), 3.71 (dd, *J* = 11.0, 7.5 Hz, 1H), 2.68 (dd, *J* = 13.9, 6.6 Hz, 1H), 2.45 (dd, *J* = 13.9, 7.9 Hz, 1H), 1.46 (s, 3H), 1.24 (dd, *J* = 13.0, 7.3 Hz, 1H (OH)); ^{13}C NMR (125 MHz, $CDCl_3$): δ = 142.0 (e), 134.4 (o), 133.3 (e), 132.0 (e), 128.1 (o), 127.9 (o), 127.4 (o), 126.0 (o), 125.7 (o, 2C), 124.8 (o), 117.6 (e), 71.6 (e), 43.4 (e), 42.9 (e), 21.9 (o); IR (neat): $\tilde{\nu}$ = 3372 (br), 3058 (w), 2973 (w), 2923 (w), 2875 (w), 1638 (w), 1599 (w), 1506 (w), 1468 (w), 1436 (w), 1415 (w), 1377 (w), 1314 (w), 1274 (w), 1243 (w), 1203 (w), 1133 (w), 1031 (m), 997 (m), 946 (w), 912 (m), 890 (w), 854 (m), 815 (s), 770 (w), 745 (vs), 695 (w), 662 (w) cm^{-1}; HRMS (CI(CH_4)) calcd for ($C_{16}H_{18}O$ + H): 227.1431; found: 227.1422; elemental analysis (%) calcd for $C_{16}H_{18}O$: C 84.91, H 8.02; found: C 84.98, H 8.17.

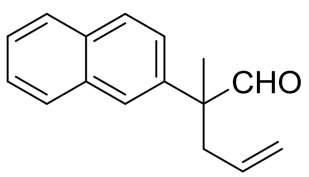

9d

Compound 9d. This compound was obtained from **9dc** (0.44 mmol, 100mg) following the same procedure as for the preparation of **1**. Colourless oil (83 mg, 84%). ^{1}H NMR (500 MHz, $CDCl_3$): δ = 9.60 (s, 1H), 7.89-7.80 (m, 3H), 7.73-7.70 (m, 1H), 7.53-7.46 (m, 2H), 7.39-7.35 (m, 1H), 5.57 (ddt, *J* = 17.1, 10.0, 7.3 Hz, 1H), 5.08 (d, *J* = 17.1 Hz, 1H), 5.03 (d, *J* = 10.1 Hz, 1H), 2.82 (dd, *J* = 14.2, 6.8 Hz, 1H), 2.72 (dd, *J* = 14.2, 7.8 Hz, 1H), 1.56 (s, 3H); ^{13}C NMR (125 MHz, $CDCl_3$): δ = 201.9 (o), 136.8 (e), 133.4 (e), 133.1 (o), 132.4 (e), 128.6 (o), 128.0 (o), 127.5 (o), 126.5 (o), 126.3 (o), 126.2 (o), 125.0 (o), 118.7 (e), 53.8 (e), 40.5 (e), 18.9 (o); IR (neat): $\tilde{\nu}$ = 3058 (w), 2932 (w), 2977 (w), 2805 (w), 2706 (w), 1720 (vs), 1639 (w), 1598 (w), 1505 (w), 1456 (w), 1437 (w), 1416 (w), 1389 (w), 1374 (w), 1275 (w), 1245 (w), 1192 (w), 1132 (w), 1056 (w), 1018 (w), 995 (m), 949 (w), 915 (m), 891 (w), 879 (w), 856 (m), 816 (s), 769 (w), 747 (s), 667 (m) cm^{-1}; HRMS (CI(NH_4)) calcd for ($C_{16}H_{16}O$ + NH_4): 242.1539; found: 242.1533.

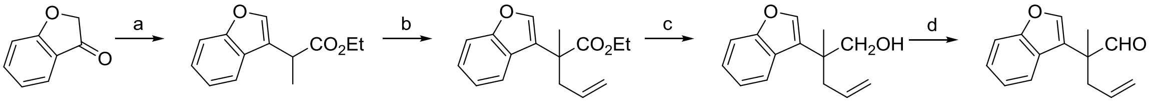

[(a)] ethyl-2-(triphenylphosphoranylidene)propionate, toluene, reflux, 37%. [(b)] i) LDA, THF, -78 °C; ii) allyl bromide, nBu_4NI, -78 °C to rt, 57%. [(c)] $LiAlH_4$, Et_2O, 70%. [(d)] $(COCl)_2$, DMSO, Et_3N, CH_2Cl_2, 95%.

Compound 9eb. Intermediate **9ea** was first obtained as colourless oil (486 mg, 37%) from 3-coumaranone (6.54 mmol, 878 mg) and ethyl-2-(triphenylphosphoranylidene)propionate (6.54 mmol, 2.37 g) following a known procedure.[6] Then **9eb** was obtained from **9ea** (2.22 mmol, 485 mg) following the same procedure as for the preparation of **9bb**. Purification by flash column chromatography (petroleum ether/Et_2O = 24:1) afforded **9ga** as a colourless oil (328 mg, 57%). ^{1}H NMR (500 MHz, $CDCl_3$): δ = 7.63 (d, *J* = 7.8 Hz, 1H), 7.48 (s, 1H), 7.47 (d, *J* = 8.2 Hz, 1H), 7.30-7.26 (m, 1H), 7.21 (t, *J* = 7.5 Hz, 1H), 5.65 (ddt, *J* = 17.1, 10.0, 7.2 Hz, 1H), 5.12-5.04 (m, 2H), 4.18–4.08 (m, 2H), 2.90 (dd, *J* = 13.7, 7.5 Hz, 1H), 2.81 (dd, *J* = 13.8, 7.1 Hz, 1H), 1.61 (s, 3H), 1.16 (t, *J* = 7.1 Hz, 3H); ^{13}C NMR (125 MHz, $CDCl_3$): δ = 174.8 (e), 155.7 (e), 141.4 (o), 133.4 (o), 126.2 (e), 124.2 (o), 123.5 (e), 122.4 (o), 121.1 (o), 118.7 (e), 111.6 (o), 61.1 (e), 44.9 (e), 41.8 (e), 22.1 (o), 14.1 (o); IR (neat): $\tilde{\nu}$ = 3077 (w), 2981 (w), 2938 (w), 1726 (s), 1640 (w), 1454 (s), 1377 (m), 1281 (w), 1233 (m), 1205 (m), 1144 (m), 1110 (m), 1091 (s), 1063 (w), 1014 (m), 995 (w), 957 (w), 917 (m), 858 (m), 808 (w), 758 (w), 744 (vs) cm^{-1}; HRMS (CI(NH_4)) calcd for ($C_{16}H_{18}O_3$ + H): 259.1329; found: 259.1332.

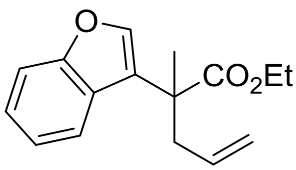

9eb

Compound 9ec. This compound was obtained from **9eb** (1.27 mmol, 328 mg) following the same procedure as for the preparation of **1b**. Colourless oil (191 mg, 70%). ^{1}H NMR (500 MHz, $CDCl_3$): δ = 7.74 (ddd, *J* = 7.7, 1.1, 0.9 Hz, 1H), 7.49 (ddd, *J* = 8.2, 1.1, 0.7 Hz, 1H), 7.44 (s, 1H), 7.27 (dddd, *J* = 8.2, 7.2, 1.3, 0.3 Hz, 1H), 7.23 (ddd, *J* = 7.8, 7.2, 1.1 Hz, 1H), 5.66 (dddd, *J* = 17.1, 10.0, 7.7, 6.7 Hz, 1H), 5.07 (ddt, *J* = 17.0, 2.2, 1.4 Hz, 1H), 5.01 (ddt, *J* = 10.1, 2.1, 1.0 Hz, 1H), 3.90 (dd, *J* = 10.9, 5.9 Hz, 1H), 3.75 (11.0, 7.4 Hz, 1H), 2.71 (ddt, *J* = 13.8, 6.8, 1.2 Hz, 1H), 2.49 (13.8, 7.9, 1.1 Hz, 1H), 1.40 (s, 3H), 1.31 (dd, *J* = 7.4, 5.9 Hz, 1H (OH)); ^{13}C NMR (125 MHz, $CDCl_3$): δ = 156.0 (e), 142.5 (o), 134.2 (o), 126.4 (e), 124.2 (o, 2C), 122.4 (o), 121.5 (o), 117.9 (e), 111.9 (o), 69.6 (e), 41.1 (e), 40.2 (e), 21.5 (o); IR (neat): $\tilde{\nu}$ = 3370 (br), 3074 (w), 2976 (w), 2933 (w), 1639 (w), 1453 (m), 1374 (w), 1258 (w), 1202 (w), 1112 (m), 1097 (m), 1041 (m), 1015 (m), 915 (m), 858 (m), 805 (w), 768 (m), 744 (vs), 698 (w) cm^{-1}; HRMS (CI(CH_4)) calcd for (($C_{14}H_{16}O_2$ − H_2O) + H): 199.1117; found: 199.1114.

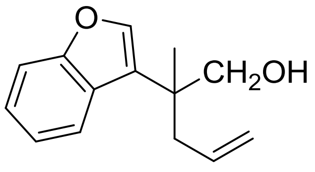

9ec

Compound 9e. This compound was obtained from **9dc** (0.88 mmol, 190 mg) following the same procedure as for the preparation of **1**. Colourless oil (178 mg, 95%). ^{1}H NMR (500 MHz, $CDCl_3$): δ = 9.58 (s, 1H), 7.55–7.51 (m, 1H), 7.53 (s, 1H), 7.52–7.49 (m, 1H), 7.31 (ddd, *J* = 8.3, 7.2, 1.1 Hz, 1H), 7.23 (ddd, *J* = 7.9, 7.2, 1.0 Hz, 1H), 5.61 (dddd, *J* = 17.0, 10.1, 7.7, 6.9 Hz, 1H), 5.12-5.04 (m, 2H), 2.86 (ddt, *J* = 14.1, 6.9, 1.2 Hz, 1H), 2.72 (ddt, *J* = 14.1, 7.7, 1.0 Hz, 1H), 1.52 (s, 3H); ^{13}C NMR (125 MHz, $CDCl_3$): δ = 201.1 (o), 155.9 (e), 142.6 (o), 132.6 (o), 125.8 (e), 124.6 (o), 122.8 (o), 120.8 (o), 119.9 (e), 118.9 (e), 111.8 (o), 49.3 (e), 38.5 (e), 18.5 (o); IR (neat): $\tilde{\nu}$ = 3370 (br), 3074 (w), 2976 (w), 2933 (w), 1639 (w), 1453 (m), 1374 (w), 1258 (w), 1202 (w), 1112 (m), 1097 (m), 1041 (m), 1015 (m), 915 (m), 858 (m), 805 (w), 768 (m), 744 (vs), 698 (w) cm^{-1}; 3076 (w), 2979 (w), 2936 (w), 2807 (w), 2709 (w), 1724 (s), 1640 (w), 1615 (w), 1568 (w),

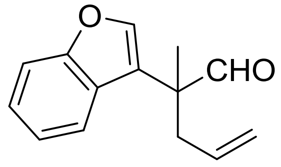

9e

[6] A. M. Venkatesan, O. D. Santos, M. Asselin, G. T. Grosu, D. A. Evrard, R. E. Mershaw, K. Meagher, US 20090054454 A1

1454 (m), 1418 (w), 1388 (w), 1372 (w), 1328 (w), 1292 (w), 1252 (w), 1203 (w), 1164 (w), 1112 (m), 1097 (m), 1016 (m), 995 (m), 918 (m), 857 (m), 879 (w), 816 (w), 768 (m), 743 (vs), 656 (w) cm^{-1}; HRMS (CI(CH_4)) calcd for ($C_{14}H_{14}O_2$ + H): 215.1067; found: 215.1067.

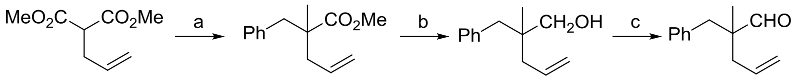

9fa **9fb** **9f**

[(a)] i) NaH, benzyl bromide, THF; ii) LiCl, DMSO, 155 °C; iii) LDA, THF, -78 °C; iv) MeI, 65% over three steps. [(b)] $LiAlH_4$, Et_2O, 82%. [(c)] $(COCl)_2$, DMSO, Et_3N, CH_2Cl_2, 72%.

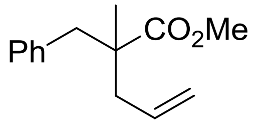

9fa

Compound 9fa. Under N_2 atmosphere, NaH (9.33 mmol, 373 mg, 60% dispersion in oil) was suspended in THF (16 mL) and the solution was cooled to 0 °C. Dimethyl allyl malonate (6.22 mmol, 1 mL) was added *via* syringe and the mixture was stirred to room temperature for 30 minutes. Benzyl bromide (7.46 mmol, 892 µL) was then added at 0 °C and the reaction was stirred to room temperature for 90 minutes. The reaction was quenched with a saturated aqueous solution of ammonium chloride and extracted with Et_2O. The combined organic layers were dried over $MgSO_4$ and the solvent was removed under reduced pressure, affording the crude dimethyl 2-allyl-2-benzylmalonate which was then dissolved in DMSO (28 mL) under N_2 atmosphere. Lithium chloride (10.07 mmol, 427 mg) and distilled water (0.2 mL) were added. After heating to 155 °C for 16 hours, the reaction was quenched with brine, extracted with Et_2O (3 × 30 mL) and the combined organic layers were washed with water (3 × 50 mL) and then dried over $MgSO_4$, filtered and concentrated. The resulting crude oil was purified by flash column chromatography (petroleum ether/Et_2O = 99:1), affording methyl 2-benzylpent-4-enoate (608 mg, 65%) as a colourless oil. The methylation procedure described for the preparation of **9bb** was applied to this material. Purification by flash column chromatography (petroleum ether/Et_2O = 50:1) afforded **9fa** as a colourless oil (643 mg, quantitative). ^{1}H NMR (500 MHz, $CDCl_3$): δ = 7.28-7.19 (m, 3H), 7.11-7.08 (m, 2H), 5.76 (dddd, J = 15.2, 10.9, 8.0, 7.4 Hz, 1H), 5.10-5.05 (m, 2H), 3.65 (s, 3H), 3.01 (d, J = 13.4 Hz, 1H), 2.73 (d, J = 13.3 Hz, 1H), 2.51 (dd, J = 13.7, 7.0 Hz, 1H), 2.18 (ddt, J = 13.7, 7.7, 1.1 Hz, 1H), 1.11 (s, 3H); ^{13}C NMR (125 MHz, $CDCl_3$): δ = 176.6 (e), 137.5 (e), 134.0 (o), 130.1 (o, 2C), 128.0 (o, 2C), 126.5 (o), 118.2 (e), 51.5 (o), 47.5 (e), 45.1 (e), 43.5 (e), 20.9 (o); IR (neat): $\tilde{\nu}$ = 3065 (w), 3030 (w), 2980 (w), 2949 (w), 1728 (vs), 1640 (w), 1605 (w), 1496 (m), 1455 (m), 1434 (m), 1381 (w), 1333 (w), 1212 (m), 1195 (m), 1143 (m), 1120 (w), 1082 (w), 1058 (w), 1032 (w), 994 (m), 917 (m), 861 (w), 808 (w), 770 (w), 742 (m), 701 (vs) cm^{-1}; HRMS (CI(CH_4)) calcd for ($C_{14}H_{18}O_2$ + H): 219.1379; found: 219.1382; elemental analysis (%) calcd for $C_{14}H_{18}O_2$: C 77.03, H 8.31; found: C 77.24, H 8.41.

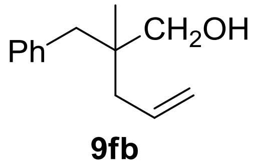

9fb

Compound 9fb. This compound was obtained from **9fa** (2.29 mmol, 500 mg) following the same procedure as for the preparation of **1b**. Colourless oil (355 mg, 82%). ^{1}H NMR (500 MHz, $CDCl_3$): δ = 7.30-7.25 (m, 2H), 7.24-7.17 (m, 3H), 5.92 (ddt, J = 17.3, 9.8, 7.7 Hz, 1H), 5.14-5.08 (m, 2H), 3.34 (d, J = 5.9 Hz, 2H), 2.67 (d, J = 13.2 Hz, 1H), 2.57 (d, J = 13.2 Hz, 1H), 2.13 (ddt, J = 13.8, 7.6, 1.1 Hz, 1H), 2.04 (ddt, J = 13.8, 7.4, 1.2 Hz, 1H), 1.34 (t, J = 5.9 Hz, 1H (OH)); ^{13}C NMR (125 MHz, $CDCl_3$): δ = 138.3 (e), 135.0 (o), 130.5 (o, 2C), 127.8 (o, 2C), 125.9 (o), 68.4 (e), 42.9 (e), 41.6 (e), 39.1 (e), 21.1 (o); IR (neat): $\tilde{\nu}$ = 3376 (br), 3074 (w), 3028 (w), 3003 (w), 2960 (w), 2923 (w), 2874 (w), 1638 (w), 1603 (w), 1495 (m), 1453 (m), 1414 (w), 1376 (w), 1317 (w), 1229 (w), 1155 (w), 1057 (w), 1029 (s), 998 (m), 912 (s), 877 (w), 813 (w), 780 (m), 729 (m), 700 (vs) cm^{-1}; HRMS (CI(NH_4)) calcd for ($C_{13}H_{18}O$ + NH_4): 208.1696; found: 208.1697.

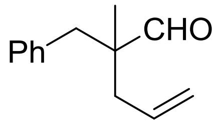

9f

Compound 9f. This compound was obtained from **9fb** (0.53 mmol, 100 mg) following the same procedure as for the preparation of **1**. Colourless oil (71 mg, 72%). ^{1}H NMR (500 MHz, $CDCl_3$): δ = 9.60 (s, 1H), 7.29-7.25 (m, 2H), 7.24-7.19 (m, 1H), 7.11-7.08 (m, 2H), 5.74 (ddt, J = 17.2, 9.9, 7.2 Hz, 1H), 5.14-5.07 (m, 2H), 2.89 (d, J = 13.8 Hz,

1H), 2.75 (d, *J* = 13.8 Hz, 1H), 2.36 (dd, *J* = 14.1, 7.2 Hz, 1H), 2.19 (ddt, *J* = 14.1, 7.6 Hz, 1.1 Hz, 1H), 1.03 (s, 3H); ^{13}C NMR (125 MHz, $CDCl_3$): δ = 205.9 (o), 136.6 (e), 133.0 (o), 130.2 (o, 2C), 128.2 (o, 2C), 126.6 (o), 118.8 (e), 50.0 (e), 41.7 (e), 39.9 (e), 18.5 (o); IR (neat): $\tilde{\nu}$ = 3065 (w), 3030 (w), 2977 (w), 2929 (w), 2713 (w), 1723 (s), 1640 (w), 1604 (w), 1497 (m), 1454 (m), 1417 (w), 1395 (w), 1373 (w), 1326 (w), 1181 (w), 1076 (w), 1031 (w), 995 (m), 917 (m), 861 (w), 809 (w), 782 (m), 740 (m), 701 (vs), 664 (w) cm^{-1}; HRMS (CI(NH_4)) calcd for ($C_{13}H_{16}O$ + NH_4): 206.1539; found: 206.1539; elemental analysis (%) calcd for $C_{13}H_{16}O$: C 82.94, H 8.57; found: C 83.00, H 8.75.

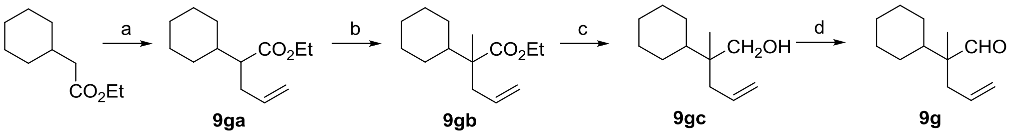

9ga 9gb 9gc 9g

[(a)] i) LDA, THF, -78 °C; ii) allyl bromide, nBu_4NI, -78 °C to rt, 62%. [(b)] i) LDA, THF, -78 °C; ii) MeI, 62%. [(c)] $LiAlH_4$, Et_2O, 77%. [(d)] $(COCl)_2$, DMSO, Et_3N, CH_2Cl_2, 72%.

Compound 9gb. This compound was obtained from ethyl 2-cyclohexylpent-4-enoate (2.38 mmol, 500 mg) following the same procedure as for the preparation of **9bb**. Colourless oil (331 mg, 62%). ^1H NMR (500 MHz, $CDCl_3$): δ = 5.74-5.65 (m, 1H), 5.05-5.00 (m, 2H), 4.13 (d, *J* = 7.2 Hz, 1H), 4.11 (q, *J* = 7.1 Hz, 2H), 2.40 (dd, *J* = 13.5, 6.9 Hz, 1H), 2.16 (dd, *J* = 13.5, 7.8 Hz, 1H), 1.82-1.57 (m, 5H), 1.49–1.42 (m, 1H), 1.25 (*t*, *J* = 7.1 Hz, 3H), 1.27–1.17 (m, 2H), 1.16–1.04 (m, 2H), 1.02 (s, 3H), 1.02–0.92 (m, 1H); ^{13}C NMR (125 MHz, $CDCl_3$): δ = 176.7 (e), 134.7 (o), 117.5 (e), 60.0 (e), 49.8 (e), 45.3 (o), 41.8 (e), 28.5 (e), 27.0 (e), 26.8 (e, 2C), 26.6 (e), 16.8 (o), 14.3 (o); IR (neat): $\tilde{\nu}$ = 3078 (w), 2980 (m), 2928 (s), 2854 (m), 1725 (vs), 1640 (w), 1449 (m), 1382 (m), 1366 (w), 1281 (m), 1214 (s), 1189 (s), 1139 (s), 1097 (m), 1061 (m), 1026 (m), 994 (w), 913 (m), 863 (w), 850 (w), 778 (w), 756 (w) cm^{-1}; HRMS (CI(CH_4)) calcd for ($C_{14}H_{24}O_2$ + H): 225.1850; found: 225.1852; elemental analysis (%) calcd for $C_{14}H_{24}O_2$: C 74.95, H 10.89; found: C 74.99, H 10.72.

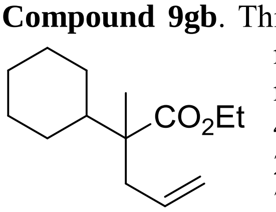

9gb

Compound 9gc. This compound was obtained from **9gb** (1.47 mmol, 330 mg) following the same procedure as for the preparation of **1b**. Colourless oil (208 mg, 77%). ^1H NMR (500 MHz, $CDCl_3$): δ = 5.89 (ddt, *J* = 17.2, 9.9, 7.4 Hz, 1H), 5.11-5.03 (m, 2H), 3.47 (dd, *J* = 11.2, 6.5 Hz, 1H), 3.43 (dd, *J* = 11.1, 6.0 Hz, 1H), 2.16-2.05 (m, 2H), 1.82-1.62 (m, 5H), 1.34 (tt, *J* = 17.9, 2.9 Hz, 1H), 1.30–1.25 (m, 1H), 1.25–1.17 (m, 2H), 1.13 (tt, *J* = 12.6, 3.4 Hz, 1H), 1.08–0.96 (m, 2H), 0.78 (s, 3H); ^{13}C NMR (125 MHz, $CDCl_3$): δ = 136.0 (o), 116.9 (e), 68.2 (e), 42.1 (o), 40.2 (e), 40.1 (e), 27.2 (e, 2C), 27.1 (e), 27.0 (e), 26.7 (e), 18.7 (o); IR (neat): $\tilde{\nu}$ = 3363 (br), 3074 (w), 2922 (vs), 2852 (s), 1638 (m), 1448 (m), 1415 (w), 1377 (w), 1270 (w), 1155 (w), 1028 (s), 997 (m), 909 (vs), 892 (m), 845 (m), 807 (w), 761 (w), 663 (w) cm^{-1}; HRMS (CI(CH_4)) calcd for ($C_{12}H_{22}O$ + H): 183.1744; found: 183.1742; elemental analysis (%) calcd for $C_{12}H_{22}O$: C 79.06, H 12.16; found: C 79.25, H 12.28.

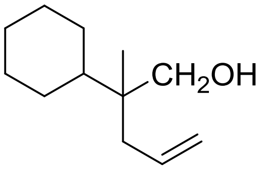

9gc

Compound 9g. This compound was obtained from **9fb** (0.55 mmol, 100 mg) following the same procedure as for the preparation of **1**. Colourless oil (71 mg, 72%). ^1H NMR (500 MHz, $CDCl_3$): δ = 9.49 (s, 1H), 5.72-5.61 (m, 1H), 5.08-5.01 (m, 2H), 2.34 (dd, *J* = 14.1, 7.0 Hz, 1H), 2.21 (dd, *J* = 14.1, 7.9 Hz, 1H), 1.84-1.64 (m, 4H), 1.62-1.48 (m, 2H), 1.30-1.18 (m, 2H), 1.17-0.98 (m, 3H), 0.96 (s, 3H); ^{13}C NMR (125 MHz, $CDCl_3$): δ = 207.1 (o), 133.5 (o), 118.1 (e), 51.7 (e), 42.2 (o), 38.4 (e), 28.0 (e), 26.8 (e), 26.7 (e, 2C), 26.4 (e), 14.8 (o); IR (neat): $\tilde{\nu}$ = 3078 (w), 2978 (w), 2926 (s), 2854 (m), 2701 (w), 1722 (vs), 1640 (m), 1450 (m), 1417 (w), 1396 (w), 1375 (w), 1351 (w), 1272 (w), 1188 (w), 993 (m), 913 (s), 893 (w), 868 (w), 845 (m), 798 (w), 769 (w) cm^{-1}; HRMS (CI(CH_4)) calcd for ($C_{12}H_{20}O$ + H): 181.1587; found: 181.1589.

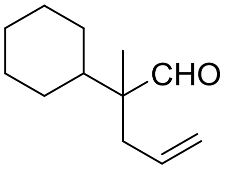

9g

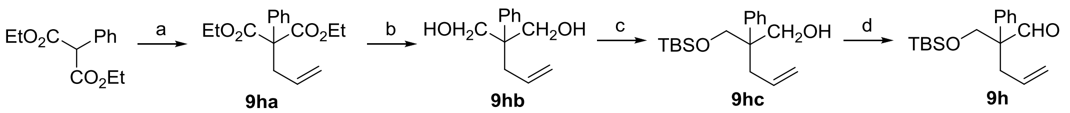

[(a)] NaH, allyl bromide, DMF, 83%. [(b)] $LiAlH_4$, Et_2O, 74%. [(c)] TBSCl, imidazole, DMF, 69%. [(d)] $(COCl)_2$, DMSO, Et_3N, CH_2Cl_2, 96%.

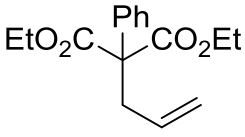

9ha

Compound 9ha. Under a N_2 atmosphere, sodium hydride (31.74 mmol, 1.27 g, 60% dispersion in oil) was suspended in DMF (106 mL) and cooled to 0 °C. Diethyl phenylmalonate (21.16 mmol, 4.57 mL) was added slowly *via* syringe and stirred at 0 °C for 10 minutes before adding allyl bromide (31.74 mmol, 2.85 mL), stirring for 1 hour. The reaction mixture was then quenched with a saturated aqueous solution of ammonium chloride and extracted with Et_2O. The organic layer washed several times with water and then dried over $MgSO_4$, filtered and concentrated. The crude oil was purified by flash column chromatography (petroleum ether/Et_2O = 24:1), affording **9ha** as a colourless oil (4.86 g, 83%); ^{1}H NMR (500 MHz, $CDCl_3$): δ = 7.44-7.39 (m, 2H), 7.36-7.25 (m, 3H), 5.76 (ddt, *J* = 17.1, 10.1, 7.1 Hz, 1H), 5.10-5.02 (m, 2H), 4.28–4.15 (m, 4H), 3.07 (dt, *J* = 7.2, 1.2 Hz, 2H), 1.24 (t, *J* = 7.1 Hz, 6H), in agreement with previously reported data.[7]

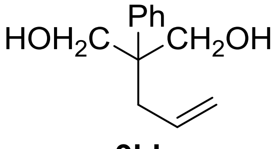

9hb

Compound 9hb. This compound was obtained from **9ha** (17.62 mmol, 4.86 g) following the same procedure as for the preparation of **1b**, except that 2.2 equiv. of $LiAlH_4$ (38.76 mmol, 1.47 g) in Et_2O (100 mL) was used. White solid (2.51 g, 74%). ^{1}H NMR (500 MHz, $CDCl_3$): δ = 7.41-7.36 (m, 4H), 7.29-7.24 (m, 1H), 5.54 (ddt, *J* = 17.2, 10.0, 7.2 Hz, 1H), 5.10-5.03 (m, 1H), 5.02-4.96 (m, 1H), 4.08 (dd, *J* = 11.0, 5.6 Hz, 2H), 3.94 (dd, *J* = 11.1, 6.4 Hz, 2H), 2.47 (dt, *J* = 7.3, 1.1 Hz, 2H), 2.10 (t, *J* = 5.8 Hz, 2H); ^{13}C NMR (125 MHz, $CDCl_3$): δ = 141.0 (e), 133.7 (o), 128.7 (o, 2C), 127.0 (o, 2C), 126.7 (o), 117.9 (e), 68.1 (e, 2C), 47.1 (e), 38.8 (o); IR (neat): $\tilde{\nu}$ = 3356 (br), 3062 (w), 2935 (w), 1639 (w), 1600 (w), 1581 (w), 1499 (m), 1467 (w), 1445 (m), 1416 (w), 1296 (w), 1267 (w), 1237 (w), 1149 (w), 1051 (m), 1031 (m), 1008 (m), 998 (m), 967 (w), 915 (m), 870 (w), 844 (w), 766 (m), 737 (m), 698 (vs), 674 (w) cm^{-1}; HRMS (CI(NH_4)) calcd for ($C_{12}H_{16}O_2$ + NH_4): 210.1489; found: 210.1495.

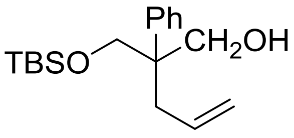

9hc

Compound 9hc. Under a N_2 atmosphere, **9hb** (13.06 mmol, 2.5 g) and imidazole (13.06 mmol, 889 mg) were dissolved in DMF (130 mL) and cooled to 0 °C. *tert*-Butyldimethylsilyl chloride (13.06 mmol, 1.97 g) was added in one batch and the reaction was stirred for 1 hour at room temperature. The reaction mixture was then quenched with a saturated aqueous solution of ammonium chloride and extracted with Et_2O. The organic layer washed several times with water and then dried over $MgSO_4$, filtered and concentrated. The crude oil was purified by flash column chromatography (petroleum ether/Et_2O = 9:1), affording **9hc** as a colourless oil (2.76 g, 69%). ^{1}H NMR (500 MHz, $CDCl_3$): δ = 7.36-7.31 (m, 4H), 7.25-7.20 (m, 1H), 5.51 (ddt, *J* = 17.1, 10.0, 7.2 Hz, 1H), 5.07-5.00 (m, 1H), 4.98-4.93 (m, 1H), 4.02 (d, *J* = 9.7 Hz, 1H), 4.00 (dd, *J* = 11.0, 5.5 Hz, 1H), 3.90 (dd, *J* = 11.1, 7.0 Hz, 1H), 3.86 (d, *J* = 9.8 Hz, 1H), 2.61 (dd, *J* = 6.8, 5.5 Hz, 1H), 2.53 (d, *J* = 7.3 Hz, 2H), 0.88 (s, 9H), 0.07 (s, 3H), 0.05 (s, 3H); ^{13}C NMR (125 MHz, $CDCl_3$): δ = 141.6 (e), 133.9 (o), 128.2 (o, 2C), 126.9 (o, 2C), 126.3 (o), 117.5 (e), 68.4 (e, 2C), 46.8 (e), 38.0 (e), 25.7 (o, 3C), 18.0 (e), −5.7 (o), −5.8 (o); IR (neat): $\tilde{\nu}$ = 3443 (br), 3063 (w), 2954 (m), 2929 (m), 2884 (w), 2857 (m), 1639 (w), 1602 (w), 1499 (w), 1472 (m), 1469 (w), 1413 (w), 1389 (w), 1362 (w), 1253 (m), 1090 (m), 1037 (w), 1006 (w), 939 (w), 914 (m), 833 (vs), 814 (w), 774 (s), 697 (s), 669 (m) cm^{-1}; HRMS (CI(NH_4)) calcd for ($C_{18}H_{30}O_2Si$ + H): 307.2088; found: 307.2097.

---

[7] D. Nečas, M. Turský, M. Kotora, *J. Am. Chem. Soc.* **2004**, *126*, 10222

Compound 9h. This compound was obtained from **9hc** (0.65 mmol, 200 mg) following the same procedure as for the preparation of **1**. Colourless oil (190 mg, 96%). ^{1}H NMR (500 MHz, $CDCl_3$): δ = 9.59 (s, 1H), 7.39-7.34 (m, 2H), 7.31-7.26 (m, 1H), 7.18-7.14 (m, 2H), 5.51 (ddt, *J* = 17.1, 10.1, 7.3 Hz, 1H), 5.07–5.00 (ddt, *J* = 17.0, 2.1, 1.4 Hz, 1H), 5.02 (ddt, *J* = 10.1, 2.0, 1.0 Hz, 1H), 4.21 (d, *J* = 9.9 Hz, 1H), 4.02 (d, *J* = 9.9 Hz, 1H), 2.80-2.75 (m, 2H), 0.85 (s, 9H), 0.02 (s, 3H), −0.01 (s, 3H); ^{13}C NMR (125 MHz, $CDCl_3$): δ = 201.5 (o), 136.6 (e), 133.0 (o), 128.7 (o, 2C), 127.5 (o, 2C), 127.4 (o), 118.5 (e), 63.5 (e), 58.8 (e), 35.2 (e), 25.7 (o, 3C), 18.1 (e), −5.77 (o), −5.79 (o); IR (neat): $\tilde{\nu}$ = 3078 (w), 2955 (m), 2929 (m), 2886 (w), 2857 (m), 2710 (w), 1731 (m), 1641 (m), 1600 (m), 1497 (w), 1472 (m), 1464 (w), 1447 (w), 1415 (w), 1389 (w), 1362 (w), 1253 (m), 1169 (w), 1099 (s), 1020 (w), 1006 (w), 939 (w), 918 (m), 835 (vs), 815 (m), 776 (s), 757 (m), 738 (w), 698 (m), 671 (w) cm^{-1}; HRMS ($CI(NH_4)$) calcd for ($C_{18}H_{28}O_2Si$ + H): 305.1931; found: 305.1929.

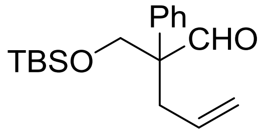

9h

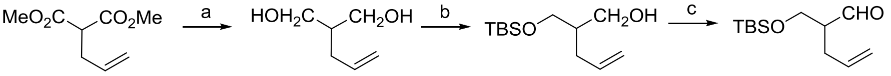

9ia **9ib** **9i**

[(a)] $LiAlH_4$, Et_2O, 63%. [(b)] NaH, TBSCl, THF, 83%. [(d)] $(COCl)_2$, DMSO, Et_3N, CH_2Cl_2, 64%.

Compound 9ia. Under a N_2 atmosphere, $LiAlH_4$ (8.71 mmol, 331 mg) was suspended in Et_2O (14 mL) and cooled to 0 °C. Dimethyl allylmalonate (5.81 mmol, 934 μL) was added *via* syringe and stirred at 0 °C for 15 minutes, before adding an additional $LiAlH_4$ (8.71 mmol, 331 mg) at 0 °C. The reaction was stirred until complete by TLC. The reaction mixture was quenched carefully at 0 °C with a saturated aqueous solution of sodium sulfate dropwise. The white precipitate was filtered over celite pad, the filtrate was evaporated under reduced pressure and the crude material was purified by flash chromatography (petroleum ether/Et_2O = 1:2) afforded **9ia** (429 mg, 63%).[8]

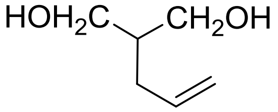

9ia

Compound 9ib. Under a N_2 atmosphere, sodium hydride (4.03 mmol, 161 mg, 60% in oil) was suspended in THF (37 mL) and cooled to 0 °C. **9ia** (3.66 mmol, 425 mg) was added and stirred for 15 minutes before adding *tert*-Butyldimethylsilyl chloride (3.66 mmol, 552 mg) in one batch and stirring until complete by TLC at room temperature. The reaction was quenched with a saturated aqueous solution of ammonium chloride, extracted with Et_2O and the organic layer was dried over $MgSO_4$, filtered and concentrated. The crude oil was purified by flash column chromatography (petroleum ether/Et_2O = 9:1), affording **9ib** as a colourless oil (700 mg, 83%). ^{1}H NMR (500 MHz, $CDCl_3$): δ = 5.78 (ddt, *J* = 17.1, 10.1, 7.1 Hz, 1H), 5.08-5.01 (m, 2H), 3.79 (ddd, *J* = 9.9, 4.2, 0.7 Hz, 1H), 3.77-3.72 (m, 1H), 3.65 (ddd, *J* = 10.9, 6.9, 4.7 Hz, 1H), 3.62 (dd, *J* = 9.9, 7.2 Hz, 1H), 2.70 (dd, *J* = 6.5, 4.8 Hz, 1H), 2.05 (tt, *J* = 10.6, 1.3 Hz, 2H), 1.87-1.79 (m, 1H), 0.90 (s, 9H), 0.07 (s, 6H); ^{13}C NMR (125 MHz, $CDCl_3$): δ = 136.3 (o), 116.2 (e), 66.1 (e), 65.6 (e), 41.8 (o), 32.3 (e), 25.8 (o, 3C), 18.1 (e), −5.7 (o), −5.9 (o); IR (neat): $\tilde{\nu}$ = 3347 (br), 3078 (w), 2954 (m), 2929 (m), 2885 (w), 2857 (m), 1641 (w), 1471 (m), 1441 (w), 1413 (w), 1389 (w), 1361 (w), 1252 (m), 1089 (m), 1038 (m), 1006 (w), 993 (w), 938 (w), 912 (m), 875 (w), 832 (vs), 813 (w), 773 (s), 668 (m) cm^{-1}; HRMS ($CI(NH_4)$) calcd for ($C_{12}H_{26}O_2Si$ + H): 231.1775; found: 231.1782.

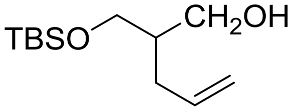

9ib

[8] K. Mori, N. Chiba, *Liebigs Ann. Chemie* **1989**, 957

Compound 9i. This compound was obtained from **9ib** (0.87 mmol, 200 mg) following the same procedure as for the preparation of **1**. Colourless oil (127 mg, 64%).^{1}H NMR (500 MHz, $CDCl_3$): δ = 9.74 (d, *J* = 1.7 Hz, 1H), 5.81-5.72 (m, 1H), 5.12-5.04 (m, 2H), 3.90 (dd, *J* = 10.3, 4.6 Hz, 1H), 3.83 (dd, *J* = 10.2, 5.8 Hz, 1H), 2.53-2.43 (m, 2H), 2.32-2.23 (m, 1H), 0.87 (s, 9H), 0.05 (s, 6H); ^{13}C NMR (125 MHz, $CDCl_3$): δ = 204.1 (o), 135.0 (o), 117.2 (e), 61.2 (e), 53.5 (o), 29.7 (e), 25.8 (o, 3C), 18.2 (e), −5.56 (o), −5.58 (o); IR (neat): $\tilde{\nu}$ = 2954 (m), 2929 (m), 2886 (w), 2857 (m), 1713 (m), 1643 (w), 1472 (m), 1441 (w), 1414 (w), 1389 (w), 1361 (w), 1253 (m), 1187 (w), 1100 (br, m), 1006 (w), 992 (w), 938 (w), 916 (m), 834 (vs), 814 (w), 776 (s), 668 (m) cm^{-1}; HRMS (CI(NH_4)) calcd for ($C_{12}H_{24}O_2Si$ + H): 229.1618; found: 229.1629.

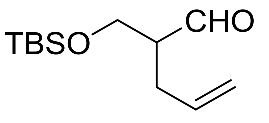

9i

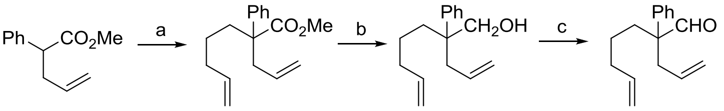

9ka **9kb** **9k**

[(a)] i) NaHMDS, DMF ; ii) bromopent-1-ene, 69%. [(b)] $LiAlH_4$, Et_2O, 87%. [(d)] $(COCl)_2$, DMSO, Et_3N, CH_2Cl_2, 93%.

Compound 9ka. Under a N_2 atmosphere, solid NaHMDS (3.16 mmol, 579 mg) was dissolved in DMF (8 mL) and cooled to 0 °C. Methyl 2-phenylpent-4-enoate[4] (1.58 mmol, 300 mg) was added *via* syringe and stirred at room temperature for 15 minutes before adding bromopent-1-ene (2.37 mmol, 254 µL) and stirring at room temperature for 16 hours. The reaction was quenched with a saturated aqueous solution of ammonium chloride and extracted with Et_2O. The organic layer was washed several times with water, then dried over $MgSO_4$, filtered and concentrated. The crude oil was purified by flash column chromatography (petroleum ether/Et_2O = 99:1), affording **9ka** as a colourless oil (279 mg, 69%). ^{1}H NMR (500 MHz, $CDCl_3$): δ = 7.35-7.29 (m, 2H), 7.26-7.21 (m, 3H), 5.74 (ddt, *J* = 17.0, 10.3, 6.7 Hz, 1H), 5.51 (dddd, *J* = 17.0, 10.2, 7.7, 6.8 Hz, 1H), 5.08–5.01 (m, 2H), 4.98 (dq, *J* = 17.1, 1.7 Hz, 1H), 4.93 (ddt, *J* = 10.1, 2.1, 1.1 Hz, 1H), 3.64 (s, 3H), 2.86-2.79 (m, 1H), 2.78-2.71 (m, 1H), 2.08-1.94 (m, 4H), 1.28-1.12 (m, 2H); ^{13}C NMR (125 MHz, $CDCl_3$): δ = 175.9 (e), 142.2 (e), 138.3 (o), 133.5 (o), 128.3 (o, 2C), 126.7 (o), 126.4 (o, 2C), 118.2 (e), 114.7 (e), 53.7 (e), 51.9 (o), 39.2 (e), 34.0 (e), 33.8 (e), 23.2 (e); IR (neat): $\tilde{\nu}$ = 3076 (w), 2978 (w), 2949 (w), 1729 (vs), 1640 (m), 1600 (w), 1497 (m), 1446 (m), 1433 (w), 1416 (w), 1208 (m), 1137 (m), 1074 (w), 1034 (w), 993 (m), 912 (s), 832 (w), 778 (w), 736 (m), 698 (vs) cm^{-1}; HRMS (CI(CH_4)) calcd for ($C_{17}H_{22}O_2$ + H): 259.1693; found: 259.1693.

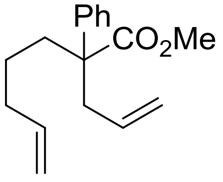

9ka

Compound 9kb. This compound was obtained from **9ka** (1.14 mmol, 278 mg) following the same procedure as for the preparation of **1b**. Colourless oil (227 mg, 87%). ^{1}H NMR (500 MHz, $CDCl_3$): δ = 7.39-7.29 (m, 4H), 7.25-7.20 (m, 1H), 5.71 (ddt, *J* = 17.2, 15.2, 7.0 Hz, 2H), 5.16-5.09 (m, 1H), 5.08-5.03 (m, 1H), 4.99–4.94 (m, 1H), 4.94–4.89 (m, 1H), 3.81-3.73 (m, 2H), 2.62 (dd, *J* = 13.9, 7.1 Hz, 1H), 2.48 (dd, *J* = 14.0, 7.5 Hz, 1H), 2.03-1.95 (m, 2H), 1.71-1.63 (m, 2H), 1.31-1.11 (m, 3H); ^{13}C NMR (125 MHz, $CDCl_3$): δ = 144.0 (e), 138.6 (o), 134.6 (o), 128.4 (o, 2C), 126.7 (o, 2C), 126.1 (o), 117.7 (e), 114.6 (e), 68.0 (e), 45.8 (e), 38.9 (e), 34.5 (e), 34.2 (e), 22.6 (e); IR (neat): $\tilde{\nu}$ = 3397 (br), 3074 (w), 2976 (w), 2935 (m), 1829 (w), 1639 (m), 1600 (w), 1580 (w), 1498 (w), 1459 (w), 1444 (m), 1415 (w), 1385 (w), 1328 (w), 1157 (w), 1043 (m), 994 (m), 909 (s), 829 (w), 766 (m), 697 (vs), 672 (w), 663 (w) cm^{-1}; HRMS (CI(NH_4)) calcd for ($C_{16}H_{22}O$ + NH_4): 248.2009; found: 248.2014.

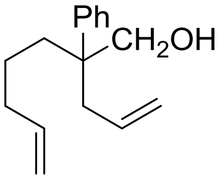

9kb

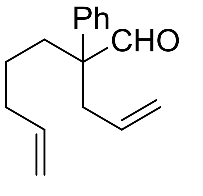

9k

Compound 9k. This compound was obtained from **9kb** (1.04 mmol, 225 mg) following the same procedure as for the preparation of **1**. Colourless oil (206 mg, 93%). ^{1}H NMR (500 MHz, $CDCl_3$): δ = 9.50 (s, 1H), 7.41-7.35 (m, 2H), 7.31-7.27 (m, 1H), 7.24-7.19 (m, 2H), 5.73 (ddt, *J* = 17.0, 10.3, 6.7 Hz, 1H), 5.53 (ddt, *J* = 17.1, 10.1, 7.2 Hz, 1H), 5.10-5.02 (m, 2H), 4.99 (dq, *J* = 17.1, 1.6 Hz, 1H), 4.95 (ddt, *J* = 10.2, 2.0, 1.2 Hz, 1H), 2.78-2.17 (m, 1H), 2.71 (m, 1H), 2.07-2.01 (m, 2H), 1.99-1.86 (m, 2H), 1.31-1.16 (m, 2H); ^{13}C NMR (125 MHz, $CDCl_3$): δ = 202.2 (o), 138.5 (e), 138.1 (o), 132.8 (o), 128.8 (o, 2C), 127.5 (o, 2C), 127.3 (o), 118.4 (e), 114.9 (e), 57.0 (e), 36.7 (e), 34.0 (e), 31.4 (e), 22.8 (e); IR (neat): $\tilde{\nu}$ = 3077 (w), 2978 (w), 2940 (w), 2864 (w), 2708 (w), 1722 (s), 1640 (m), 1599 (w), 1581 (w), 1495 (m), 1459 (w), 1446 (m), 1417 (w), 1386 (w), 1321 (w), 1078 (w), 1032 (w), 993 (m), 912 (s), 825 (w), 760 (m), 698 (vs) cm^{-1}; HRMS (CI(NH_4)) calcd for ($C_{16}H_{20}O$ + NH_4): 246.1852; found: 246.1854.

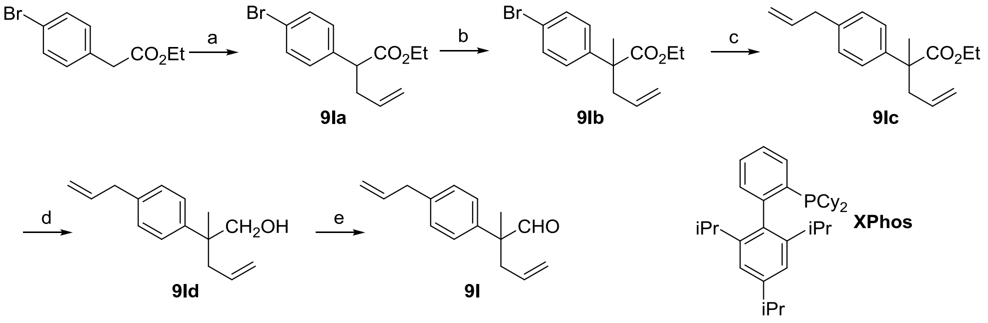

(a) i) NaH, DMF ; ii) allyl bromide, 70%. (b) i) NaH, DMF, MeI, 69%. (c) $Pd(OAc)_2$, XPhos, CsF, allyltributylstannane, dimethoxy-ethane, 80 °C, 89%. (d) $LiAlH_4$, Et_2O, 73%. (e) $(COCl)_2$, DMSO, Et_3N, CH_2Cl_2, 93%.

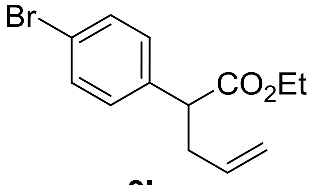

9la

Compound 9la. Under a N_2 atmosphere, NaH (6.17 mmol, 247 mg, 60% dispersion in oil) was suspended in DMF (62 mL) and the mixture was cooled to 0 °C. Ethyl 4-bromophenylacetate (6.17 mmol, 1.50 g) was added and stirred for 15 minutes before adding allyl bromide (6.17 mmol, 554 μL) at 0 °C. The reaction was stirred for 1 hour, before quenching with saturated ammonium chloride, extracting with Et_2O and washing the organic layer several times with water. The organic layer was then dried with $MgSO_4$, filtered and concentrated. The crude was purified by flash column chromatography (petroleum ether/Et_2O = 50:1), affording **9la** as a colourless oil (1.22 g, 70%). %); ^{1}H NMR (500 MHz, $CDCl_3$): δ = 7.47-7.42 (m, 2H), 7.21-7.17 (m, 2H), 5.69 (ddt, *J* = 17.1, 10.2, 6.8 Hz, 1H), 5.06 (dq, *J* = 17.1, 1.6 Hz, 1H), 5.01 (ddt, *J* = 10.2, 2.0, 1.0 Hz, 1H), 4.18–4.05 (m, 2H), 3.58 (dd, *J* = 8.1, 7.4 Hz, 1H), 2.79 (dddt, *J* = 8.3, 14.3, 7.1, 1.2 Hz, 1H), 2.51-2.44 (m, 1H), 1.21 (t, *J* = 7.1 Hz, 3H), in agreement with previously reported data.[9]

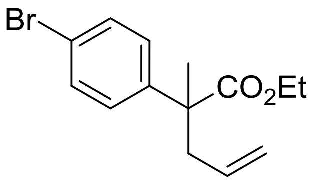

9lb

Compound 9lb. Under a N_2 atmosphere, sodium hydride (7.06 mmol, 282 mg, 60% dispersion in oil) was suspended in DMF (18 mL) and the mixture cooled to 0 °C. **9la** (3.53 mmol, 1.0 g) was added and stirred for 15 minutes before adding methyl iodide (7.06 mmol, 440 μL) at 0 °C. The reaction was stirred for 16 hours, before quenching with saturated ammonium chloride, extracting with Et_2O and washing the organic layer several times with water. The organic layer was then dried with $MgSO_4$, filtered and concentrated. The crude was purified by flash column chromatography (petroleum ether/Et_2O = 50:1), affording **9lb** as a colourless oil (723 mg, 69%). ^{1}H NMR (500 MHz, $CDCl_3$): δ = 7.46-7.42 (m, 2H), 7.21-7.16 (m, 2H), 5.63-5.53 (m, 1H), 5.09-5.03 (m, 2H), 4.12 (q, *J* = 7.1 Hz, 2H), 2.78 (dd, *J* = 13.7, 7.4 Hz, 1H), 2.66-2.59 (m, 1H), 1.51 (s, 3H), 1.19 (t, *J* = 7.1 Hz, 3H); ^{13}C NMR (125 MHz, $CDCl_3$): δ = 175.2 (e), 142.4 (e), 134.5 (o), 131.4 (o, 2C), 127.9 (o, 2C), 120.7 (e), 118.7 (e), 61.0 (e), 52.2 (o), 43.6 (e), 22.5 (o), 14.0 (o); IR (neat): $\tilde{\nu}$ = 3077 (w), 2979 (m),

[9] H. Cai, Z. Yuan, W. Zhu, G.Zhu, *Chem. Commun.* **2011**, *47*, 8682

2939 (w), 1725 (vs), 1640 (w), 1590 (w), 1490 (m), 1461 (w), 1397 (m), 1377 (w), 1288 (w), 1228 (s), 1142 (s), 1117 (w), 1082 (s), 1008 (s), 958 (w), 917 (s), 858 (w), 821 (s), 773 (w), 752 (m), 726 (w), 715 (w), 695 (w), 667 (w) cm^{-1}; HRMS (CI(NH$_4$)) calcd for ($C_{14}H_{17}{}^{79}BrO_2$ + H): 297.0485; found: 297.0494.

Compound 9lc. Pd(OAc)$_2$ (0.034 mmol, 7.6 mg), XPhos (0.067 mmol, 32 mg) and cesium fluoride (1.48 mmol, 225 mg) were added to a two-neck flask which was equipped with a reflux condenser and then flushed with N_2. **9lb** (0.67 mmol, 200 mg) in dimethoxy-ethane (6.7 mL) and allyltributylstannane (0.74 mmol, 229 µL) were syringed into flask and the mixture was heated at 80 °C for 18 hours. The mixture was cooled to room temperature and concentrated. Et$_2$O was added to the residue thus obtained and after filtration over celite, the filtrate was concentrated. Purification by flash column chromatography (pentane/Et$_2$O = 99:1) afforded **9lc** as a colourless oil (155 mg, 89%). ^{1}H NMR (500 MHz, CDCl$_3$): δ = 7.25-7.21 (m, 2H), 7.17-7.13 (m, 2H), 6.01-5.92 (m, 1H), 5.62 (ddt, *J* = 17.1, 10.1, 7.0 Hz, 1H), 5.11-5.02 (m, 4H), 4.13 (q, *J* = 7.1 Hz, 2H), 3.37 (d, *J* = 6.8 Hz, 2H), 2.82 (dd, *J* = 13.7, 7.3 Hz, 1H), 2.63 (dd, *J* = 13.7, 7.1 Hz, 1H), 1.51 (s, 3H), 1.19 (t, *J* = 7.1 Hz, 3H); ^{13}C NMR (125 MHz, CDCl$_3$): δ = 175.7 (e), 141.2 (e), 138.4 (e), 137.2 (o), 134.2 (o), 128.4 (o, 2C), 126.0 (o, 2C), 118.2 (e), 115.8 (e), 60.7 (e), 52.0 (o), 43.7 (e), 39.7 (e), 22.6 (o), 14.0 (o); IR (neat): $\tilde{\nu}$ = 3078 (w), 2979 (m), 2938 (w), 1726 (vs), 1639 (m), 1513 (m), 1459 (w), 1434 (w), 1416 (w), 1377 (w), 1280 (w), 1229 (m), 1142 (s), 1096 (m), 1019 (m), 994 (m), 957 (w), 913 (s), 857 (w), 841 (w), 814 (w), 772 (w), 726 (w), 699 (w), 662 (w) cm^{-1}; HRMS (CI(NH$_4$)) calcd for ($C_{17}H_{22}O_2$ + H): 259.1693; found: 259.1703.

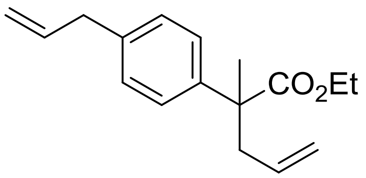

9lc

Compound 9ld. This compound was obtained from **9lc** (0.59 mmol, 153 mg) following the same procedure as for the preparation of **1b**. Colourless oil (94 mg, 73%). ^{1}H NMR (500 MHz, CDCl$_3$): δ = 7.31-7.27 (m, 2H), 7.20-7.16 (m, 2H), 5.97 (ddt, *J* = 17.1, 10.0, 6.9 Hz, 1H), 5.60 (ddt, *J* = 17.1, 10.0, 7.3 Hz, 1H), 5.12-4.96 (m, 4H), 3.73 (d, *J* = 10.9 Hz, 1H), 3.59 (d, *J* = 11.0 Hz, 1H), 3.38 (d, *J* = 6.7 Hz, 2H), 2.55 (dd, *J* = 13.7, 6.6 Hz, 1H), 2.35 (dd, *J* = 13.8, 8.1 Hz, 1H), 1.32 (s, 3H), 1.20 (br s, 1H(OH)); ^{13}C NMR (125 MHz, CDCl$_3$): δ = 142.2 (e), 137.9 (e), 137.3 (o), 134.6 (o), 128.6 (o, 2C), 126.7 (o, 2C), 17.5 (e), 115.8 (e), 71.7 (e), 42.9 (e, 2C), 39.7 (e), 21.9; IR (neat): $\tilde{\nu}$ = 3392 (br), 3077 (w), 2977 (w), 2913 (w), 2247 (w), 1638 (m), 1513 (w), 1467 (w), 1434 (w), 1415 (w), 1373 (w), 1296 (w), 1031 (m), 1018 (m), 994 (m), 906 (vs), 838 (w), 807 (w), 729 (vs) cm^{-1}; HRMS (CI(NH$_4$)) calcd for ($C_{15}H_{20}O$ + NH$_4$): 234.1852; found: 234.1861.

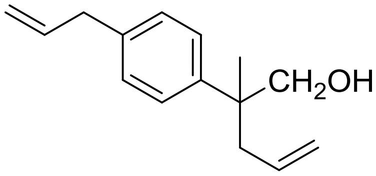

9ld

Compound 9l. This compound was obtained from **9kb** (0.43 mmol, 92 mg) following the same procedure as for the preparation of **1**. Colourless oil (85 mg, 93%).^{1}H NMR (500 MHz, CDCl$_3$): δ = 9.50 (s, 1H), 7.24-7.16 (m, 4H), 6.01–5.91 (m, 1H), 5.55 (ddt, *J* = 17.1, 10.0, 7.2 Hz, 1H), 5.11-5.01 (m, 4H), 3.38 (d, *J* = 6.7 Hz, 2H), 2.68 (dd, *J* = 14.1, 6.8 Hz, 1H), 2.62 (dd, *J* = 14.1, 7.7 Hz, 1H), 1.43 (s, 3H); ^{13}C NMR (125 MHz, CDCl$_3$): δ = 201.9 (o), 139.2 (e), 137.1 (e), 137.0 (o), 133.2 (o), 129.0 (o, 2C), 127.2 (o, 2C), 118.5 (e), 116.0 (e), 53.3 (e), 40.5 (e), 39.7 (e), 18.8 (o); IR (neat): $\tilde{\nu}$ = 3078 (w), 2978 (w), 2916 (w), 2805 (w), 2708 (w), 1722 (vs), 1639 (m), 1513 (m), 1456 (w), 1433 (w), 1416 (w), 1388 (w), 1371 (w), 1273 (w), 1189 (w), 1119 (w), 1077 (w), 1019 (m), 993 (m), 912 (vs), 841 (m), 804 (m), 733 (w), 714 (w), 660 cm^{-1}; HRMS (CI(NH$_4$)) calcd for ($C_{15}H_{18}O$ + NH$_4$): 232.1696; found: 232.1698.

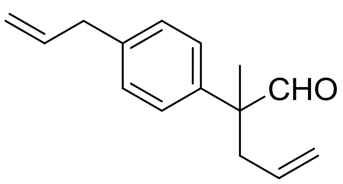

9l

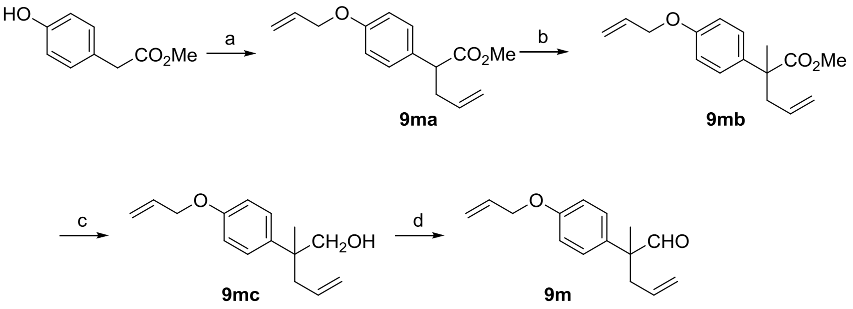

[(a)] i) NaH, THF; ii) allyl bromide, 82%. [(b)] i) NaH, DMF, MeI, 77%. [(c)] $LiAlH_4$, Et_2O, 97%. [(e)] $(COCl)_2$, DMSO, Et_3N, CH_2Cl_2, 87%.

Compound 9ma. Under a N_2 atmosphere, NaH (6.02 mmol, 282 mg, 60% dispersion in oil) was suspended in THF (30 mL) and the mixture was cooled to 0 °C. Methyl 4-hydroxyphenylacetate (3.01 mmol, 500 mg) was added and the mixture was stirred for 15 minutes before adding allyl bromide (6.02 mmol, 521 μL) at 0 °C. The mixture was stirred for 16 hours, before quenching with a saturated aqueous solution of ammonium chloride, extracting with Et_2O and washing several times the organic layer with water. The organic layer was then dried over $MgSO_4$, filtered and concentrated. Purification by flash column chromatography (petroleum ether/Et_2O = 99:1), afforded **9ma** as colourless oil (612 mg, 82%).^{1}H NMR (500 MHz, $CDCl_3$): δ = 7.24-7.19 (m, 2H), 6.89-6.84 (m, 2H), 6.05 (ddt, *J* = 17.2, 10.6, 5.3 Hz, 1H), 5.71 (ddt, *J* = 17.1, 10.2, 6.8 Hz, 1H), 5.40 (dq, *J* = 17.3, 1.6 Hz, 1H), 5.28 (dq, *J* = 10.6, 1.4 Hz, 1H), 5.06 (ddt, *J* = 17.1, 1.6, 1.5 Hz, 1H), 5.00 (ddt, *J* = 10.1, 2.0, 1.0 Hz, 1H), 4.52 (dt, *J* = 5.3, 1.5 Hz, 2H), 3.65 (s, 3H), 3.59 (dd, *J* = 8.5, 7.1 Hz, 1H), 2.89-2.74 (m, 1H), 2.53-2.44 (m, 1H), ^{13}C NMR (125 MHz, $CDCl_3$): δ = 174.1 (e), 157.8 (e), 135.3 (o), 133.2 (o), 130.8 (e), 128.9 (o, 2C), 117.6 (e), 116.9 (e), 114.8 (o, 2C), 68.8 (e), 51.9 (o), 50.5 (o), 37.6 (e); IR (neat): $\tilde{\nu}$ = 3079 (w), 2982 (w), 2951 (w), 1732 (vs), 1642 (w), 1610 (m), 1583 (w), 1509 (vs), 1456 (w), 1435 (m), 1343 (w), 1300 (w), 1242 (s), 1223 (s), 1198 (m), 1178 (w), 1159 (vs), 1119 (m), 1021 (m), 994 (s), 917 (s), 830 (s), 793 (m), 750 (w), 708 (w) cm^{-1}; HRMS (CI(NH_4)) calcd for ($C_{15}H_{18}O_3$ + H): 247.1329; found: 247.1330.

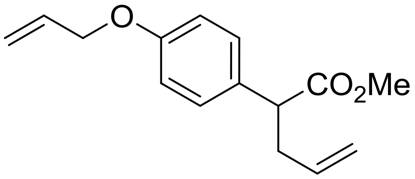

9ma

Compound 9mb. This compound was obtained from **9ma** (4.07 mmol, 1.0 g) following the same procedure as for the preparation of **9lb**. Colourless oil (817 mg, 77%). ^{1}H NMR (500 MHz, $CDCl_3$): δ = 7.25-7.20 (m, 2H), 6.89-6.84 (m, 2H), 6.05 (ddt, *J* = 17.2, 10.6, 5.3 Hz, 1H), 5.60 (ddt, *J* = 17.1, 10.0, 7.2 Hz, 1H), 5.44-5.38 (m, 1H), 5.30-5.26 (m, 1H), 5.09-5.01 (m, 2H), 4.54-4.50 (m, 2H), 3.65 (s, 3H), 2.80 (dd, *J* = 13.7, 7.4 Hz, 1H), 2.62 (dd, *J* = 13.8, 7.0 Hz, 1H), 1.51 (s, 3H); ^{13}C NMR (125 MHz, $CDCl_3$): δ = 176.4 (e), 157.3 (e), 135.5 (e), 134.1 (o), 133.3 (o), 127.0 (o, 2C), 118.3 (e), 117.6 (e), 114.5 (o, 2C), 68.7 (e), 52.0 (o), 49.2 (e), 43.8 (e), 22.6 (o); IR (neat): $\tilde{\nu}$ = 3079 (w), 2980 (w), 2950 (w), 1727 (s), 1640 (w), 1609 (m), 1581 (w), 1510 (s), 1458 (m), 1434 (m), 1377 (w), 1287 (w), 1230 (s), 1184 (s), 1143 (m), 1119 (w), 1097 (w), 1022 (m), 995 (s), 910 (s), 828 (s), 806 (w), 774 (w), 730 (vs), 664 cm^{-1}; HRMS (CI(NH_4)) calcd for ($C_{16}H_{20}O_3$ + H): 261.1485; found: 261.1491.

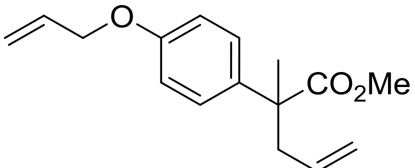

9mb

Compound 9mc. This compound was obtained from **9mb** (0.35 mmol, 90 mg) following the same procedure as for the preparation of **1b**. Colourless oil (77 mg, 97%). ^{1}H NMR (500 MHz, $CDCl_3$): δ = 7.29-7.25 (m, 2H), 6.92-6.88 (m, 2H), 6.06 (ddt, *J* = 17.2, 10.6, 5.3 Hz, 1H), 5.60 (dddd, *J* = 17.0, 10.2, 7.9, 6.7 Hz, 1H), 5.42 (dq, *J* = 17.2, 1.6 Hz, 1H), 5.29 (dq, *J* = 10.5, 1.4 Hz, 1H), 5.06-5.01 (m, 1H), 5.00-4.97 (m, 1H), 4.53 (dt, *J* = 5.3, 1.5 Hz, 2H), 3.70 (d, *J* = 10.9 Hz, 1H), 3.57 (d, *J* = 10.9 Hz, 1H), 2.55-2.49 (m,

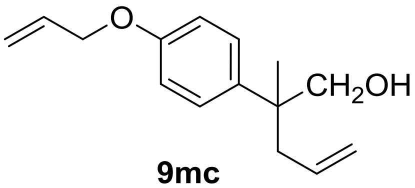

9mc

1H), 2.36-2.30 (m, 1H), 1.31 (s, 3H); ^{13}C NMR (125 MHz, $CDCl_3$): δ = 156.9 (e), 136.6 (e), 134.6 (o), 133.3 (o), 127.7 (o, 2C), 117.6 (e), 117.4 (e), 114.6 (o, 2C), 71.8 (e), 68.8 (e), 43.0 (e), 42.5 (e), 21.9 (o); IR (neat): $\tilde{\nu}$ = 3382 (br), 3074 (w), 2974 (w), 2920 (w), 2873 (w), 1638 (w), 1608 (m), 1579 (w), 1510 (vs), 1457 (m), 1425 (w), 1372 (w), 1295 (m), 1243 (s), 1185 (s), 1154 (w), 1118 (w), 1023 (s), 996 (s), 913 (s), 826 (s), 735 (m), 701 (w), 667 (w) cm^{-1}; HRMS (CI(NH_4)) calcd for ($C_{15}H_{20}O_2$ + NH_4): 250.1802; found: 250.1805.

Compound 9m. This compound was obtained from **9kb** (0.86 mmol, 200 mg) following the same procedure as for the preparation of **1**. Colourless oil (172 mg, 87%).^{1}H NMR (500 MHz, $CDCl_3$): δ = 9.46 (s, 1H), 7.18-7.13 (m, 2H), 6.95-6.90 (m, 2H), 6.05 (ddt, *J* = 17.3, 10.5, 5.3 Hz, 1H), 5.55 (dddd, *J* = 17.0, 10.1, 7.6, 6.9 Hz, 1H), 5.41 (dq, *J* = 17.2, 1.6 Hz, 1H), 5.29 (dq, *J* = 10.5, 1.4 Hz, 1H), 5.08-5.01 (m, 2H), 4.53 (dt, *J* = 5.3, 1.5 Hz, 2H), 2.69-2.63 (m, 1H), 2.59 (ddt, *J* = 14.1, 7.7, 1.0 Hz, 1H), 1.41 (s, 3H); ^{13}C NMR (125 MHz, $CDCl_3$): δ = 201.8 (o), 157.8 (e), 133.3 (o), 133.1 (o), 131.3 (e), 128.3 (o, 2C), 118.4 (e), 117.7 (e), 115.0 (o, 2C), 68.8 (e), 52.9 (e), 40.5 (e), 18.8 (o); IR (neat): $\tilde{\nu}$ = 3078 (w), 2978 (w), 2931 (w), 2805 (w), 2709 (w), 1720 (s), 1640 (w), 1606 (m), 1579 (w), 1510 (vs), 1457 (m), 1425 (w), 1387 (w), 1371 (w), 1297 (m), 1247 (s), 1184 (s), 1155 (w), 1118 (w), 1020 (m), 995 (s), 916 (s), 826 (s), 728 (m) cm^{-1}; HRMS (CI(NH_4)) calcd for ($C_{15}H_{18}NO_2$ + NH_4): 248.1645; found: 248.1650.

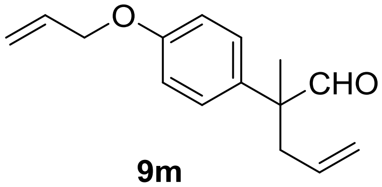

9m

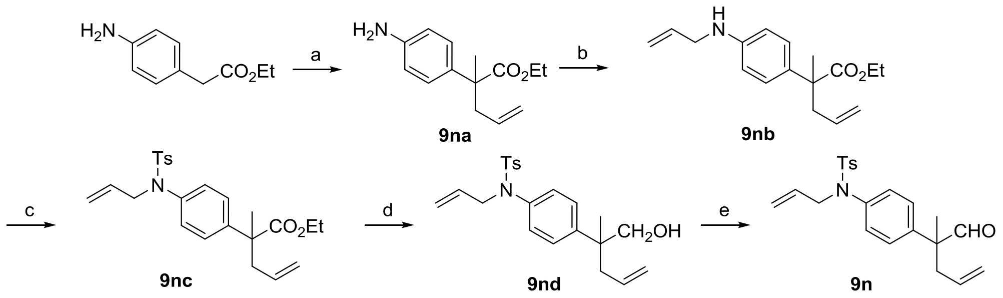

[a] i) Benzaldehyde, $MgSO_4$, CH_2Cl_2; ii) LDA, allyl bromide, –78 °C to r.t.; iii) LDA, MeI, –78 °C to r.t., 78% over three steps. [b] i) K_2CO_3, allyl bromide, DMF, 44%. [c] TsCl, Et_3N, CH_2Cl_2, 74%. [d] $LiAlH_4$, Et_2O, 86%. [e] $(COCl)_2$, DMSO, Et_3N, CH_2Cl_2, 80%.

Compound 9na. Ethyl (4-aminophenyl)acetate (2.79 mmol, 500 mg), magnesium sulfate (500 mg) and benzaldehyde (3.35 mmol, 342 μL) were dissolved in DCM (5 mL) and stirred for 16 hours at room temperature. The reaction was filtered and concentrated, affording a crude product that was used in the next step without further purification. This material (2.79 mmol, 745 mg) was used in the allylation procedure followed for the preparation of **1a**, except that nBu_4NI was not added. The crude residue thus obtained was then used in the methylation procedure described for the preparation of **9bb**. Upon completion, the mixture was cooled to 0 °C and then quenched carefully with concentrated hydrochloric acid. After basifying the mixture with 10% sodium hydroxide solution, it was extracted with Et_2O. The organic layer was dried over $MgSO_4$ and the solvent removed under reduced pressure. Purification by flash column chromatography (petroleum ether/Et_2O = 4:1) afforded **9na** as a colourless oil (465 mg, 78%). ^{1}H NMR (500 MHz, $CDCl_3$): δ = 7.15-7.08 (m, 2H), 6.66-6.62 (m, 2H), 5.63 (ddt, *J* = 17.1, 10.0, 7.1 Hz, 1H), 5.09-5.01 (m, 2H), 4.10 (q, *J* = 7.1 Hz, 2H), 3.61 (br s, 2H), 2.82-2.75 (m, 1H), 2.60 (ddt, *J* = 13.7, 7.1, 1.2 Hz, 1H), 1.48 (s, 3H), 1.18 (t, *J* = 7.1 Hz, 3H); ^{13}C NMR (125 MHz, $CDCl_3$): δ = 176.0 (e), 144.9 (e), 134.4 (o), 133.3 (e), 126.8 (o, 2C), 118.0 (e), 114.9 (o, 2C), 60.6 (e), 48.8 (e), 43.7 (e), 22.5 (o), 14.0 (o); IR (neat): $\tilde{\nu}$ = 2978 (w), 2937 (w), 1714 (s), 1623 (s), 1515 (vs), 1464 (m), 1444 (m), 1377 (m), 1277 (m), 1227 (s), 1189 (m), 1172 (m), 1142 (m), 1098 (m), 1020 (m), 997 (m), 956 (w), 916 (m), 859 (m), 826 (m), 774 (w), 744 (w), 658 (w) cm^{-1}; HRMS (CI(NH_4)) calcd for ($C_{14}H_{19}NO_2$ + H): 234.1489; found: 234.1496.

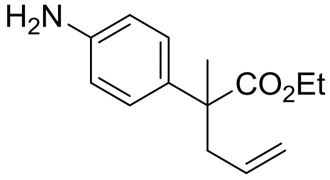

9na

Compound 9nb. Under a N_2 atmosphere, **9na** (1.99 mmol, 465 mg), potassium carbonate (2.19 mmol, 302 mg) dissolved in DMF (4 mL) was stirred at room temperature for 5 minutes before adding allyl bromide (1.99 mmol, 172 μL). After stirring for 16 hours, the mixture was quenched with water and extracted with Et_2O. The organic phase was dried over $MgSO_4$ and the solvent was removed under reduced pressure. Purification by flash column chromatography (petroleum ether/Et_2O = 5:1) afforded **9nb** as a colourless oil (236 mg, 44%). ^{1}H NMR (500 MHz, $CDCl_3$): δ = 7.18-7.09 (m, 2H), 6.60-6.56 (m, 2H), 6.00-5.91 (m, 1H), 5.64 (ddt, *J* = 17.1, 10.0, 7.1 Hz, 1H), 5.31-5.25 (m, 1H), 5.18-5.14 (m, 1H), 5.09-5.00 (m, 2H), 4.11 (q, *J* = 7.1 Hz, 2H), 3.79-3.72 (m, 2H + 1H(NH)), 2.79 (dd, *J* = 13.8, 7.3 Hz, 1H), 2.63-2.56 (m, 1H), 1.48 (s, 3H), 1.19 (t, *J* = 7.1 Hz, 3H); ^{13}C NMR (125 MHz, $CDCl_3$): δ = 176.1 (e), 146.6 (e), 135.5 (o), 134.6 (o), 132.3 (e), 126.8 (o, 2C), 117.9 (e), 116.2 (e), 112.7 (o, 2C), 60.6 (e), 48.8 (e), 46.6 (e), 43.8 (e), 22.5 (o), 14.0 (o); IR (neat): $\tilde{\nu}$ = 3076 (w), 2978 (w), 2936 (w), 1719 (s), 1640 (w), 1614 (m), 1519 (vs), 1464 (w), 1445 (w), 1418 (w), 1376 (w), 1324 (w), 1228 (m), 1193 (m), 1142 (m), 1096 (m), 1022 (m), 996 (m), 916 (m), 859 (w), 822 (w), 774 (w), 655 (w) cm^{-1}; HRMS (CI(NH_4)) calcd for ($C_{17}H_{23}NO_2$ + H): 274.1802; found: 274.1811.

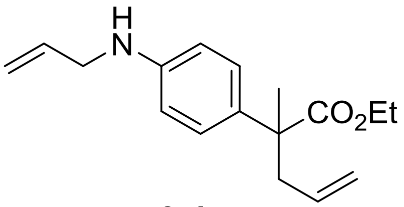

9nb

Compound 9nc. Under a N_2 atmosphere, **9nb** (0.86 mmol, 236 mg) was dissolved in CH_2Cl_2 (4.3 mL). Triethylamine (1.30 mmol, 180 μL) was added at room temperature and the mixture was stirred for 5 minutes before adding tosyl chloride (1.30 mmol, 247 mg). The reaction was stirred until complete by TLC, and was quenched with a saturated aqueous solution of ammonium chloride and extracted with Et_2O. The organic layer was dried over $MgSO_4$, filtered and concentrated. Purification by flash column chromatography (petroleum ether/Et_2O = 9:1) afforded **9nc** as a colourless oil (272 mg, 74%). ^{1}H NMR (500 MHz, $CDCl_3$): δ = 7.50-7.45 (m, 2H), 7.25-7.20 (m, 4H), 7.01-6.97 (m, 2H), 5.73 (ddt, *J* = 16.8, 10.5, 6.4 Hz, 1H), 5.58 (ddt, *J* = 17.2, 9.9, 7.4 Hz, 1H), 5.11-5.02 (m, 4H), 4.17-4.13 (m, 2H), 4.13 (q, *J* = 7.2 Hz, 2H), 2.79 (dd, *J* = 13.7, 7.3 Hz, 1H), 2.60 (dd, *J* = 13.7, 7.2 Hz, 1H), 2.42 (s, 3H), 1.50 (s, 3H), 1.18 (t, *J* = 7.1 Hz, 3H); ^{13}C NMR (125 MHz, $CDCl_3$): δ = 175.2 (e), 143.4 (e), 142.9 (e), 137.6 (e), 135.4 (e), 133.7 (o), 132.7 (o), 129.3 (o, 2C), 128.4 (o, 2C), 127.6 (o, 2C), 126.5 (o, 2C), 118.6 (e), 118.5 (e), 60.8 (e), 53.4 (e), 49.6 (e), 43.7 (e), 22.4 (o), 21.4 (o), 13.9 (o); IR (neat): $\tilde{\nu}$ = 3077 (w), 2980 (w), 2933 (w), 1725 (s), 1641 (w), 1598 (w), 1508 (m), 1457 (m), 1418 (w), 1378 (w), 1350 (s), 1304 (w), 1228 (m), 1185 (vs), 1164 (w), 1145 (w), 1118 (w), 1092 (m), 997 (w), 923 (w), 865 (m), 815 (m), 763 (w), 710 (w), 663 (s), 587 (s), 548 (s) cm^{-1}; HRMS (CI(NH_4)) calcd for ($C_{24}H_{29}NO_4S$ + H): 428.1890; found: 428.1904.

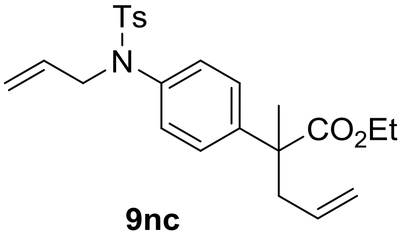

9nc

Compound 9nd. This compound was obtained from **9nc** (0.64 mmol, 272 mg) following the same procedure as for the preparation of **1b**. Colourless oil (210 mg, 86%). ^{1}H NMR (500 MHz, $CDCl_3$): δ = 7.51-7.46 (m, 2H), 7.31-7.22 (m, 4H), 7.05-6.99 (m, 2H), 5.74 (ddt, *J* = 16.2, 11.1, 6.1 Hz, 1H), 5.55 (dddd, *J* = 16.9, 10.2, 7.9, 6.7 Hz, 1H), 5.09 (dq, *J* = 17.1, 1.4 Hz, 1H), 5.06–4.97 (m, 3H), 4.20-4.11 (m, 2H), 3.73 (dd, *J* = 11.0, 5.8 Hz, 1H), 3.60 (dd, *J* = 11.0, 7.3 Hz, 1H), 2.49 (dd, *J* = 13.9, 6.7 Hz, 1H), 2.43 (s, 3H), 2.35 (dd, *J* = 13.9, 7.8 Hz, 1H), 1.31 (s, 3H), 1.23 (dd, *J* = 7.3, 5.9 Hz, 1H (OH)); ^{13}C NMR (125 MHz, $CDCl_3$): δ = 144.4 (e), 143.4 (e), 137.2 (e), 135.5 (e), 134.2 (o), 132.9 (o), 129.4 (o, 2C), 128.5 (o, 2C), 127.6 (o, 2C), 127.2 (o, 2C), 118.6 (e), 117.7 (e), 71.3 (e), 53.5 (e), 43.1 (e), 43.0 (e), 21.8 (o), 21.5 (o); IR (neat): $\tilde{\nu}$ = 3427 (br), 3074 (w), 2973 (w), 2923 (w), 2873 (w), 1640 (w), 1598 (w), 1509 (m), 1454 (w), 1417 (w), 1345 (s), 1306 (w), 1290 (w), 1226 (w), 1185 (w), 1163 (vs), 1091 (m), 1063 (w), 1038 (w), 1018 (w), 998 (w), 922 (m), 868 (m), 815 (m), 764 (w), 741 (w), 707 (w), 664 (s), 599 (m), 582 (m), 549 (s) cm^{-1}; HRMS (CI(NH_4)) calcd for ($C_{22}H_{27}NO_3S$ + H): 386.1784; found: 386.1788.

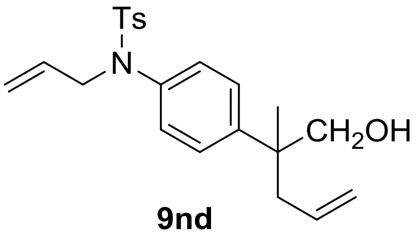

9nd

Compound 9n. This compound was obtained from **9ne** (0.54 mmol, 210 mg) following the same procedure as for the preparation of **1**. Colourless oil (166 mg, 80%). ^{1}H NMR (500 MHz, $CDCl_3$): δ = 9.49 (s, 1H), 7.50-7.41 (m, 2H), 7.26-7.21 (m, 2H), 7.18-7.13 (m, 2H), 7.07-7.02 (m, 2H), 5.70 (ddt, *J* = 16.8, 10.5, 6.3 Hz, 1H), 5.50 (ddt, *J* = 16.8, 9.1, 7.8 Hz, 1H), 5.09-4.98 (m, 4H), 4.14 (d, *J* = 6.2 Hz, 2H), 2.66-2.55 (m, 2H), 2.41 (s, 3H), 1.41 (s, 3H); ^{13}C NMR (125 MHz, $CDCl_3$): δ = 201.5 (o), 143.5 (e), 138.9 (e), 138.2 (e), 135.2 (e), 132.8 (o), 132.6 (o), 129.4 (o, 2C), 128.8 (o, 2C), 127.6 (o, 4C), 118.8 (e), 118.7 (e), 53.32 (e), 53.28 (e), 40.6 (e), 21.4 (o), 18.7 (o); IR (neat): $\tilde{\nu}$ = 3076 (w), 2978 (w), 2924 (w), 2809 (w), 2711 (w), 1721 (s), 1641 (w), 1598 (m), 1507 (m), 1454 (w), 1417 (w), 1345 (s), 1306 (w), 1290 (w), 1224 (w), 1185 (w), 1161 (vs), 1118 (w), 1090 (s), 1064 (m), 1018 (m), 995 (m), 920 (s), 865 (s), 814 (s), 764 (m), 740 (w), 707 (m), 660 (vs), 583 (vs), 546 (vs) cm^{-1}; HRMS (CI(NH_4)) calcd for ($C_{22}H_{25}NO_3S$ + H): 384.1628; found: 384.1641.

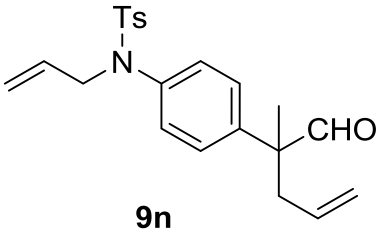

9n

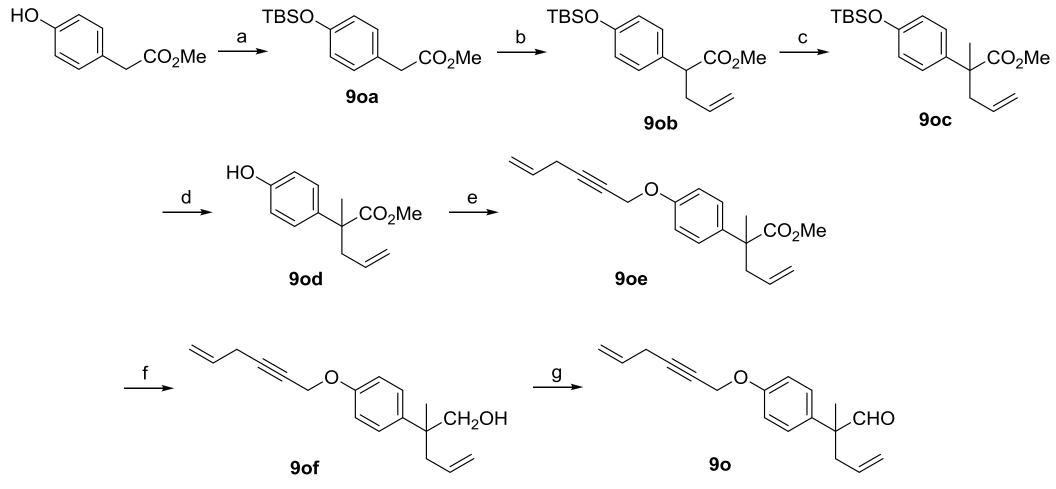

[(a)] TBSCl, Et_3N, DMF, 88%. [(b)] i) LDA, THF, –78 °C; ii) allyl bromide, nBu_4NI, –78 °C to r.t., 85%. [(c)] i) LDA, THF, –78 °C ; ii) MeI, –78 °C to r.t., 76%. [(d)] $nBu_4F•3H_2O$, THF, 87%. [(e)] PPh_3, diethyl azodicarboxylate, hex-5-en-2-yn-1-ol, THF, 59%. [(f)] $LiAlH_4$, Et_2O, 96%. [(g)] $(COCl)_2$, DMSO, Et_3N, CH_2Cl_2, 89%.

Compound 9oa. Methyl 4-hydroxyphenylacetate (12.04 mmol, 2.0 g) was dissolved in DMF (30 mL), triethylamine (13.24 mmol, 1.84 mL) was added and stirred for 10 minutes at room temperature before adding *tert*-Butyldimethylsilyl chloride (13.24 mmol, 2.0 g), stirring for 1 hour at room temperature. The mixture was quenched with a saturated aqueous solution of ammonium chloride, extracted with Et_2O and the combined organic layers were washed several times with water, then dried over $MgSO_4$, filtered and concentrated. Purification by flash column chromatography (petroleum ether/Et_2O = 19:1) afforded **9oa** as a colourless oil (2.98 g, 88%).^{1}H NMR (500 MHz, $CDCl_3$): δ = 7.15-7.10 (m, 2H), 6.81-6.76 (m, 2H), 3.69 (s, 3H), 3.55 (s, 2H), 0.98 (s, 9H), 0.19 (s, 6H), in agreement with data previously reported.[10]

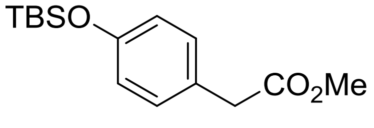

9oa

[10] A. S. K. Hashmi, L. Schwartz, J. W. Bats, *J. Prakt. Chem.* **2000**, *342*, 40

Compound 9ob. This compound was obtained from **9oa** (9.96 mmol, 2.79 g) following the same procedure as for the preparation of **1a**. Colourless oil (2.71 g, 85%). ^{1}H NMR (500 MHz, $CDCl_3$): δ = 7.17-7.14 (m, 2H), 6.79-6.75 (m, 2H), 5.70 (ddt, J = 17.1, 10.2, 6.8 Hz, 1H), 5.06 (ddt, J = 17.1, 1.5, 1.4 Hz, 1H), 5.01-4.97 (m, 1H), 3.65 (s, 3H), 3.57 (dd, J = 8.6, 6.9 Hz, 1H), 2.82-2.74 (m, 1H), 2.50-2.43 (m, 1H), 0.97 (s, 9H), 0.19 (s, 6H); ^{13}C NMR (125 MHz, $CDCl_3$): δ = 174.1 (e), 154.9 (e), 135.4 (o), 131.2 (e), 128.9 (o, 2C), 120.1 (o, 2C), 116.8 (e), 51.9 (o), 50.6 (o), 37.8 (e), 25.7 (o, 3C), 18.2 (e), –4.4 (o), –4.6 (o); IR (neat): $\tilde{\nu}$ = 2953 (m), 2931 (m), 2859 (m), 1738 (s), 1642 (w), 1608 (m), 1509 (vs), 1472 (w), 1435 (w), 1390 (w), 1362 (w), 1344 (w), 1260 (vs), 1197 (m), 1161 (s), 994 (w), 914 (s), 839 (s), 808 (m), 780 (s), 695 (w) cm^{-1}; HRMS (ESI) calcd for ($C_{18}H_{28}O_3Si$ + Na): 343.1705; found: 343.1702.

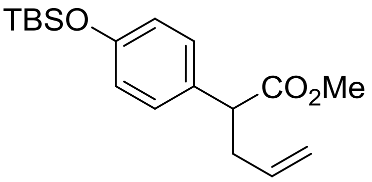

9ob

Compound 9oc. This compound was obtained from **9ob** (8.42 mmol, 2.7 g) following the same procedure as for the preparation of **9bb**. Colourless oil (2.12 g, 76%). ^{1}H NMR (500 MHz, $CDCl_3$): δ = 7.18-7.13 (m, 2H), 6.80-6.75 (m, 2H), 5.60 (ddt, J = 17.1, 10.1, 7.2 Hz, 1H), 5.08-5.01 (m, 2H), 3.65 (s, 3H), 2.79 (dd, J = 13.7, 7.4 Hz, 1H), 2.61 (dd, J = 13.7, 7.1 Hz, 1H), 1.50 (s, 3H), 0.97 (s, 9H), 0.19 (s, 6H); ^{13}C NMR (125 MHz, $CDCl_3$): δ = 176.4 (e), 154.3 (e), 135.9 (e), 134.2 (o), 127.0 (o, 2C), 119.7 (o, 2C), 118.2 (e), 52.0 (o), 49.3 (e), 43.9 (e), 25.6 (o, 3C), 22.6 (o), 18.1 (e), –4.4 (o, 2C); IR (neat): $\tilde{\nu}$ = 2952 (m), 2931 (m), 2887 (w), 2858 (w), 1731 (s), 1640 (w), 1607 (m), 1509 (s), 1472 (m), 1463 (m), 1434 (w), 1412 (w), 1390 (w), 1377 (w), 1362 (w), 1255 (s), 1178 (m), 1143 (m), 1111 (w), 1093 (w), 1013 (w), 995 (w), 912 (vs), 835 (vs), 807 (s), 779 (vs), 735 (w), 697 (w), 663 (w) cm^{-1}; HRMS (CI(NH_4)) calcd for ($C_{19}H_{30}O_3Si$ + H): 337.2050; found: 337.2058.

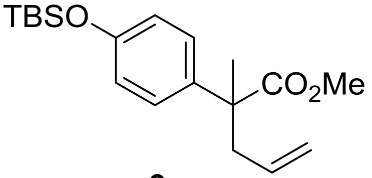

9oc

Compound 9od. Intermediate **9oc** (6.35 mmol, 2.12 g) was dissolved in THF (32 mL). Tetrabutylammonium fluoride trihydrate (7.62 mmol, 2.4 g) was added in one portion and the mixture was stirred at room temperature for 1 hour. The reaction was quenched with water, extracted with Et_2O and the organic phase dried (magnesium sulfate), filtered and concentrated. The resultant crude was purified by flash column chromatography (petroleum ether/Et_2O = 9:1) afforded **9of** as a colourless oil (1.22 g, 87%). ^{1}H NMR (500 MHz, $CDCl_3$): δ = 7.21-7.16 (m, 2H), 6.81-6.76 (m, 2H), 5.60 (ddt, J = 17.1, 10.1, 7.2 Hz, 1H), 5.09-5.02 (m, 2H), 4.68 (br s, 1H (OH)), 3.65 (s, 3H), 2.81-2.76 (m, 1H), 2.62 (ddt, J = 13.7, 7.0, 1.2 Hz, 1H), 1.50 (s, 3H); ^{13}C NMR (125 MHz, $CDCl_3$): δ = 176.9 (e), 154.5 (e), 135.2 (e), 134.0 (o), 127.2 (o, 2C), 118.4 (e), 115.2 (o, 2C), 52.2 (o), 49.2 (e), 43.7 (e), 22.6 (o); IR (neat): $\tilde{\nu}$ = 3398 (br), 3076 (w), 2979 (w), 2951 (w), 1728 (m), 1703 (s), 1640 (w), 1613 (m), 1594 (m), 1514 (vs), 1434 (m), 1378 (m), 1220 (vs), 1180 (s), 1144 (s), 1111 (m), 1093 (w), 1013 (w), 995 (m), 957 (w), 916 (m), 856 (w), 830 (s), 775 (w), 754 (w), 737 (m), 704 (w), 661 (w) cm^{-1}; HRMS (CI(NH_4)) calcd for ($C_{13}H_{16}O_3$ + H): 221.1172; found: 221.1179.

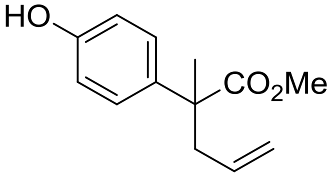

9od

Compound 9oe. In a schlenk flask under N_2, **9od** (0.91 mmol, 200 mg), triphenyl phosphine (1.00 mmol, 262 mg), diethyl azodicarboxylate (1.00 mmol, 174 mg) and hex-5-en-2-yn-1-ol[11] (1.36 mmol, 131 mg) were added in THF (3.6 mL). The flask was sealed and refluxed at 70 °C for 18 hours. The reaction was concentrated, and the crude residue was purified by flash column chromatography (petroleum ether/Et_2O = 99:1), affording **9oe** as a colourless oil (160 mg, 59%). ^{1}H NMR (500 MHz, $CDCl_3$): δ = 7.26-

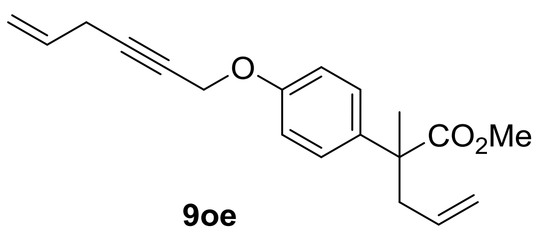

9oe

[11] Prepared according to T. Yoshinori, K. Ohmori, M. G. Schrems, A. Pfaltz, K. Suzuki, *Angew. Chem. Int. Ed.* **2010**, *49*, 881.

7.21 (m, 2H), 6.96-6.91 (m, 2H), 5.79 (ddt, J = 16.8, 10.2, 5.2 Hz, 1H), 5.60 (ddt, J = 17.1, 10.1, 7.2 Hz, 1H), 5.28 (dtd, J = 17.0, 1.9, 1.5 Hz, 1H), 5.11 (dq, J = 10.0, 1.7Hz, 1H), 5.09-5.02 (m, 2H), 4.69 (t, J = 2.1 Hz, 2H), 3.65 (s, 3H), 3.03-3.00 (m, 2H), 2.83-2.77 (m, 1H), 2.63 (ddt, J = 13.7, 7.0, 1.2 Hz, 1H), 1.51 (s, 3H); ^{13}C NMR (125 MHz, $CDCl_3$): δ = 176.4 (e), 156.6 (e), 136.0 (e), 134.1 (o), 131.9 (o), 127.1 (o, 2C), 118.3 (e), 116.4 (e), 114.7 (o, 2C), 84.6 (e), 77.3 (e), 56.4 (e), 52.1 (o), 49.3 (e), 43.8 (e), 23.1 (e), 22.7 (o); IR (neat): $\tilde{\nu}$ = 3077 (w), 2980 (w), 2950 (w), 1727 (vs), 1641 (m), 1608 (m), 1582 (w), 1510 (vs), 1457 (m), 1434 (m), 1417 (w), 1376 (m), 1282 (m), 1223 (s), 1184 (s), 1142 (m), 1119 (w), 1097 (m), 1052 (w), 1011 (s), 915 (s), 828 (s), 805 (w), 774 (w), 751 (w), 735 (w) cm^{-1}; HRMS (CI(NH_4)) calcd for ($C_{19}H_{22}O_3$ + H): 299.1642; found: 299.1654.

Compound 9of. This compound was obtained from **9oe** (0.53 mmol, 158 mg) following the same procedure as for the preparation of **1b**. Colourless oil (138 mg, 96%). ^{1}H NMR (500 MHz, $CDCl_3$): δ = 7.31-7.26 (m, 2H), 6.99-6.93 (m, 2H), 5.80 (ddt, J = 17.0, 10.1, 5.3 Hz, 1H), 5.60 (dddd, J = 17.0, 10.2, 7.9, 6.7 Hz, 1H), 5.28 (dq, J = 17.0, 1.8 Hz, 1H), 5.11 (dq, J = 10.0, 1.7 Hz, 1H), 5.07-5.01 (m, 1H), 5.01-4.97 (m, 1H), 4.70 (t, J = 2.2 Hz, 2H), 3.71 (d, J = 11.0 Hz, 1H), 3.57 (d, J = 10.9 Hz, 1H), 3.04-3.00 (m, 2H), 2.55-2.49 (m, 1H), 2.36-2.30 (m, 1H), 1.31 (s, 4H); ^{13}C NMR (125 MHz, $CDCl_3$): δ = 156.1 (e), 137.1 (e), 134.5 (o), 131.9 (o), 127.7 (o, 2C), 117.4 (e), 116.3 (e), 114.7 (o, 2C), 84.5 (e), 77.3 (e), 71.7 (e), 56.3 (e), 43.0 (e), 42.5 (e), 23.0 (e), 21.9 (o); IR (neat): $\tilde{\nu}$ = 3410 (br), 3074 (w), 2974 (w), 2917 (w), 1640 (m), 1608 (m), 1580 (w), 1510 (vs), 1456 (w), 1416 (w), 1372 (m), 1296 (m), 1262 (w), 1225 (s), 1185 (s), 1138 (w), 1117 (w), 1011 (vs), 951 (w), 913 (s), 826 (s), 805 (w), 734 (w) cm^{-1}; HRMS (CI(NH_4)) calcd for ($C_{18}H_{22}O_2$ + NH_4): 288.1958; found: 288.1968.

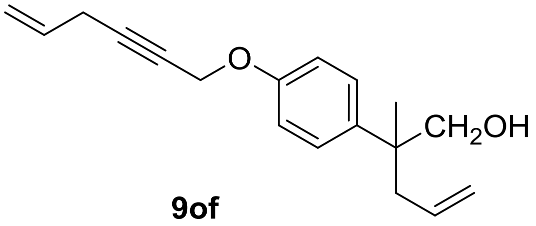

9of

Compound 9o. This compound was obtained from **9of** (0.51 mmol, 137 mg) following the same procedure as for the preparation of **1**. Colourless oil (121 mg, 89%).^{1}H NMR (500 MHz, $CDCl_3$): δ = 9.47 (s, 1H), 7.20-7.16 (m, 2H), 7.01-6.97 (m, 2H), 5.79 (ddt, J = 17.0, 10.0, 5.3 Hz, 1H), 5.55 (dddd, J = 17.0, 10.1, 7.7, 6.9 Hz, 1H), 5.27 (dtd, J = 17.0, 1.9, 1.5 Hz, 1H), 5.10 (dq, J = 10.0, 1.7 Hz, 1H), 5.06 (ddt, J = 16.7, 2.1, 1.4 Hz, 1H), 5.03 (ddt, J = 9.8, 2.0, 1.1 Hz, 1H), 4.71 (t, J = 2.2 Hz, 2H), 3.03-3.00 (m, 2H), 2.67 (ddt, J = 14.1, 6.9, 1.3 Hz, 1H), 2.60 (ddt, J = 14.1, 7.7, 1.1 Hz, 1H), 1.42 (s, 3H); ^{13}C NMR (125 MHz, $CDCl_3$): δ = 201.7 (o), 156.9 (e), 133.2 (o), 131.8 (o, e, 2C), 128.2 (o, 2C), 118.4 (e), 116.3 (e), 115.1 (o, 2C), 84.7 (e), 77.1 (e), 56.3 (e), 52.8 (e), 40.5 (e), 23.0 (e), 18.8 (o); IR (neat): $\tilde{\nu}$ = 3079 (w), 2978 (w), 2927 (w), 2809 (w), 2709 (w), 1720 (vs), 1641 (m), 1607 (m), 1580 (w), 1509 (vs), 1457 (w), 1417 (w), 1372 (m), 1297 (m), 1262 (w), 1243 (w), 1227 (s), 1185 (s), 1138 (w), 1118 (w), 1050 (w), 1010 (s), 914 (s), 826 (s), 806 (w), 728 (m) cm^{-1}; HRMS (CI(NH_4)) calcd for ($C_{18}H_{20}O_2$ + NH_4): 286.1802; found: 286.1811.

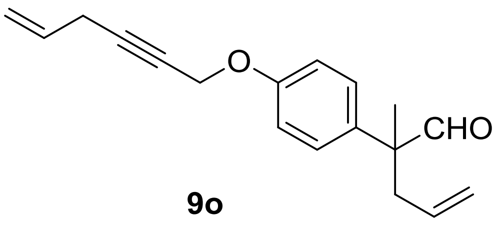

9o

Preparation of compounds 11, 13, 15,[12] and 18

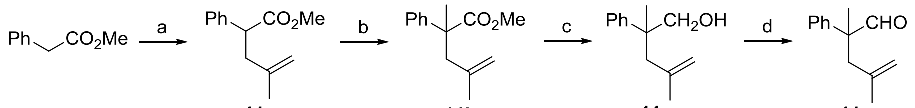

11a 11b 11c 11

[(a)] i) LDA, THF, –78 °C; ii) 3-Bromo-2-methylpropene, nBu_4NI, –78 °C to r.t., 67%. [(b)] i) LDA, THF, –78 °C ; ii) MeI, –78 °C to r.t., 95%. [(c)] $LiAlH_4$, Et_2O, 84%. [(d)] $(COCl)_2$, DMSO, Et_3N, CH_2Cl_2, 91%.

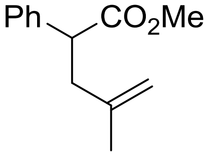

11a

Compound 11a. This compound was obtained from methyl phenylacetate (6.66 mmol, 0.96 mL) the same procedure as for the preparation of **1a**, except that 3-Bromo-2-methylpropene (7.32 mmol, 0.74 mL) was used. Colourless oil (915 mg, 67%); ^1H NMR (500 MHz, $CDCl_3$): δ = 7.35-7.29 (m, 4H), 7.29-7.23 (m, 1H), 4.75 (s, 1H), 4.69 (s, 1H), 3.81 (dd, J = 9.1, 6.4 Hz, 1H), 3.65 (s, 3H), 2.84 (dd, J = 14.7, 9.1 Hz, 1H), 2.44 (dd, J = 14.7, 6.4 Hz, 1H), 1.72 (s, 3H), in agreement with previously reported data.[4]

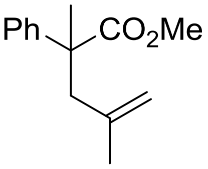

11b

Compound 11b. This compound was obtained from **11a** (4.48 mmol, 915 mg) following the same procedure as for the preparation of **9bb**. Colourless oil (933 mg, 95%). ^1H NMR (500 MHz, $CDCl_3$): δ = 7.38-7.29 (m, 4H), 7.26-7.22 (m, 1H), 4.84-4.82 (m, 1H), 4.67-4.44 (m, 1H), 3.65 (s, 3H), 2.96 (d, J = 13.6 Hz, 1H), 2.64 (d, J = 13.6 Hz, 1H), 1.56 (s, 3H), 1.48 (s, 3H); ^{13}C NMR (125 MHz, $CDCl_3$): δ = 176.6 (e), 143.9 (e), 142.1 (e), 128.3 (o, 2C), 126.8 (o), 126.0 (o, 2C), 115.2 (e), 52.1 (o), 49.5 (e), 47.0 (e), 23.7 (o), 22.3 (o); IR (neat): $\tilde{\nu}$ = 3073 (w), 2982 (w), 2949 (w), 1731 (vs), 1644 (w), 1600 (w), 1497 (w), 1446 (m), 1434 (w), 1378 (w), 1301 (w), 1260 (w), 1204 (m), 1144 (w), 1105 (m), 1075 (w), 1032 (w), 986 (w), 896 (m), 821 (w), 763 (w), 730 (w), 698 (m) cm^{-1}; HRMS (CI(NH_4)) calcd for ($C_{14}H_{18}O_2$ + H): 219.1378; found: 219.1379; elemental analysis (%) calcd for $C_{14}H_{18}O_2$: C 77.03, H 8.31; found: C 77.37, H 8.34.

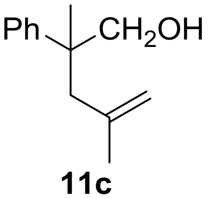

11c

Compound 11c. This compound was obtained from **11b** (2.29 mmol, 500 mg) following the same procedure as for the preparation of **1b**. Colourless oil (364 mg, 84%). ^1H NMR (500 MHz, $CDCl_3$): δ = 7.41-7.31 (m, 4H), 7.25-7.20 (m, 1H), 4.77-4.74 (m, 1H), 4.59-4.56 (m, 1H), 3.80 (dd, J = 10.9, 5.1 Hz, 1H), 3.60 (dd, J = 10.9, 8.2 Hz, 1H), 2.50 (d, J = 13.6 Hz, 1H), 2.33 (dd, J = 13.6, 0.6 Hz, 1H), 1.38 (s, 3H), 1.35-1.33 (m, 3H), 1.24 (dd, J = 8.2, 5.1 Hz, 1H (OH)); ^{13}C NMR (125 MHz, $CDCl_3$): δ = 144.9 (e), 142.5 (e), 128.2 (o, 2C), 126.7 (o, 2C), 126.0 (o), 114.3 (e), 72.0 (e), 46.6 (e), 43.0 (e), 24.4 (o), 21.5 (o); IR (neat): $\tilde{\nu}$ = 3382 (br), 3071 (w), 2967 (w), 2919 (w), 1642 (w), 1600 (w), 1497 (w), 1445 (m), 1374 (m), 1265 (w), 1148 (w), 10076 (w), 1027 (s), 981 (w), 908 (s), 764 (m), 732 (vs), 700 (s) cm^{-1}; HRMS (CI(NH_4)) calcd for ($C_{13}H_{18}O$ + NH_4): 208.1696; found: 208.1692.

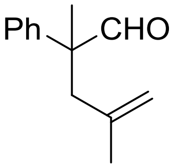

11

Compound 11. This compound was obtained from **11c** (0.53 mmol, 100 mg) following the same procedure as for the preparation of **1**. Colourless oil (90 mg, 91%). ^1H NMR (500 MHz, $CDCl_3$): δ = 9.54 (s, 1H), 7.40-7.35 (m, 2H), 7.31-7.27 (m, 3H), 4.82-4.79 (m, 1H), 4.63-4.61 (m, 1H), 2.72 (d, J = 13.9 Hz, 1H), 2.65 (d, J = 13.9 Hz, 1H), 1.47 (s, 3H), 1.40 (s, 3H); ^{13}C NMR (125 MHz, $CDCl_3$): δ = 201.9 (o), 141.5 (e), 139.9 (e), 128.8 (o, 2C), 127.3 (o, 2C), 115.5 (e), 53.5 (e), 44.3 (e), 24.2 (o), 18.6 (o); IR (neat): $\tilde{\nu}$ = 3074 (w), 3026 (w), 2970 (w), 2942 (w), 2805 (w), 2710 (w), 1721 (vs), 1644 (m), 1599 (w), 1582 (w), 1494 (m), 1446 (m), 1376 (m), 1320 (w), 1258 (w), 1159 (w), 1079 (w), 1030 (w), 983 (w), 895 (m), 852 (w), 804 (w), 760 (m), 699 (vs) cm^{-1}; elemental analysis (%) calcd for $C_{13}H_{16}O$: C 82.94, H 8.57; found: C 82.49, H 8.69.

---

[12] **15** is a known compound, see: C. Aïssa, K. Y-T. Ho, D. J. Tetlow, M. Pin-Nó, *Angew. Chem. Int. Ed* **2014**, 53, 4209

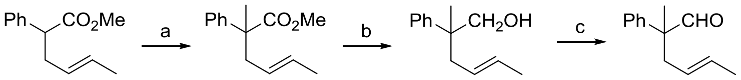

13a **13b** **13**

[(a)] i) NaH, DMF; ii) MeI, 76%. [(b)] $LiAlH_4$, Et_2O, quantitative. [(c)] $(COCl)_2$, DMSO, Et_3N, CH_2Cl_2, 83%.

Compound 13a. Under a N_2 atmosphere, NaH (0.81 mmol, 32 mg, 60% in oil) was suspended in DMF (8 mL) and the mixture was cooled to 0 °C. (*E*)-methyl 2-phenylhex-4-enoate (0.81 mmol, 166 mg) was added and the mixture was stirred for 15 minutes before adding methyl iodide (0.81 mmol, 51 µL) at 0 °C. The reaction was stirred for 16 hours, before quenching with a saturated aqueous solution of ammonium chloride, extracting with Et_2O and washing the organic layer several times with water. The organic layer was dried over $MgSO_4$, filtered and concentrated. Purification by flash column chromatography (petroleum ether/Et_2O = 90:1) afforded **13a** as a colourless oil (134 mg, 76%). ^{1}H NMR (500 MHz, $CDCl_3$): δ = 7.35-7.28 (m, 4H), 7.26-7.21 (m, 1H), 5.48 (dqt, *J* = 15.1, 6.5, 1.2 Hz, 1H), 5.28-5.20 (m, 1H), 3.65 (s, 3H), 2.77 (dd, *J* = 13.7, 7.3 Hz, 1H), 2.60–2.52 (m, 1H), 1.65–1.60 (m, 3H), 1.51 (s, 3H); ^{13}C NMR (125 MHz, $CDCl_3$): δ = 176.3 (e), 143.6 (e), 128.9 (o), 128.3 (o, 2C), 126.6 (o), 126.2 (o), 125.9 (o, 2C), 51.9 (o), 50.2 (e), 42.4 (e), 22.7 (o), 17.9 (o); IR (neat): $\tilde{\nu}$ = 3026 (w), 2984 (w), 2949 (w), 2856 (w), 1729 (vs), 1600 (w), 1583 (w), 1497 (m), 1446 (m), 1434 (m), 1377 (m), 1264 (m), 1223 (m), 1202 (m), 1135 (m), 1102 (m), 1072 (m), 1030 (m), 968 (m), 940 (w), 924 (w), 857 (w), 794 (w), 768 (m), 734 (m), 697 (vs) cm^{-1}; HRMS (CI(NH_4)) calcd for ($C_{14}H_{18}O_2$ + H): 219.1380; found: 219.1385.

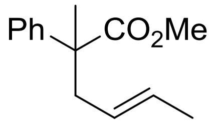

13a

Compound 13b. This compound was obtained from **13a** (0.61 mmol, 133 mg) following the same procedure as for the preparation of **1b**. Colourless oil (118 mg, quantitative). ^{1}H NMR (500 MHz, $CDCl_3$): δ = 7.39-7.32 (m, 4H), 7.25-7.20 (m, 1H), 5.46 (dqt, *J* = 15.2, 6.3, 1.3 Hz, 1H), 5.27-5.19 (m, 1H), 3.74 (dd, *J* = 11.0, 5.1 Hz, 1H), 3.60 (dd, *J* = 11.0, 7.0 Hz, 1H), 2.50–2.43 (m, 1H), 2.34–2.27 (m, 1H), 1.68–1.58 (m, 3H), 1.31 (s, 3H), 1.20 (t, *J* = 6.3 Hz, 1H (OH)); ^{13}C NMR (125 MHz, $CDCl_3$): δ = 145.0 (e), 128.3 (o, 2C), 127.9 (o), 126.7 (o), 126.6 (o, 2C), 126.0 (o), 71.6 (e), 43.2 (e), 41.6 (e), 21.8 (o), 17.9 (o); IR (neat): $\tilde{\nu}$ = 3374 (br), 3089 (w), 3058 (w), 3024 (w), 2964 (w), 2916 (m), 2879 (w), 1601 (w), 1497 (m), 1444 (m), 1376 (m), 1156 (w), 1024 (s), 967 (s), 913 (w), 842 (w), 758 (m), 700 (vs) cm^{-1}; HRMS (CI(NH_4)) calcd for ($C_{13}H_{18}O$ + NH_4): 208.1696; found: 208.1706.

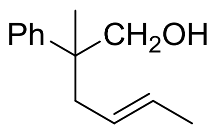

13b

Compound 13. This compound was obtained from **11c** (0.61 mmol, 115 mg) following the same procedure as for the preparation of **1**. Colourless oil (94 mg, 83%). ^{1}H NMR (500 MHz, $CDCl_3$): δ = 9.52 (s, 1H), 7.41-7.35 (m, 2H), 7.31-7.23 (m, 3H), 5.47 (dqt, *J* = 15.2, 6.3, 1.2 Hz, 1H), 5.23-5.15 (m, 1H), 2.62-2.58 (m, 2H), 1.62–1.58 (m, 3H), 1.41 (s, 3H); ^{13}C NMR (125 MHz, $CDCl_3$): δ = 202.4 (o), 139.9 (e), 129.2 (o), 128.8 (o, 2C), 127.2 (o), 127.1 (o, 2C), 125.3 (o), 53.8 (e), 39.3 (e), 19.0 (o), 17.9 (o); IR (neat): $\tilde{\nu}$ = 3059 (w), 3026 (w), 29877 (w), 2918 (w), 2855 (w), 2805 (w), 2706 (w), 1722 (vs), 1600 (w), 1582 (w), 1495 (m), 1445 (m), 1389 (w), 1372 (w), 1332 (w), 1261 (w), 1158 (w), 1076 (w), 1029 (m), 967 (s), 917 (m), 861 (w), 836 (w), 759 (m), 725 (w), 698 (vs), 656 (w) cm^{-1}; HRMS (CI(NH_4)) calcd for ($C_{13}H_{16}O$ + NH_4): 206.1539; found: 206.1535.

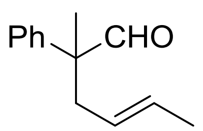

13

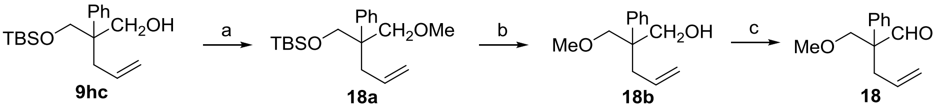

[(a)] i) NaH, THF; ii) MeI, 99%. [(b)] HCl, MeOH, 81%. [(c)] $(COCl)_2$, DMSO, Et_3N, CH_2Cl_2, 85%.

Compound 18a. Under a N_2 atmosphere, sodium hydride (1.17 mmol, 47 mg, 60% in oil) was suspended in THF (5 mL) and cooled to 0 °C. **9hc** (0.98 mmol, 300 mg) was added *via* syringe and was stirred to room temperature for 15 minutes. Methyl iodide (1.17 mmol, 73 μL) was added and the reaction was stirred at room temperature for 17 hours. The reaction was quenched with a saturated aqueous solution of ammonium chloride, extracted with Et_2O, and the organic layer was washed several times with water, then dried over $MgSO_4$, filtered and concentrated. Purification by flash column chromatography (petroleum ether/Et_2O = 98:1) afforded **18a** as a colourless oil (310 mg, 99%); ^{1}H NMR (500 MHz, $CDCl_3$): δ = 7.34-7.27 (m, 4H), 7.21-7.16 (m, 1H), 5.51 (ddt, *J* = 17.2, 10.0, 7.2 Hz, 1H), 5.02-4.97 (m, 1H), 4.94-4.90 (m, 1H), 3.83 (d, *J* = 9.4 Hz, 1H), 3.77 (d, *J* = 9.4 Hz, 1H), 3.64 (d, *J* = 9.0 Hz, 1H), 3.59 (d, *J* = 9.0 Hz, 1H), 3.32 (s, 3H), 2.53-2.43 (m, 2H), 0.87 (s, 9H), 0.00 (s, 3H), –0.02 (s, 3H); ^{13}C NMR (125 MHz, $CDCl_3$): δ = 142.9 (e), 134.4 (o), 127.9 (o, 2C), 126.9 (o, 2C), 126.0 (o), 117.2 (e), 75.1 (e), 65.6 (e), 59.1 (o), 46.9 (e), 38.6 (e), 25.8 (o, 3C), 18.2 (e), –5.7 (o, 2C); IR (neat): $\tilde{\nu}$ = 3061 (w), 2953 (w), 2927 (m), 2887 (w), 2856 (m), 1639 (w), 1601 (w), 1498 (w), 1471 (w), 1462 (w), 1446 (w), 1388 (w), 1360 (w), 1308 (w), 1252 (m), 1195 (w), 1093 (s), 1028 (w), 1005 (w), 975 (w), 938 (w), 913 (m), 834 (vs), 814 (w), 774 (s), 734 (w), 696 (s), 671 (m) cm^{-1}; HRMS (CI(NH_4)) calcd for ($C_{19}H_{32}O_2Si$ + H): 321.2244; found: 321.2251.

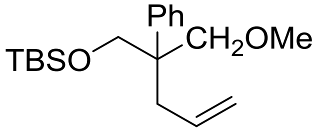

18a

Compound 18b. A solution made of concentrated hydrochloric acid (100 μL) in methanol (5mL) and **18a** (1.06 mmol, 340 mg) was stirred at room temperature for 2 hours. The mixture was quenched with water and extracted with Et_2O. The organic layer was dried over $MgSO_4$, filtered and concentrated. Purification by flash column chromatography (pentane/Et_2O = 4:1) afforded **18b** as a colourless oil (177 mg, 81%); ^{1}H NMR (500 MHz, $CDCl_3$): δ = 7.38-7.32 (m, 4H), 7.26-7.21 (m, 1H), 5.49 (ddt, *J* = 17.2, 10.0, 7.2 Hz, 1H), 5.06-5.00 (m, 1H), 4.98-4.94 (m, 1H), 3.98 (dd, *J* = 11.1, 6.0 Hz, 1H), 3.88 (dd, *J* = 11.2, 6.5 Hz, 1H), 3.85 (d, *J* = 9.2 Hz, 1H), 3.67 (d, *J* = 9.2 Hz, 1H), 3.40 (s, 3H), 2.53 (t, *J* = 6.3 Hz, 1H (OH)), 2.50 (dt, *J* = 7.3, 1.1 Hz, 2H); ^{13}C NMR (125 MHz, $CDCl_3$): δ = 141.6 (e), 133.7 (o), 128.3 (o, 2C), 126.7 (o, 2C), 126.3 (o), 117.6 (e), 77.8 (e), 68.6 (e), 59.2 (o), 46.4 (e), 39.0 (e); IR (neat): $\tilde{\nu}$ = 3435 (br), 3061 (w), 2978 (w), 2924 (w), 2890 (w), 1638 (w), 1600 (w), 1581 (w), 1498 (w), 1478 (w), 1446 (m), 1415 (w), 1385 (w), 1297 (w), 1196 (m), 1157 (w), 1102 (s), 1037 (m), 1020 (m), 998 (m), 968 (w), 912 (s), 766 (m), 732 (m), 697 (vs), 674 (w) cm^{-1}; HRMS (CI(NH_4)) calcd for ($C_{13}H_{18}O_2$ + H): 207.1380; found: 207.1381.

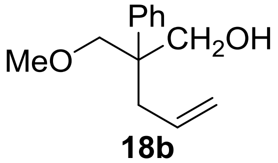

18b

Compound 18. This compound was obtained from **18b** (0.85 mmol, 175 mg) following the same procedure as for the preparation of **1**. Colourless oil (148 mg, 85%). ^{1}H NMR (500 MHz, $CDCl_3$): δ = 9.59 (s, 1H), 7.40-7.35 (m, 2H), 7.32-7.28 (m, 1H), 7.20-7.16 (m, 2H), 5.51 (ddt, *J* = 17.1, 10.0, 7.3 Hz, 1H), 5.10-5.01 (m, 2H), 3.98 (d, *J* = 9.3 Hz, 1H), 3.80 (d, *J* = 9.3 Hz, 1H), 3.36 (s, 3H), 2.79-2.71 (m, 2H); ^{13}C NMR (125 MHz, $CDCl_3$): δ = 201.0 (o), 136.4 (e), 132.9 (o), 128.8 (o, 2C), 127.5 (o), 127.4 (o, 2C), 118.7 (e), 72.9 (e), 59.5 (o), 57.9 (e), 36.0 (e); IR (neat): $\tilde{\nu}$ = 3076 (w), 2980 (w), 2925 (w), 2894 (w), 2822 (w), 1726 (vs), 1640 (w), 1599 (w), 1496 (m), 1448 (m), 1379 (w), 1193 (w), 1110 (vs), 1027 (m), 998 (m), 972 (m), 919 (m), 762 (m), 699 (vs) cm^{-1}; HRMS (CI(NH_4)) calcd for ($C_{13}H_{18}O_2$ + H): 205.1223; found: 205.1225.

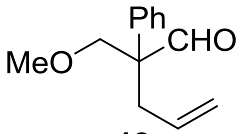

18

Preparation of compound *cis*-10a

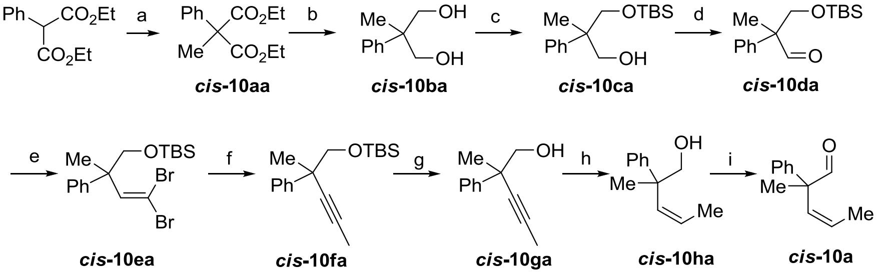

[(a)] i) NaH, THF; ii) MeI, 60%. [(b)] $LiAlH_4$, Et_2O, 0 °C, 68%. [(c)] TBSCl, imidazole, DMF, 79%. [(d)] $(COCl)_2$, DMSO, Et_3N, −78 °C. 94%. [(e)] PPh_3, CBr_4, CH_2Cl_2, 88%. [(f)] i) nBuLi, ii) MeI, THF, 94%. [(g)] nBu_4NF (1M inTHF), 51%. [(h)] $Pd/BaSO_4$, H_2, pyridine, 83%. [(i)] $(COCl)_2$, DMSO, Et_3N, −78 °C, .

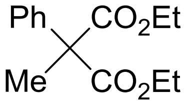

***cis*-10aa**

Compound *cis*-10aa. Under a N_2 atmosphere, NaH (16.93 mmol, 677 mg, 60% I oil) was suspended in THF (42 mL) and cooled to 0 °C. Diethyl 2-phenylmalonate (8.47 mmol, 2.0 g) was added *via* syringe and stirred for 30 minutes at 0 °C. Methyl iodide (16.93 mmol, 1 mL) was the added and the mixture was stirred for 48 hours at room temperature. The reaction was quenched with a saturated aqueous solution of ammonium chloride, extracted with Et_2O, and the combined organic layers were dried over $MgSO_4$, filtered and concentrated. Purification by flash column chromatography (petroleum ether/Et_2O = 30:1) afforded ***cis*-10aa** as a colourless oil (1.27 g, 60%). ^{1}H NMR (500 MHz, $CDCl_3$): δ = 7.39-7.31 (m, 4H), 7.29 (m, 1H), 4.29–4.19 (m, 4H), 1.86 (s, 3H), 1.25 (t, *J* = 7.2 Hz, 6H), in agreement with the previously reported data.[13]

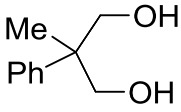

***cis*-10ba**

Compound *cis*-10ba. This compound was obtained from ***cis*-10aa** (6.52 mmol, 1.62 g) following the same procedure as for the preparation of **1b**, except that 2.5 equiv. of $LiAlH_4$ (16.29 mmol, 619 mg) was used. White solid (731 mg, 68%); ^{1}H NMR (500 MHz, $CDCl_3$): δ = 7.45-7.41 (m, 2H), 7.40-7.35 (m, 2H), 7.29-7.24 (m, 1H), 3.97 (dd, *J* = 11.0, 6.0 Hz, 2H), 3.84 (dd, *J* = 11.0, 5.9 Hz, 2H), 2.09 (t, *J* = 6.0 Hz, 2H), 1.31 (s, 3H), in agreement with the previously reported data.[14]

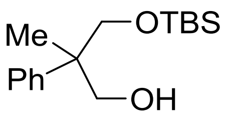

***cis*-10ca**

Compound *cis*-10ca. Imidazole (1.75 mmol, 119mg) was dissolved in DMF (22 mL) under N_2 atmosphere. Then, ***cis*-10ba** (1.75 mmol, 290 mg) was added in one portion and the mixture was stirred for 15 minutes, before adding *tert*-butyldimethylsilyl chloride (1.75 mmol, 264 mg) in one portion and the mixture was stirred until complete by TLC. The rmixture was quenched with a saturated aqueous solution of ammonium chloride, extracted with Et_2O (2 × 20 mL), and the combined organic layers were washed with water (2 × 40 mL) before drying over $MgSO_4$, filtration and concentration. Purification by flash column chromatography (petroleum ether/Et_2O = 9/1) afforded ***cis*-10ca** as a colourless oil (386 mg, 79%). ^{1}H NMR (500 MHz, $CDCl_3$): δ = 7.43-7.38 (m, 2H), 7.37-7.31 (m, 2H), 7.26-7.21 (m, 1H), 4.02-3.96 (m, 2H), 3.70 (dd, *J* = 10.9, 6.5 Hz, 1H), 3.70 (d, *J* = 9.8 Hz, 1H), 2.57 (t, *J* = 6.1 Hz, 1H), 1.34 (s, 3H), 0.88 (s, 9H), 0.05 (s, 3H), 0.03 (s, 3H); ^{13}C NMR (125 MHz, $CDCl_3$): δ = 143.6 (e), 128.1 (o, 2C), 126.5 (o, 2C), 126.3 (o), 70.3 (e), 69.9 (e), 44.2 (e), 25.7 (o, 3C), 20.1 (o), 18.0 (e), −5.8 (o, 2C); IR (neat): $\tilde{\nu}$ = 3428 (br), 3060 (w), 2955 (m), 2929 (m), 2884 (w), 2857 (m), 1602 (w), 1498 (w), 1472 (m), 1464 (m), 1445 (w), 1389 (w), 1362 (w), 1253 (m), 1097 (m), 1043 (m) 1028 (m), 1006 (m), 964 (w), 939 (w), 910 (w), 833 (vs), 814 (m), 774 (s), 759 (m), 734 (w), 697 (s), 666 (m) cm^{-1}; HRMS (CI(NH_4)) calcd for ($C_{16}H_{28}O_2Si$ + H): 281.1931; found: 281.1937.

---

[13] H. Shimakoshi, M. Abiru, S.-i. Izumi, Y. Hisaeda, *Chem. Commun.* **2009**, 6427
[14] C. E. Katz, J. Aube, *J. Am. Chem. Soc.* **2003**, *125*, 13948

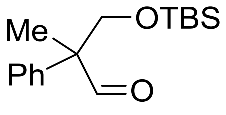

***cis*-10da**

Compound *cis*-10da. This compound was obtained from ***cis*-10ca** (1.77 mmol, 497 mg) following the same procedure as for the preparation of **1**. Colourless oil (463 mg, 94%). ^{1}H NMR (500 MHz, $CDCl_3$): δ = 9.66 (s, 1H), 7.39-7.34 (m, 2H), 7.31-7.23 (m, 3H), 4.19 (d, *J* = 9.9 Hz, 1H), 3.84 (d, *J* = 9.9 Hz, 1H), 1.49 (s, 3H), 0.84 (s, 9H), 0.01 (s, 3H), −0.02 (s, 3H); ^{13}C NMR (125 MHz, $CDCl_3$): δ = 201.9 (o), 138.5 (e), 128.5 (o, 2C), 127.2 (o), 127.0 (o, 2C), 67.2 (e), 55.7 (e), 25.6 (o, 3C), 18.0 (e), 17.4 (o), −5.8 (o), −5.9 (o); IR (neat): $\tilde{\nu}$ = 3063 (w), 2955 (w), 2929 (w), 2886 (w), 2857 (w), 2711 (w), 1726 (m), 1601 (w), 1496 (w), 1472 (w), 1463 (w), 1446 (w), 1390 (w), 1362 (w), 1252 (m), 1187 (w), 1100 (s), 1075 (m), 1030 (w), 1006 (w), 964 (w), 939 (w), 917 (w), 832 (vs), 815 (m), 775 (s), 756 (m), 737 (w), 697 (s), 671 (m) cm^{-1}; HRMS (CI(NH_4)) calcd for ($C_{16}H_{26}O_2Si$ + H): 279.1775; found: 279.1769.

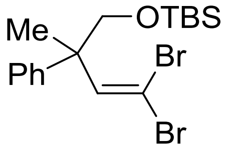

***cis*-10ea**

Compound *cis*-10ea. Under N_2 atmosphere, triphenyl phosphine (8.71 mmol, 2.28 g), carbon tetrabromide (4.35 mmol, 1.44 g) and ***cis*-10da** (2.18 mmol, 605 mg) were dissolved in CH_2Cl_2 (4.4 mL) and stirred for 16 hours. The reaction was concentrated, and the residue was triturated using pentane. The filtrate was concentrated to afford an oil which was purified by flash column chromatography (pentane only) to afford ***cis*-10ea** as a colourless oil (835 mg, 88%). ^{1}H NMR (500 MHz, $CDCl_3$): δ = 7.34-7.28 (m, 4H), 7.25-7.20 (m, 1H), 7.10 (s, 1H), 3.61 (d, *J* = 9.5 Hz, 1H), 3.43 (d, *J* = 9.5 Hz, 1H), 1.59 (s, 3H), 0.87 (s, 9H), −0.04 (s, 3H), −0.05 (s, 3H); ^{13}C NMR (125 MHz, $CDCl_3$): δ = 143.5 (o), 143.0 (e), 128.1 (o, 2C), 127.3 (o, 2C), 126.3 (o), 89.3 (e), 72.7 (e), 49.3 (e), 25.8 (o, 3C), 20.2 (o), 18.2 (e), −5.6 (o, 2C); IR (neat): $\tilde{\nu}$ = 3025 (w), 2953 (m), 2928 (m), 2896 (w), 2855 (m), 1603 (w), 1494 (w), 1470 (w), 1462 (w), 1445 (w), 1405 (w), 1387 (w), 1361 (w), 1283 (w), 1251 (m), 1230 (w), 1141 (w), 1095 (s), 1068 (s), 1029 (m), 1005 (m), 968 (m), 938 (w), 914 (w), 901 (w), 833 (vs), 774 (vs), 756 (m), 720 (w), 696 (s), 668 (m) cm^{-1}; HRMS (CI(NH_4)) calcd for ($C_{17}H_{26}{}^{79}Br_2OSi$+ H): 433.0192; found: 433.0185.

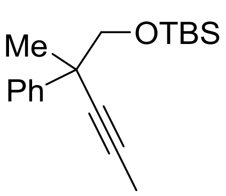

***cis*-10fa**

Compound *cis*-10fa. Under N_2 atmosphere, ***cis*-10ea** (1.87 mmol, 810 mg) was dissolved in THF (18 mL) and the mixture was cooled to −78 °C. *n*Butyllithium (4.67 mmol, 1.87 mL, 2.5 M in hexanes) was added and the mixture was stirred for 1 hour at room temperature. The reaction was cooled to −78 °C, before adding methyl iodide (4.67 mmol, 290 µL) and the mixture was stirred at room temperature for 16 hours. The reaction was quenched with saturated aqueous solution of ammonium chloride, extracted with Et_2O and the organic layer was then dried over $MgSO_4$, filtered and concentrated. Purification by flash column chromatography (pentane/Et_2O = 99/1) afforded ***cis*-10fa** as a colourless oil (503 mg, 94%); ^{1}H NMR (500 MHz, $CDCl_3$): δ = 7.58-7.52 (m, 2H), 7.33-7.27 (m, 2H), 7.24-7.18 (m, 1H), 3.67 (d, *J* = 9.4 Hz, 1H), 3.62 (d, *J* = 9.4 Hz, 1H), 1.88 (s, 3H), 1.57 (s, 3H), 0.84 (s, 9H), −0.06 (s, 3H), −0.10 (s, 3H); ^{13}C NMR (125 MHz, $CDCl_3$): δ = 143.9 (e), 127.8 (o, 2C), 127.0 (o, 2C), 126.4 (o), 83.5 (e), 78.5 (e), 72.4 (e), 42.3 (e), 25.8 (o, 3C), 24.6 (o), 18.3 (e), 3.6 (o), −5.6 (o, 2C); IR (neat): $\tilde{\nu}$ = 3026 (w), 2954 (m), 2928 (m), 2856 (m), 1602 (w), 1496 (w), 1471 (m), 1463 (m), 1446 (w), 1381 (w), 1361 (m), 1291 (w), 1253 (m), 1191 (w), 1136 (m), 1100 (s), 1029 (m), 1006 (m), 955 (w), 939 (w), 855 (m), 834 (vs), 809 (m), 774 (vs), 760 (s), 697 (vs), 666 (m); HRMS (ESI) calcd for ($C_{18}H_{28}OSi$ + Na): 311.1807; found: 311.1805; elemental analysis (%) calcd for $C_{18}H_{28}OSi$: C 74.94, H 9.78; found: C 74.93, H 9.80.

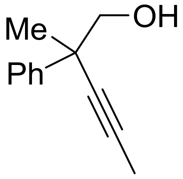

***cis*-10ga**

Compound *cis*-10ga. Under N_2 atmosphere, ***cis*-10fa** (0.694 mmol, 200 mg) was dissolved in THF (3.5 mL) and tetrabutylammonium fluoride (3.47 mmol, 3.5 mL, 1M solution in THF) was added and the mixture was stirred for 1 hour. The reaction was quenched with brine, extracted with Et_2O, and the organic layer was dried over $MgSO_4$, filtered and concentrated. Purification by flash column chromatography (petroleum ether/Et_2O = 5/1) afforded ***cis*-10ga** as a colourless oil (71 mg, 51%); ^{1}H NMR (500 MHz,

$CDCl_3$): δ = 7.56-7.51 (m, 2H), 7.38-7.32 (m, 2H), 7.28-7.23 (m, 1H), 3.68 (dd, *J* = 10.5, 6.4 Hz, 1H), 3.64 (dd, *J* = 10.5, 7.9 Hz, 1H), 1.93 (s, 3H), 1.79 (dd, *J* = 7.8, 6.4 Hz, 1H), 1.57 (s, 3H); ^{13}C NMR (125 MHz, $CDCl_3$): δ = 142.9 (e), 128.3 (o, 2C), 126.8 (o), 126.4 (o, 2C), 82.1 (e), 80.3 (e), 71.9 (e), 43.2 (e), 25.4 (o), 3.6 (o); IR (neat): $\tilde{\nu}$ = 3414 (br), 3059 (w), 2974 (m), 2919 (m), 2872 (m), 1601 (w), 1494 (m), 1445 (m), 1382 (w), 1268 (w), 1048 (s), 1026 (s), 943 (w), 760 (s), 680 (vs) cm^{-1}; HRMS (CI(NH_4)) calcd for ($C_{12}H_{14}O + NH_4$): 192.1383; found: 192.1386.

Compound *cis*-10ha. Under a N_2 atmosphere, ***cis*-10ga** (0.402 mmol, 70 mg), Pd/$BaSO_4$ (47 mg, 5% Pd), and pyridine (1.5 mL) were added together in a round bottom flask. The inert atmosphere was replaced with hydrogen by purging the reaction vessel three times. The reaction mixture stirred under a hydrogen atmosphere (balloon) for 16 hours. The crude reaction mixture was then absorbed on silica, and the plug of silica was then washed with Et_2O during filtration. After concentration of the filtrate, ***cis*-10ha** was obtained as a colourless oil (59 mg, 83%). ^{1}H NMR (500 MHz, $CDCl_3$): δ = 7.42-7.37 (m, 2H), 7.36-7.29 (m, 2H), 7.24-7.19 (m, 1H), 5.68 (dq, *J* = 11.5, 1.5 Hz, 1H), 5.60 (dq, *J* = 11.5, 7.0 Hz, 1H), 3.66 (m, 2H), 1.50 (s, 3H), 1.31 (t, *J* = 6.8 Hz, 1H), 1.23 (dd, *J* = 7.0, 1.6 Hz, 3H); ^{13}C NMR (125 MHz, $CDCl_3$): δ = 145.6 (e), 135.6 (o), 128.3 (o, 2C), 127.1 (o), 127.0 (o, 2C), 126.0 (o), 72.6 (e), 45.6 (e), 23.4 (o), 14.8 (o); IR (neat): $\tilde{\nu}$ = 3373 (br), 3057 (w), 3017 (w), 2965 (w), 2932 (w), 2872 (w), 1650 (w), 1600 (w), 1493 (m), 1444 (m), 1379 (w), 1255 (w), 1155 (w), 1036 (m), 1024 (m), 967 (w), 955 (w), 939 (w), 761 (m), 720 (w), 697 (vs), 671 (w), 654 (w) cm^{-1}; HRMS (CI(NH_4)) calcd for ($C_{12}H_{16}O + NH_4$): 194.1539; found: 194.1541.

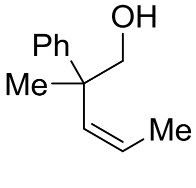

***cis*-10ha**

Compound *cis*-10a. This compound was obtained from ***cis*-10ha** (0.31 mmol, 55 mg) following the same procedure as for the preparation of **1**. Colourless oil (46 mg, 85%). %); ^{1}H NMR (500 MHz, $CDCl_3$): δ = 9.57 (s, 1H), 7.40-7.35 (m, 2H), 7.33-7.27 (m, 3H), 5.85-5.73 (m, 2H), 1.58 (s, 3H), 1.42 (dd, *J* = 6.8, 1.3 Hz, 3H); ^{13}C NMR (125 MHz, $CDCl_3$): δ = 198.9 (o), 140.8 (e), 130.9 (o), 129.5 (o), 128.8 (o, 2C), 127.4 (o, 2C), 127.1 (o), 56.3 (e), 22.1 (o), 15.1 (o); IR (neat): $\tilde{\nu}$ = 3022 (w), 2980 (w), 2931 (w), 2812 (w), 2711 (w), 1724 (vs), 1598 (w), 1492 (m), 1445 (m), 1386 (w), 1075 (w), 1028 (w), 949 (w), 902 (w), 763 (m), 699 (s), 677 (w) cm^{-1}; HRMS (CI(NH_4)) calcd for ($C_{12}H_{14}O + NH_4$): 192.1383; found: 192.1383.

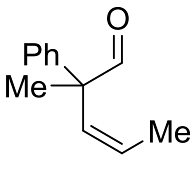

***cis*-10a**

Preparation of [Rh(nbd)(DTBM-Segphos)]BF_4{i.e. [Rh(nbd)(L1)]BF_4}

To a 2-neck round-bottomed flask containing $[Rh(nbd)_2]BF_4$ (143 mg, 0.38 mmol), *rac*-DTBM-SEGPHOS (410 mg, 0.35 mmol) and degassed CH_2Cl_2 (8.7 mL) were added under N_2, and the mixture was stirred for 1 hour at room temperature. The resultant red solution was concentrated, affording an orange precipitate that was collected by filtration, washed diethyl ether and dried in *vacuo* to give [Rh(nbd)(*rac*-DTBM-SEGPHOS]BF_4 (462 mg, 91 %) as pale orange solid.

The purity of each batch of this pre-catalyst was assessed by elemental analysis (%) calcd for $C_{81}H_{108}BF_4O_8P_2Rh_2$ (see below). All the batches perfomed equally well in the procedure described in this paper.

	Batch No 2	Batch No 2	Batch No 3	Batch No 4	Batch No 5	Batch No 6
calcd	found	found	found	found	found	found
C 66.57	C 65.43	C 65.34	C 63.22	C 63.46	C 63.38	C 65.39
H 7.45	H 7.54	H 7.45	H 7.25	H 7.18	H 7.18	H 7.27

Representative procedure for the rhodium-catalyzed isomerization – [Rh(nbd)(rac-DTBM-SEGPHOS)]BF_4 (0.0085, 12.4 mg) was added to a flame-dried J-Young Schlenk flask under N_2. Degassed acetone (1.7 mL) was added and the orange solution was hydrogenated by adding H_2 (1.64 mL) over 3 minutes *via* syringe. The resultant pale orange solution was sealed and stirred for 1 hour at room temperature. The solution was degassed by freeze-thaw method, before transferring *via* cannula to a second flame-dried J-Young Schlenk flask containing **1** (0.0847 mmol, 20 mg). The tube was sealed and the reaction was heated at 60 °C for 17 hours. The reaction mixture was cooled to room temperature, and the solvent was removed under reduced pressure. Purification by flash column chromatography (pentane/Et_2O, 98:1) afforded **2** as colourless oil (17.4 mg, 86%).

Compound	Isolated Yield (%)	Isolated Yield (%)	Average yield (%)
**10a**	77	75	**76**
**10b**	76	85	**81**
**10c**	93	91	**92**
**10d**	85	77	**81**
**10e**	80	85	**83**
**10f**	77	78	**78**
**10g**	98	97	**98**
**10h**	88	92	**90**
**10i**	63	-	**63**[a]
**10j**	78	82	**80**
**10k**	73	71	**72**
**10l**	75	80	**78**
**10m**	75	80	**78**
**10n**	83	85	**84**
**10o**	75	75	**75**

Table SI-1. Duplicate results from which the averages presented in Scheme 2 of the manuscript are derived.

Note: in the case of **10a**, **10b**, **10d**, **10j**, **10k**, **10l**, **10n** and **10o**, the yield of isolated product as indicated in table SI-1 were obtained after additional flash column chromatography on silica gel impregnated 10% silver nitrate (pentane/Et_2O = 99:1) to remove minor traces of starting material. Also in the case of **10k** and **10o**, the reaction was carried out at room temperature.

Analytical data for compounds 2, 5, 6, 8, and 10a–10o

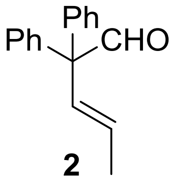

2

Compound 2. ^{1}H NMR (500 MHz, $CDCl_3$): δ = 9.88 (s, 1H), 7.39-7.27 (m, 6H), 7.17-7.11 (m, 4H), 6.21 (dq, *J* = 15.7, 1.6 Hz, 1H), 5.21 (dq, *J* = 15.7, 6.5 Hz, 1H), 1.81 (dd, *J* = 6.5, 1.7 Hz, 3H); ^{13}C NMR (125 MHz, $CDCl_3$): δ = 197.8 (o), 139.9 (e, 2C), 131.2 (o), 130.8 (o), 129.6 (o, 4C), 128.5 (o, 4C), 127.3 (o, 2C), 66.8 (e), 18.5 (o); IR (neat): $\tilde{\nu}$ = 3059 (w), 3030 (w), 2917 (w), 2854 (w), 2819 (w), 2721 (w), 1724 (s), 1597 (w), 1493 (m), 1446 (m), 1378 (w), 1186 (w), 1158 (w), 1119 (w), 1083 (w), 1034 (w), 1014 (w), 977 (m), 845 (w), 772 (w), 756 (m), 699 (vs), 659 (w) cm^{-1}; HRMS (CI(CH_4)) calcd for ($C_{17}H_{16}O$ + H): 237.1275; found: 237.1279; elemental analysis (%) calcd for $C_{17}H_{16}O$: C 86.40, H 6.82; found: C 85.88, H 6.97.

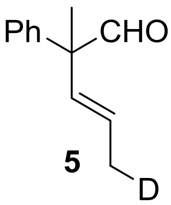

5

Compound 5. This compound was obtained from **3** (0.171 mmol, 30 mg) following the representative procedure described for the preparation of **2** with additional flash column chromatography on silica gel impregnated 10% silver nitrate (pentane/Et_2O = 99:1) to remove minor traces of starting material. Colourless oil (23.4 mg, 78%); ^{1}H NMR (500 MHz, $CDCl_3$): δ = 9.54 (s, 1H), 7.40-7.35 (m, 2H), 7.31-7.22 (m, 3H), 5.81 (dt, *J* = 15.8, 1.6 Hz, 1H), 5.63-5.55 (m, 1H), 1.81-1.77 (m, 2H), 1.51 (s, 3H); ^{13}C NMR (125 MHz, $CDCl_3$): δ = 199.6 (o), 140.9 (e), 131.1 (o), 128.8 (o, 2C), 128.5 (o), 127.4 (o, 2C), 127.2 (o), 57.1 (e), 20.9 (o), 18.2 (e, *J* = 19.36 Hz, t); IR (neat): $\tilde{\nu}$ = 3026 (w), 2980 (w), 2919 (w), 2810 (w), 2711 (w), 1722 (vs), 1599 (w), 1581 (w), 1492 (m), 1446 (m), 1424 (w), 1388 (w), 1369 (w), 1314 (w), 1270 (w), 1145 (w), 1076 (w), 1028 (m), 969 (m), 914 (w), 887 (w), 869 (w), 760 (s), 698 (vs) cm^{-1}; HRMS (CI(NH_4)) calcd for ($C_{12}H_{13}DO$ + NH_4): 193.1446; found: 193.1447.

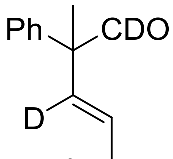

6

Compound 6. This compound was obtained from **4** (0.171 mmol, 30 mg) following the representative procedure described for the preparation of **2** with additional flash column chromatography on silica gel impregnated 10% silver nitrate (pentane/Et_2O = 99:1) to remove minor traces of starting material. Colourless oil (23.6 mg, 78%); ^{1}H NMR (500 MHz, $CDCl_3$): δ = 7.40-7.35 (m, 2H), 7.31-7.22 (m, 3H), 5.62-5.56 (m, 1H), 1.81 (d, *J* = 6.5 Hz, 3H), 1.51 (s, 3H); ^{13}C NMR (125 MHz, $CDCl_3$): δ = 199.3 (e, t, *J* = 27.3 Hz), 141.0 (e), 130.7 (e, t, *J* = 23.7 Hz), 128.8 (o, 2C), 128.5 (o), 127.4 (o, 2C), 127.2 (o), 56.9 (e), 20.8 (o), 18.4 (o); IR (neat): $\tilde{\nu}$ = 3059 (w), 3025 (w), 2979 (w), 2935 (w), 2855 (w), 2231 (w), 2107 (w), 2055 (w), 1711 (vs), 1599 (w), 1492 (m), 1446 (m), 1373 (m), 1157 (w), 1077 (w), 1048 (m), 1027 (w), 1011 (w), 895 (w), 796 (w), 759 (m), 699 (vs) cm^{-1}; HRMS (CI(NH_4)) calcd for ($C_{12}H_{12}D_2O$ + NH_4): 194.1508; found: 194.1513.

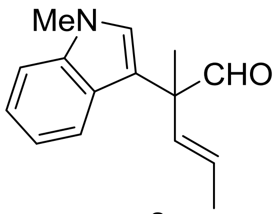

8

Compound 8. Colourless oil (18.6 mg, 93%); ^{1}H NMR (500 MHz, $CDCl_3$): δ = 9.54 (s, 1H), 7.54 (d, *J* = 8.1 Hz, 1H), 7.33 (d, *J* = 8.3 Hz, 1H), 7.27-7.22 (m, 1H), 7.12-7.07 (m, 1H), 6.96 (s, 1H), 5.95 (dq, *J* = 15.7, 1.6 Hz, 1H), 5.60 (dq, *J* = 15.7, 6.5 Hz, 1H), 3.79 (s, 3H), 1.79 (dd, *J* = 6.5, 1.6 Hz, 3H), 1.61 (s, 3H); ^{13}C NMR (125 MHz, $CDCl_3$): δ = 199.8 (o), 137.6 (e), 130.9 (o), 127.6 (o), 127.2 (o), 126.1 (e), 121.9 (o), 120.6 (o), 119.3 (o), 113.9 (e), 109.5 (o), 52.7 (e), 32.9 (o), 20.5 (o), 18.4 (o); IR (neat): $\tilde{\nu}$ = 3049 (w), 2965 (w), 2933 (w), 2880 (w), 2805 (w), 2703 (w), 1718 (s), 1615 (w), 1541 (w), 1483 (m), 1466 (m), 1449 (m), 1425 (w), 1373 (m), 1329 (m), 1251 (m), 1233 (w), 1150 (w), 1136 (w), 1101 (w), 1078 (w), 1049 (w), 1017 (w), 967 (m), 926 (w), 905 (w), 810 (w), 786 (w), 737 (vs), 674 (w) cm^{-1}; HRMS (CI(CH_4)) calcd for ($C_{15}H_{17}NO$ + H): 228.1383; found: 228.1378.

Compound 10a. Colourless oil. ^{1}H NMR (500 MHz, $CDCl_3$): δ = 9.54 (s, 1H), 7.40-7.35 (m, 2H), 7.31-7.22 (m, 3H), 5.81 (dq, *J* = 15.8, 1.6 Hz, 1H), 5.60 (dq, *J* = 15.8, 6.4 Hz, 1H), 1.81 (dd, *J* = 6.4, 1.7 Hz, 3H), 1.51 (s, 3H); ^{13}C NMR (125 MHz, $CDCl_3$): δ = 199.6 (o), 141.0 (e), 131.1 (o), 128.8 (o, 2C), 128.6 (o), 127.5 (o, 2C), 127.3 (o), 57.1 (e), 20.9 (o), 18.5 (o); IR (neat): $\tilde{\nu}$ = 3026 (w), 2979 (w), 2935 (w), 2856 (w), 2810 (w), 2712 (w), 1725 (vs), 1599 (w), 1582 (w), 1492 (m), 1446 (m), 1389 (w), 1149 (w), 1076 (w), 1028 (w), 970 (m), 917 (w), 839 (w), 786 (w), 760 (m), 700 (s) cm^{-1}; HRMS (CI(CH_4)) calcd for ($C_{12}H_{14}O$ + H): 175.1117; found: 175.1120; elemental analysis (%) calcd for $C_{12}H_{14}O$: C 82.72, H 8.10; found: C 82.71, H 8.44.

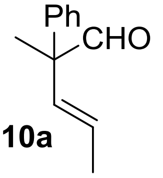

10a

Compound 10b. Colourless oil. ^{1}H NMR (500 MHz, $CDCl_3$): δ = 9.49 (s, 1H), 7.18-7.14 (m, 2H), 6.93-6.88 (m, 2H), 5.82-5.75 (m, 1H), 5.57 (dq, *J* = 15.8, 6.5 Hz, 1H), 3.80 (s, 3H), 1.80 (dd, *J* = 6.4, 1.6 Hz, 3H), 1.48 (s, 3H); ^{13}C NMR (125 MHz, $CDCl_3$): δ = 199.7 (o), 158.7 (e), 132.7 (e), 131.4 (o), 128.6 (o, 2C), 128.2 (o), 114.2 (o, 2C), 56.3 (e), 55.3 (o), 20.9 (o), 18.5 (o); IR (neat): $\tilde{\nu}$ = 3077 (w), 2978 (w), 2914 (w), 2708 (w), 1720 (vs), 1638 (m), 1607 (m), 1579 (m), 1510 (vs), 1461 (m), 1431 (w), 1416 (w), 1383 (w), 1296 (m), 1245 (vs), 1184 (vs), 1137 (w), 1118 (w), 1079 (w), 996 (s), 970 (s), 911 (vs), 826 (vs), 728 (m) cm^{-1}; HRMS (CI(NH_4)) calcd for ($C_{13}H_{16}O_2$ + H): 205.1223; found: 205.1231.

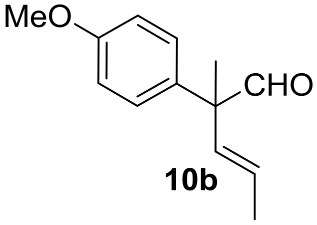

10b

Compound 10c. Colourless oil. ^{1}H NMR (500 MHz, $CDCl_3$): δ = 9.50 (s, 1H), 7.36-7.32 (m, 2H), 7.19-7.15 (m, 2H), 5.75 (dq, *J* = 15.7, 1.5 Hz, 1H), 5.59 (dq, *J* = 15.8, 6.4 Hz, 1H), 1.80 (dd, *J* = 6.4, 1.6 Hz, 3H), 1.49 (s, 3H); ^{13}C NMR (125 MHz, $CDCl_3$): δ = 199.1 (o), 139.4 (e), 133.3 (e), 130.6 (o), 129.1 (o), 128.93 (o, 2C), 128.91 (o, 2C), 56.7 (e), 20.9 (o), 18.5 (o); IR (neat): $\tilde{\nu}$ = 3028 (w), 2979 (w), 2936 (w), 2918 (w), 2812 (w), 2855 (w), 2712 (w), 1722 (vs), 1594 (w), 1492 (m), 1449 (w), 1402 (w), 1314 (w), 1186 (w), 1147 (w), 1096 (s), 1057 (w), 1013 (s), 970 (s), 916 (w), 824 (s), 785 (w), 757 (m), 720 (m), 680 (w) cm^{-1}; HRMS (CI(NH_4)) calcd for ($C_{12}H_{13}{}^{35}ClO$ + NH_4): 226.0993; found: 226.0991.

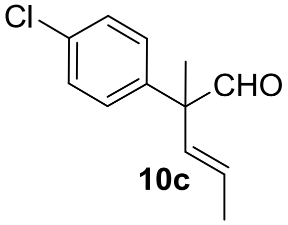

10c

Compound 10d. Colourless oil. ^{1}H NMR (500 MHz, $CDCl_3$): δ = 9.63 (s, 1H), 7.87-7.80 (m, 3H), 7.71-7.67 (m, 1H), 7.52-7.46 (m, 2H), 7.35 (dd, *J* = 8.6, 1.9 Hz, 1H), 5.91 (dq, *J* = 15.8, 1.6 Hz, 1H), 5.64 (dq, *J* = 15.8, 6.5 Hz, 1H), 1.85 (dd, *J* = 6.5, 1.7 Hz, 3H), 1.60 (s, 3H); ^{13}C NMR (125 MHz, $CDCl_3$): δ = 199.7 (o), 138.4 (e), 133.4 (e), 132.4 (e), 131.1 (o), 128.9 (o), 128.5 (o), 128.0 (o), 127.5 (o), 126.3 (o, 2C), 126.2 (o), 125.5 (o), 57.3 (e), 21.0 (o), 18.6 (o); IR (neat): $\tilde{\nu}$ = 3056 (w), 2978 (w), 2935 (w), 2811 (w), 2709 (w), 1725 (vs), 1632 (w), 1599 (w), 1506 (w), 1449 (w), 1377 (w), 1274 (w), 1130 (w), 1061 (w), 970 (m), 894 (w), 857 (m), 818 (m), 747 (m), 658 (w) cm^{-1}; HRMS (CI(NH_4)) calcd for ($C_{16}H_{16}O$ + H): 225.1235; found: 225.1280.

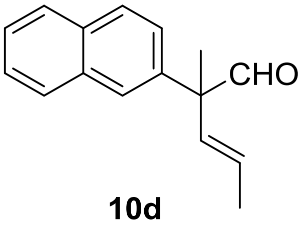

10d

Compound 10e. Colourless oil. ^{1}H NMR (500 MHz, $CDCl_3$): δ = 9.57 (s, 1H), 7.53 (s, 1H), 7.51–7.48 (m, 1H), 7.48–7.45 (m, 1H), 7.32-7.28 (m, 1H), 7.23-7.19 (m, 1H), 5.85 (dq, *J* = 15.8, 1.6 Hz, 1H), 5.63 (dq, *J* = 15.7, 6.5 Hz, 1H), 1.79 (dd, *J* = 6.5, 1.6 Hz, 3H), 1.61 (s, 3H); ^{13}C NMR (125 MHz, $CDCl_3$): δ = 198.9 (o), 155.9 (e), 142.4 (o), 129.3 (o), 129.1 (o), 126.0 (e), 124.5 (o), 122.6 (o), 121.3 (o), 120.7 (e), 111.8 (o), 51.9 (e), 20.1 (o), 18.3 (o); IR (neat): $\tilde{\nu}$ = 3031 (w), 2973 (w), 2937 (w), 2855 (w), 2810 (w), 2712 (w), 1726 (s), 1615 (w), 1568 (w), 1453 (m), 1379 (w), 1330 (w), 1281 (w), 1265 (w), 1207 (w), 1158 (w), 1110 (w), 1097 (m), 1017 (m), 968 (m), 931 (w), 907 (w), 858 (m), 810 (w), 786 (w), 768 (w), 745 (vs), 710 (w), 668 (w) cm^{-1}; HRMS (CI(NH_4)) calcd for ($C_{14}H_{14}O_2$ + H): 215.1067; found: 215.1065.

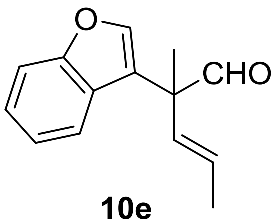

10e

Compound 10f. Colourless oil. ^{1}H NMR (500 MHz, $CDCl_3$): δ = 9.46 (s, 1H), 7.27-7.18 (m, 3H), 7.11-7.06 (m, 2H), 5.49 (dq, *J* = 16.6, 5.5 Hz, 1H), 5.45-5.41 (m, 1H), 2.95 (d, *J* = 13.5 Hz, 1H), 2.83 (d, *J* = 13.6 Hz, 1H), 1.73 (dd, *J* = 5.7, 1.0 Hz, 3H), 1.09 (s, 3H); ^{13}C NMR (125 MHz, $CDCl_3$): δ = 202.6 (o), 136.8 (e), 131.2 (o), 130.4 (o, 2C), 128.2 (o), 128.0 (o, 2C), 126.4 (o), 52.9 (e), 42.2 (e), 18.4 (o), 18.3 (o); IR (neat): $\tilde{\nu}$ = 3063 (w), 3029 (w), 2967 (w), 2929 (w), 2806 (w), 2705 (w), 1721 (s), 1604 (w), 1496 (m), 1453 (m), 1377 (w), 1320 (w), 1184 (w), 1068 (w), 1031 (w), 970 (m), 912 (w), 872 (w), 781 (m), 734 (m), 700 (vs) cm^{-1}; HRMS (CI(NH_4)) calcd for ($C_{13}H_{16}O + NH_4$): 206.1539; found: 206.1542.

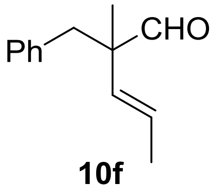

10f

Compound 10g. Colourless oil. ^{1}H NMR (500 MHz, $CDCl_3$): δ = 9.35 (s, 1H), 5.49 (dq, *J* = 15.8, 6.2 Hz, 1H), 5.39 (dq, *J* = 15.8, 1.4 Hz, 1H), 1.81–1.74 (m, 2H), 1.73 (dd, *J* = 6.2, 1.3 Hz, 3H), 1.71–1.62 (m, 3H), 1.52-1.45 (m, 1H), 1.30-1.19 (m, 2H), 1.12 (tt, *J* = 12.9, 3.5 Hz, 1H), 1.07–0.91 (m, 2H), 1.05 (s, 3H); ^{13}C NMR (125 MHz, $CDCl_3$): δ = 203.6 (o), 130.9 (o), 127.7 (o), 55.3 (e), 42.7 (o), 27.9 (e), 27.0 (e), 26.8 (e), 26.7 (e), 26.5 (e), 18.5 (o), 13.9 (o); IR (neat): $\tilde{\nu}$ = 2925 (s), 2853 (s), 2696 (w), 1725 (vs), 1449 (s), 1396 (w), 1376 (w), 1350 (w), 1322 (w), 1270 (w), 1203 (w), 1011 (w), 970 (s), 931 (w), 907 (m), 846 (w), 782 (w), 659 cm^{-1}; HRMS (CI(NH_4)) calcd for ($C_{12}H_{20}O$ + H): 181.1587; found: 181.1589.

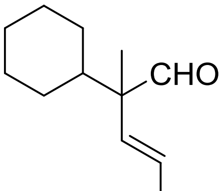

10g

Compound 10h. Colourless oil. ^{1}H NMR (500 MHz, $CDCl_3$): δ = 9.64 (s, 1H), 7.38-7.33 (m, 2H), 7.31-7.22 (m, 3H), 5.78 (dq, *J* = 16.0, 1.6 Hz, 1H), 5.50 (dq, *J* = 16.0, 6.5 Hz, 1H), 4.12 (d, *J* = 9.9 Hz, 1H), 4.08 (d, *J* = 9.9 Hz, 1H), 1.78 (dd, *J* = 6.5, 1.7 Hz, 3H), 0.81 (s, 9H), –0.04 (s, 3H), –0.06 (s, 3H); ^{13}C NMR (125 MHz, $CDCl_3$): δ = 200.2 (o), 138.1 (e), 130.4 (o), 128.6 (o, 2C), 128.4 (o, 2C), 127.3 (o), 66.4 (e), 62.5 (e), 25.7 (o, 3C), 18.7 (o), 18.1 (e), –5.75 (o), –5.78 (o); IR (neat): $\tilde{\nu}$ = 3027 (w), 2954 (m), 2928 (m), 2884 (w), 2856 (m), 2714 (w), 1724 (s), 1600 (w), 1494 (w), 1471 (w), 1463 (w), 1447 (w), 1389 (w), 1361 (w), 1253 (m), 1187 (w), 1105 (s), 1022 (w), 1006 (w), 971 (m), 938 (w), 836 (vs), 815 (w), 776 (s), 757 (m), 698 (s), 672 (w) cm^{-1}; HRMS (CI(NH_4)) calcd for ($C_{18}H_{28}O_2Si$ + H): 305.1931; found: 305.1940.

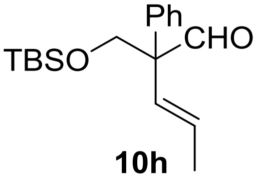

10h

Compound 10j. Colourless oil. ^{1}H NMR (500 MHz, $CDCl_3$): δ = 9.51 (s, 1H), 7.38-7.34 (m, 2H), 7.30-7.25 (m, 1H), 7.24-7.20 (m, 2H), 5.75 (dq, *J* = 16.1, 1.5 Hz, 1H), 5.52 (dq, *J* = 16.0, 6.4 Hz, 1H), 5.02-4.96 (m, 1H), 2.70 (d, *J* = 7.1 Hz, 2H), 1.80 (dd, *J* = 6.4, 1.5 Hz, 3H), 1.62 (s, 3H), 1.48 (s, 3H); ^{13}C NMR (125 MHz, $CDCl_3$): δ = 199.6 (o), 139.3 (e), 134.4 (e), 130.1 (o), 129.8 (o), 128.5 (o, 2C), 128.4 (o, 2C), 127.1 (o), 118.8 (o), 60.9 (e), 33.5 (e), 25.8 (o), 18.7 (o), 17.9 (o); IR (neat): $\tilde{\nu}$ = 3026 (w), 2967 (w), 2916 (w), 2856 (w), 2715 (w), 1722 (vs), 1599 (w), 1494 (m), 1447 (m), 1377 (m), 1108 (w), 1027 (w), 972 (m), 838 (w), 760 (m), 699 (vs) cm^{-1}; HRMS (CI(NH_4)) calcd for ($C_{16}H_{20}O$ + H): 229.1587; found: 229.1589.

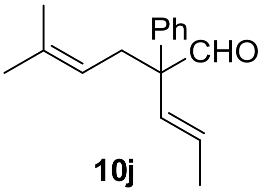

10j

Compound 10k. Colourless oil.^{1}H NMR (500 MHz, $CDCl_3$): δ = 9.47 (s, 1H), 7.40-7.34 (m, 2H), 7.31-7.25 (m, 1H), 7.24-7.19 (m, 2H), 5.79-5.68 (m, 2H), 5.55 (dq, *J* = 16.0, 6.4 Hz, 1H), 5.00-4.90 (m, 2H), 2.07-1.90 (m, 4H), 1.82 (dd, *J* = 6.4, 1.7 Hz, 3H), 1.30-1.14 (m, 2H); ^{13}C NMR (125 MHz, $CDCl_3$): δ = 199.1 (o), 139.4 (e), 138.4 (o), 130. 1 (o), 129.8 (o), 128.7 (o, 2C), 128.2 (o, 2C), 127.2 (o), 114.8 (e), 60.7 (e), 34.16 (e), 34.15 (e), 23.6 (e), 18.7 (o); IR (neat): $\tilde{\nu}$ = 3061 (w), 3025 (w), 2939 (m), 2856 (w), 2809 (w), 2715 (w), 1722 (vs), 1640 (w), 1599 (w), 1493 (m), 1446 (m), 1378 (w), 1309 (w), 1084 (w), 1032 (w), 973 (m), 910 (m), 838 (w), 759 (m), 700 (s) cm^{-1}; HRMS (CI(NH_4)) calcd for ($C_{16}H_{20}O$ + H): 229.1587; found: 229.1592.

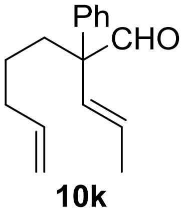

10k

Compound 10l. Colourless oil. ^{1}H NMR (500 MHz, $CDCl_3$): δ = 9.52 (s, 1H), 7.22-7.14 (m, 4H), 5.96 (ddt, *J* = 16.9, 10.1, 6.8 Hz, 1H), 5.80 (dq, *J* = 15.8, 1.6 Hz, 1H), 5.58 (dq, *J* = 15.8, 6.5 Hz, 1H), 5.12-5.06 (m, 2H), 3.38 (d, *J* = 6.8 Hz, 2H), 1.80 (dd, *J* = 6.5, 1.7 Hz, 3H), 1.49 (s, 3H); ^{13}C NMR (125 MHz, $CDCl_3$): δ = 199.7 (o), 139.1 (e), 138.6 (e), 137.1 (o), 131.2 (o), 129.0 (o, 2C), 128.4 (o), 127.5 (o, 2C), 116.0 (e), 56.8 (e), 39.7 (e), 20.8 (o), 18.5 (o); IR (neat): $\tilde{\nu}$ = 3079 (w), 3025 (w), 2978 (w), 2917 (w), 2854 (w), 2808 (w), 2710 (w), 1725 (vs), 1638 (m), 1510 (m), 1448 (w), 1435 (w), 1378 (w), 1297 (w), 1188 (w), 1148 (w), 1116 (w), 1088 (w), 1060 (w), 1038 (w), 1019 (m), 992 (w), 969 (m), 913 (s), 840 (m), 805 (m), 744 (w), 716 (w), 662 (w) cm^{-1}; HRMS (CI(NH_4)) calcd for ($C_{15}H_{18}O$ + H): 215.1430; found: 215.1439.

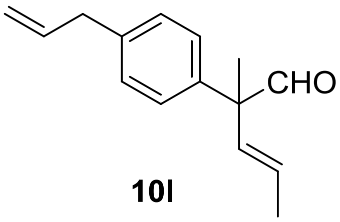

10l

Compound 10m. Colourless oil. ^{1}H NMR (500 MHz, $CDCl_3$): δ = 9.49 (s, 1H), 7.17-7.12 (m, 2H), 6.94-6.89 (m, 2H), 6.05 (ddt, *J* = 17.3, 10.6, 5.3 Hz, 1H), 5.78 (dq, *J* = 15.8, 1.6 Hz, 1H), 5.57 (dq, *J* = 15.8, 6.4 Hz, 1H), 5.41 (dq, *J* = 17.3, 1.6 Hz, 1H), 5.29 (dq, *J* = 10.5, 1.4 Hz, 1H), 4.53 (dt, *J* = 5.3, 1.5 Hz, 2H), 1.80 (dd, *J* = 6.5, 1.7 Hz, 3H), 1.48 (s, 3H); ^{13}C NMR (125 MHz, $CDCl_3$): δ = 199.7 (o), 157.8 (e), 133.2 (o), 132.8 (e), 131.4 (o), 128.6 (o, 2C), 128.2 (o), 117.7 (e), 115.0 (o, 2C), 68.8 (e), 56.3 (e), 20.9 (o), 18.5 (o); IR (neat): $\tilde{\nu}$ = 3025 (w), 2978 (w), 2934 (w), 2857 (w), 2809 (w), 2711 (w), 1723 (vs), 1649 (w), 1606 (m), 1579 (m), 1508 (vs), 1453 (m), 1426 (w), 1384 (w), 1367 (w), 1294 (m), 1246 (vs), 1228 (w), 1182 (s), 1154 (w), 1114 (w), 1060 (w), 1021 (s), 996 (m), 970 (s), 922 (s), 827 (vs), 727 (m), 661 (w) cm^{-1}; HRMS (CI(NH_4)) calcd for ($C_{15}H_{18}O_2$ + H): 231.1380; found: 231.1388.

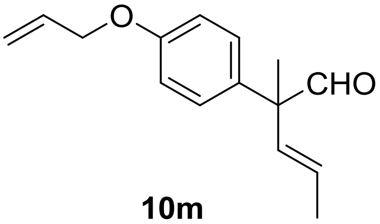

10m

Compound 10n. Colourless oil. ^{1}H NMR (500 MHz, $CDCl_3$): δ = 9.51 (s, 1H), 7.52-7.47 (m, 2H), 7.27-7.23 (m, 2H), 7.18-7.14 (m, 2H), 7.08-7.04 (m, 2H), 5.77 (dq, *J* = 15.9, 1.6 Hz, 1H), 5.73 (ddt, *J* = 16.9, 10.4, 6.5 Hz, 1H), 5.58 (dq, *J* = 15.8, 6.5 Hz, 1H), 5.11-5.03 (m, 2H), 4.17-4.13 (m, 2H), 2.43 (s, 3H), 1.80 (dd, *J* = 6.5, 1.6 Hz, 3H), 1.48 (s, 3H); ^{13}C NMR (125 MHz, $CDCl_3$): δ = 199.2 (o), 143.5 (e), 140.4 (e), 138.3 (e), 135.5 (e), 132.7 (o), 130.8 (o), 129.5 (o, 2C), 128.9 (o, 2C), 128.8 (o), 128.0 (o, 2C), 127.7 (o, 2C), 118.8 (e), 56.8 (e), 53.5 (e), 21.5 (o), 20.8 (o), 18.5 (o); IR (neat): $\tilde{\nu}$ = 3028 (w), 2973 (w), 2954 (w), 2926 (w), 2854 (w), 2807 (w), 2709 (w), 1722 (s), 1646 (m), 1504 (m), 1452 (w), 1433 (w), 1404 (w), 1382 (w), 1339 (s), 1305 (m), 1222 (m), 1162 (w), 1107 (vs), 1091 (w), 1060 (m), 1036 (w), 1018 (m), 1008 (w), 976 (m), 920 (m), 864 (m), 842 (w), 814 (m), 800 (w), 786 (w), 766 (m), 746 (w), 710 (m), 656 (s) cm^{-1}; HRMS (CI(NH_4)) calcd for ($C_{22}H_{25}NO_3S$ + H): 384.1628; found: 384.1639.

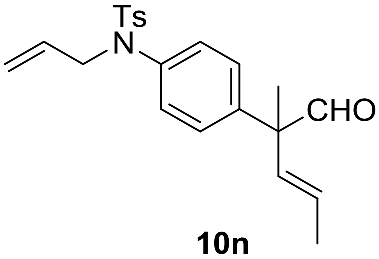

10n

Compound 10o. Colourless oil. ^{1}H NMR (500 MHz, $CDCl_3$): δ = 9.49 (s, 1H), 7.18-7.14 (m 2H), 7.00-6.96 (m, 2H), 5.83-5.75 (m, 2H), 5.57 (dq, *J* = 15.8, 6.5 Hz, 1H), 5.28 (ddt, *J* = 17.0, 1.8, 1.7 Hz, 1H), 5.10 (ddt, *J* = 10.0, 1.7, 1.6 Hz, 1H), 4.70 (t, *J* = 2.1 Hz, 2H), 3.03-3.00 (m, 2H), 1.80 (dd, *J* = 6.5, 1.6 Hz, 3H), 1.48 (s 3H); ^{13}C NMR (125 MHz, $CDCl_3$): δ = 199.6 (o), 157.0 (e), 133.4 (e), 131.9 (o), 131.4 (o), 128.6 (o, 2C), 128.2 (o), 116.4 (e), 115.2 (o, 2C), 84.7 (e), 77.1 (e), 56.4 (e, 2C), 23.1 (e), 20.9 (o), 18.5 (o); IR (neat): $\tilde{\nu}$ = 2979 (w), 2918 (w), 2812 (w), 2712 (w), 2252 (w), 1724 (s), 1642 (w), 1606 (m), 1580 (w), 1508 (vs), 1450 (m), 1417 (w), 1376 (m), 1296 (m), 1262 (w), 1222 (s), 1182 (s), 1138 (w), 1114 (w), 1050 (w), 1010 (s), 970 (m), 910 (s), 807 (w), 724 (vs) cm^{-1}; HRMS (CI(NH_4)) calcd for ($C_{18}H_{20}O_2$ + H): 269.1536; found: 269.1546.

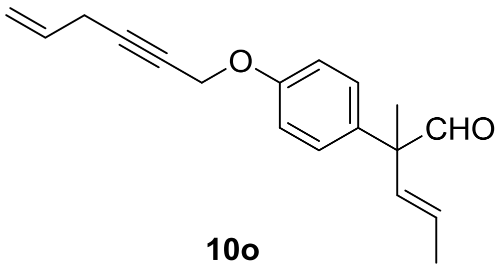

10o

Analytical data for compounds 12 and 14

Compound 12. This compound was obtained from **11** (0.106 mmol, 20 mg) following the representative procedure described for the preparation of **2**. Purification by flash column chromatography (pentane/Et_2O = 19:1) enabled isolation of **12** as colourless oil (4.5 mg, 23%), besides recovered **11** (13 mg, 67%). ^{1}H NMR (500 MHz, $CDCl_3$): δ = 7.40-7.36 (m, 2H), 7.35-7.30 (m, 2H), 7.24-7.20 (m, 1H), 2.63 (ddd, J = 18.0, 7.0, 2.3 Hz, 1H), 2.44-2.33 (m, 1H), 2.30 (ddd, J = 12.7, 6.1, 2.3 Hz, 1H), 2.04-1.96 (m, 2H), 1.42 (s, 3H), 1.18 (d, J = 6.4 Hz, 3H); ^{13}C NMR (125 MHz, $CDCl_3$): δ = 220.4 (e), 144.5 (e), 128.3 (o, 2C), 126.4 (o), 126.2 (o, 2C), 54.0 (e), 47.9 (e), 47.4 (e), 27.6 (o), 24.9 (o), 20.5 (o); IR (neat): $\tilde{\nu}$ = 3058 (w), 3025 (w), 2956 (m), 2926 (m), 2869 (w), 1735 (vs), 1601 (w), 1495 (m), 1457 (w), 1444 (m), 1405 (w), 1378 (w), 1369 (w), 1315 (w), 1298 (w), 1256 (m), 1225 (w), 1187 (w), 1147 (m), 1119 (w), 1076 (w), 1053 (m), 1029 (m), 932 (w), 912 (w), 839 (w), 760 (s), 698 (vs) cm^{-1}; HRMS (CI(NH_4)) calcd for ($C_{13}H_{16}O$ + NH_4): 206.1539; found: 206.1543.

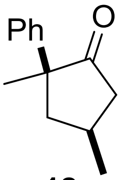

12

Compound 14. This compound was obtained from **13** (0.106 mmol, 20 mg) following the representative procedure described for the preparation of **2**, except that the active catalyst was prepared from [Rh(nbd)(**L1**)]BF_4 (0.0212 mmol, 31.1 mg) with H_2 (4.1 mL). ^{1}H NMR of the crude indicated a conversion of **13** to **14** of only 40%. Attempts to purify by flash column chromatography using 10% $AgNO_3$ impregnated silica (pentane/Et_2O = 90:1) increased the ratio of **13**/**14** to 1:2. Compound **14** could also be prepared independently by Julia-Kocienski olefination. ^{1}H NMR (500 MHz, $CDCl_3$): δ = 9.54 (s, 1H), 7.40-7.35 (m, 2H), 7.31-7.23 (m, 3H), 5.78 (dt, J = 15.9, 1.5 Hz, 1H), 5.63 (dt, J = 15.9, 6.3 Hz, 1H), 2.20-2.13 (m, 2H), 1.51 (s, 3H), 1.04 (t, J = 7.5 Hz, 3H); ^{13}C NMR (125 MHz, $CDCl_3$): δ = 199.6 (o), 141.1 (e), 135.5 (o), 128.9 (o), 128.8 (o, 2C), 127.5 (o, 2C), 127.2 (o), 56.9 (e), 26.1 (e), 21.0 (o), 13.6 (o); IR (neat): $\tilde{\nu}$ = 3059 (w), 3025 (w), 2965 (w), 2933 (w), 2873 (w), 2809 (w), 2710 (w), 1724 (s), 1599 (w), 1581 (w), 1492 (m), 1446 (m), 1388 (w), 1370 (w), 1334 (w), 1187 (w), 1156 (w), 1108 (w), 1076 (w), 1027 (m), 974 (m), 936 (w), 906 (w), 845 (w), 822 (w), 760 (s), 698 (vs) cm^{-1}; HRMS (CI(NH_4)) calcd for ($C_{13}H_{16}O$ + H): 189.1274; found: 189.1276.

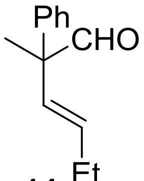

14

Preparation of compound 17 and isolation of transient intermediate 16

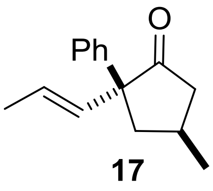

Compound 17. This compound was obtained from **15** (0.0933 mmol, 20 mg) following the representative procedure described for the preparation of **2**. Colourless oil (19.3 mg, 97%). ^{1}H NMR (500 MHz, $CDCl_3$): δ = 7.32-7.28 (m, 4H), 7.24-7.19 (m, 1H), 5.59 (dq, 15.7, 6.3 Hz, 1H), 5.51-5.46 (m, 1H), 2.63 (ddd, J = 18.5, 7.3, 2.5 Hz, 1H), 2.49 (ddd, J = 12.7, 5.7, 2.5 Hz, 1H), 2.42-2.30 (m, 1H), 1.97-1.89 (m, 2H), 1.74 (dd, J = 6.3, 1.4 Hz, 3H), 1.17 (d, J = 6.5 Hz, 3H); ^{13}C NMR (125 MHz, $CDCl_3$): δ = 217.3 (e), 142.9 (e), 132.1 (o), 128.2 (o, 2C), 127.2 (o, 2C), 127.1 (o), 126.5 (o), 61.7 (e), 47.0 (e), 46.1 (e), 27.5 (o), 20.2 (o), 18.1 (o); IR (neat): $\tilde{\nu}$ = 3059 (w), 3025 (w), 2955 (m), 2870 (w), 1739 (vs), 1600 (w), 1496 (m), 1447 (m), 1405 (w), 1378 (w), 1351 (w), 1319 (w), 1272 (w), 1237 (w), 1189 (w), 1146 (s), 1112 (w), 1079 (w), 1034 (w), 970 (s), 922 (w), 910 (w), 840 (w), 759 (s), 697 (vs) cm^{-1}; HRMS (CI(NH_4)) calcd for ($C_{15}H_{18}O$ + H): 215.1430; found: 215.1431.

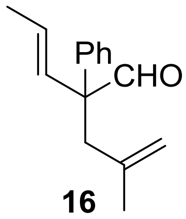

The formation of transient intermediate **16** could be observed by following the reaction at room temperature using ^{1}H NMR. By stopping the reaction before its completion, **16** could be isolated after purification by flash column chromatography on silica gel impregnated with 10% $AgNO_3$ (pentane/Et_2O = 99:1). Colourless oil (7.2 mg, 36%). ^{1}H NMR (500 MHz, $CDCl_3$): δ = 9.49 (s, 1H), 7.38-7.33 (m, 2H), 7.30-7.24 (m, 3H), 5.83 (dq, J = 16.1, 1.6 Hz, 1H), 5.54 (dq, J = 16.1, 6.4 Hz, 1H), 4.76-4.74 (m, 1H), 4.56-4.54 (m, 1H), 2.81 (d, J = 14.3 Hz, 1H), 2.76 (d, J = 14.4 Hz, 1H), 1.80 (dd, J = 6.4, 1.7 Hz, 3H), 1.46 (s, 3H); ^{13}C NMR (125 MHz, $CDCl_3$): δ = 198.7 (o), 141.3 (e), 139.1 (e), 130.1 (o), 130.0 (o), 128.6 (o, 2C), 128.4 (o, 2C), 127.3 (o), 115.2 (e), 60.3 (e), 43.1 (e), 24.5 (o), 18.7 (o); IR (neat): $\tilde{\nu}$ = 3075 (w), 3027 (w), 2967 (w), 2917 (w), 2855 (w), 2811 (w), 2717 (w), 1722 (vs), 1644 (w), 1599 (w), 1494 (w), 1447 (m), 1376 (m), 1317 (w), 1238 (w), 1188 (w), 1083 (w), 1029 (w), 974 (m), 894 (m), 758 (m), 700 (s) cm^{-1}; HRMS (CI(NH_4)) calcd for ($C_{15}H_{18}O$ + H): 215.1430; found: 215.1433.

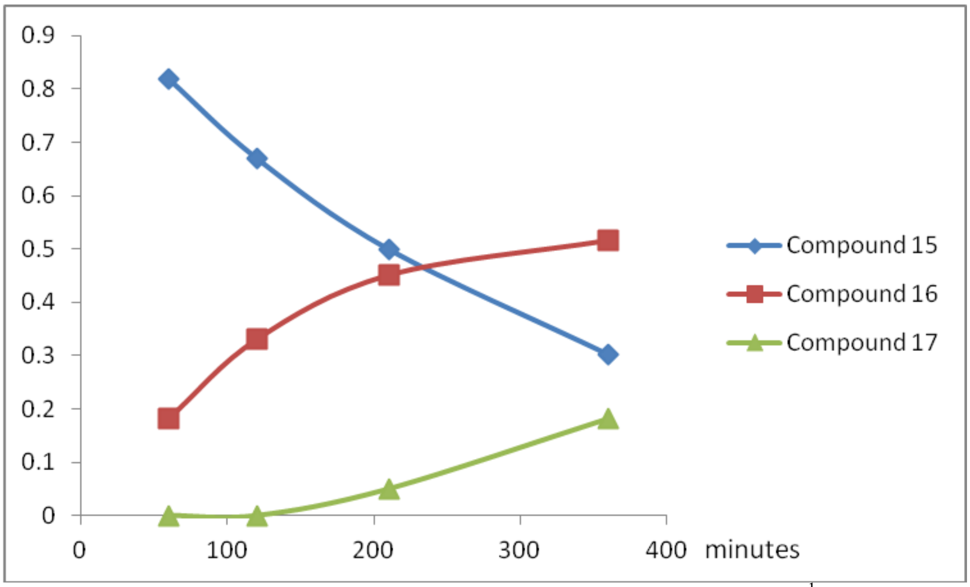

Figure SI-1. Ratios of **15**/**16**/**17** in the reaction at r.t. as measured by ^{1}H NMR

Supplementary examples of isomerization/hydroacylation tandem

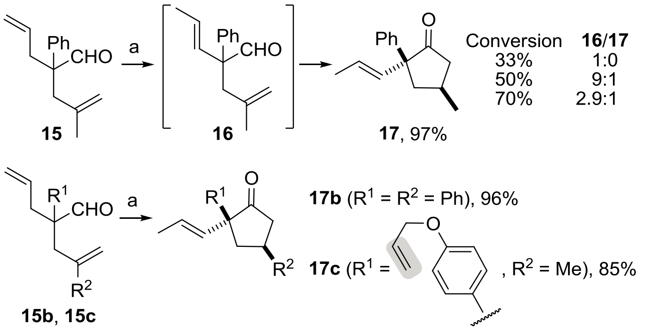

[(a)] $[Rh((\pm)\text{-}\mathbf{L1})]BF_4$ (10 mol%), acetone, 60 °C, 17h. The reaction of **15c** was performed at room temperature.

Compound 15b. Colourless oil.

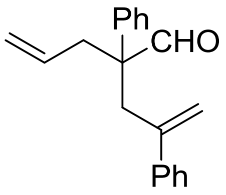

15b

^{1}H NMR (500 MHz, $CDCl_3$): δ = 9.39 (s, 1H), 7.31-7.25 (m, 2H), 7.23-7.14 (m, 6H), 7.14-7.10 (m, 2H), 5.48 (dddd, *J* = 17.1, 10.2, 7.5, 6.9 Hz, 1H), 5.18 (d, *J* = 1.6 Hz, 1H), 4.98 (ddt, *J* = 10.2, 2.1, 1.0 Hz, 1H), 4.94-4.88 (m, 2H), 3.24 (dd, *J* = 14.3, 0.9 Hz, 1H), 3.15 (dd, *J* = 14.3, 0.9 Hz, 1H), 2.71-2.61 (m, 2H); ^{13}C NMR (125 MHz, $CDCl_3$): δ = 200.8 (o), 144.8 (e), 142.1 (e), 137.7 (e), 132.7 (o), 128.4 (o, 2C), 127.9 (o, 2C), 127.7 (o, 2C), 127.2 (o), 127.1 (o), 126.6 (o, 2C), 118.8 (e), 118.0 (e), 57.4 (e), 38.1 (e), 36.2 (e); IR (neat): $\tilde{\nu}$ = 3079 (w), 3057 (w), 3024 (w), 2979 (w), 2921 (w), 2807 (w), 2714 (w), 1805 (w), 1720 (s), 1638 (w), 1626 (w), 1599 (w), 1574 (w), 1549 (w), 1494 (m), 1444 (m), 1386 (w), 1304 (w), 1261 (w), 1157 (w), 1075 (w), 1028 (w), 995 (w), 906 (s), 876 (m), 840 (w), 778 (s), 761 (m), 733 (m), 695 (vs), 666 (w) cm^{-1}; HRMS (CI(NH_4)) calcd for ($C_{20}H_{20}O$ + H): 277.1587; found: 277.1586.

Compound 15c. Colourless oil.

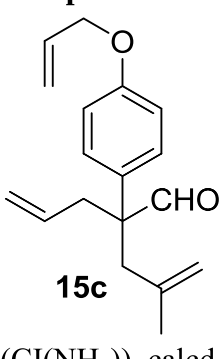

15c

^{1}H NMR (500 MHz, $CDCl_3$): δ = 9.50 (s, 1H), 7.16-7.12 (m, 2H), 6.94-6.90 (m, 2H), 6.05 (ddt, *J* = 17.3, 10.6, 5.3 Hz, 1H), 5.58 (ddt, *J* = 17.1, 10.2, 7.1 Hz, 1H), 5.41 (dq, *J* = 17.3, 1.6 Hz, 1H), 5.29 (dq, *J* = 10.5, 1.4 Hz, 1H), 5.09-5.03 (m, 2H), 4.85-4.82 (m, 1H), 4.67-4.65 (m, 1H), 4.53 (dt, *J* = 5.3, 1.5 Hz, 2H), 2.82-2.75 (m, 1H), 2.74-2.63 (m, 3H), 1.43 (s, 3H); ^{13}C NMR (125 MHz, $CDCl_3$): δ = 201.8 (o), 157.8 (e), 141.2 (e), 133.12 (o), 133.07 (o), 130.0 (e), 128.9 (o, 2C), 118.8 (e), 117.8 (e), 115.5 (e), 114.9 (o, 2C), 68.8 (e), 56.1 (e), 40.6 (e), 36.5 (e), 24.3 (o); IR (neat): $\tilde{\nu}$ = 3418 (w), 3077 (w), 2979 (w), 2919 (w), 2714 (w), 1719 (s), 1641 (m), 1607 (m), 1579 (m), 1509 (vs), 1453 (m), 1425 (m), 1376 (w), 1293 (m), 1246 (s), 1185 (s), 1120 (w), 1020 (m), 995 (s), 919 (s), 896 (w), 827 (s), 728 (w) cm^{-1}; HRMS (CI(NH_4)) calcd for ($C_{18}H_{22}O_2$ + H): 271.1693; found: 271.1691; elemental analysis (%) calcd for $C_{18}H_{22}O_2$: C 79.96, H 8.20; found: C 80.03, H 8.23.

Compound 17b. This compound was obtained from **15b** (0.072 mmol, 20 mg) following the representative procedure described for the preparation of **2**. Colourless oil (19.3 mg, 96%).

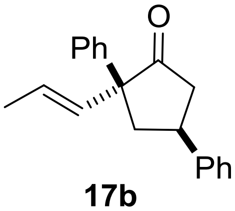

17b

^{1}H NMR (500 MHz, $CDCl_3$): δ = 7.39–7.29 (m, 8H), 7.28–7.22 (m, 2H), 5.71 (dq, *J* = 15.7, 6.4 Hz, 1H), 5.60-5.55 (m, 1H), 3.58-3.49 (m, 1H), 2.92 (ddd, *J* = 18.5, 7.6, 2.6 Hz, 1H), 2.77 (ddd, *J* = 12.8, 5.8, 2.6 Hz, 1H), 2.51 (dd, *J* = 14.5, 12.3 Hz, 1H), 2.47 (dd, *J* = 12.3, 8.6 Hz, 1H), 1.81 (dd, *J* = 6.4, 1.5 Hz, 3H); ^{13}C NMR (125 MHz, $CDCl_3$): δ = 215.8 (e), 142.7 (e), 142.4 (e), 131.7 (o), 128.7 (o, 2C), 128.3 (o, 2C), 127.7 (o), 127.2 (o, 2C), 126.83 (o, 2C), 126.77 (o, 2C), 61.5 (e), 46.1 (e), 45.1 (e), 37.8 (o), 18.2 (o); IR (neat): $\tilde{\nu}$ = 3058 (w), 3026 (w), 2915 (w), 1736 (vs), 1600 (m), 1496 (m), 1446 (m), 1404 (w), 1377 (w), 1302 (w), 1266 (w), 1205 (w), 1175 (w), 1148 (w), 1114 (m), 1083 (w), 1032 (w), 969 (m), 913 (w), 843 (w), 757 (s), 697 (vs) cm^{-1}; HRMS (ESI) calcd for ($C_{20}H_{20}O$ + Na): 299.1412; found: 299.1408; elemental analysis (%) calcd for $C_{20}H_{20}O$: C 86.92, H 7.29; found: C 86.39, H 7.35.

Compound 17c. This compound was obtained from **15c** (0.074 mmol, 20 mg) following the representative procedure described for the preparation of **2**. Colourless oil (16.9 mg, 85%). ^{1}H NMR (500 MHz, $CDCl_3$): δ = 7.24-7.20 (m, 2H), 6.88-6.85 (m, 2H), 6.04 (ddt, *J* = 17.2, 10.6, 5.3 Hz, 1H), 5.55 (dq, *J* = 15.7, 6.3 Hz, 1H), 5.48-5.42 (m, 1H), 5.40 (dq, *J* = 17.3, 1.6 Hz, 1H), 5.29-5.25 (m, 1H), 4.51 (dt, *J* = 5.3, 1.5 Hz, 2H), 2.61 (ddd, *J* = 18.5, 7.3, 2.5 Hz, 1H), 2.45 (ddd, *J* = 12.7, 5.7, 2.5 Hz, 1H), 2.40-2.27 (m, 1H), 1.95-1.87 (m, 2H), 1.73 (dd, *J* = 6.3, 1.4 Hz, 3H), 1.16 (d, *J* = 6.5 Hz, 3H); ^{13}C NMR (125 MHz, $CDCl_3$): δ = 217.7 (e), 157.3 (e), 135.1 (e), 133.4 (o), 132.3 (o), 128.3 (o, 2C), 126.8 (o), 117.6 (e), 114.4 (o, 2C), 68.8 (e), 61.1 (e), 46.9 (e), 46.0 (e), 27.4 (o), 20.2 (o), 18.1 (o); IR (neat): $\tilde{\nu}$ = 3025 (w), 2953 (m), 2925 (m), 2869 (m), 2335 (w), 2087 (w), 1737 (vs), 1649 (w), 1608 (m), 1579 (w), 1508 (vs), 1456 (m), 1425 (m), 1406 (m), 1378 (m), 1292 (m), 1244 (s), 1182 (s), 1147 (m), 1115 (m), 1080 (w), 1023 (s), 996 (s), 972 (s), 922 (s), 825 (s), 776 (w), 729 (w), 670 (w), 661 (w) cm^{-1}; HRMS (CI(NH_4)) calcd for ($C_{18}H_{22}O_2$ + H): 271.1693; found: 271.1703.

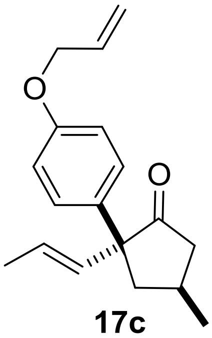

17c

Preparation of compounds (±)-19, (*R*)-19, (±)-20, (*S*,*S*)-20, (*S*)-21, (*R*)-22, and (*S*)-23

Compound (±)-19. This compound was obtained from **18** (0.196 mmol, 40 mg) following the representative procedure described for the preparation of **2**. Colourless oil (34.4 mg, 86%). ^{1}H NMR (500 MHz, $CDCl_3$): δ = 7.41-7.37 (m, 2H), 7.35-7.31 (m, 2H), 7.27-7.23 (m, 1H), 3.72 (d, *J* = 9.1 Hz, 1H), 3.44 (d, *J* = 9.2 Hz, 1H), 3.29 (s, 3H), 2.56-2.45 (m, 2H), 2.37-2.23 (m, 2H), 2.03-1.95 (m, 1H), 1.83-1.73 (m, 1H); ^{13}C NMR (125 MHz, $CDCl_3$): δ = 218.7 (e), 138.2 (e), 128.6 (o, 2C), 127.1 (o), 126.8 (o, 2C), 77.7 (e), 59.3 (o), 58.0 (e), 38.4 (e), 31.8 (e), 18.8 (e); IR (neat): $\tilde{\nu}$ = 2927 (w), 2886 (m), 1738 (vs), 1599 (w), 1497 (m), 1447 (m), 1404 (w), 1378 (w), 1314 (w), 1194 (m), 1157 (m), 1106 (s), 1047 (w), 984 (w), 937 (w), 908 (w), 811 (w), 759 (m), 700 (s) cm^{-1}; HRMS (CI(NH_4)) calcd for ($C_{13}H_{16}O_2$ + H): 205.1223; found: 205.1225; Chiral HPLC analysis (25 cm × 4.6 mm Chiralpak AD-H column), 2% isopropanol-hexane at 1 mL/min. flow rate, 230 nm; t_R (*49.5%*) 7.6 min., t_R (*50.5%*) 8.3 min.

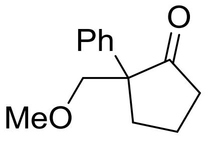

(±)-**19**

Compound (±)-20. This compound was obtained in the same reaction as side product. Colourless oil (2.1 mg, 5%); ^{1}H NMR (500 MHz, $CDCl_3$): δ = 7.43-7.38 (m, 2H), 7.35-7.30 (m, 2H), 7.26-7.22 (m, 1H), 3.63 (d, *J* = 9.2 Hz, 1H), 3.45 (d, *J* = 9.2 Hz, 1H), 3.49-3.34 (m, 1H), 3.33 (s, 3H), 2.80 (t, *J* = 10.7 Hz, 1H), 2.04 (dd, *J* = 11.0, 7.6 Hz, 1H), 1.12 (d, *J* = 7.6 Hz, 3H); ^{13}C NMR (125 MHz, $CDCl_3$): δ = 214.2 (e), 139.2 (e), 128.4 (o, 2C), 127.0 (o), 126.5 (o, 2C), 78.2 (e), 70.2 (e), 59.3 (o), 51.0 (o), 30.4 (e), 14.2 (o); IR (neat): $\tilde{\nu}$ =3058 (w), 2926 (m), 2871 (m), 2825 (m), 1775 (vs), 1600 (w), 1494 (m), 1447 (m), 1370 (w), 1267 (w), 1197 (w), 1173 (w), 1105 (vs), 1070 (w), 1028 (w), 962 (m), 913 (w), 762 (m), 730 (w), 700 (s) cm^{-1}; HRMS (CI(NH_4)) calcd for ($C_{13}H_{16}O_2$ + NH_4): 222.1495; found: 222.1495.

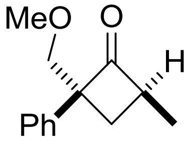

(±)-**20**

Compound (*R*)-19. [Rh(nbd)$_2$]BF_4 (0.0245, 9.2 mg) and (−)-(*R*)-**L1** (10 mmol%, 28.9 mg) were added to a flame-dried J-Young Schlenk flask under N_2. Degassed acetone (4.9 mL) was added and the orange solution was hydrogenated over 3 minutes *via* syringe (9.5 mL). The resultant pale orange solution was sealed and stirred for 1 hour at room temperature. The solution was degassed by the freeze-thaw method, before before being transferred *via* cannula to a second flame-dried J-Young Schlenk flask containing **18** (0.245 mmol, 50 mg). The tube was sealed and the reaction was stirred at room temperature for 17 hours. The solvent was removed under reduced pressure. Purification by flash column chromatography (petroleum ether/Et_2O, 99:1) afforded **(*R*)-19** as colourless oil (26 mg, 52%). $[\alpha]_D^{20} = +83°$ (*c* = 1, $CHCl_3$); Chiral HPLC analysis (25 cm x 4.6 mm Chiralpak AD-H column), 2% isopropanol-hexane at 1 mL/min. flow rate, 230 nm; t_R (*major*) 7.9 min., t_R (*minor*) 8.8 min., 96:4 enantiomeric ratio.

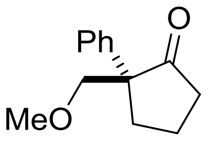

(*R*)-**19**

The absolute configuration of **(*R*)-19** was assigned by comparison with similar compounds.

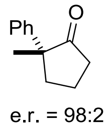

e.r. = 98:2

$[\alpha]_D^{20}$ = +54°

P. Nilsson, M. Larhed, A. Hallberg, *J. Am. Chem. Soc.* **2003**, *125*, 3430

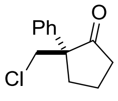

e.r. = 98:2

$[\alpha]_D^{20}$ = +85°

Q. Yin, S.-Y. You, *Org. Lett.* **2014**, *16*, 1810

Compounds **(*S*)-21** and **(*S,S*)-20** were isolated from the same reaction as inseparable mixture and their ratio was determined by ^{1}H NMR (20.5 mg, 41%).The alcohol **(*R*)-22** could be isolated as clean product after reduction of the mixture with $LiAlH_4$. **SI-1** was not isolated.

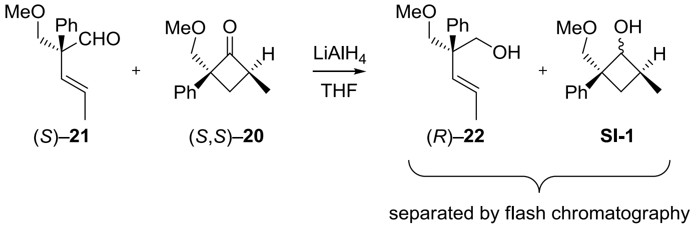

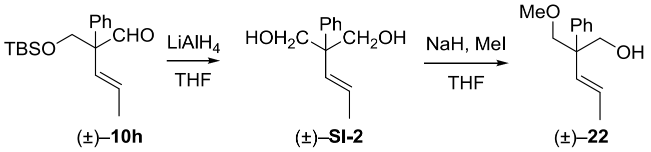

Compound (*R*)-22. ^{1}H NMR (500 MHz, $CDCl_3$): δ = 7.39-7.31 (m, 4H), 7.26-7.21 (m, 1H), 5.59-5.54 (m, 1H), 5.48 (dq, *J* = 15.9, 6.1 Hz, 1H), 4.05 (dd, *J* = 11.1, 6.5 Hz, 1H), 3.94 (dd, *J* = 11.1, 5.3 Hz, 1H), 3.83 (d, *J* = 9.2 Hz, 1H), 3.77 (d, *J* = 9.2 Hz, 1H), 3.38 (s, 3H), 2.46 (t, *J* = 6.1 Hz, 1H), 1.74 (dd, *J* = 6.1, 1.3 Hz, 3H); ^{13}C NMR (125 MHz, $CDCl_3$): δ = 142.1 (e), 133.1 (o), 128.3 (o, 2C), 127.5 (o, 2C), 126.8 (o), 126.6 (o), 78.6 (e), 68.3 (e), 59.4 (o), 50.1 (e), 18.5 (o); IR (neat): $\tilde{\nu}$ = 3425 (br), 3056 (w), 3027 (w), 2918 (w), 2882 (w), 1600 (w), 1495 (m), 1447 (m), 1377 (w), 1248 (w), 1194 (m), 1103 (s), 1038 (m), 1021 (m), 970 (s), 927 (w), 761 (s), 697 (vs) cm^{-1}; HRMS (CI(NH_4)) calcd for ($C_{13}H_{18}O_2$ + H): 207.1380; found: 207.1377, $[\alpha]_D^{20} = -11°$ (*c* = 1, $CHCl_3$); Chiral HPLC analysis (25 cm x 4.6 mm Chiralpak AD-H column), 2% isopropanol-hexane at 1 mL/min. flow rate, 230 nm; t_R (*major*) 10.9 min., t_R (*minor*) 12.1 min., 98.5:1.5 enantiomeric ratio.

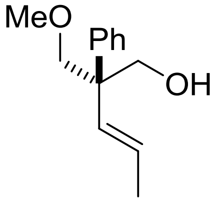

(*R*)–**22**

Compound (±)-SI-2. A solution of **10h** (0.12 mmol, 37 mg) in THF (1.2 mL) was added under N_2 to a suspension of $LiAlH_4$ (0.18 mmol, 7 mg) at 0 °C. After stirring at room temperature for 30 minutes, another portion of $LiAlH_4$ (0.18 mmol, 7 mg) was added. After stirring for 30 minutes, the TBS group was cleaved and the reaction mixture was quenched carefully at 0 °C by a dropwise addition of a saturated aqueous solution of sodium sulfate. The white precipitate was filtered over celite pad and the filtrate was evaporated under reduced pressure. Purification by flash chromatography (petroleum ether/Et_2O = 4:1) afforded **(±)-SI-2** as a colourless oil (20 mg, 87%); ^{1}H NMR (500 MHz, $CDCl_3$): δ = 7.39-7.33 (m, 4H), 7.28-7.23 (m, 1H), 5.62-5.53 (m, 2H), 4.05 (d, *J* = 11.1 Hz, 2H), 3.96 (d, *J* = 11.1 Hz, 2H), 2.09 (br s, 2H), 1.78 (d, *J* = 4.7 Hz, 3H); ^{13}C NMR (125 MHz, $CDCl_3$): δ = 141.5 (e), 132.6 (o), 128.6 (o, 2C), 128.0 (o), 127.7 (o, 2C), 126.9 (o), 67.7 (e, 2C), 51.1 (e), 18.6 (o); IR (neat): $\tilde{\nu}$ = 3288 (br), 3027 (w), 2931 (m), 2881 (w), 2357 (w), 2342 (w), 2325 (w), 1600 (w), 1493 (m), 1445 (m), 1374 (w), 1256 (w), 1159 (w), 1047 (s), 1015 (s), 980 (m), 908 (w), 837 (w), 761 (m), 733 (m), 696 (vs) cm^{-1}; HRMS (CI(NH_4)) calcd for ($C_{12}H_{16}O_2$ + NH_4): 210.1489; found: 210.1498.

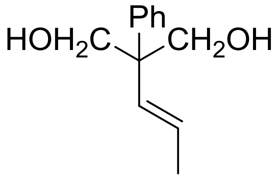

(±)–**SI-2**

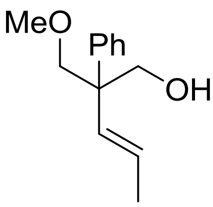

(±)–**22**

Compound (±)-22. Under a N_2 atmosphere, NaH (0.078 mmol, 3.1 mg, 60% dispersion in oil) was suspended in THF (1 mL) and the mixture was cooled to 0 °C. **(±)-SI-2** (0.078 mmol, 15 mg) in THF (0.5 mL) was added *via* syringe and was stirred to room temperature for 15 minutes. Methyl iodide (0.078 mmol, 5 μL) was added and the reaction was stirred at room temperature for 1 hour. The reaction mixture was quenched with a saturated aqueous solution of ammonium chloride and extracted with Et_2O. The organic layer was dried over $MgSO_4$, filtered and concentrated. Purification

by flash column chromatography (petroleum ether/Et_2O = 98:1) afforded **(±)-22** as a colourless oil (4.5 mg, 28%); ^1H NMR (500 MHz, $CDCl_3$): δ = 7.39-7.31 (m, 4H), 7.26-7.21 (m, 1H), 5.59-5.54 (m, 1H), 5.48 (dq, *J* = 15.9, 6.1 Hz, 1H), 4.05 (dd, *J* = 11.1, 6.5 Hz, 1H), 3.94 (dd, *J* = 11.1, 5.3 Hz, 1H), 3.83 (d, *J* = 9.2 Hz, 1H), 3.77 (d, *J* = 9.2 Hz, 1H), 3.38 (s, 3H), 2.46 (t, *J* = 6.1 Hz, 1H), 1.74 (dd, *J* = 6.1, 1.3 Hz, 3H); Chiral HPLC analysis (25 cm x 4.6 mm Chiralpak AD-H column), 2% isopropanol-hexane at 1 mL/min. flow rate, 230 nm; t_R (*50%*) 10.95 min., t_R (*50%*) 12.0 min.

Compound (*S*)-23. Triethylamine (0.29 mmol, 40 μL) was added to **(*R*)-22** (0.15 mmol, 30 mg), dissolved in CH_2Cl_2 (1.4 mL) and stirred for 5 minutes, before adding 3,5-dinitrobenzoyl chloride (0.29 mmol, 67 mg) at room temperature which was stirred for a further 20 minutes. The reaction was quenched with saturated ammonium chloride, extracted in Et_2O, dried over magnesium sulfate, filtered and concentrated. The crude was purified by flash column chromatography (pentane/Et_2O = 9:1) afforded a white solid (49 mg, 84%); which was recrystallized by dissolving **(*S*)-23** in a minimal amount of EtOAc, then layering hexane (EtOAc/hexane = 1:3 ratio) which was left on the bench for two days to obtain white crystals. Melting point = 68-70 °C; ^1H NMR (500 MHz, $CDCl_3$): δ = 9.19 (t, *J* = 2.2 Hz, 1H), 9.01 (d, *J* = 2.2 Hz, 2H), 7.41-7.34 (m, 4H), 7.29-7.25 (m, 1H), 5.71 (dq, *J* = 15.9, 1.5 Hz, 1H), 5.58 (dq, *J* = 16.0, 6.3 Hz, 1H), 4.81 (d, *J* = 10.9 Hz, 1H), 4.78 (d, *J* = 10.9 Hz, 1H), 3.80 (s, 2H), 3.36 (s, 3H), 1.78 (dd, *J* = 6.4, 1.6 Hz, 3H); ^{13}C NMR (125 MHz, $CDCl_3$): δ = 162.2 (e), 148.6 (e, 2C), 141.2 (e), 134.0 (e), 132.3 (o), 129.3 (o, 2C), 128.4 (o, 2C), 127.3 (o, 2C), 127.1 (o), 127.0 (o), 122.2 (o), 76.3 (e), 69.1 (e), 59.4 (o), 48.8 (e), 18.5 (o); IR (neat): $\tilde{\nu}$ = 3100 (w), 2918 (w), 1732 (s), 1629 (m), 1598 (w), 1542 (vs), 1496 (w), 1459 (m), 1448 (w), 1342 (vs), 1271 (vs), 1165 (s), 1106 (m), 1075 (m), 1030 (w), 974 (s), 911 (s), 830 (w), 766 (m), 728 (vs), 719 (vs), 699 (vs) cm^{-1}; HRMS (ESI) calcd for ($C_{20}H_{20}N_2O_7$ + Na): 423.1168; found: 423.1161; $[\alpha]_D^{20} = -13°$ (*c* = 1, $CHCl_3$).

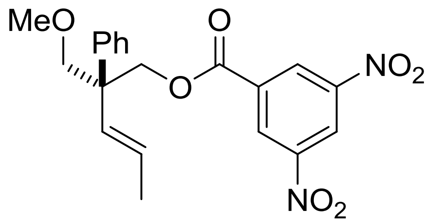

(*S*)-**23**

Calculation of enantiomeric excess of (*S*,*S*)-21 and determination of its absolute configuration

Considering the isolated yield and the enantiomeric purity of (*R*)-**19** and (*S*)-**20**, we deduced that (*R*)-**18** was completely consumed. The enantiomeric ratio for (*S*,*S*)-**21** was then calculated using the crude ratio (*S*)-**18**/(*R*)-**19**/(*S*)-**20**/(*S*,*S*)-**21** = 0.07:1:0.62:0.2 and Horeau's equation:

$$e.e._{18} \times y_{18} + e.e._{19} \times y_{19} + e.e._{20} \times y_{20} + e.e._{21} \times y_{21} = 0$$

With y is the molar fraction of **18**–**21** and the e.e. of (*R*)-**19** being arbitrarily assumed to be positive (e.e. = enantiomeric excess), we found $e.e._{21}$ = –92%.

Kinetic resolution of 16

Kinetic resolution, as performed for **(±)-18**, was also conducted on **16** (0.0933 mmol, 20 mg) to afford (+)-**16** (8 mg, 40%, $[\alpha]_D^{20} = +15.5°$ (c = 0.5, $CHCl_3$)) and (–)-**17** (10 mg, 50%, $[\alpha]_D^{20} = -91°$ (c = 1, $CHCl_3$)). Chiral HPLC analysis (25 cm x 4.6 mm Chiralpak AD-H column), 2% isopropanol-hexane at 1 mL/min. flow rate, 230 nm; t_R (*minor*) 9.3 min., t_R (*major*) 11.9 min., 2:98 enantiomeric ratio. The e.r. of (+)-**16** could not be measured directly but was determined on alcohol **SI-3**, obtained after $LiAlH_4$-reduction: chiral HPLC analysis (25 cm x 4.6 mm Chiralpak AD-H column), 6% isopropanol-hexane at 1 mL/min. flow rate, 230 nm; t_R (*minor*) 8.95 min., t_R (*major*) 11.5 min., 1:99 enantiomeric ratio. The absolute configuration of (+)-**16** and (–)-**17** was not determined.

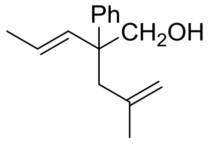

SI-3

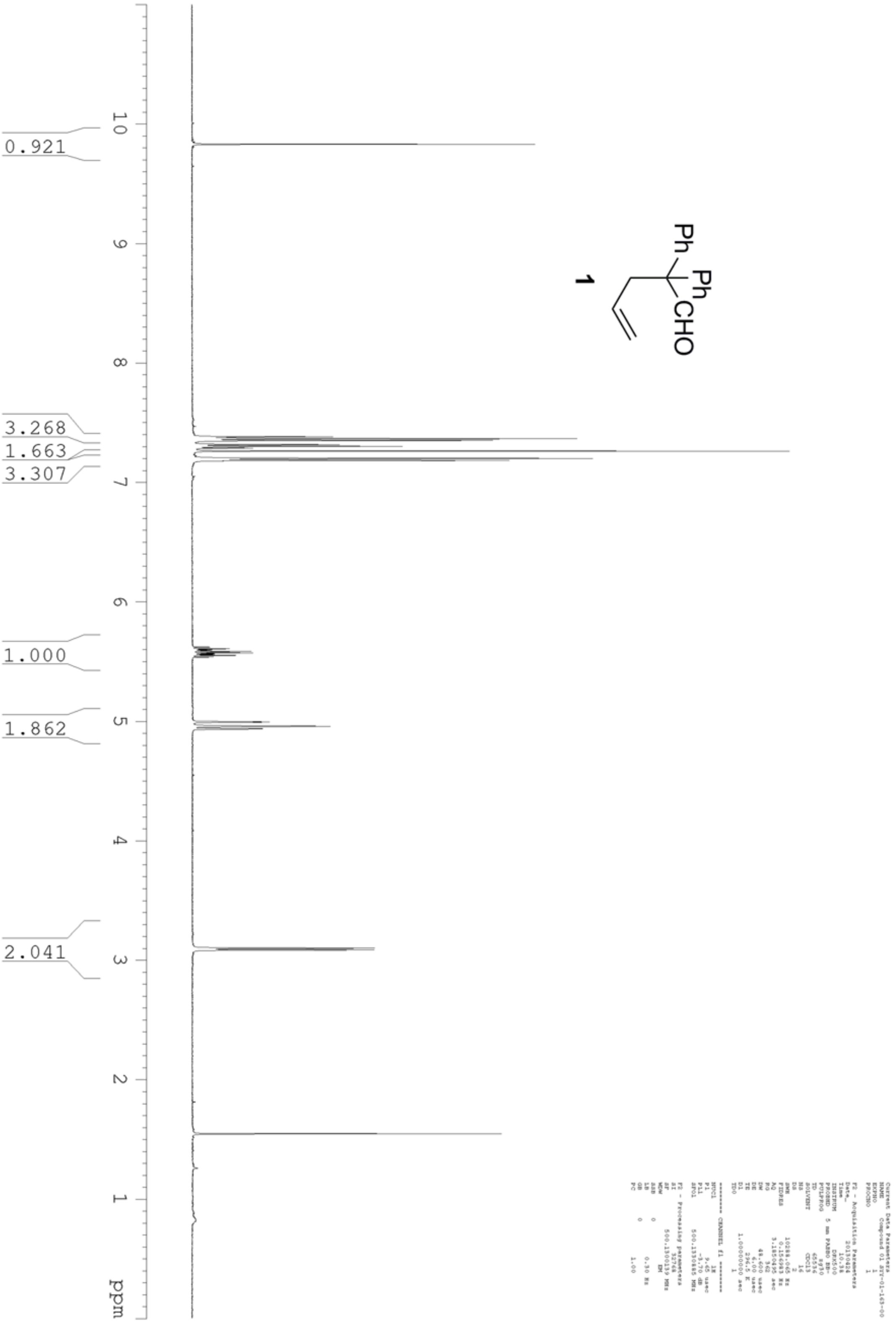
0.921
3.268
1.663
3.307
1.000
1.862
2.041
10
9
8
7
6
5
4
3
2
1
ppm
Ph
Ph
CHO
1

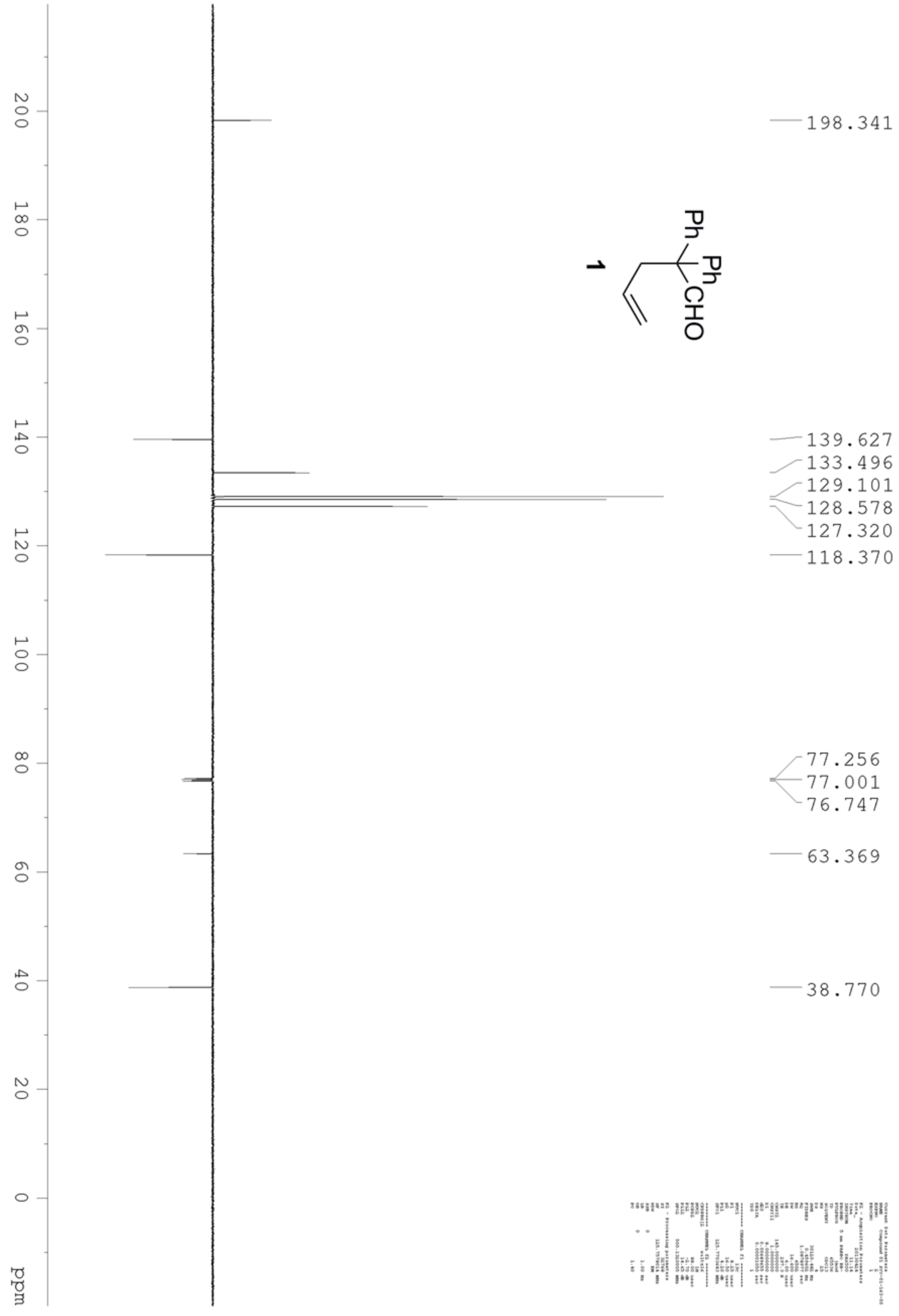
198.341
139.627
133.496
129.101
128.578
127.320
118.370
77.256
77.001
76.747
63.369
38.770
Ph
Ph
CHO
1
ppm

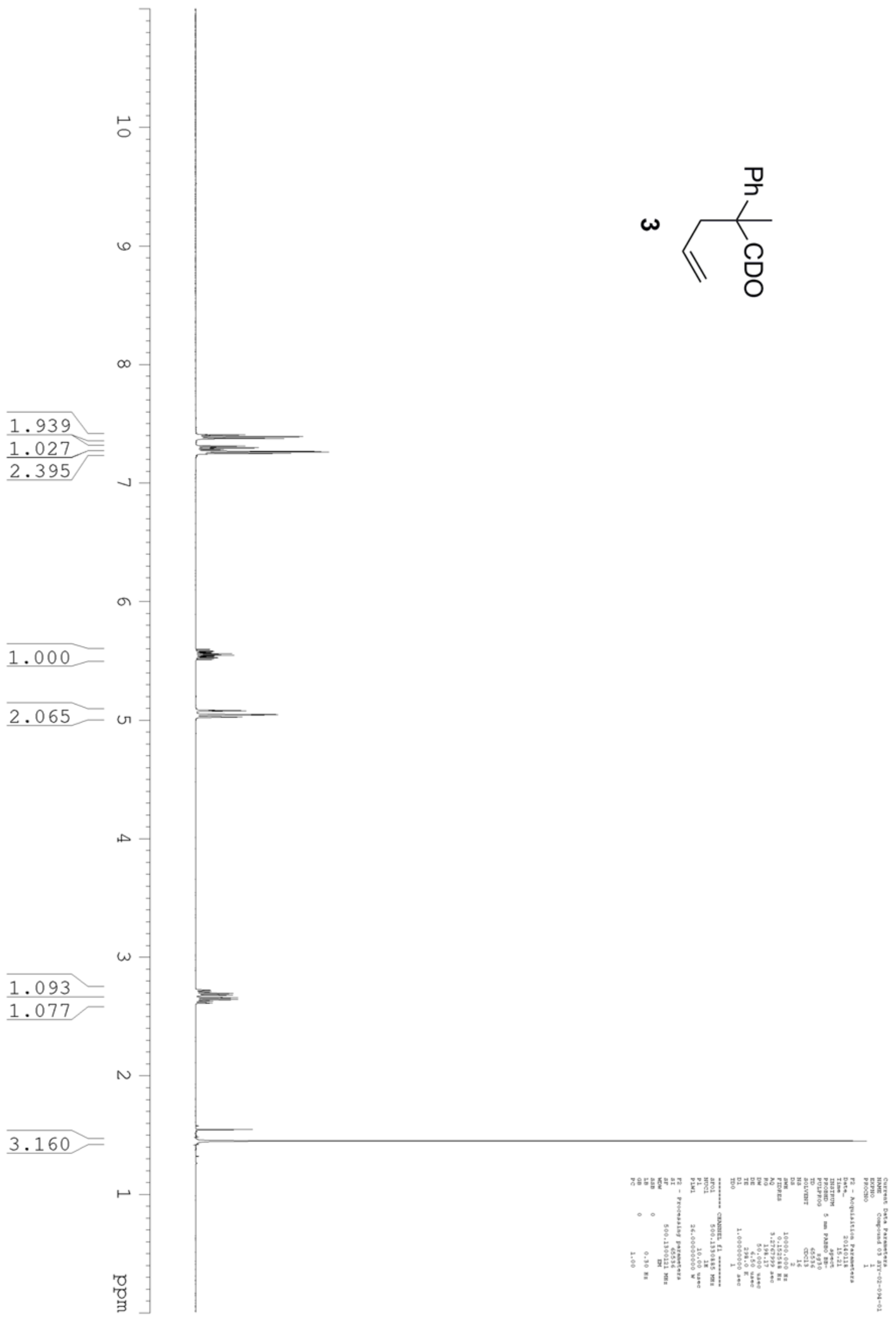

Ph
CDO
3
1.939
1.027
2.395
1.000
2.065
1.093
1.077
3.160
10
9
8
7
6
5
4
3
2
1
ppm

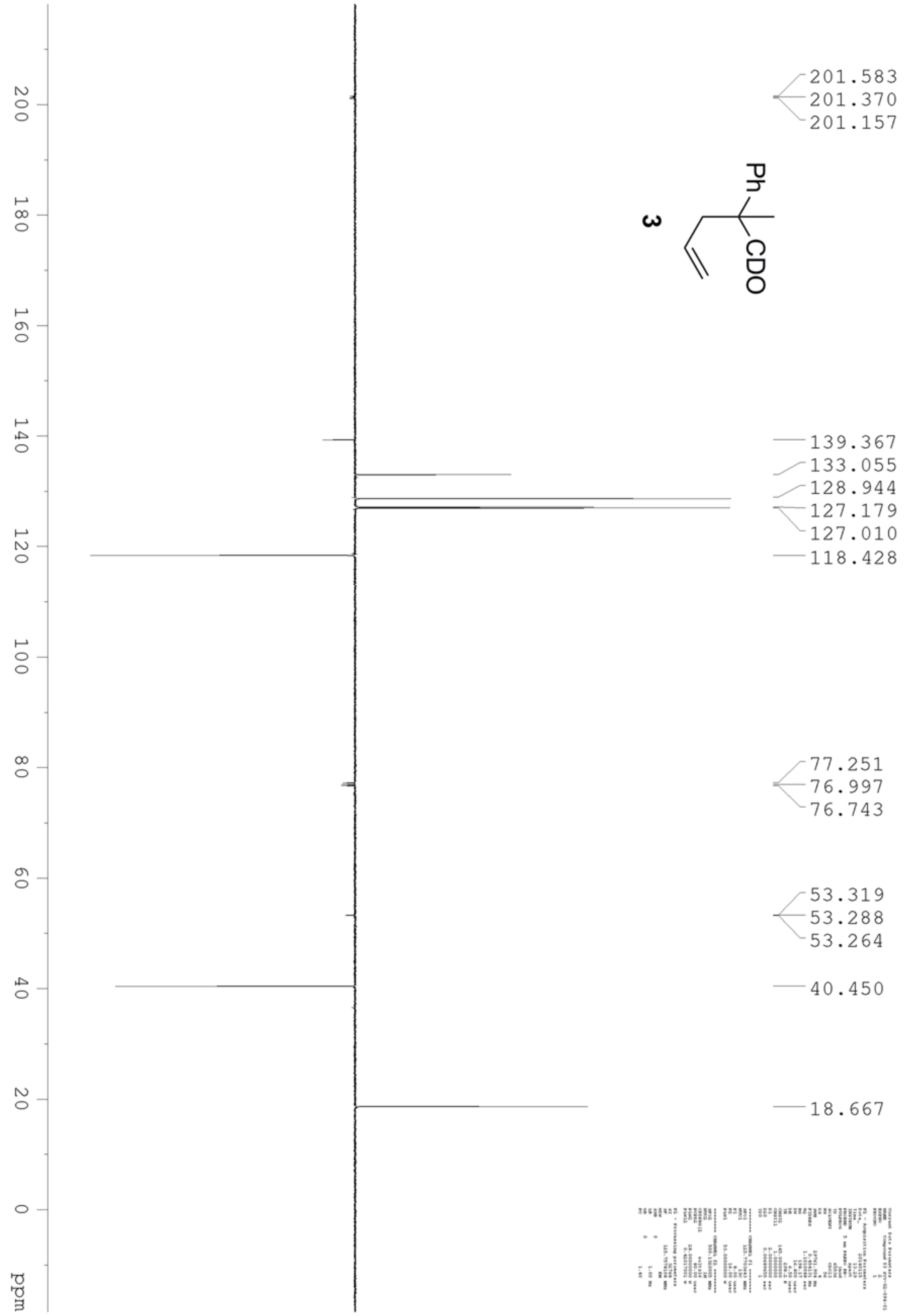

201.583
201.370
201.157
Ph
CDO
3
139.367
133.055
128.944
127.179
127.010
118.428
77.251
76.997
76.743
53.319
53.288
53.264
40.450
18.667
200
180
160
140
120
100
80
60
40
20
0
ppm

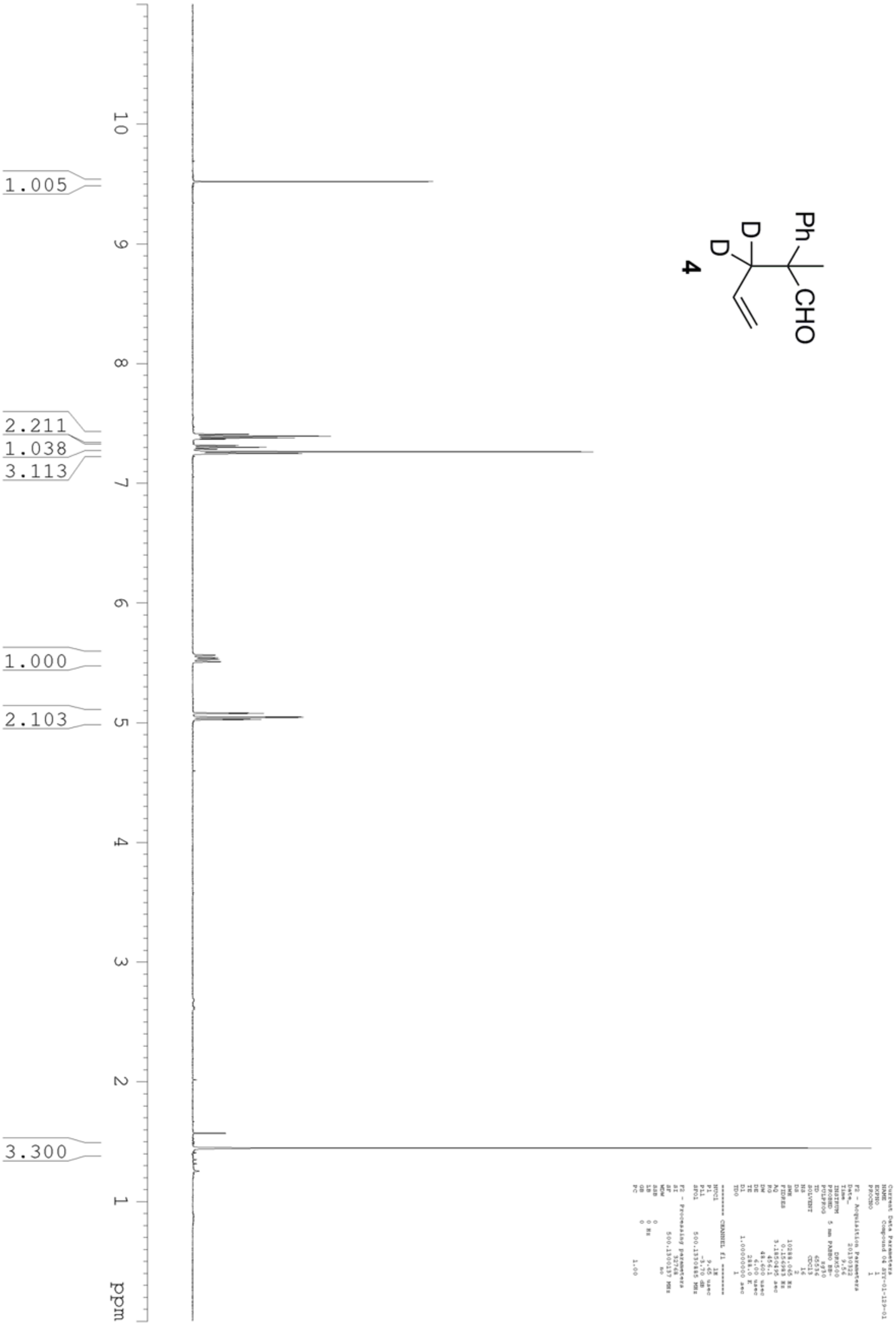
Ph
CHO
D
D
4
1.005
2.211
1.038
3.113
1.000
2.103
3.300
10
9
8
7
6
5
4
3
2
1
ppm

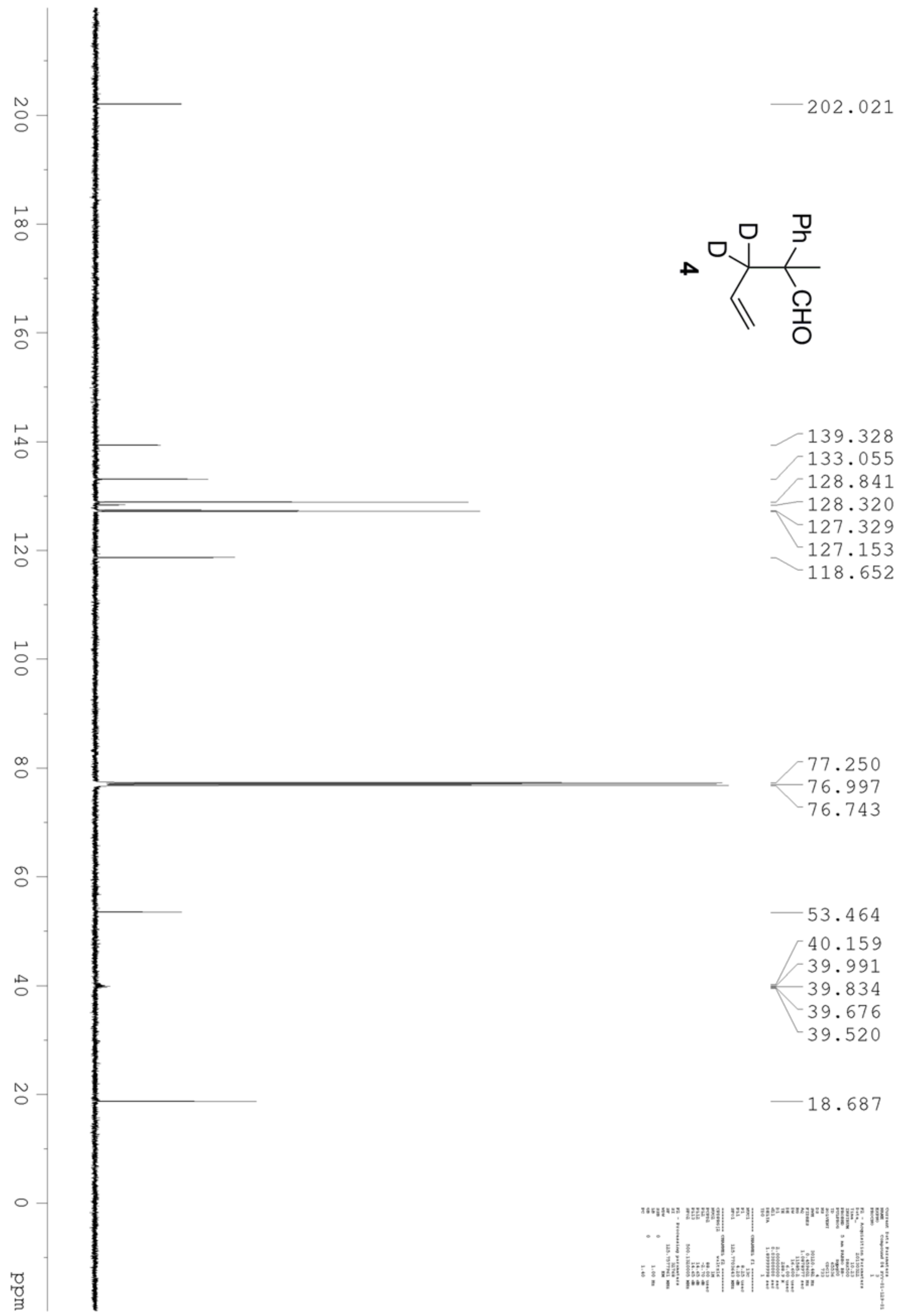
202.021
139.328
133.055
128.841
128.320
127.329
127.153
118.652
77.250
76.997
76.743
53.464
40.159
39.991
39.834
39.676
39.520
18.687
Ph
CHO
D
D
4
ppm

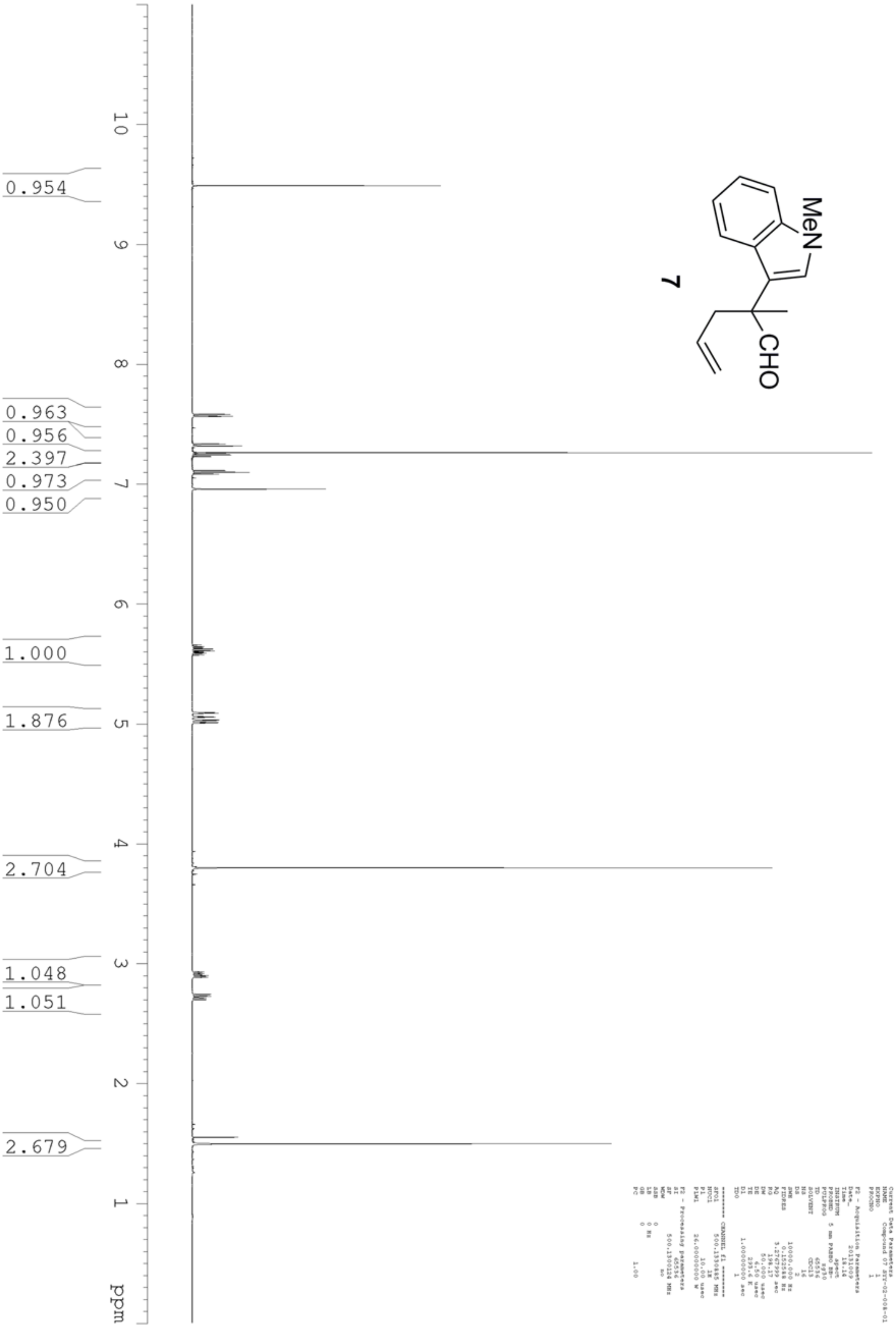
0.954
0.963
0.956
2.397
0.973
0.950
1.000
1.876
2.704
1.048
1.051
2.679
10
9
8
7
6
5
4
3
2
1
ppm
MeN
CHO
7

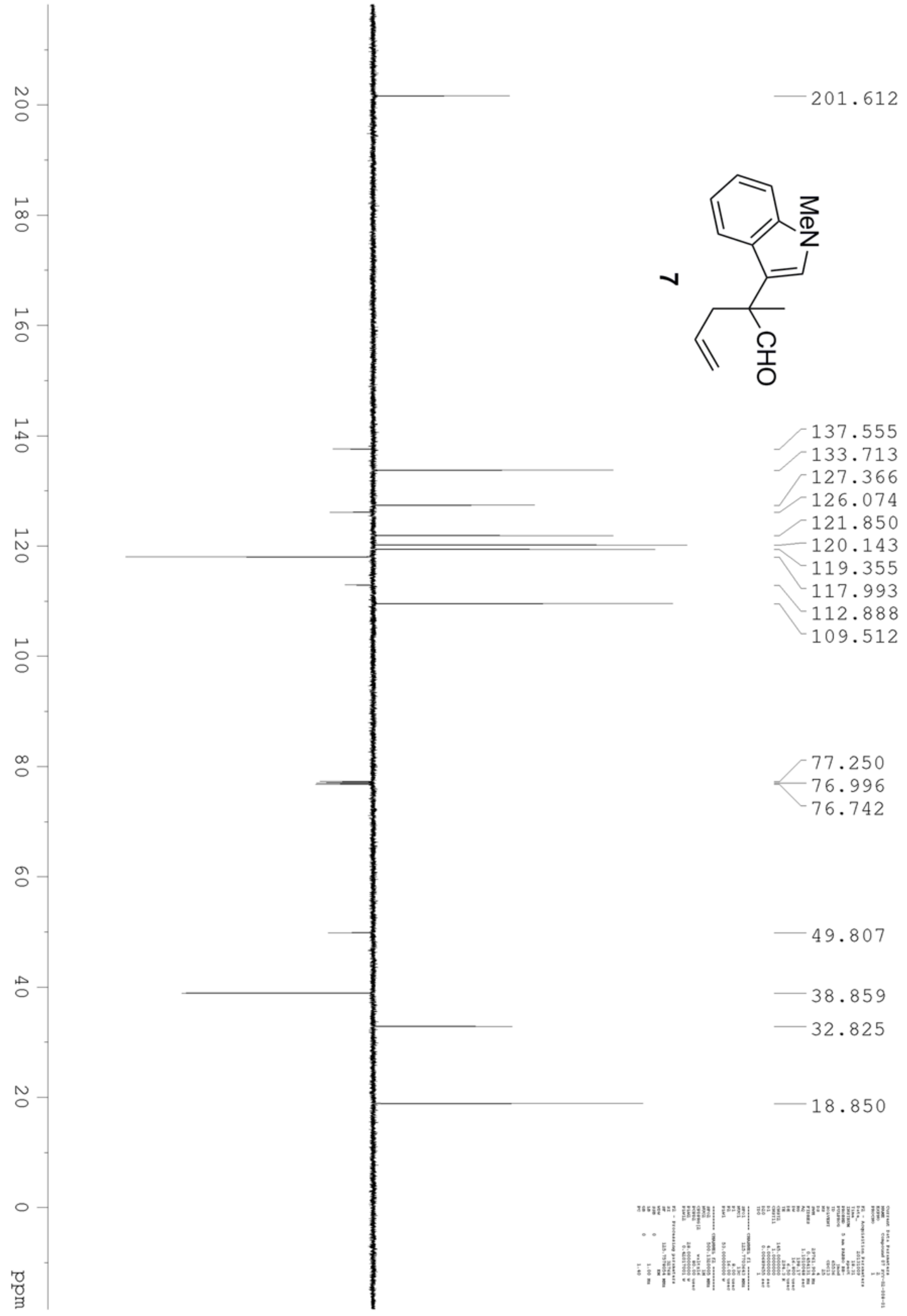
201.612
MeN
CHO
7
137.555
133.713
127.366
126.074
121.850
120.143
119.355
117.993
112.888
109.512
77.250
76.996
76.742
49.807
38.859
32.825
18.850
ppm

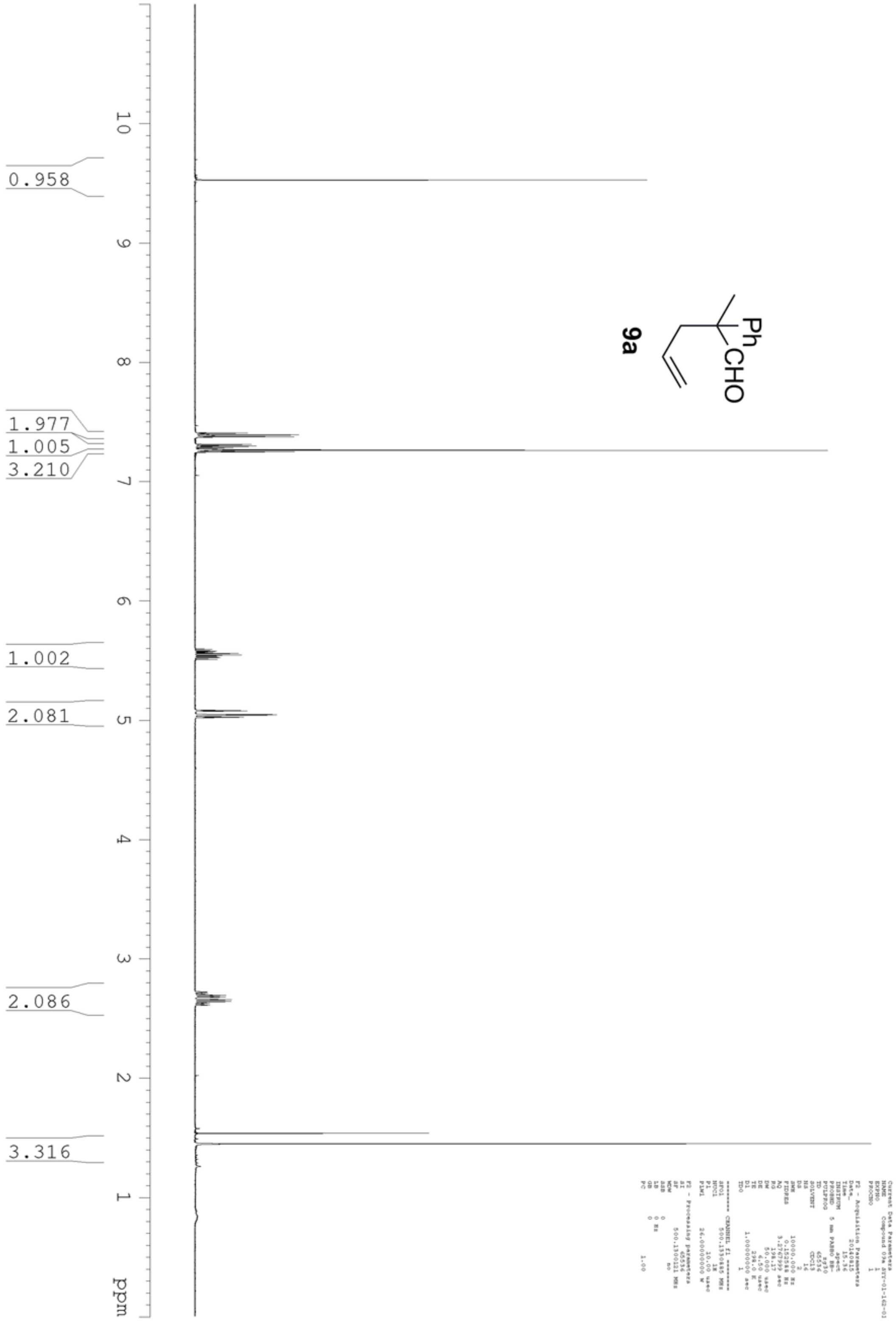

Ph
CHO
9a
0.958
1.977
1.005
3.210
1.002
2.081
2.086
3.316
10
9
8
7
6
5
4
3
2
1
ppm

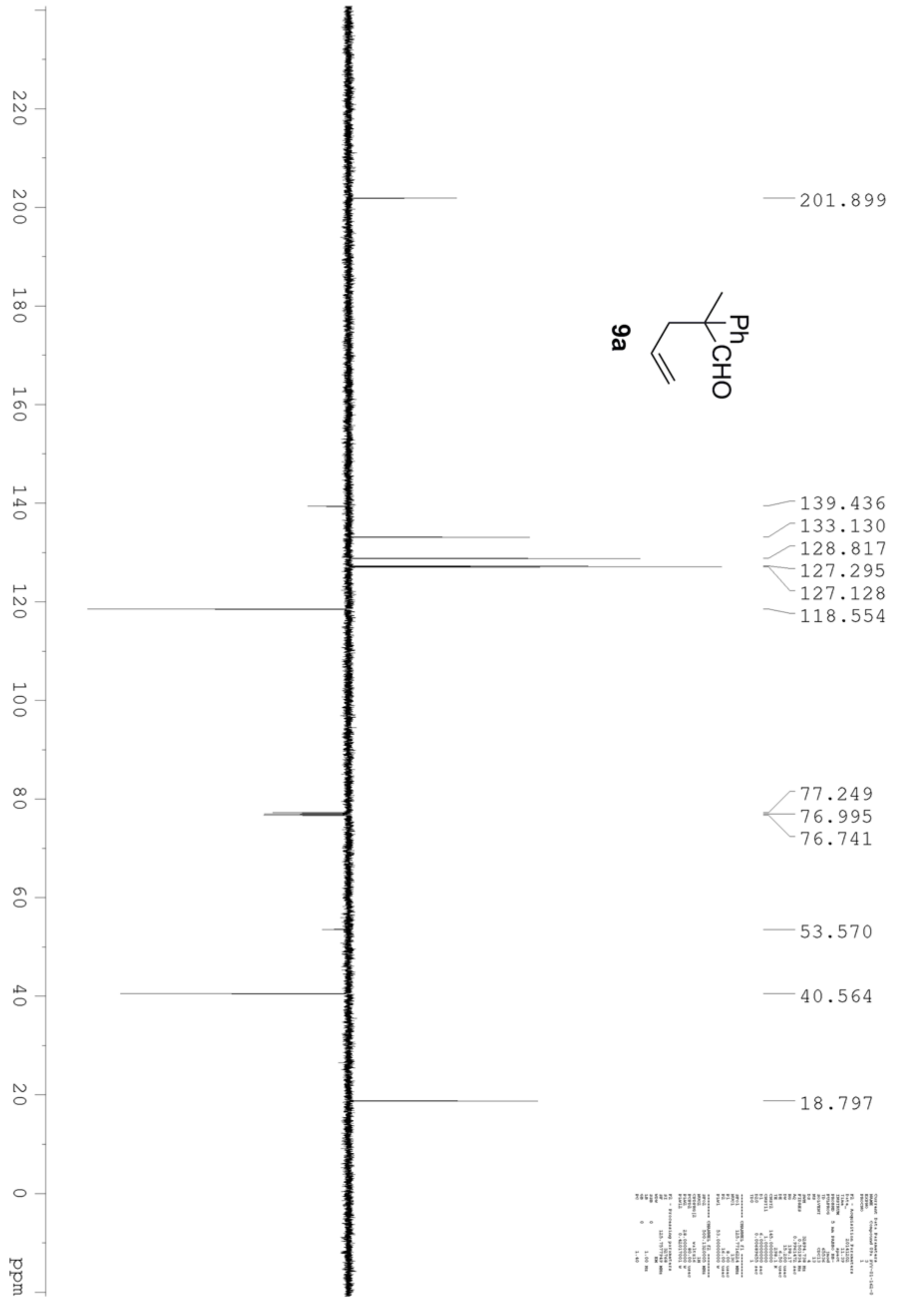
201.899
Ph
CHO
9a
139.436
133.130
128.817
127.295
127.128
118.554
77.249
76.995
76.741
53.570
40.564
18.797
220
200
180
160
140
120
100
80
60
40
20
0
ppm

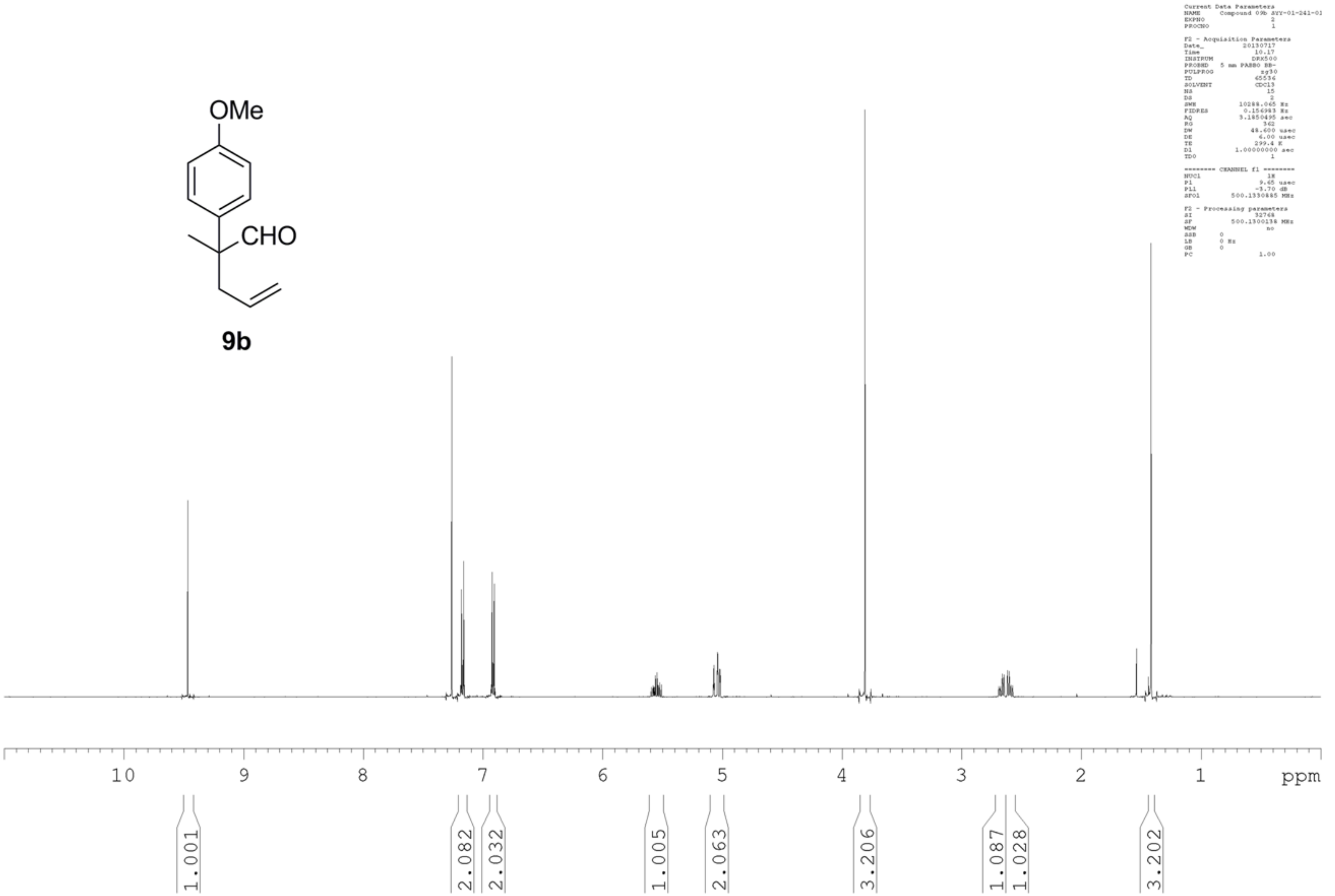
OMe
CHO
9b
Current Data Parameters
NAME Compound 09b SYY-01-241-01
EXPNO 2
PROCNO 1
F2 - Acquisition Parameters
Date_ 20130717
Time 10.17
INSTRUM DRX500
PROBHD 5 mm PABBO BB-
PULPROG zg30
TD 65536
SOLVENT CDCl3
NS 15
DS 2
SWH 10288.065 Hz
FIDRES 0.156983 Hz
AQ 3.1850495 sec
RG 362
DW 48.600 usec
DE 6.00 usec
TE 299.4 K
D1 1.00000000 sec
TD0 1
======== CHANNEL f1 ========
NUC1 1H
P1 9.65 usec
PL1 -3.70 dB
SFO1 500.1330885 MHz
F2 - Processing parameters
SI 32768
SF 500.1300138 MHz
WDW no
SSB 0
LB 0 Hz
GB 0
PC 1.00
10
9
8
7
6
5
4
3
2
1
ppm
1.001
2.082
2.032
1.005
2.063
3.206
1.087
1.028
3.202

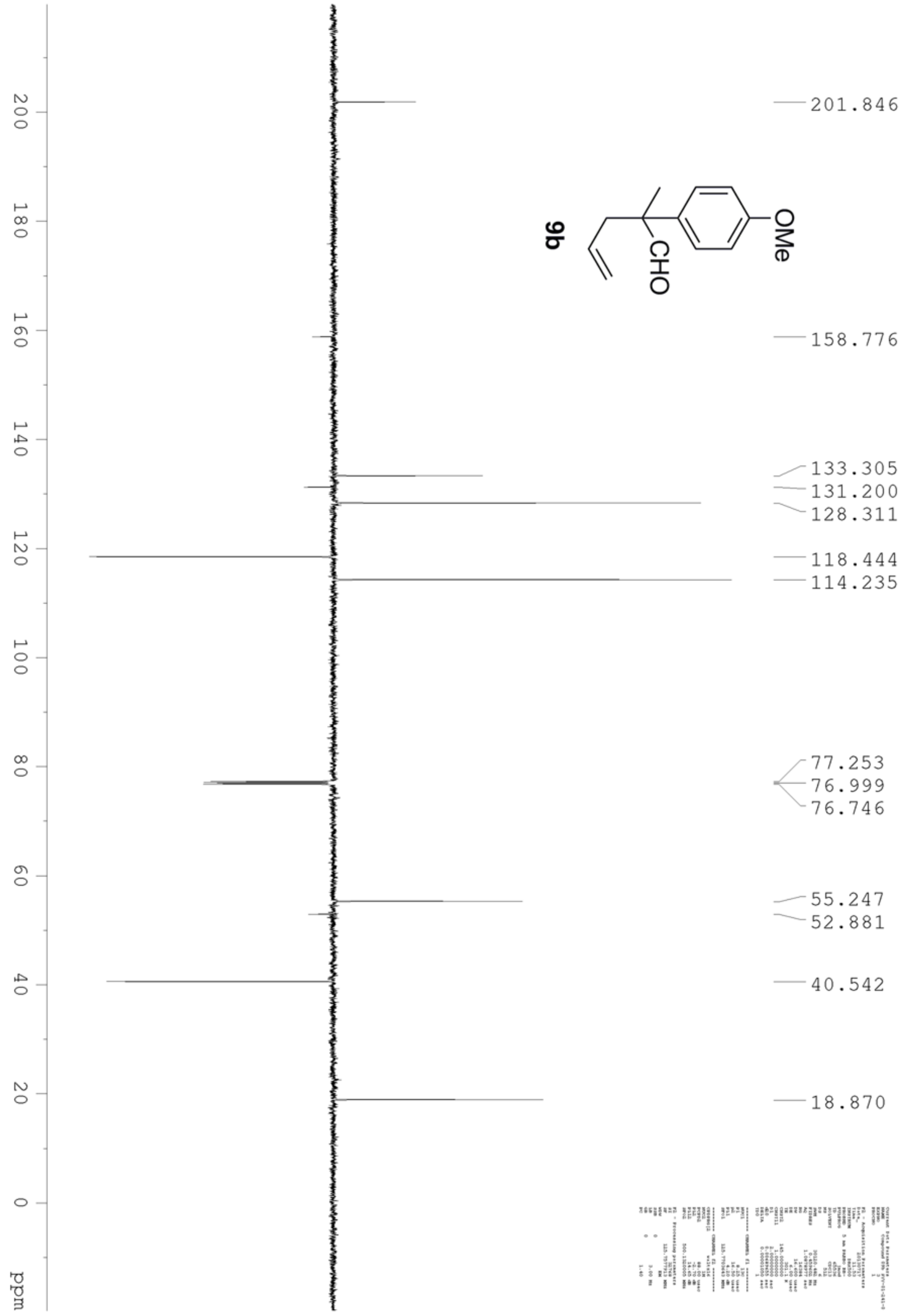
9b
CHO
OMe
201.846
158.776
133.305
131.200
128.311
118.444
114.235
77.253
76.999
76.746
55.247
52.881
40.542
18.870
200
180
160
140
120
100
80
60
40
20
0
ppm

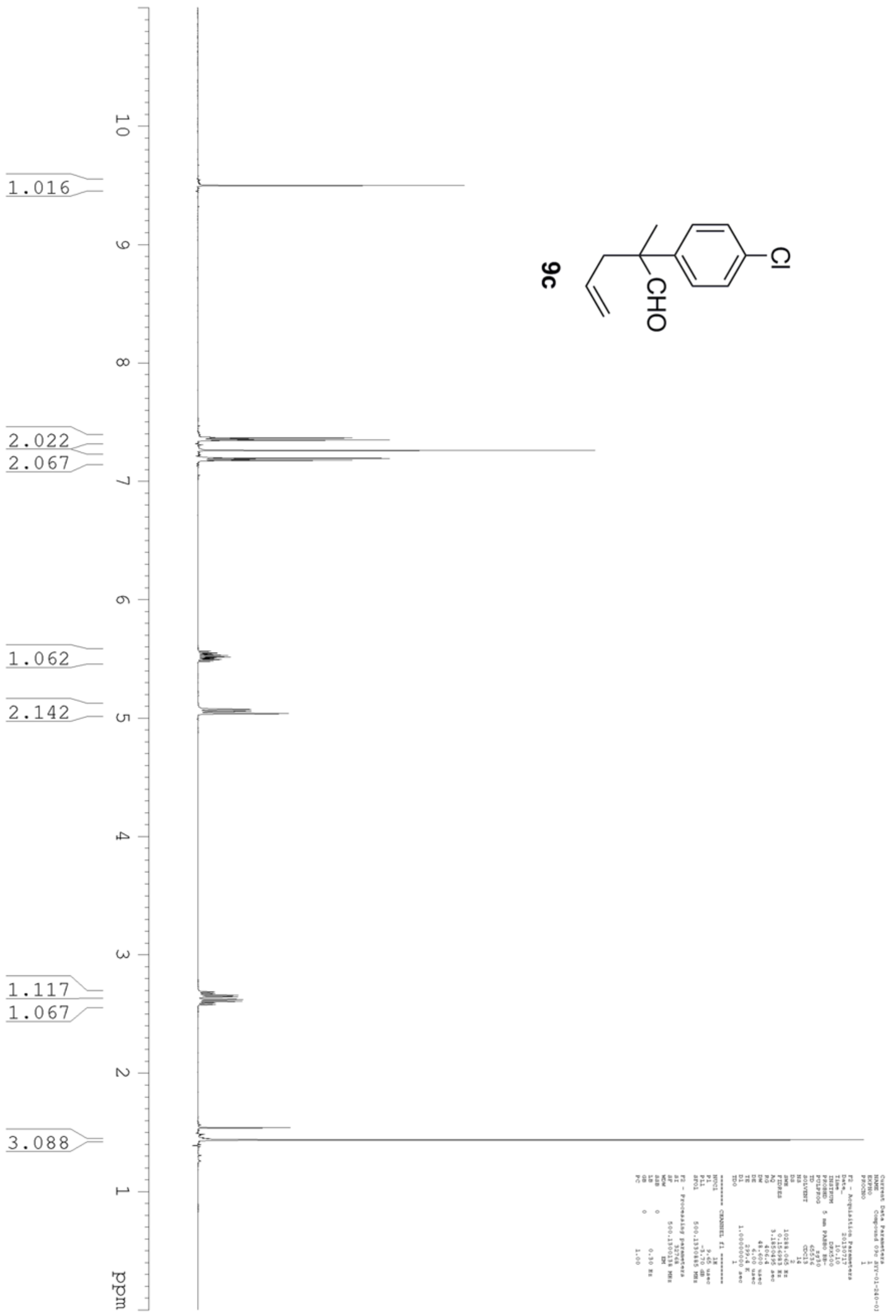
1.016
2.022
2.067
1.062
2.142
1.117
1.067
3.088
10
9
8
7
6
5
4
3
2
1
ppm
9c
CHO
Cl

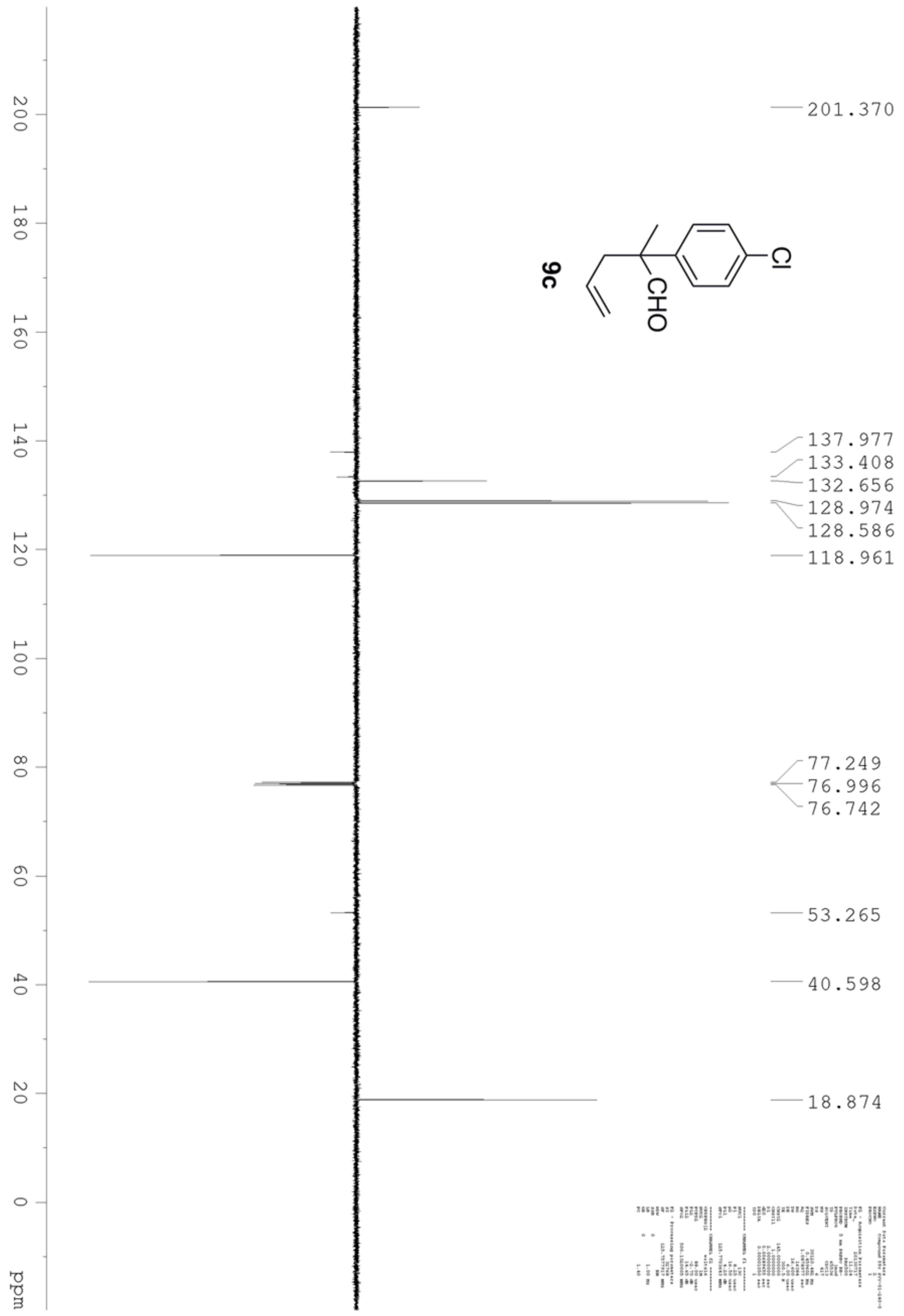
201.370
9c
CHO
Cl
137.977
133.408
132.656
128.974
128.586
118.961
77.249
76.996
76.742
53.265
40.598
18.874
ppm

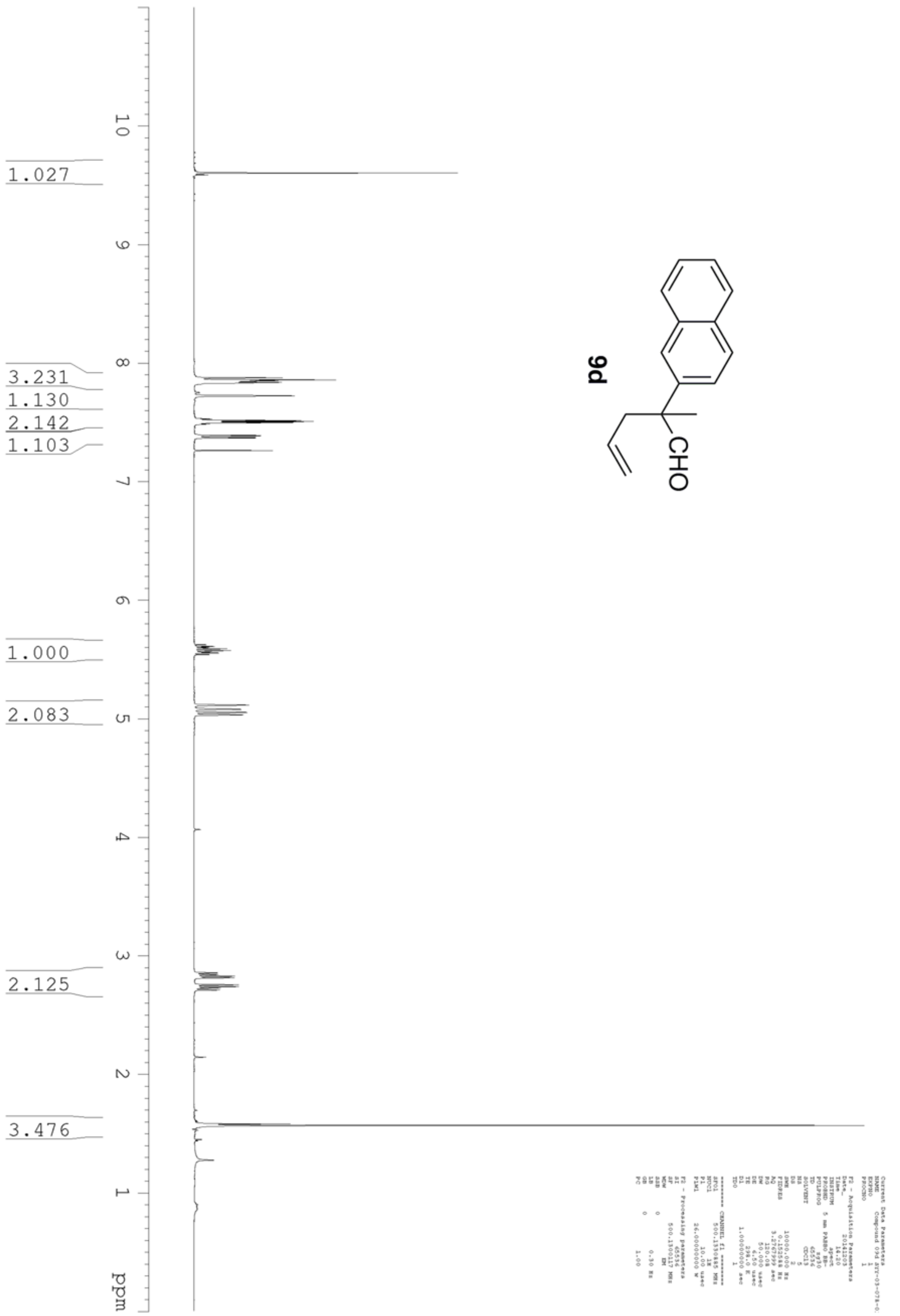
9d
CHO
1.027
3.231
1.130
2.142
1.103
1.000
2.083
2.125
3.476
10
9
8
7
6
5
4
3
2
1
ppm

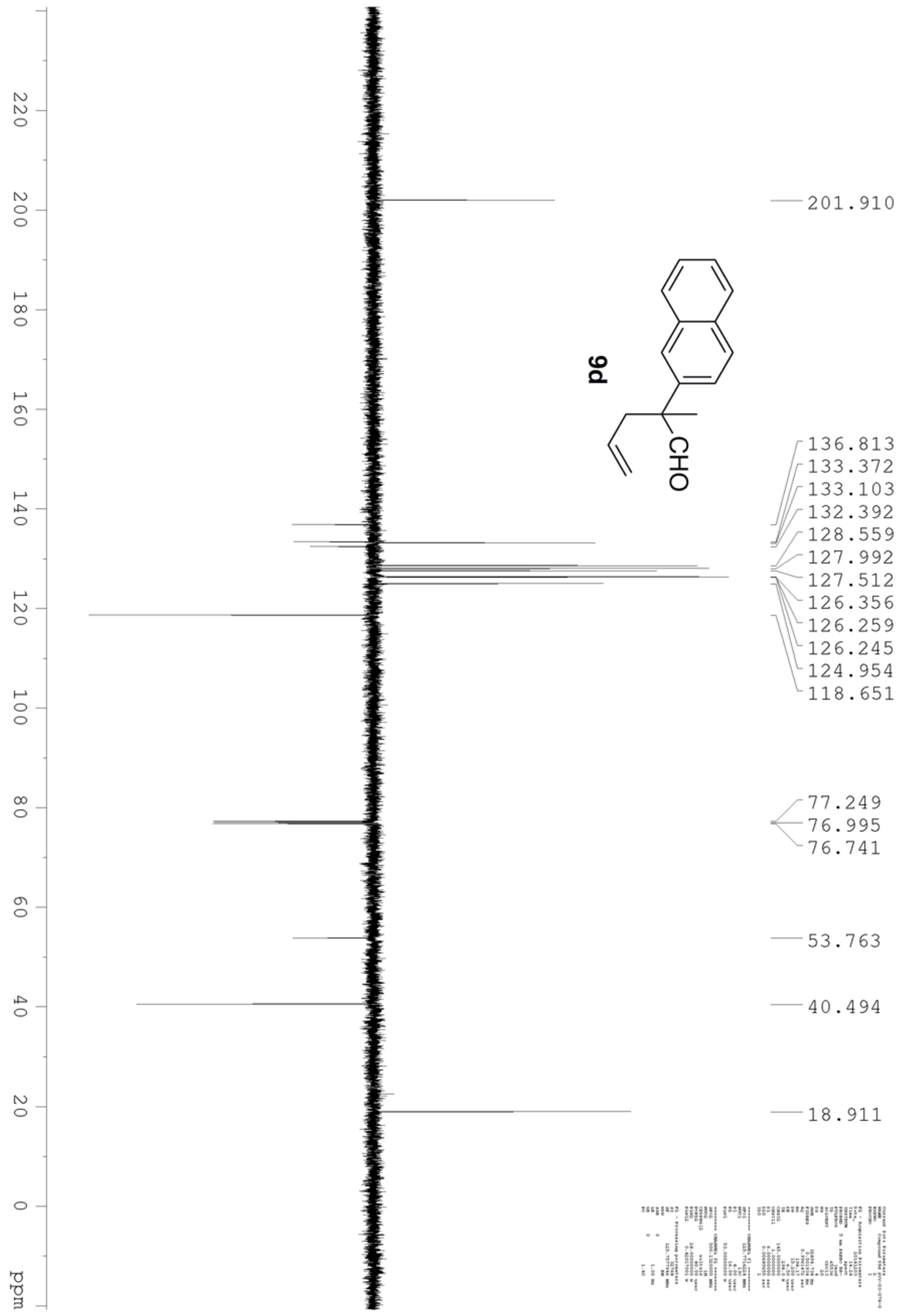
201.910
9d
CHO
136.813
133.372
133.103
132.392
128.559
127.992
127.512
126.356
126.259
126.245
124.954
118.651
77.249
76.995
76.741
53.763
40.494
18.911
ppm

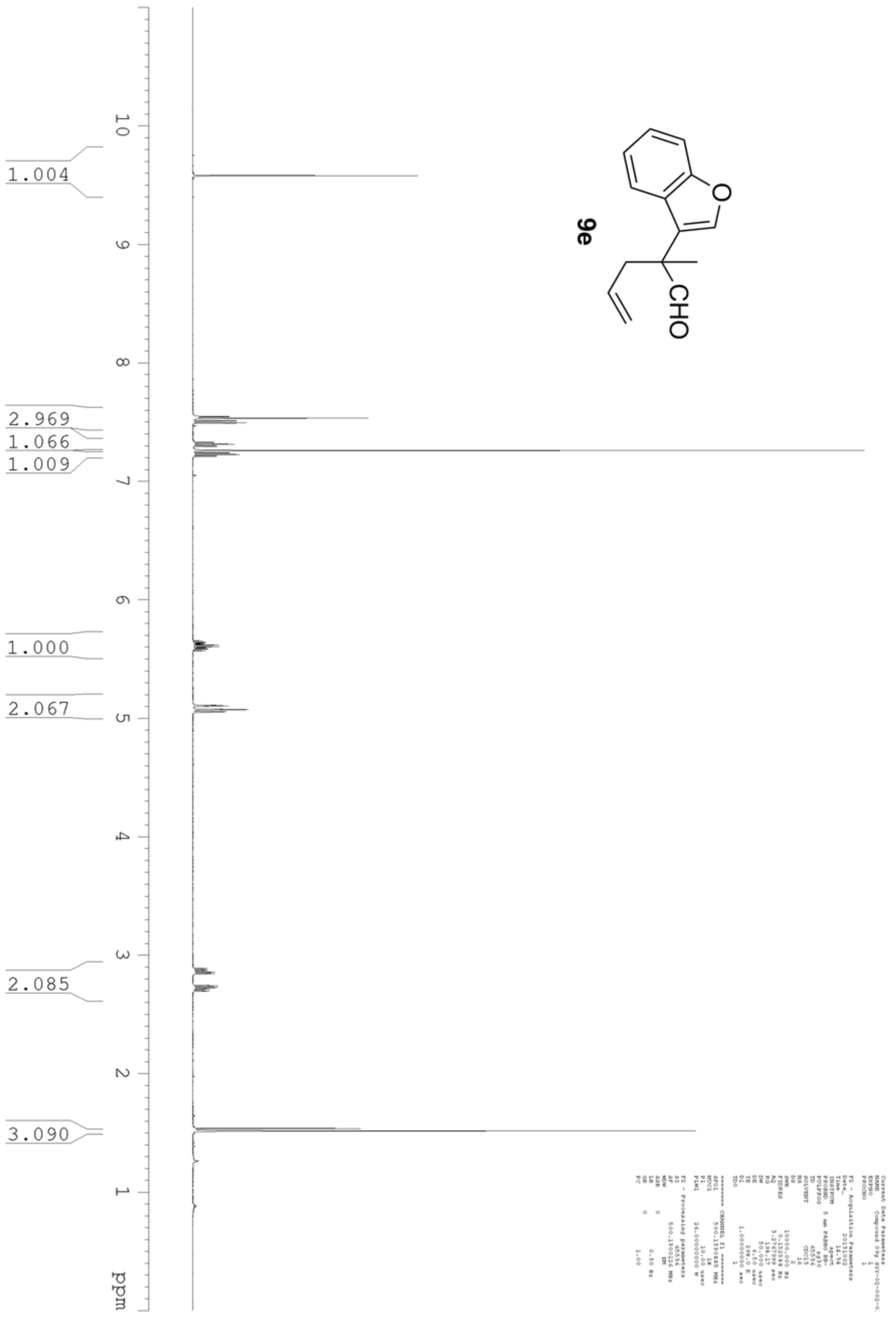

9e
O
CHO
1.004
2.969
1.066
1.009
1.000
2.067
2.085
3.090
10
9
8
7
6
5
4
3
2
1
ppm

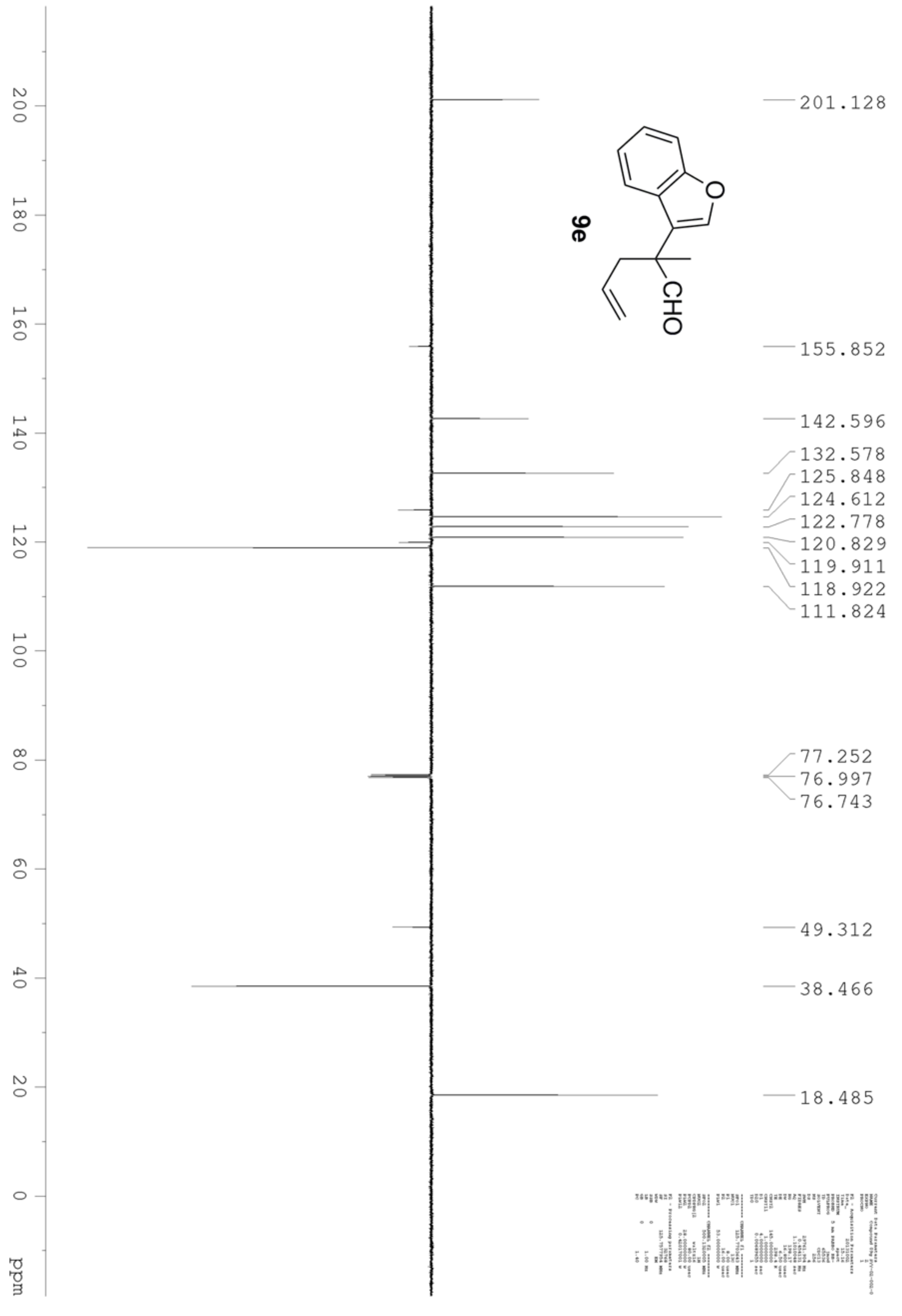
201.128
155.852
142.596
132.578
125.848
124.612
122.778
120.829
119.911
118.922
111.824
77.252
76.997
76.743
49.312
38.466
18.485
9e
CHO
O
ppm
200
180
160
140
120
100
80
60
40
20
0

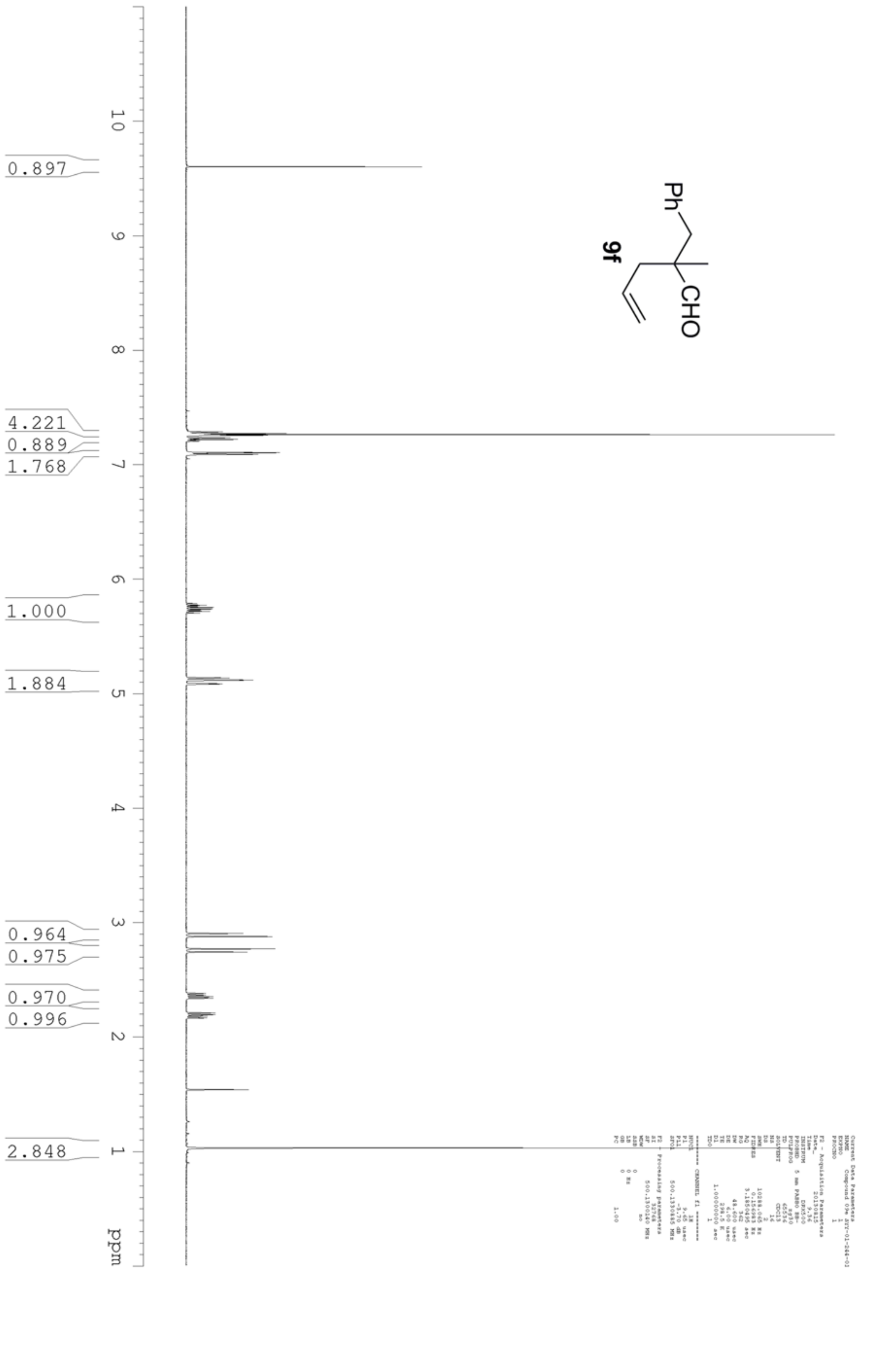

9f
CHO
Ph
0.897
4.221
0.889
1.768
1.000
1.884
0.964
0.975
0.970
0.996
2.848
10
9
8
7
6
5
4
3
2
1
ppm

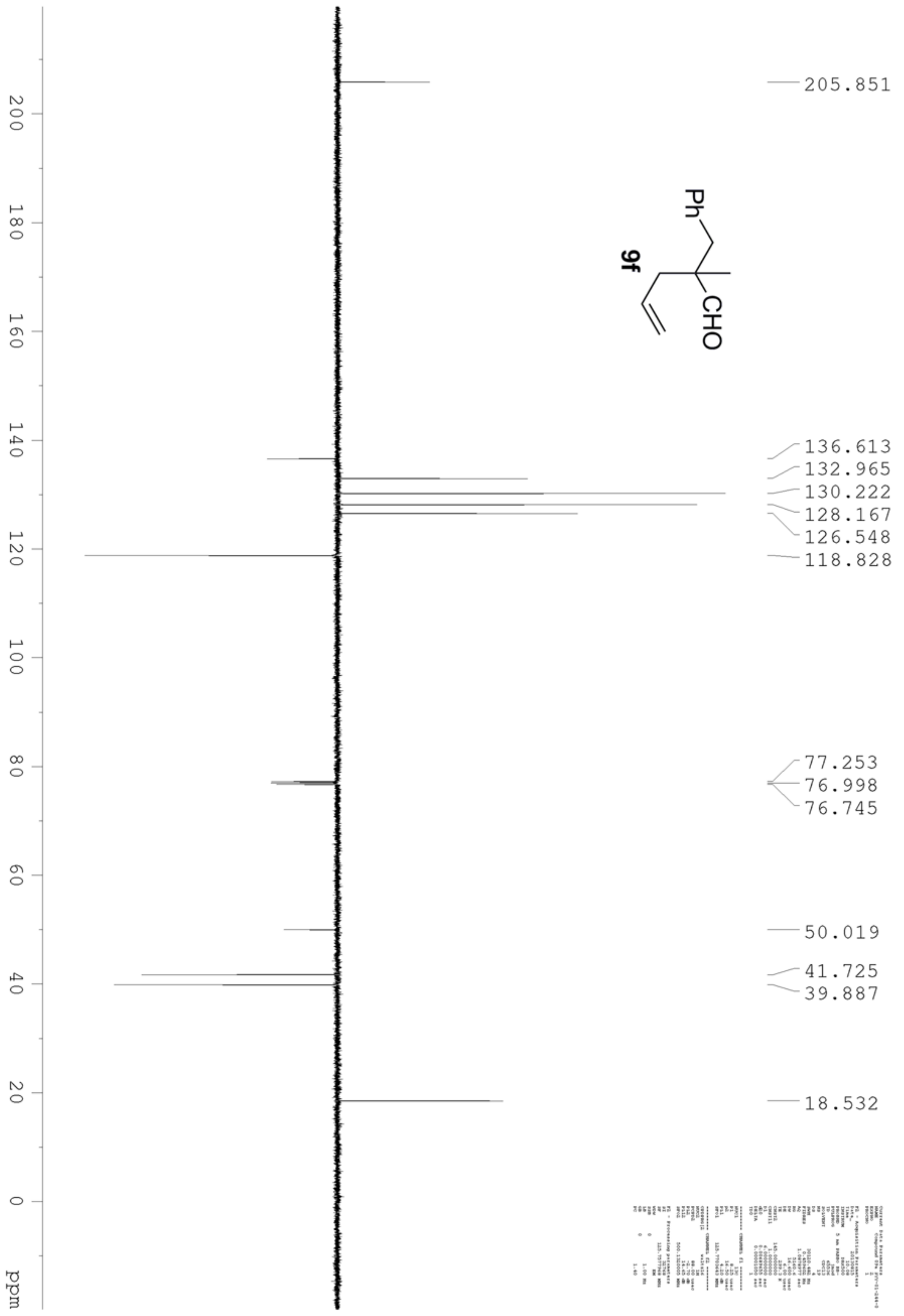

205.851
136.613
132.965
130.222
128.167
126.548
118.828
77.253
76.998
76.745
50.019
41.725
39.887
18.532
Ph
CHO
9f
ppm

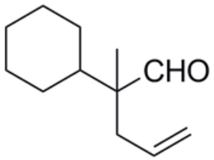

9g

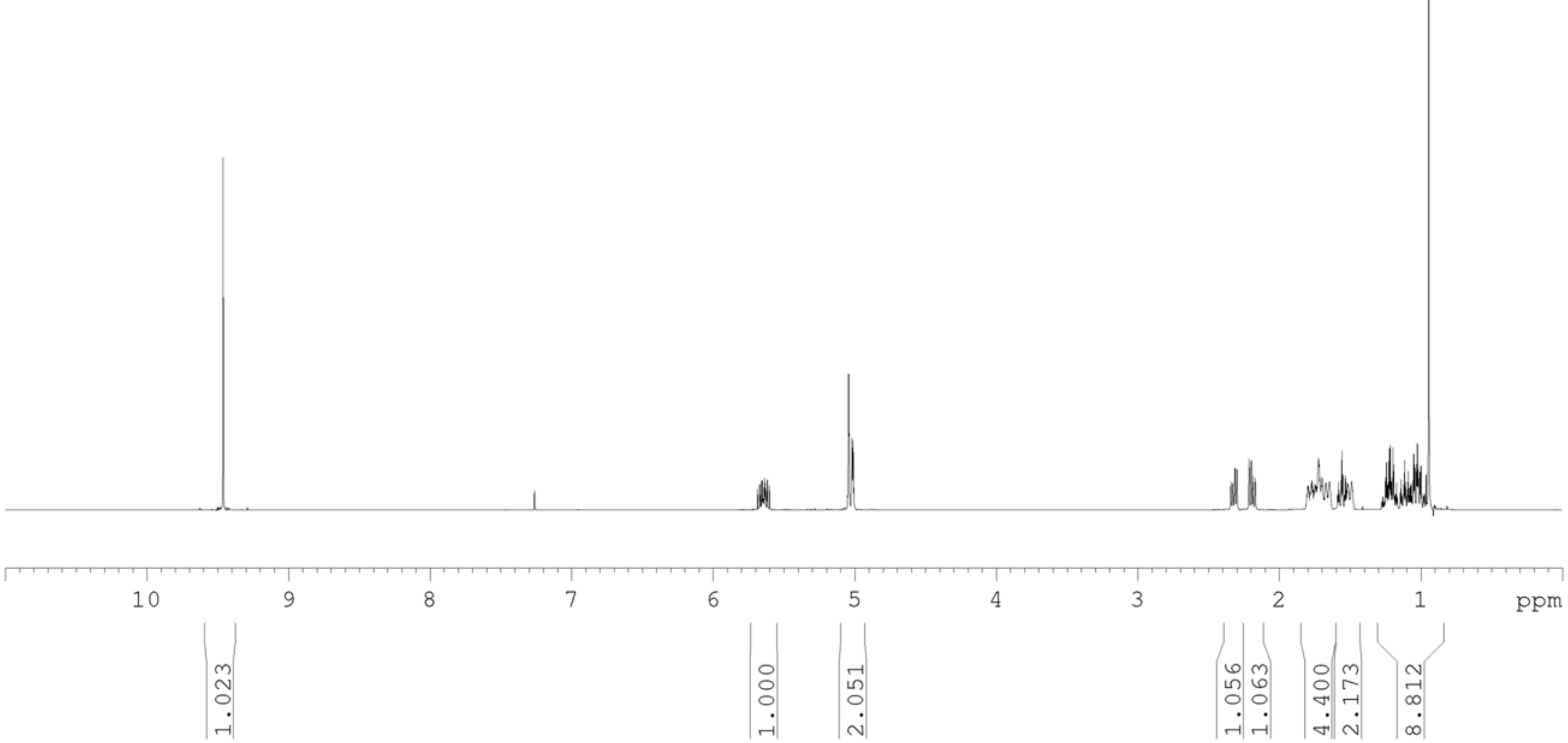

Current Data Parameters
NAME Compound 09f SYY-01-243-01
EXPNO 1
PROCNO 1
F2 - Acquisition Parameters
Date_ 20141203
Time 13.39
INSTRUM spect
PROBHD 5 mm PABBO BB-
PULPROG zg30
TD 65536
SOLVENT CDCl3
NS 6
DS 2
SWH 10000.000 Hz
FIDRES 0.152588 Hz
AQ 3.2767999 sec
RG 30.81
DW 50.000 usec
DE 6.50 usec
TE 298.0 K
D1 1.00000000 sec
TD0 1
======== CHANNEL f1 ========
SFO1 500.1330885 MHz
NUC1 1H
P1 10.00 usec
PLW1 26.00000000 W
F2 - Processing parameters
SI 65536
SF 500.1300117 MHz
WDW EM
SSB 0
LB 0.30 Hz
GB 0
PC 1.00
10
9
8
7
6
5
4
3
2
1
ppm
1.023
1.000
2.051
1.056
1.063
4.400
2.173
8.812

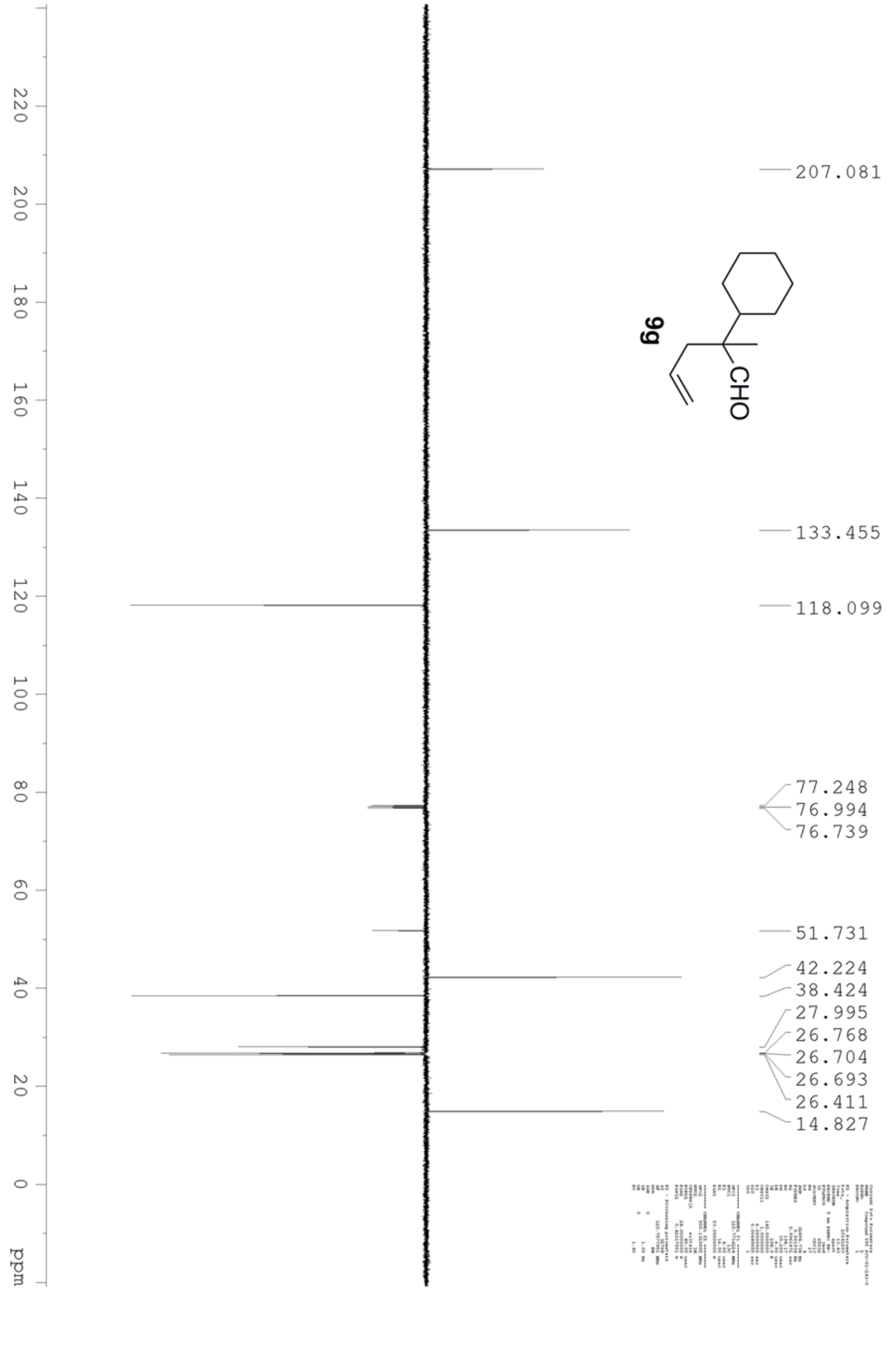

207.081
133.455
118.099
77.248
76.994
76.739
51.731
42.224
38.424
27.995
26.768
26.704
26.693
26.411
14.827
9g
CHO
ppm

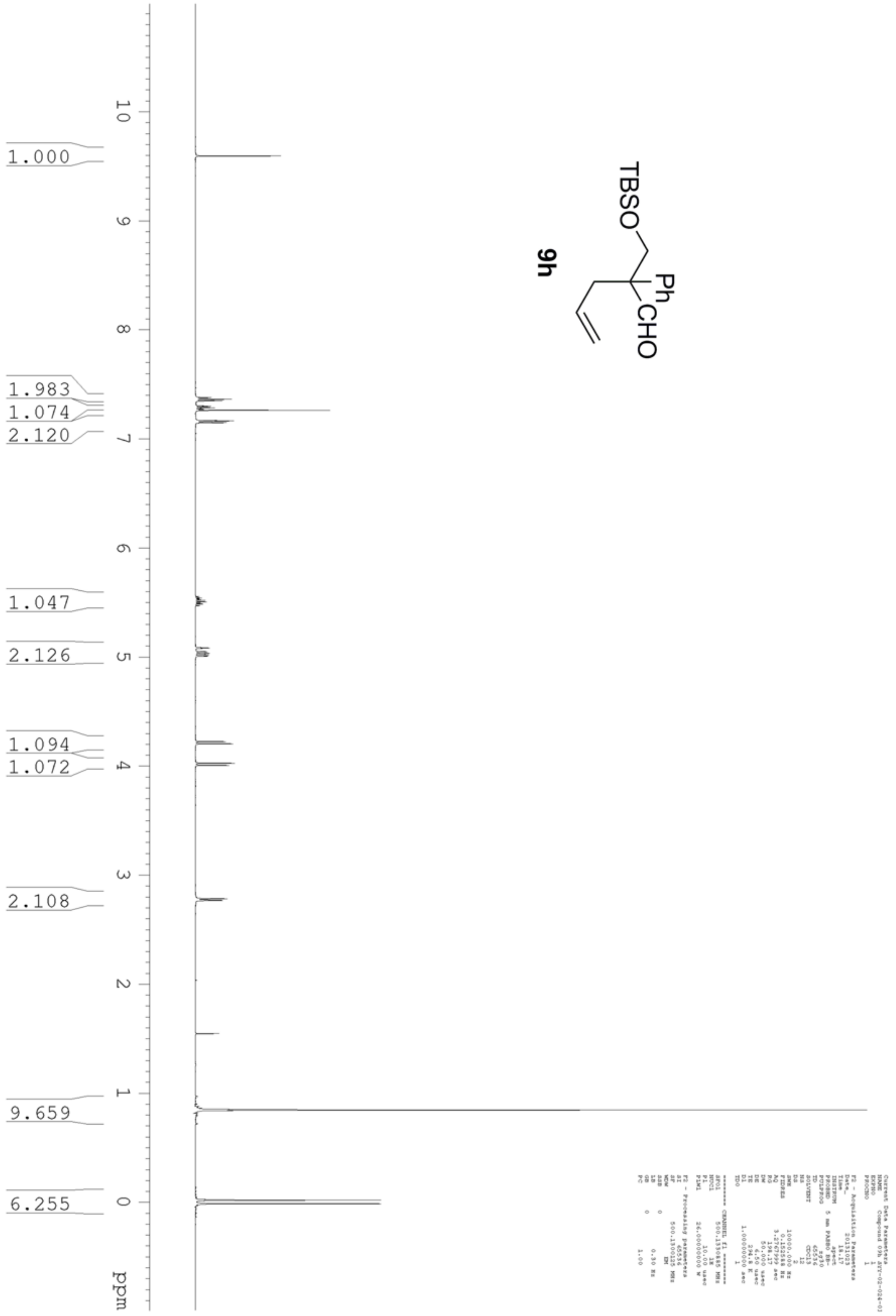

TBSO
Ph
CHO
9h
1.000
1.983
1.074
2.120
1.047
2.126
1.094
1.072
2.108
9.659
6.255
10
9
8
7
6
5
4
3
2
1
0
ppm

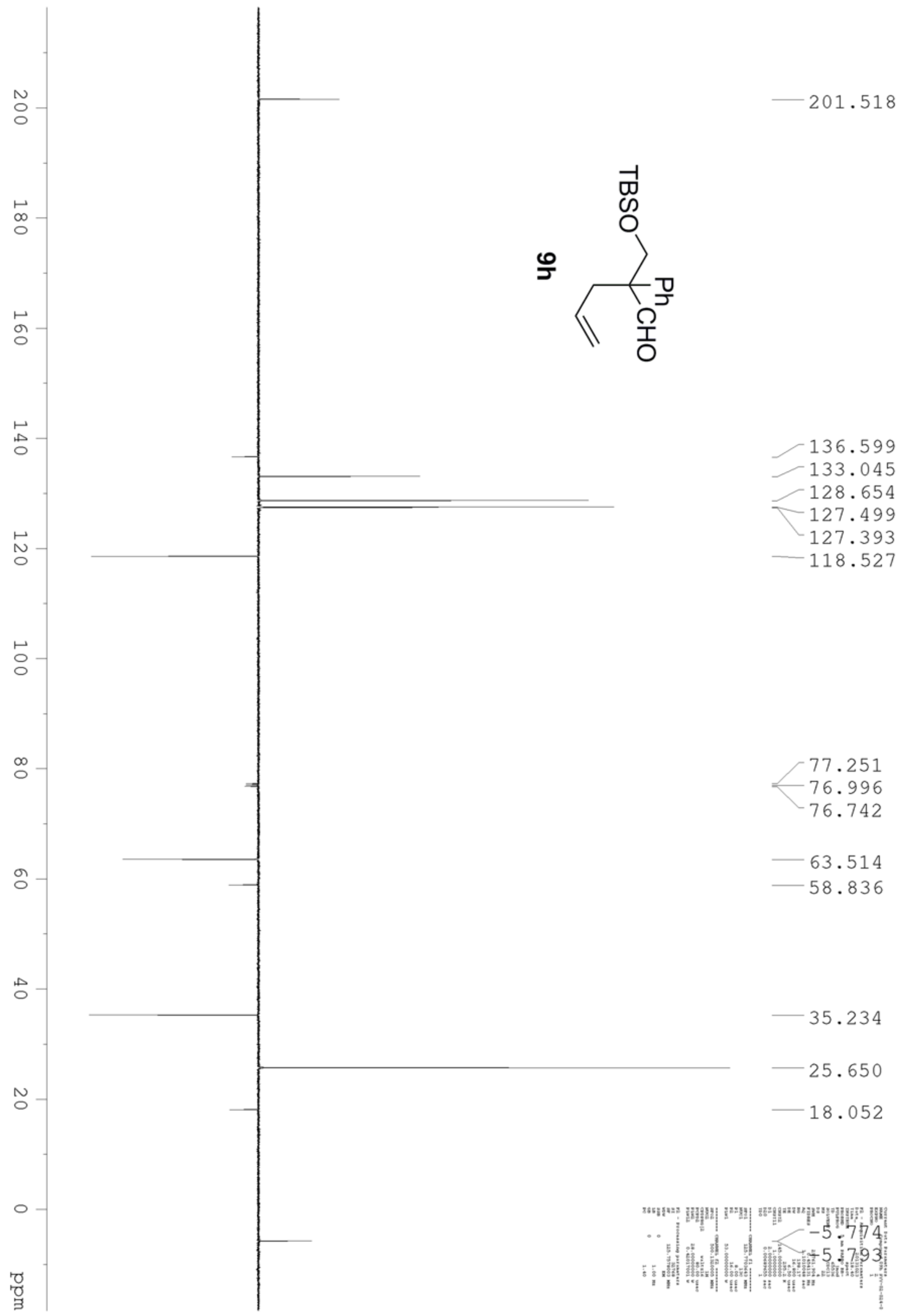
201.518
TBSO
Ph
CHO
9h
136.599
133.045
128.654
127.499
127.393
118.527
77.251
76.996
76.742
63.514
58.836
35.234
25.650
18.052
-5.774
-5.793
ppm

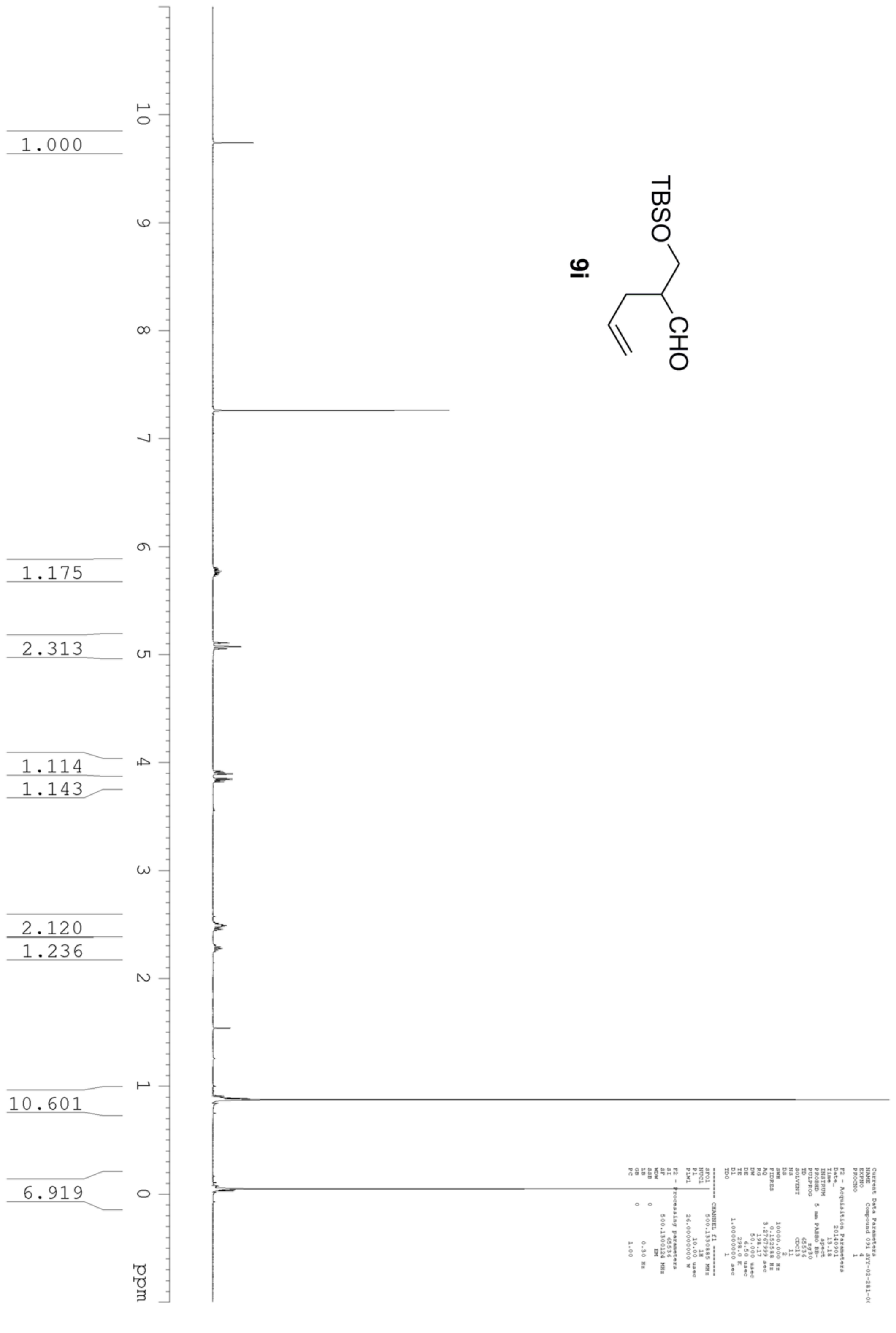
TBSO
CHO
9i
1.000
1.175
2.313
1.114
1.143
2.120
1.236
10.601
6.919
ppm

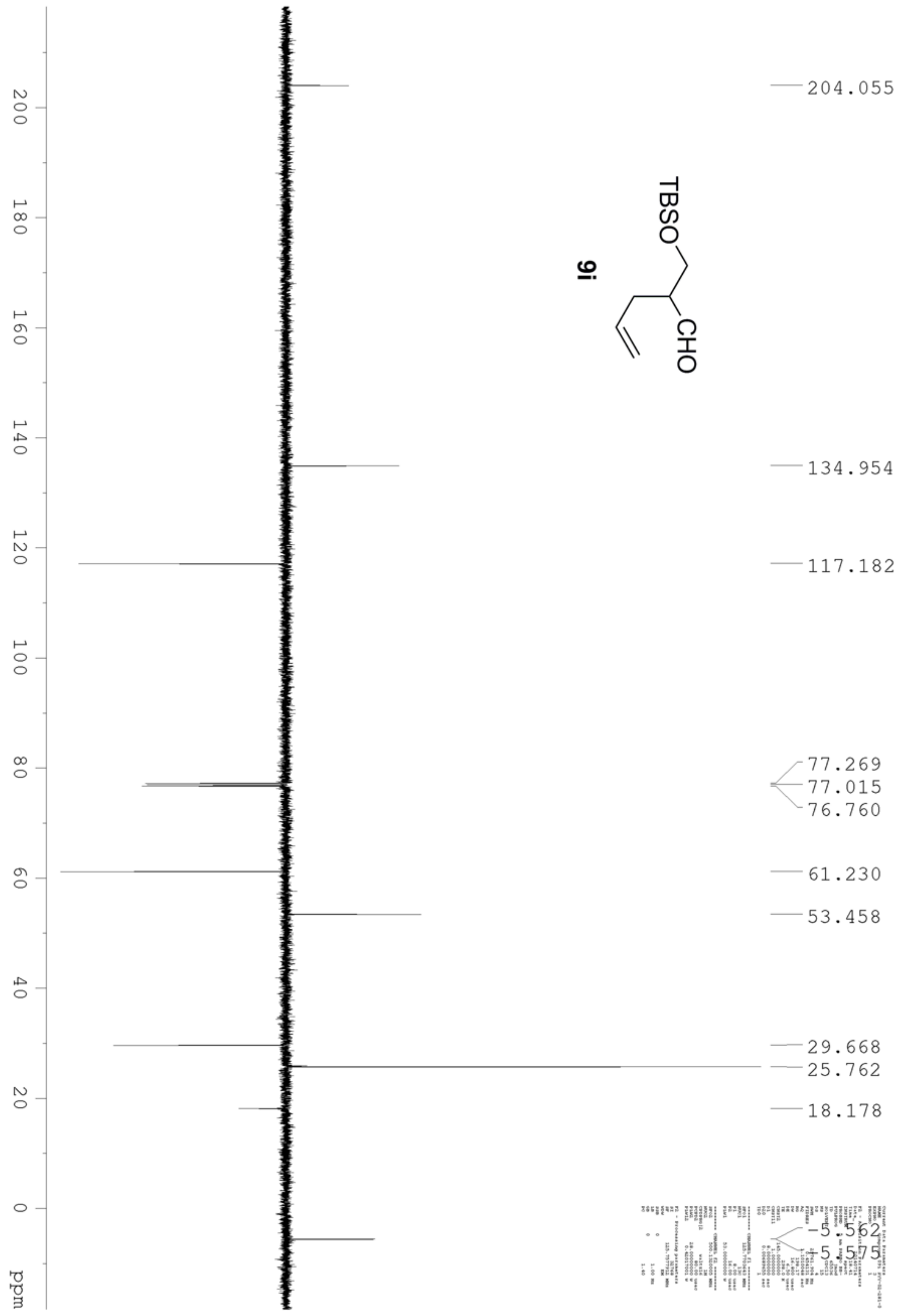

204.055
134.954
117.182
77.269
77.015
76.760
61.230
53.458
29.668
25.762
18.178
-5.562
-5.575
TBSO
CHO
9i
ppm

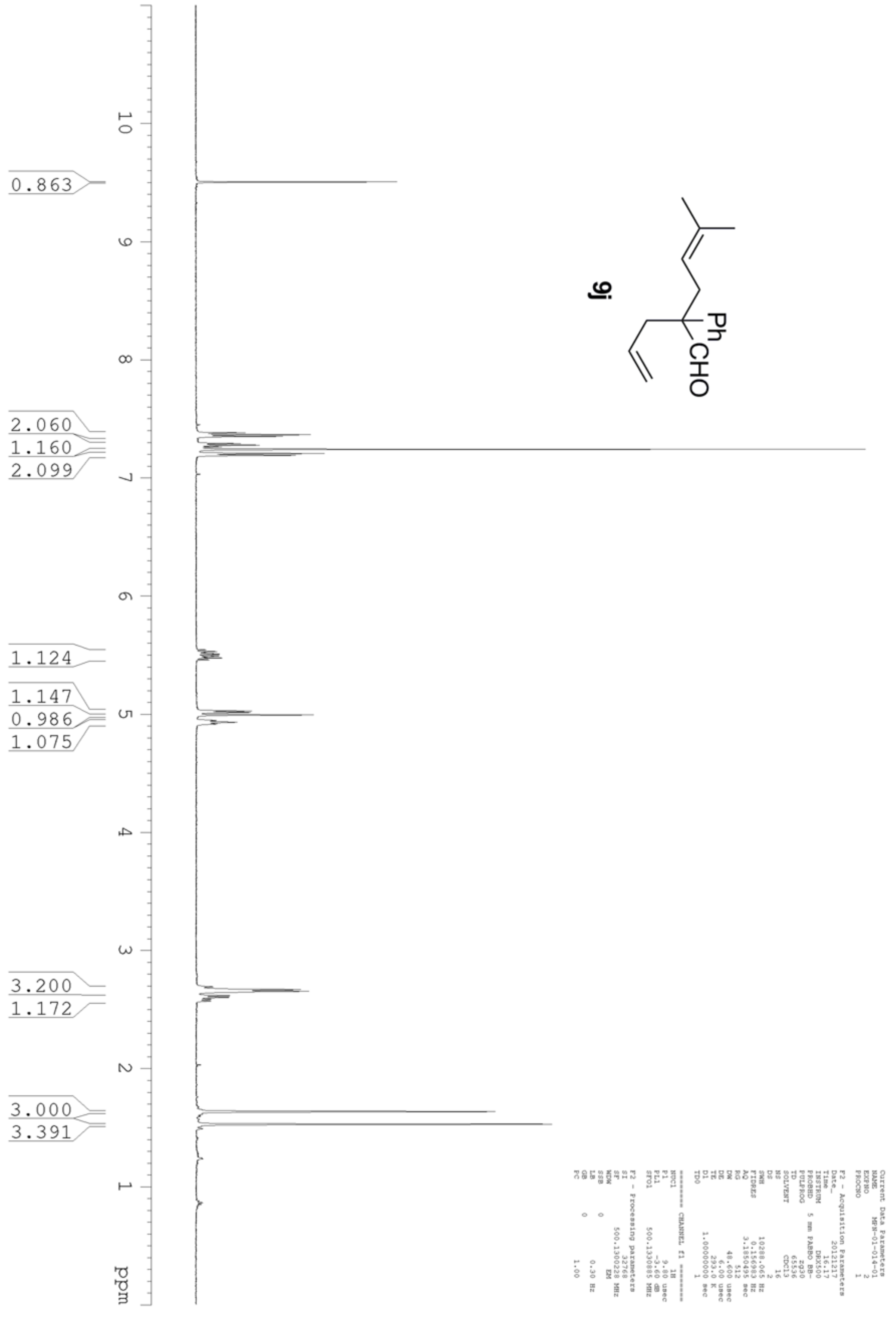

Ph
CHO
9j
0.863
2.060
1.160
2.099
1.124
1.147
0.986
1.075
3.200
1.172
3.000
3.391
10
9
8
7
6
5
4
3
2
1
ppm

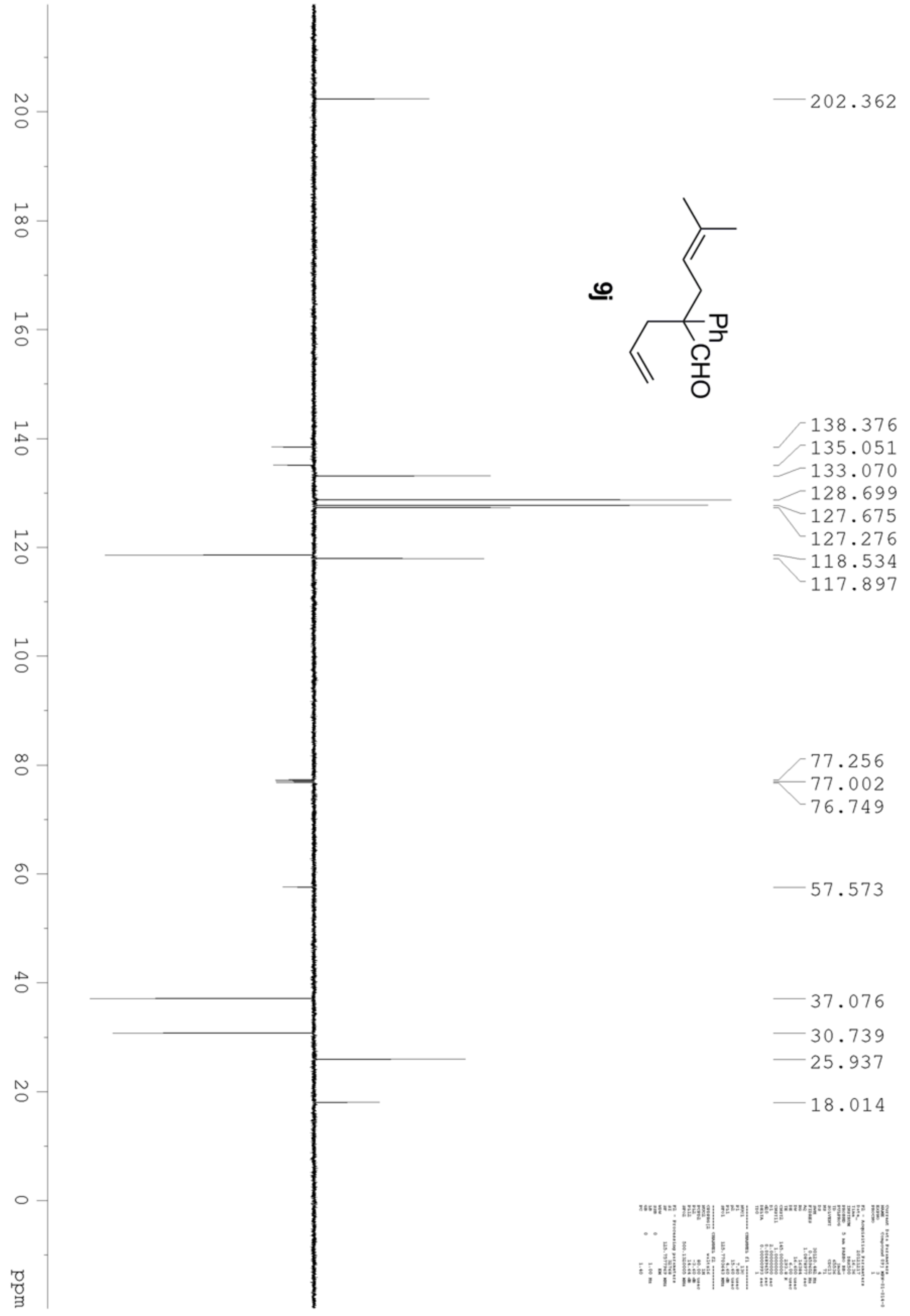
202.362
9j
Ph
CHO
138.376
135.051
133.070
128.699
127.675
127.276
118.534
117.897
77.256
77.002
76.749
57.573
37.076
30.739
25.937
18.014
200
180
160
140
120
100
80
60
40
20
0
ppm

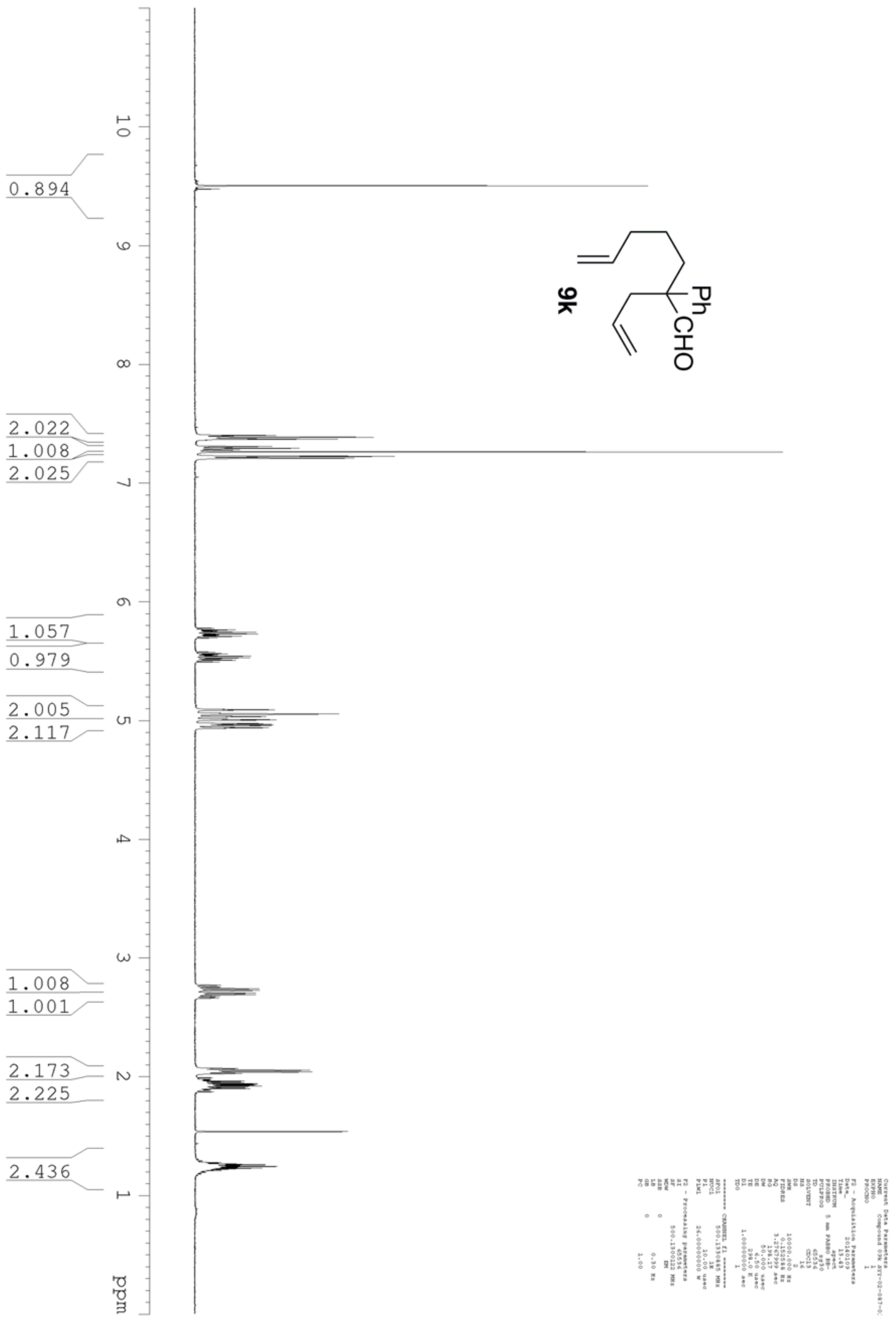

Ph
CHO
9k
ppm

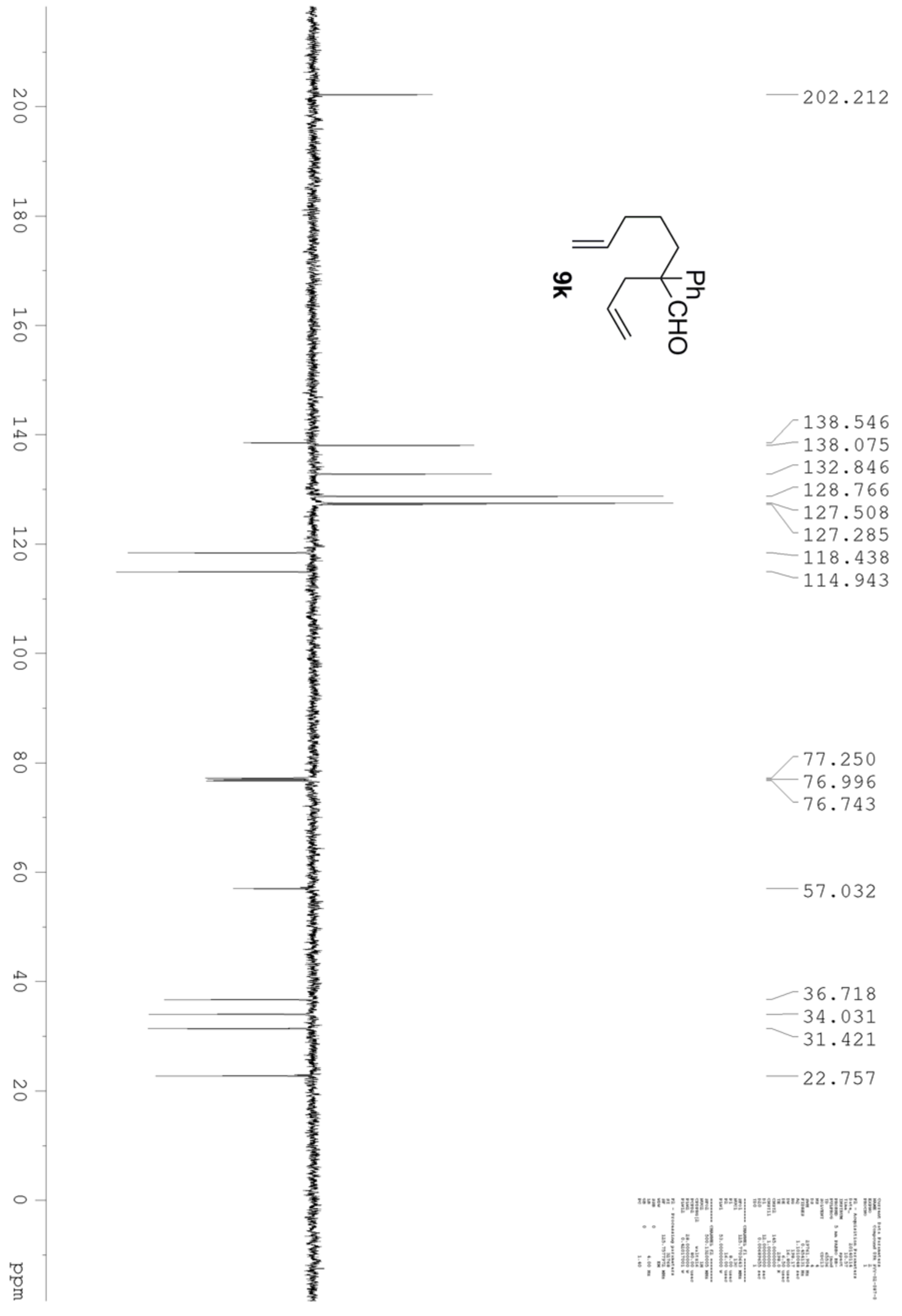

202.212
138.546
138.075
132.846
128.766
127.508
127.285
118.438
114.943
77.250
76.996
76.743
57.032
36.718
34.031
31.421
22.757
Ph
CHO
9k
ppm

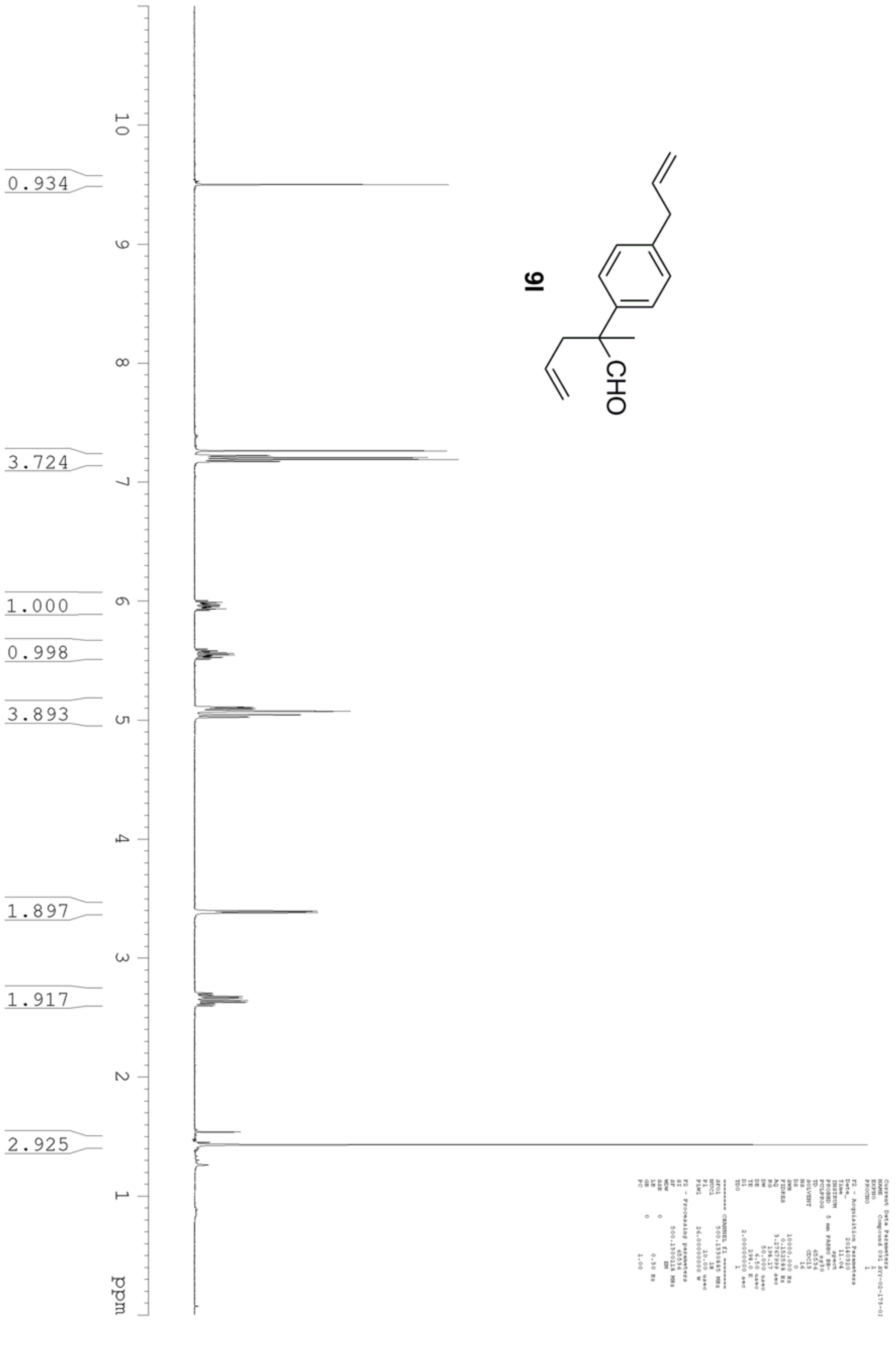
9l
CHO
0.934
3.724
1.000
0.998
3.893
1.897
1.917
2.925
10
9
8
7
6
5
4
3
2
1
ppm

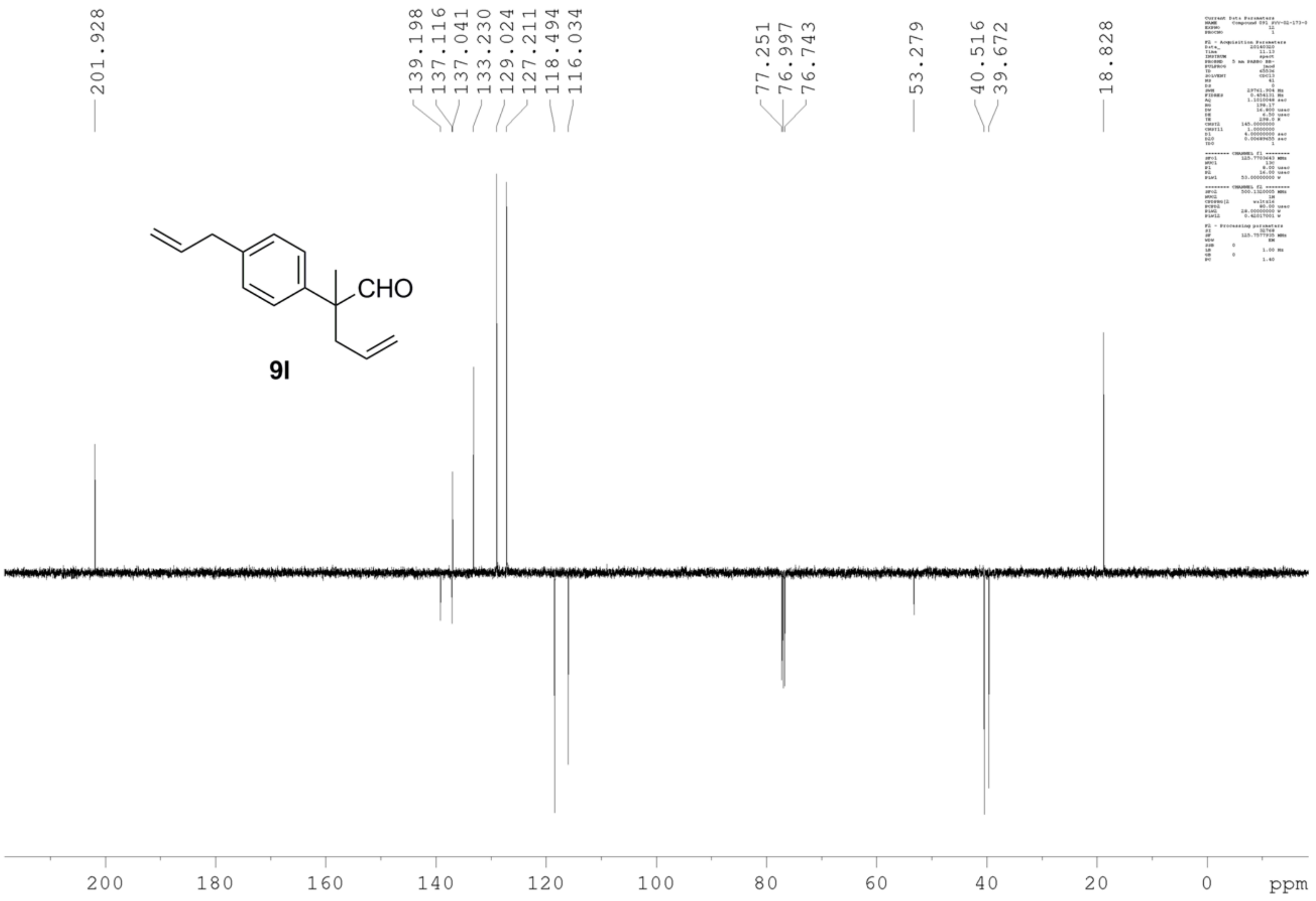
201.928
139.198
137.116
137.041
133.230
129.024
127.211
118.494
116.034
77.251
76.997
76.743
53.279
40.516
39.672
18.828
CHO
9l
200
180
160
140
120
100
80
60
40
20
0
ppm

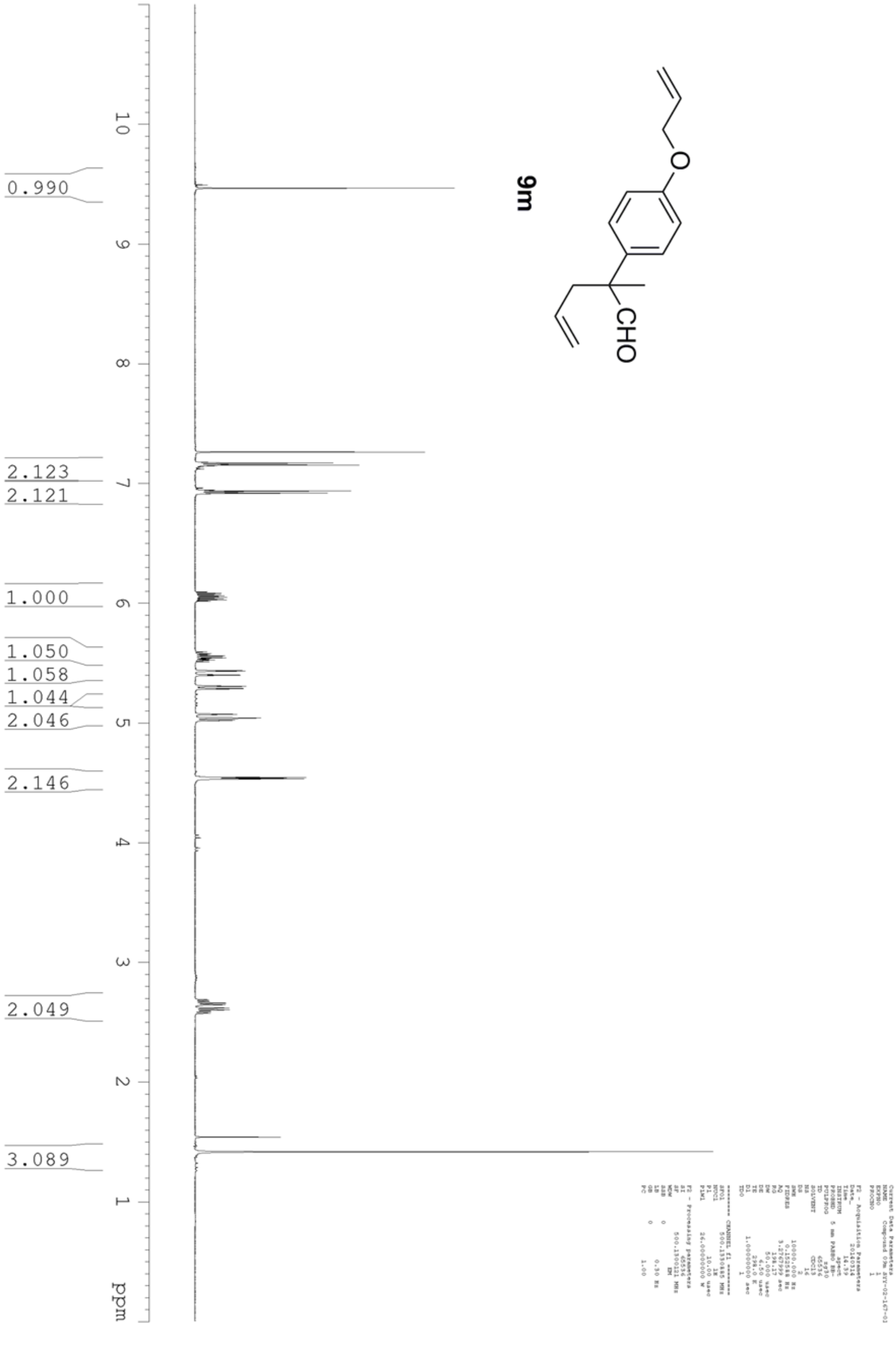
9m
CHO
O
0.990
2.123
2.121
1.000
1.050
1.058
1.044
2.046
2.146
2.049
3.089
10
9
8
7
6
5
4
3
2
1
ppm

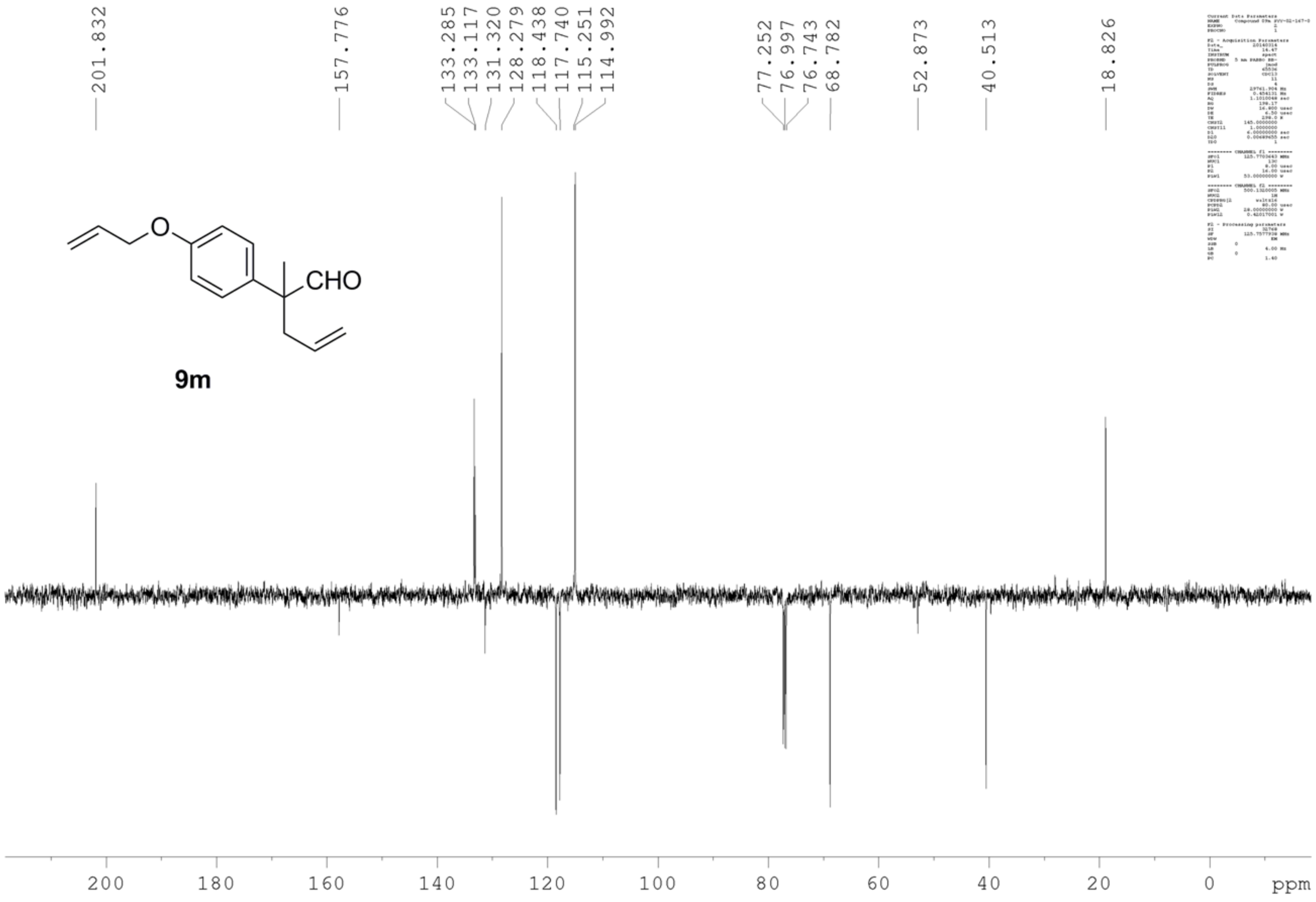
201.832
157.776
133.285
133.117
131.320
128.279
118.438
117.740
115.251
114.992
77.252
76.997
76.743
68.782
52.873
40.513
18.826
CHO
O
9m
200
180
160
140
120
100
80
60
40
20
0
ppm

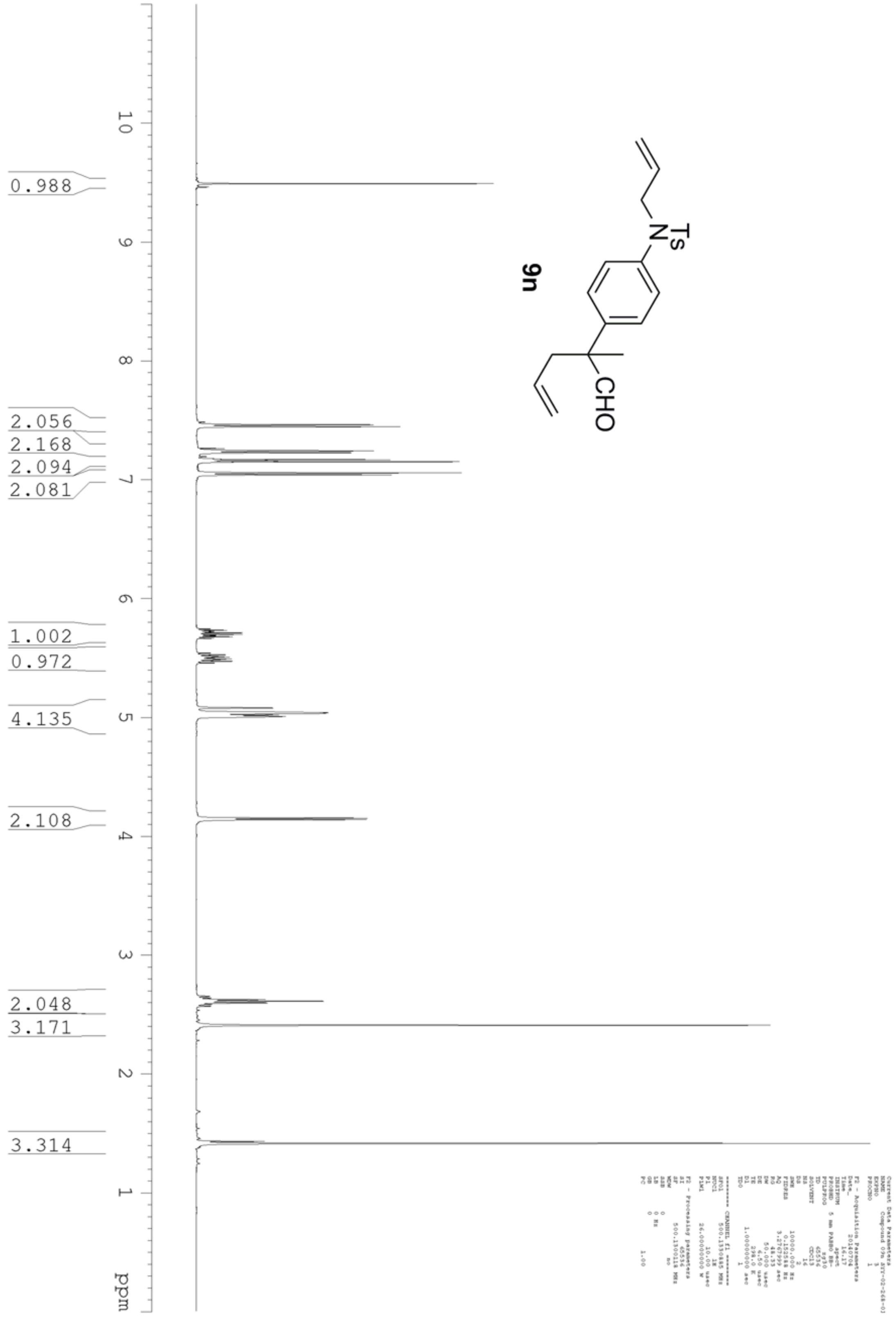
9n
Ts
N
CHO
0.988
2.056
2.168
2.094
2.081
1.002
0.972
4.135
2.108
2.048
3.171
3.314
ppm

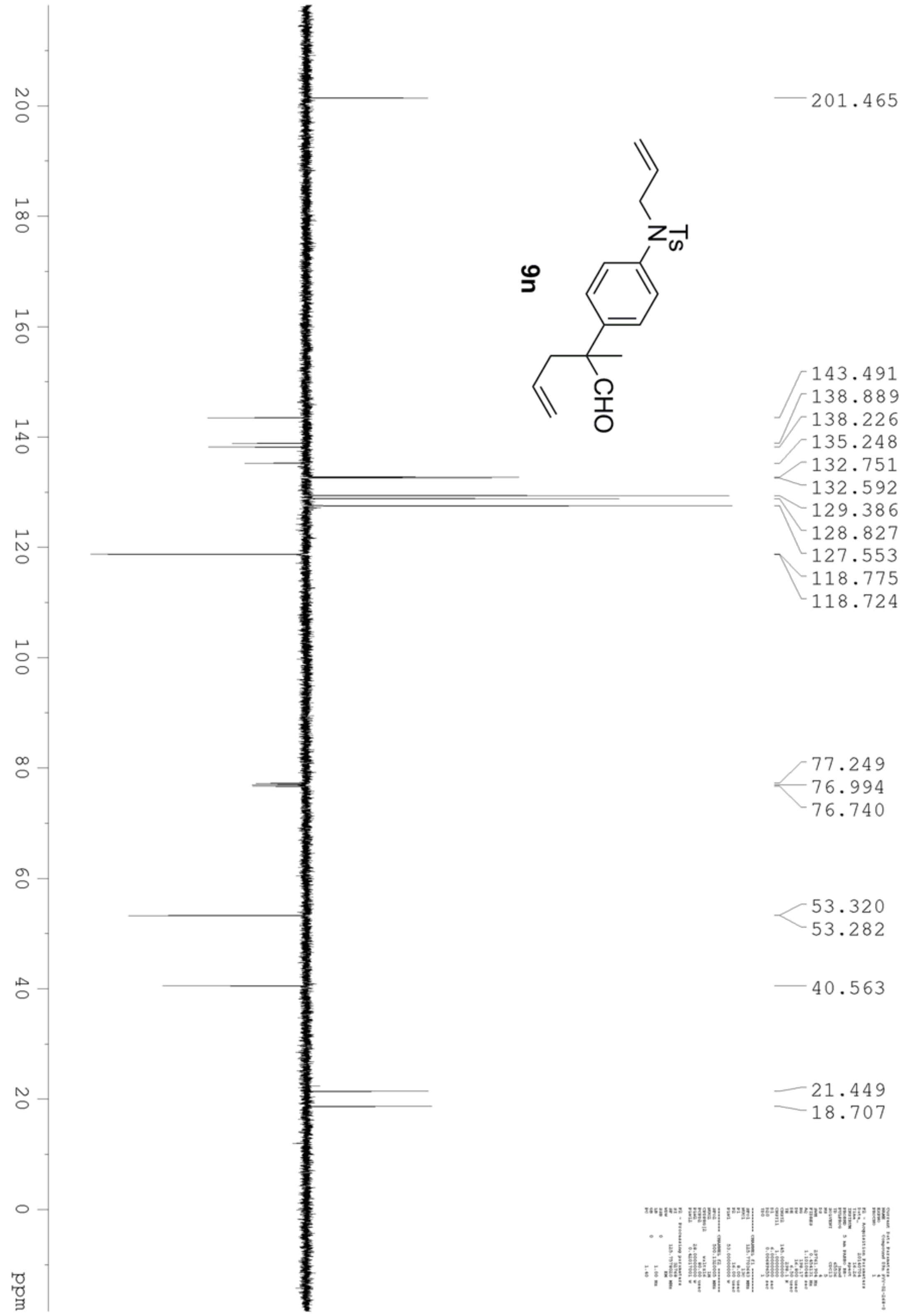
201.465
143.491
138.889
138.226
135.248
132.751
132.592
129.386
128.827
127.553
118.775
118.724
77.249
76.994
76.740
53.320
53.282
40.563
21.449
18.707
9n
Ts
N
CHO
200
180
160
140
120
100
80
60
40
20
0
ppm

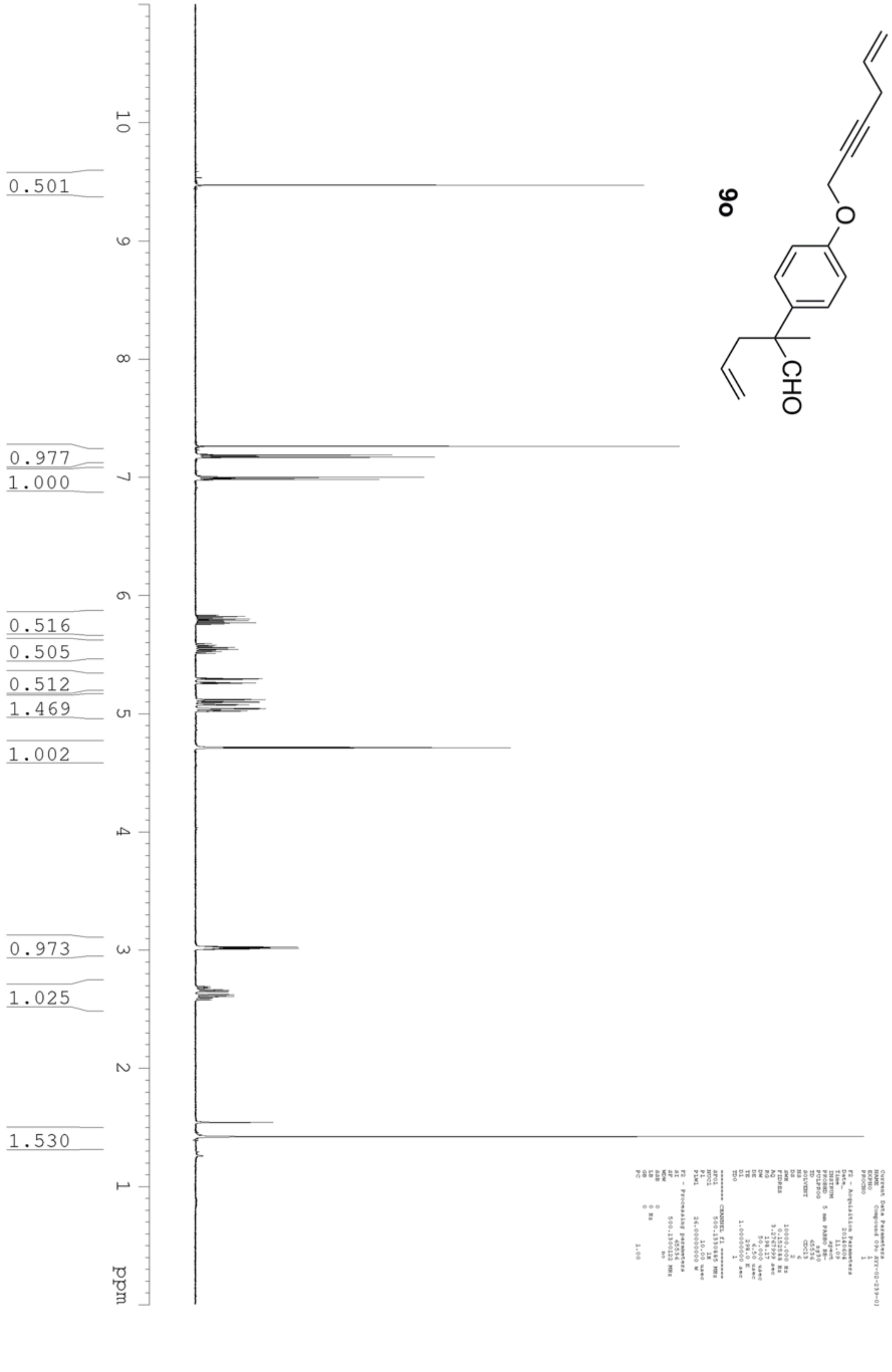
9o
CHO
O
0.501
0.977
1.000
0.516
0.505
0.512
1.469
1.002
0.973
1.025
1.530
10
9
8
7
6
5
4
3
2
1
ppm

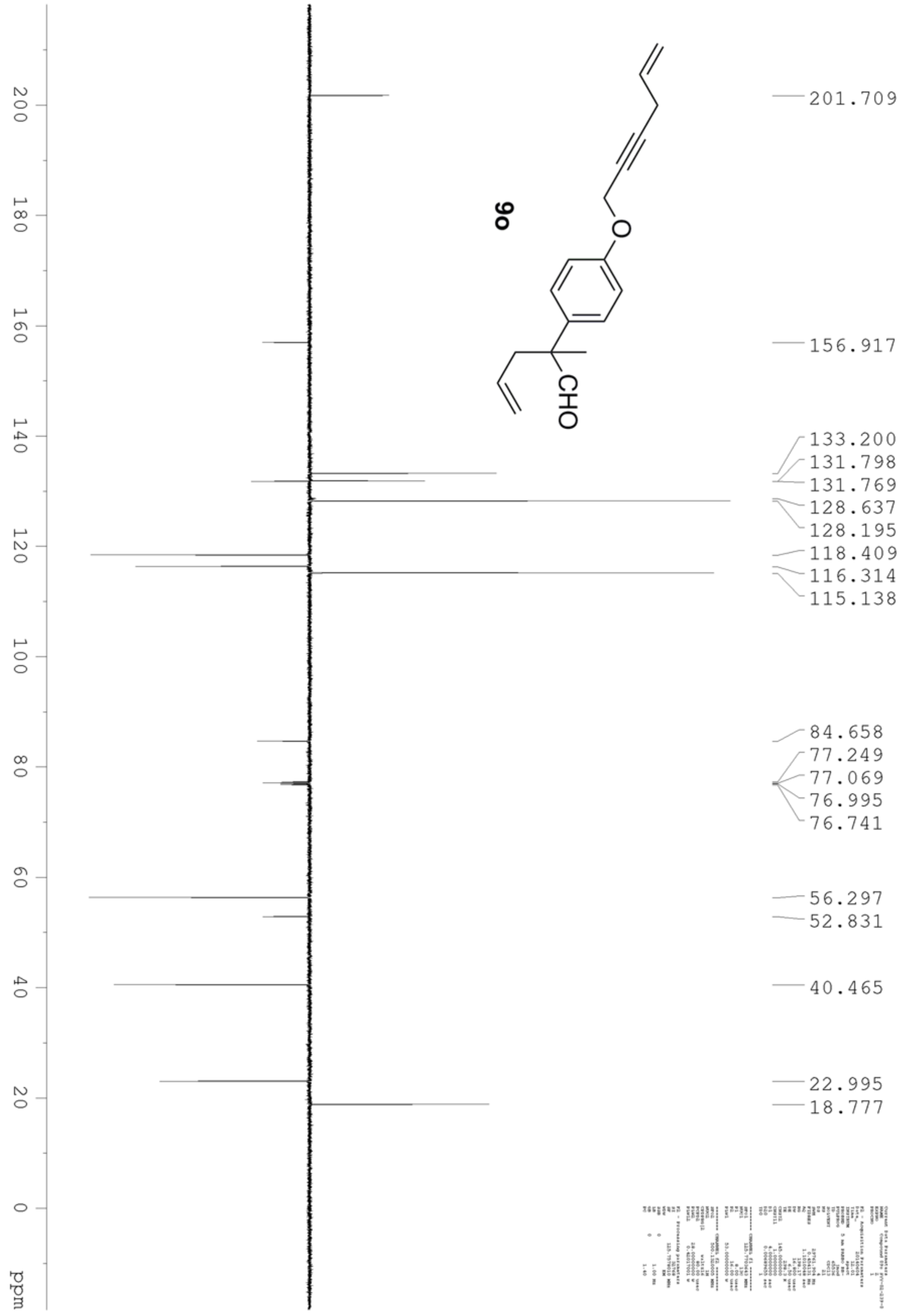
9o
O
CHO
201.709
156.917
133.200
131.798
131.769
128.637
128.195
118.409
116.314
115.138
84.658
77.249
77.069
76.995
76.741
56.297
52.831
40.465
22.995
18.777
200
180
160
140
120
100
80
60
40
20
0
ppm

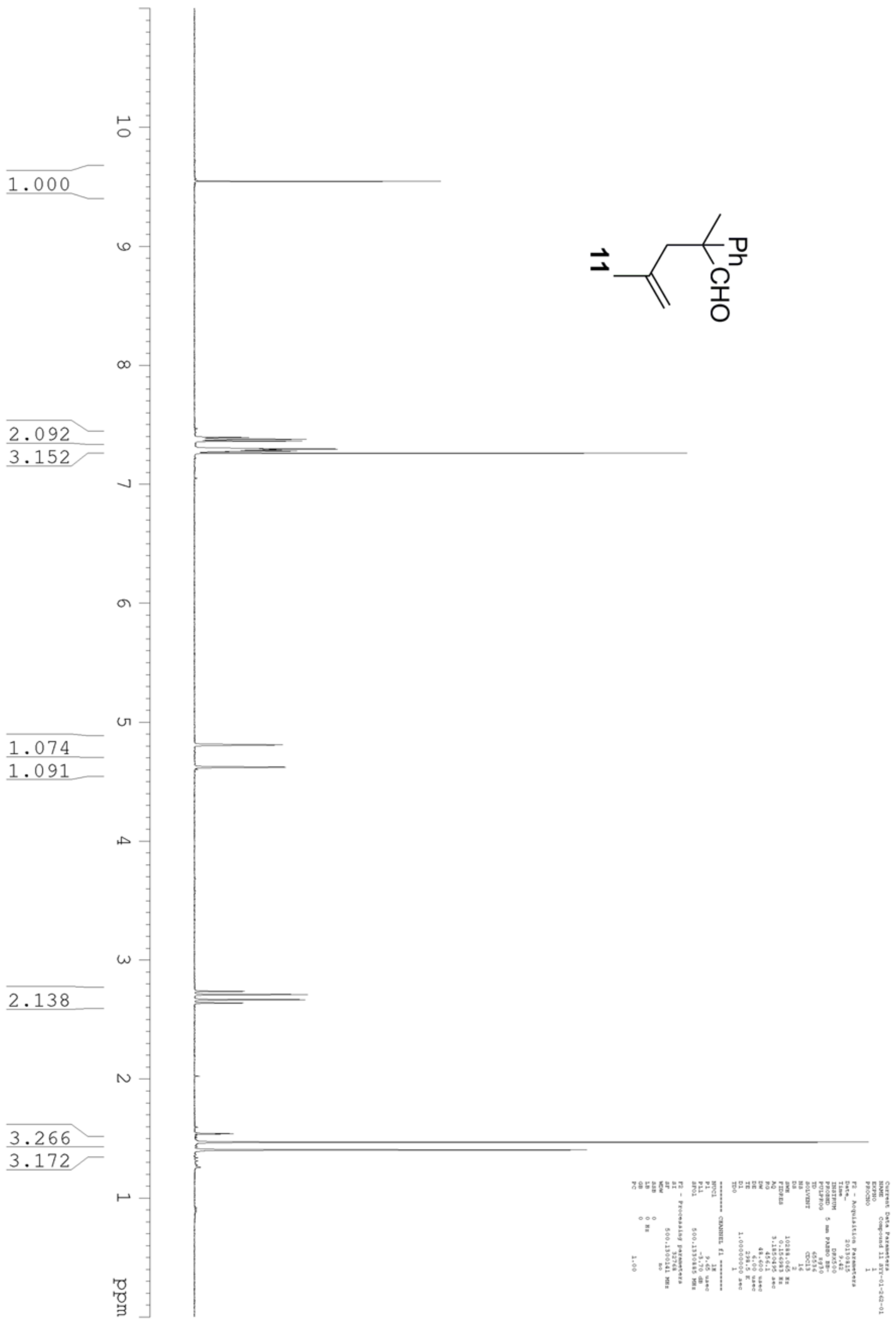

Ph
CHO
11
1.000
2.092
3.152
1.074
1.091
2.138
3.266
3.172
10
9
8
7
6
5
4
3
2
1
ppm

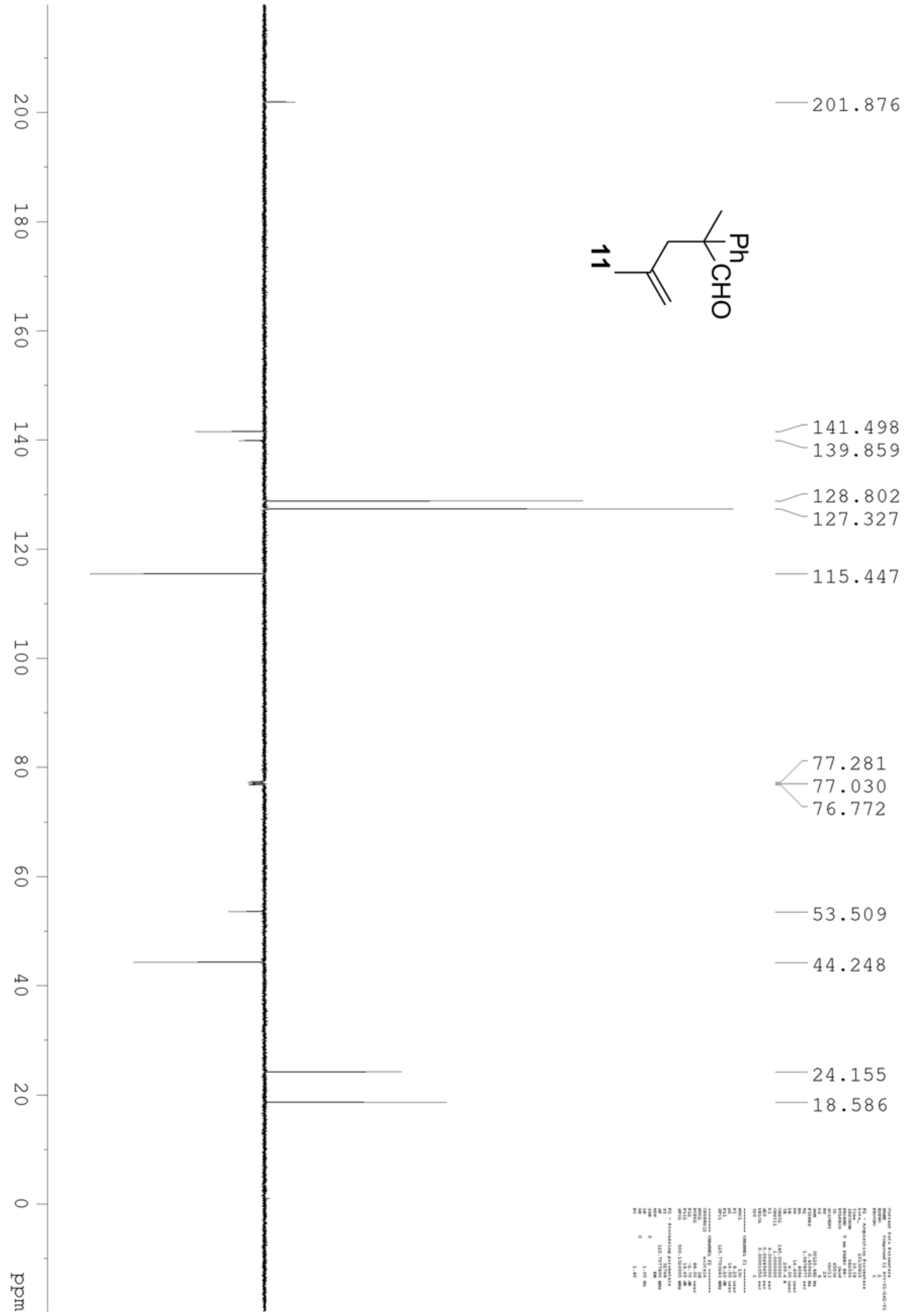
201.876
141.498
139.859
128.802
127.327
115.447
77.281
77.030
76.772
53.509
44.248
24.155
18.586
Ph
CHO
11
ppm

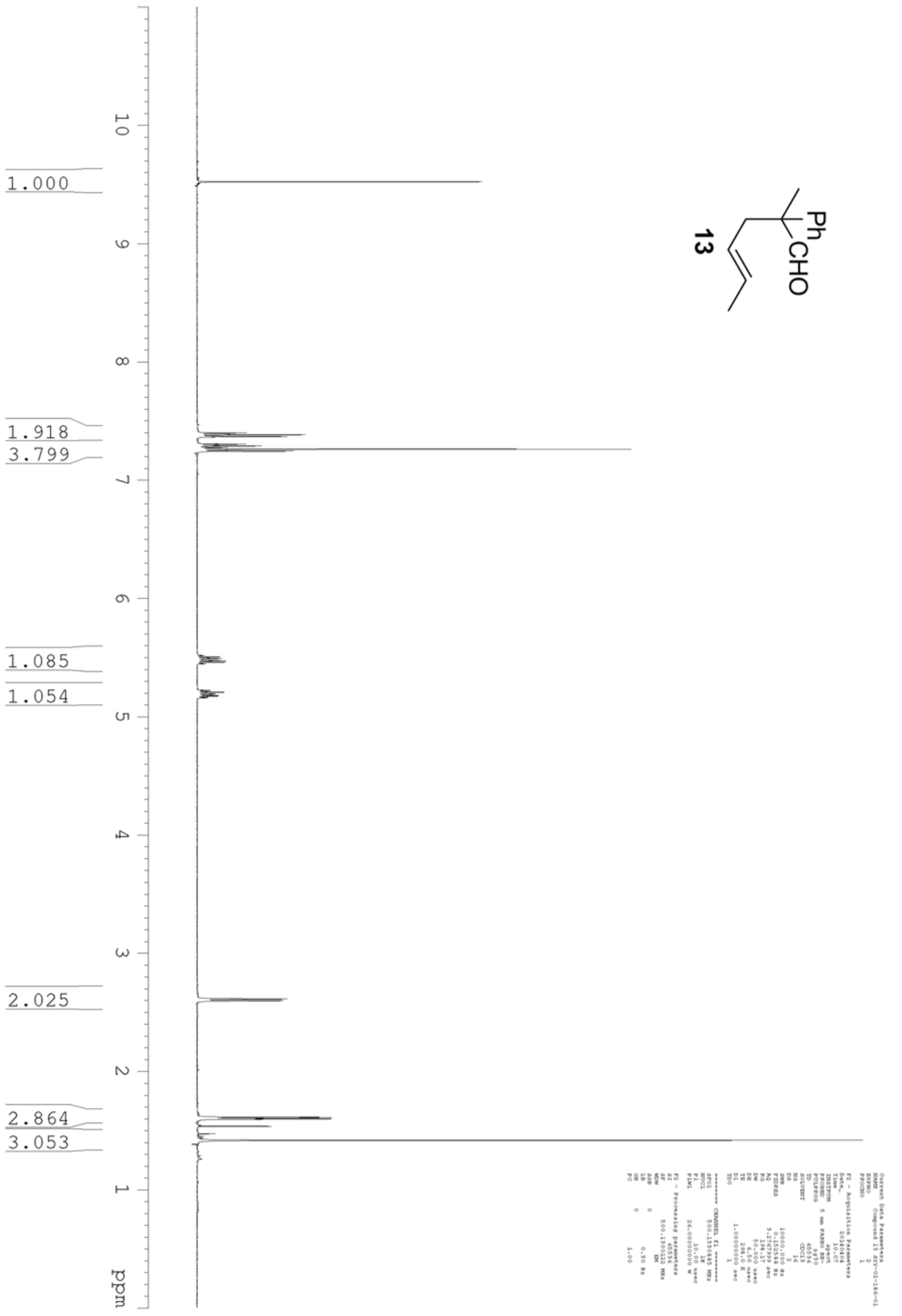

Ph
CHO
13
1.000
1.918
3.799
1.085
1.054
2.025
2.864
3.053
10
9
8
7
6
5
4
3
2
1
ppm

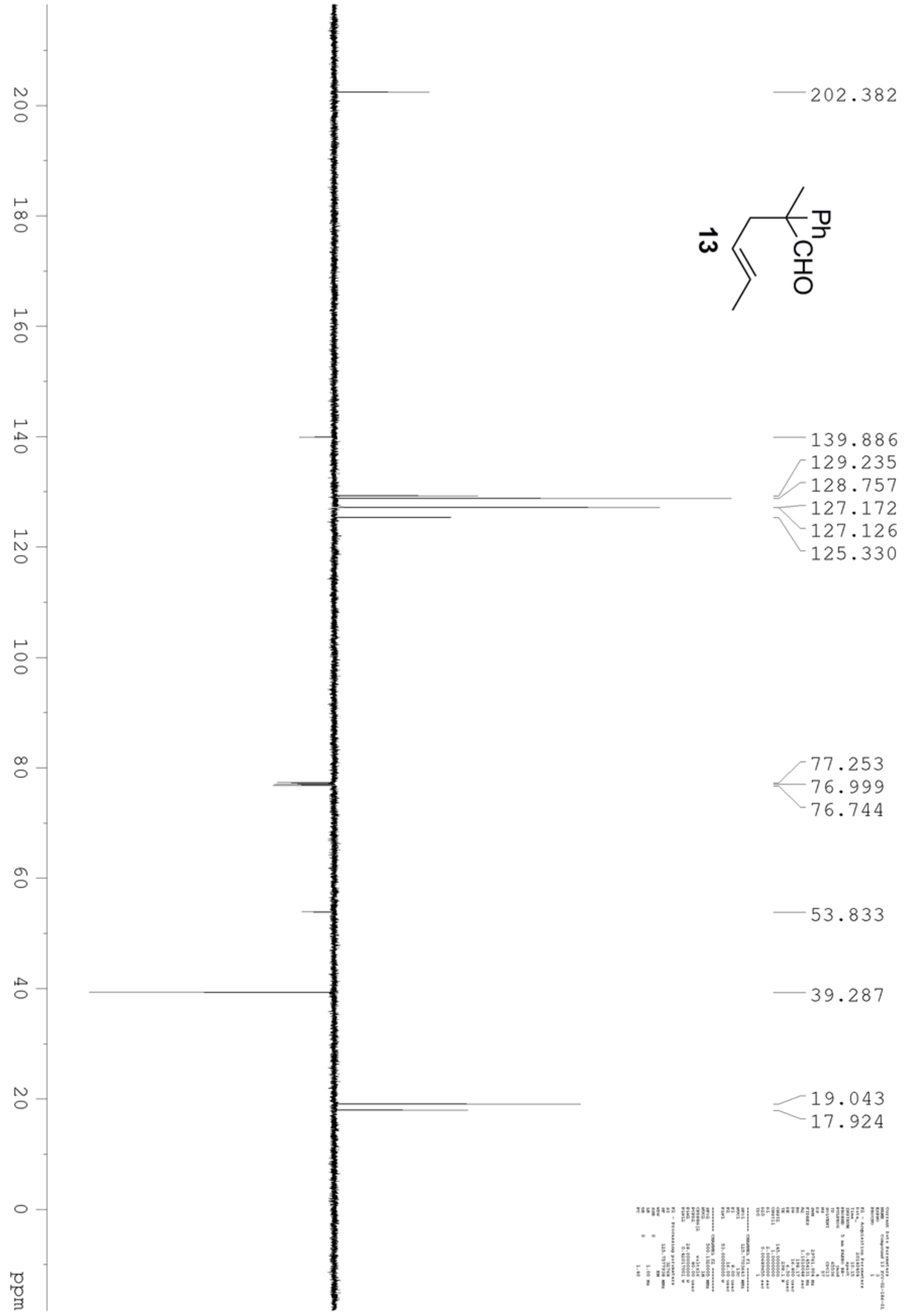
202.382
139.886
129.235
128.757
127.172
127.126
125.330
77.253
76.999
76.744
53.833
39.287
19.043
17.924
Ph
CHO
13
ppm

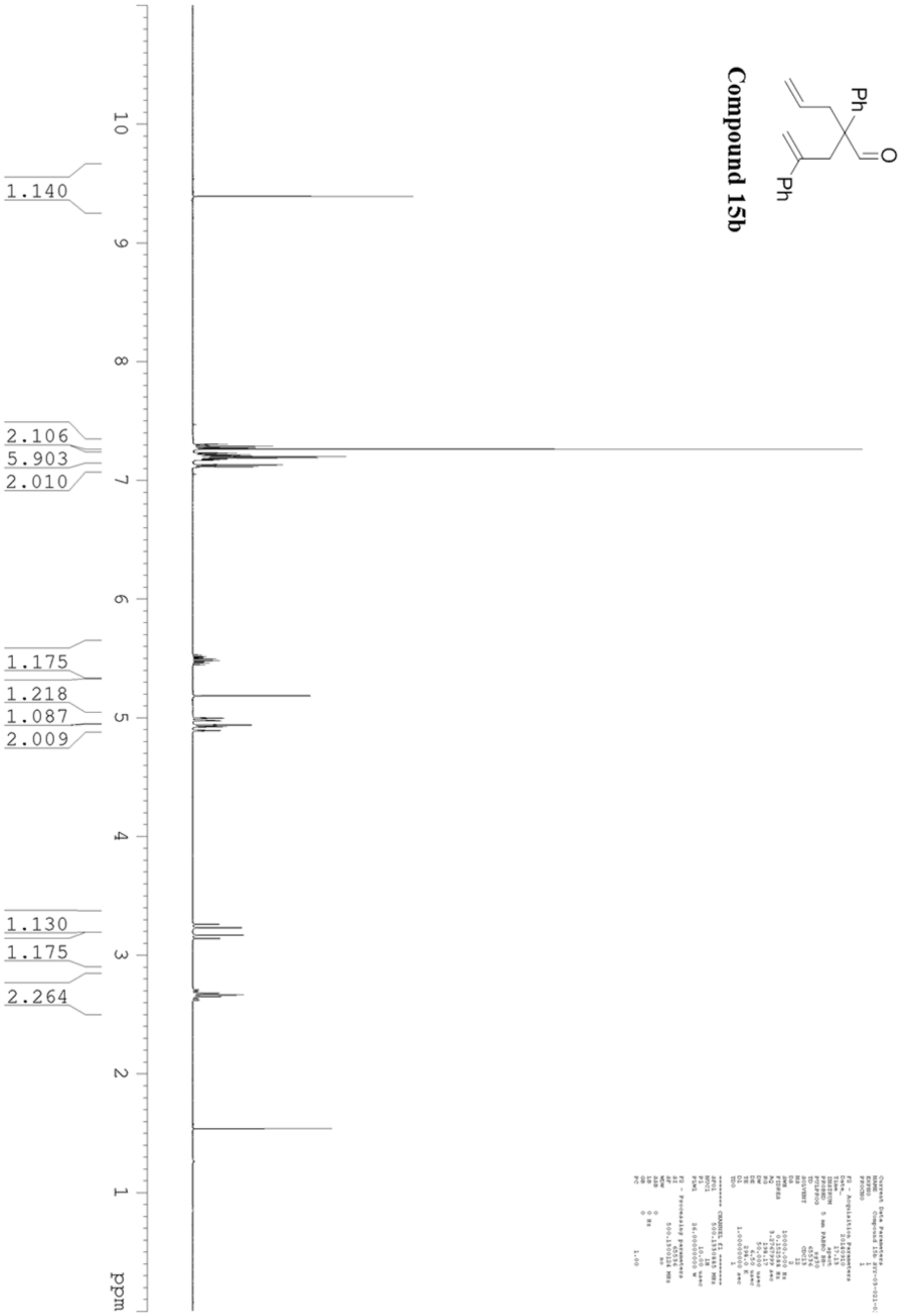
Ph
O
Ph
Compound 15b
10
9
8
7
6
5
4
3
2
1
ppm
1.140
2.106
5.903
2.010
1.175
1.218
1.087
2.009
1.130
1.175
2.264

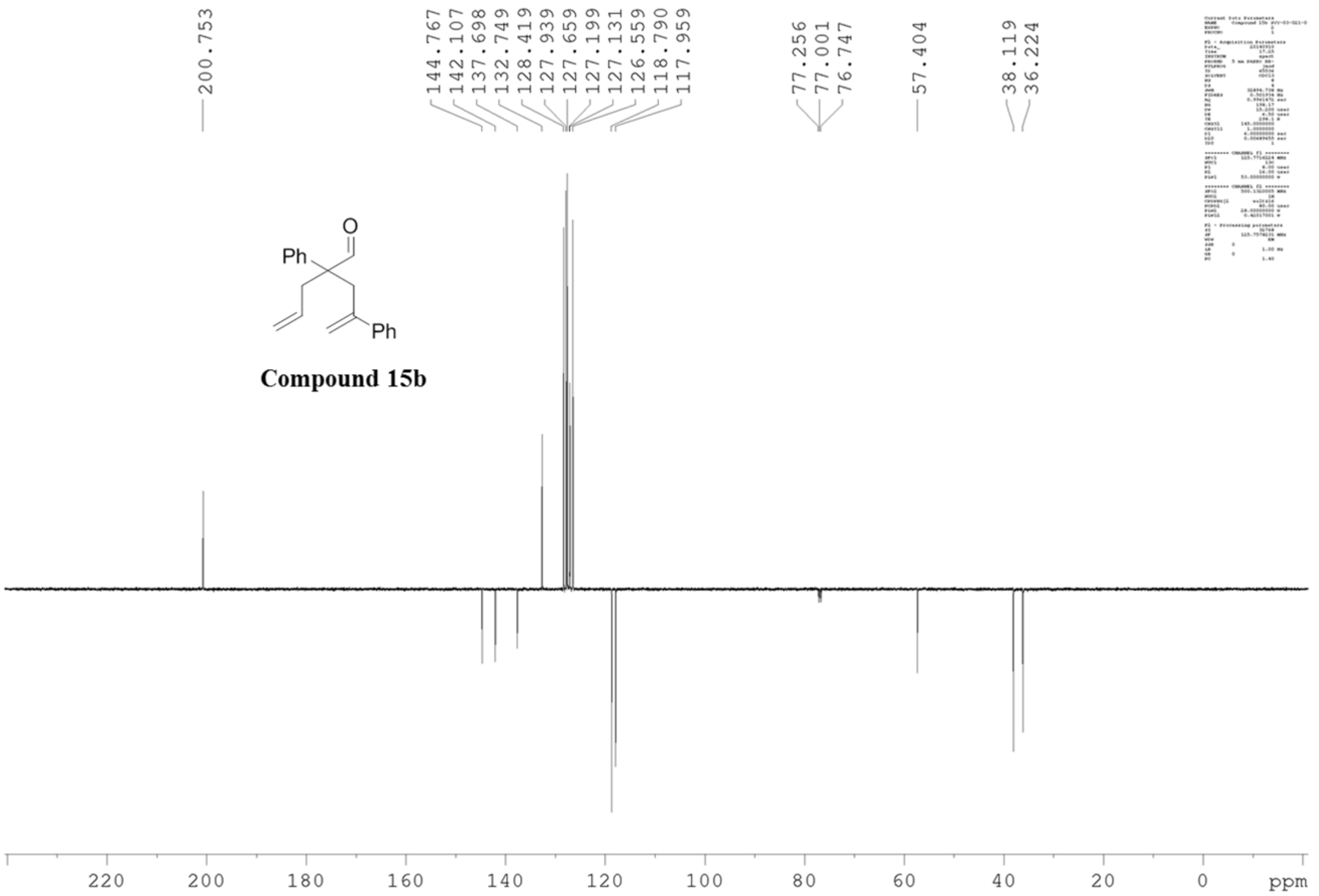
200.753
144.767
142.107
137.698
132.749
128.419
127.939
127.659
127.199
127.131
126.559
118.790
117.959
77.256
77.001
76.747
57.404
38.119
36.224
Ph
O
Ph
Compound 15b
220
200
180
160
140
120
100
80
60
40
20
0
ppm

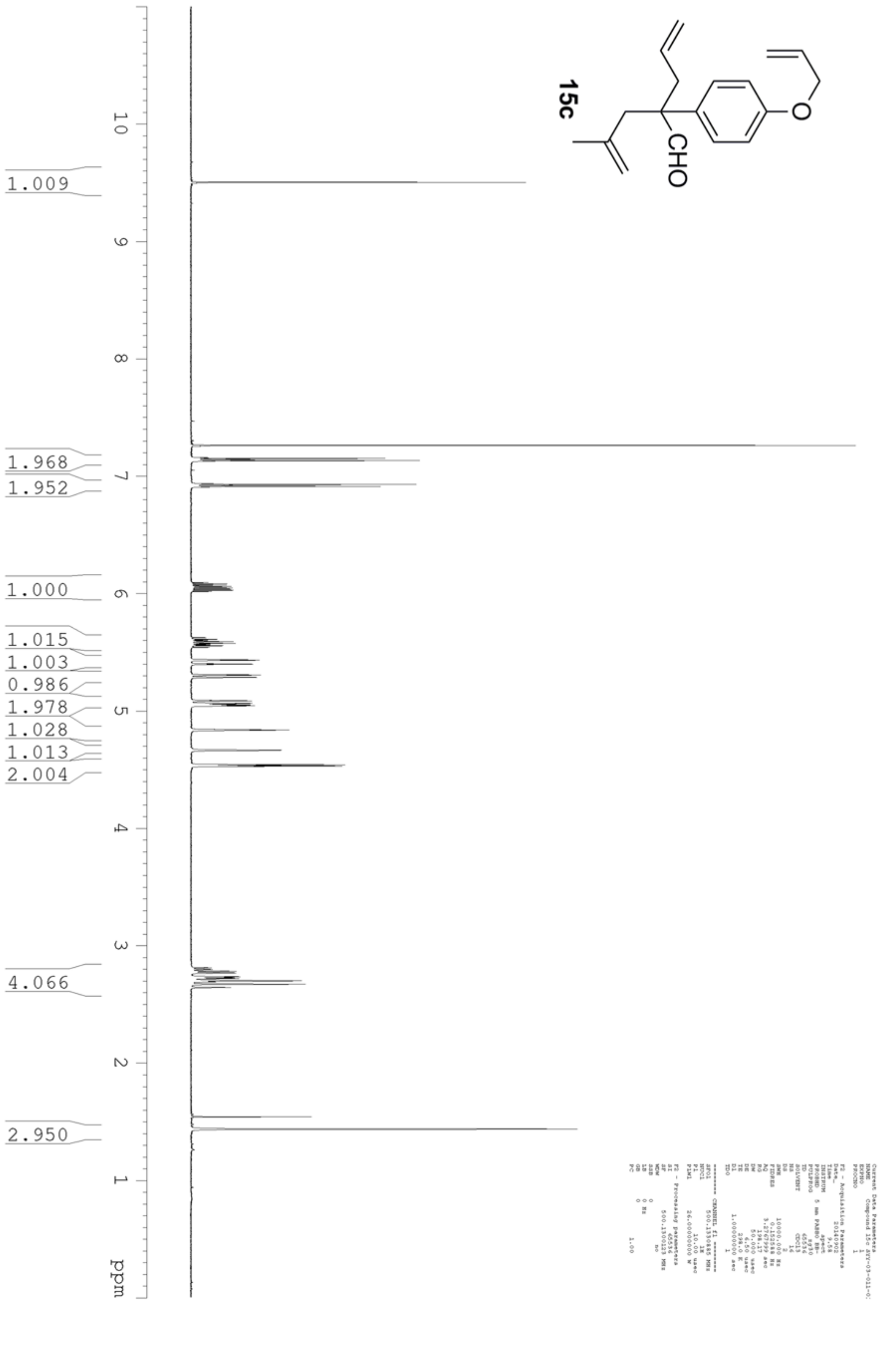
15c
CHO
O
1.009
1.968
1.952
1.000
1.015
1.003
0.986
1.978
1.028
1.013
2.004
4.066
2.950
10
9
8
7
6
5
4
3
2
1
ppm

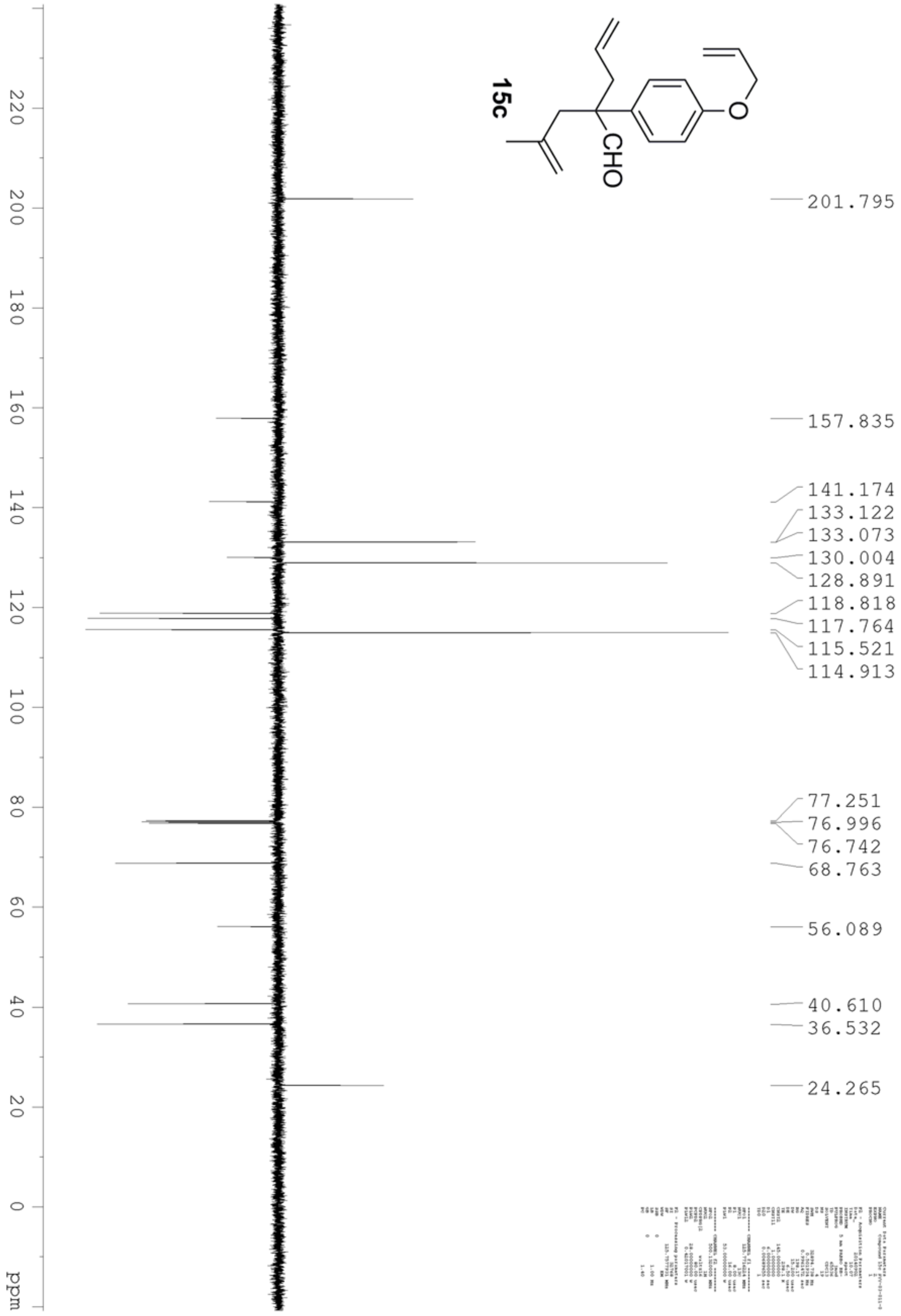
15c
CHO
O
201.795
157.835
141.174
133.122
133.073
130.004
128.891
118.818
117.764
115.521
114.913
77.251
76.996
76.742
68.763
56.089
40.610
36.532
24.265
ppm

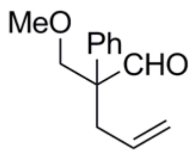

(±)-**18**

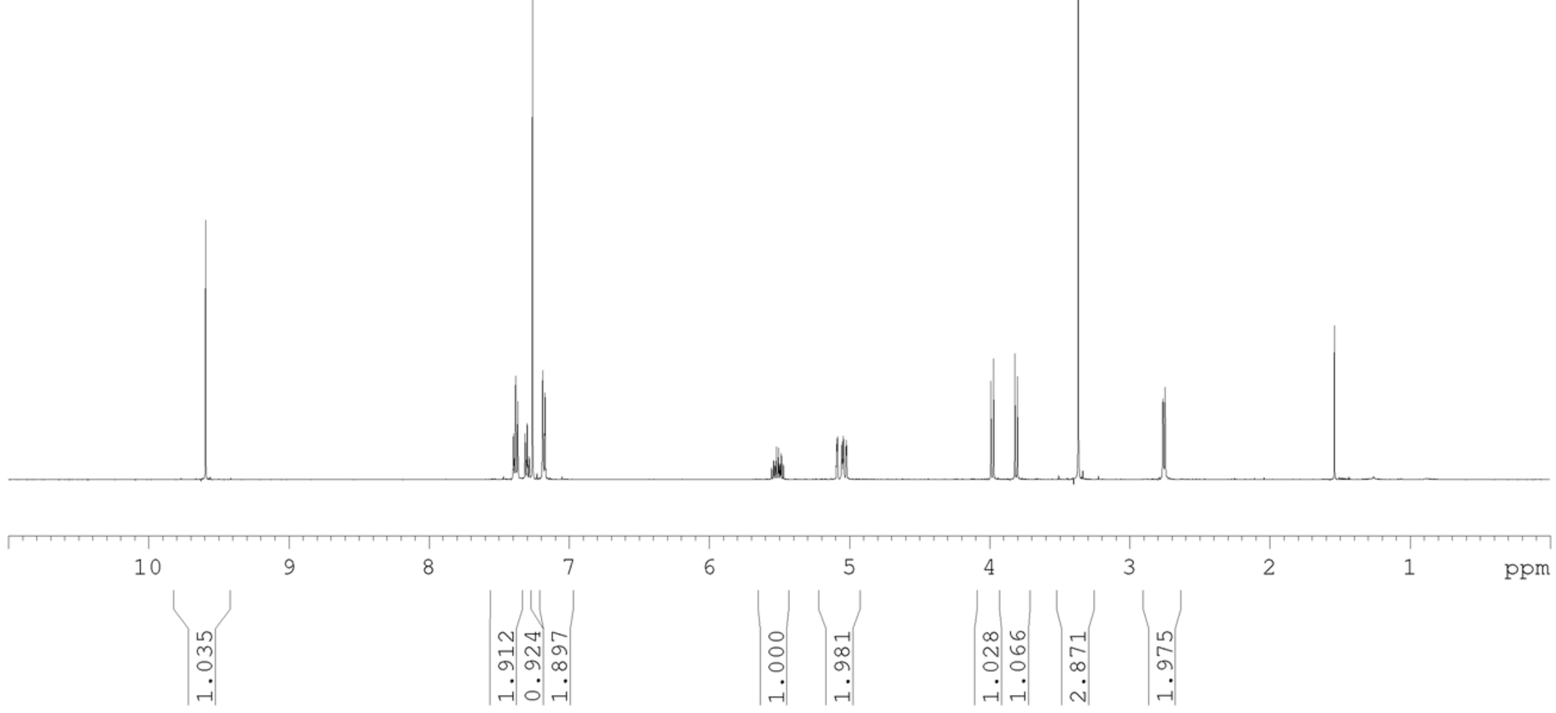

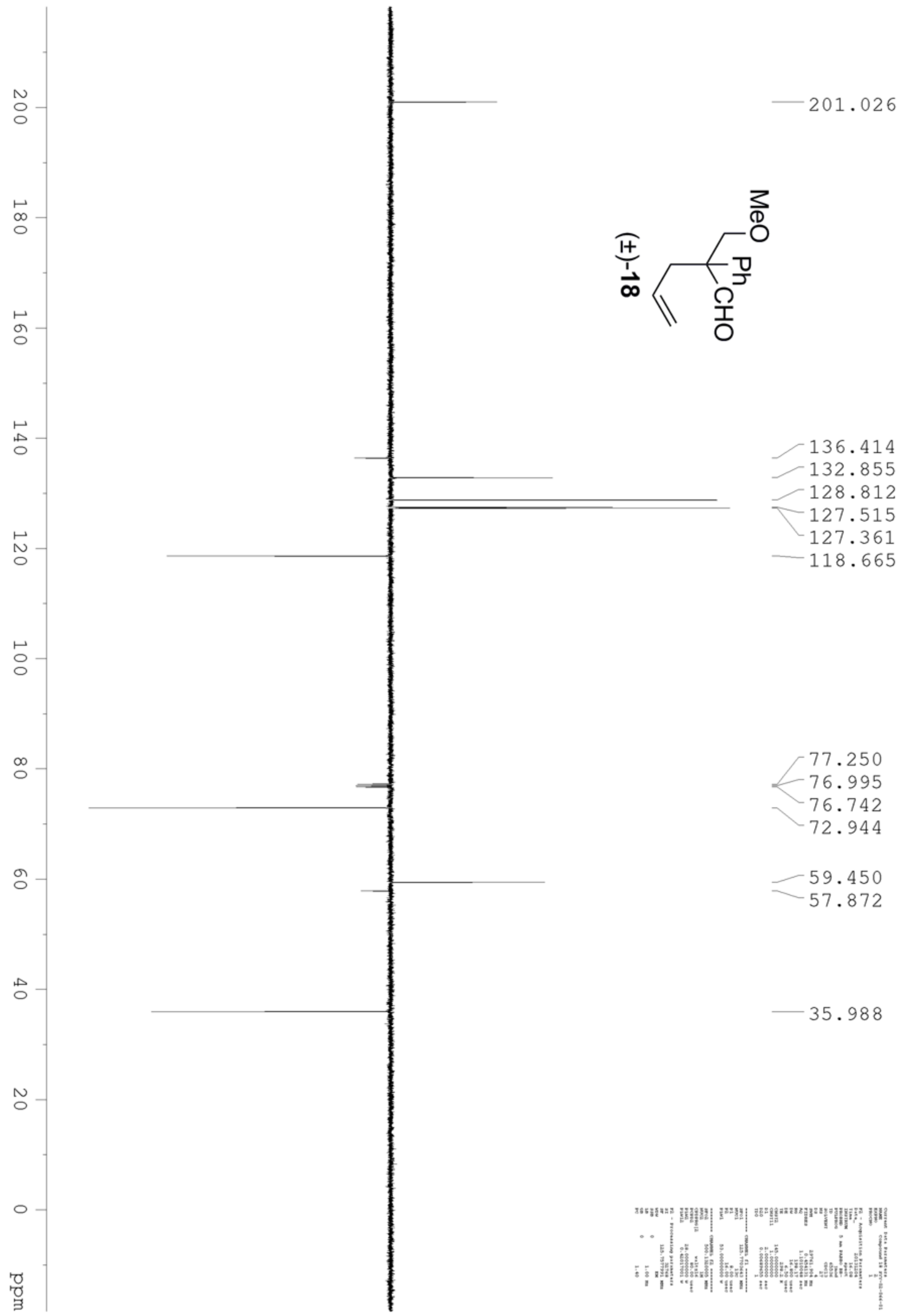
201.026
136.414
132.855
128.812
127.515
127.361
118.665
77.250
76.995
76.742
72.944
59.450
57.872
35.988
MeO
Ph
CHO
(±)-18
200
180
160
140
120
100
80
60
40
20
0
ppm

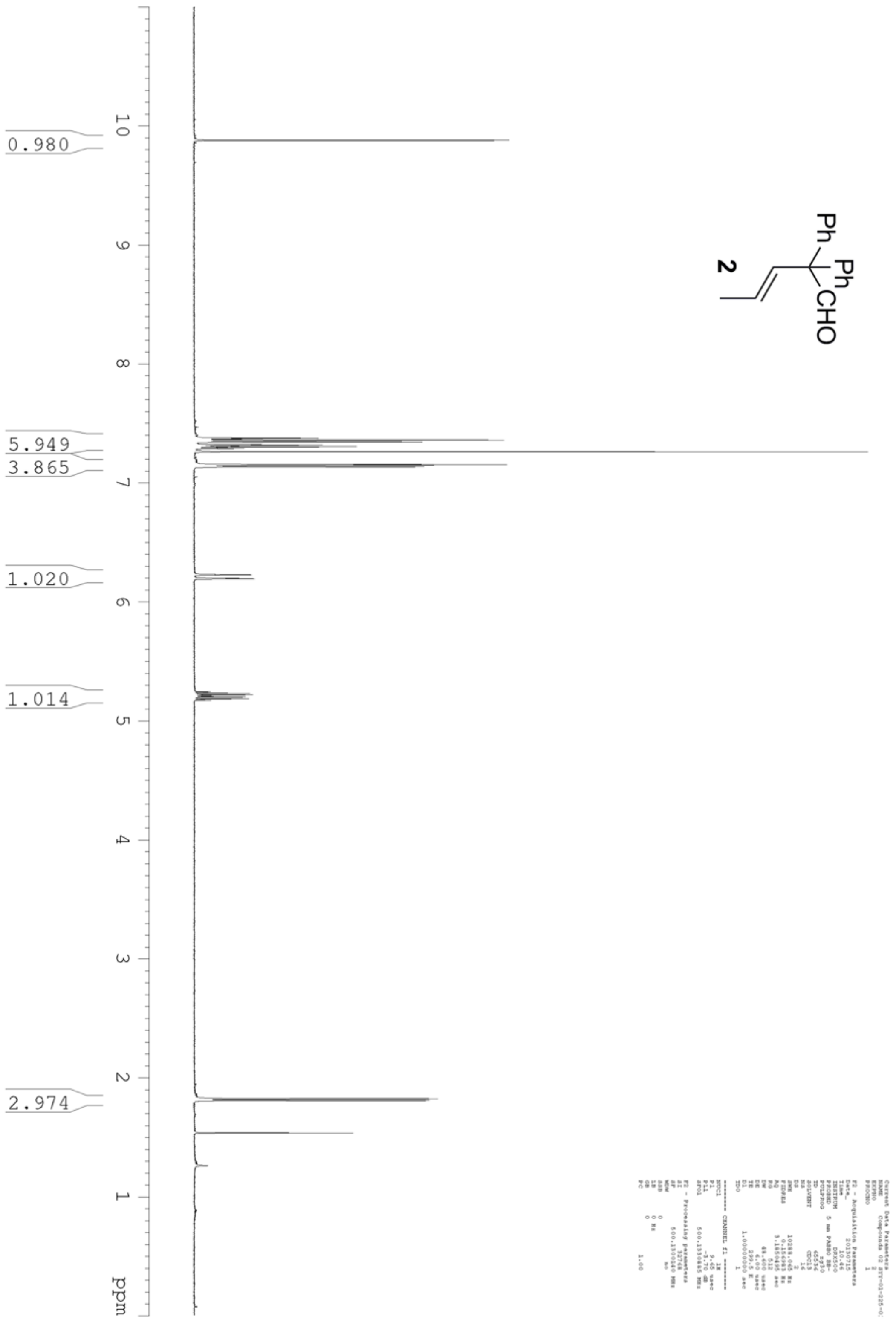
0.980
5.949
3.865
1.020
1.014
2.974
10
9
8
7
6
5
4
3
2
1
ppm
Ph
Ph
CHO
2

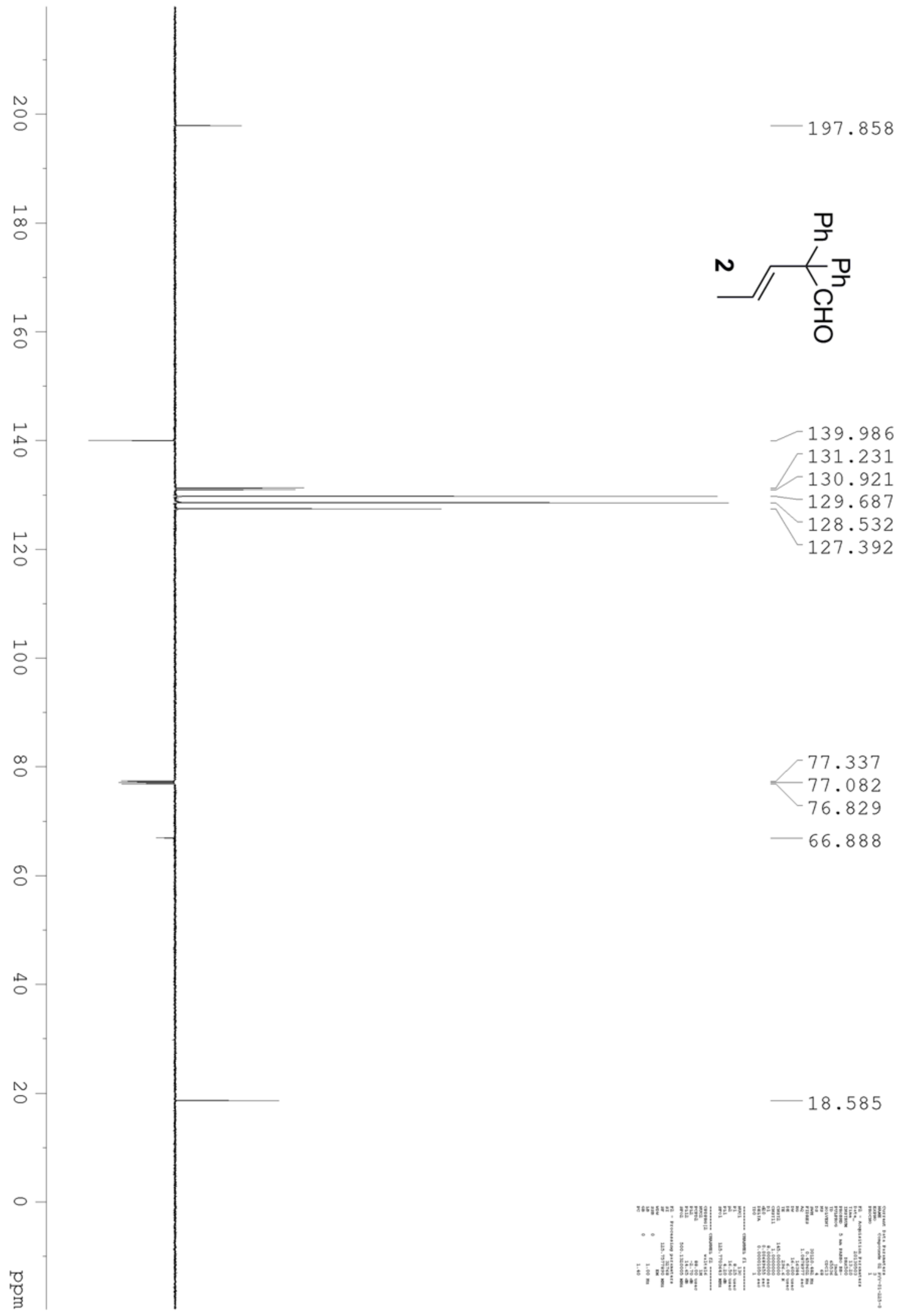
197.858
Ph
Ph
CHO
2
139.986
131.231
130.921
129.687
128.532
127.392
77.337
77.082
76.829
66.888
18.585
200
180
160
140
120
100
80
60
40
20
0
ppm

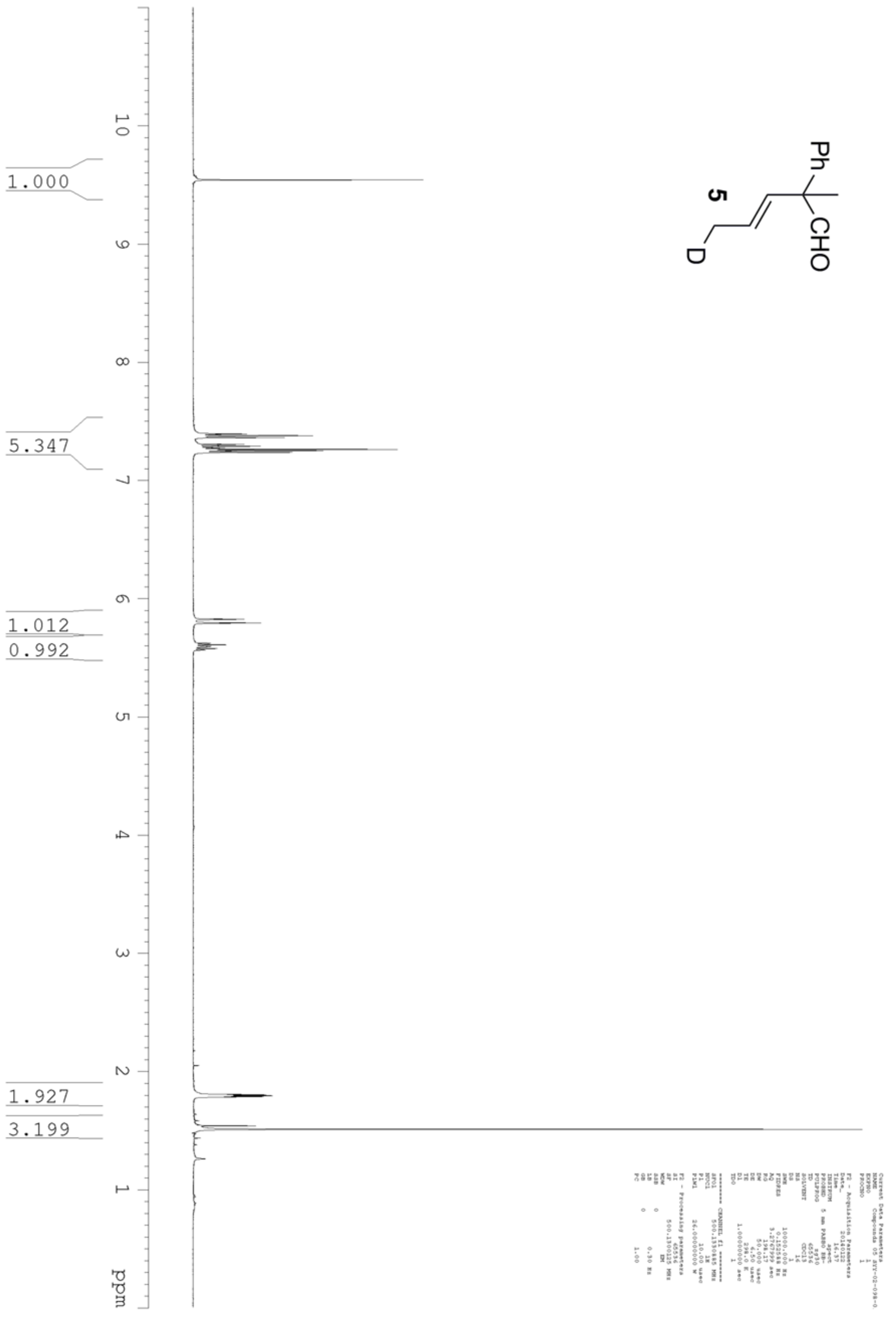

Ph
CHO
5
D
1.000
5.347
1.012
0.992
1.927
3.199
ppm

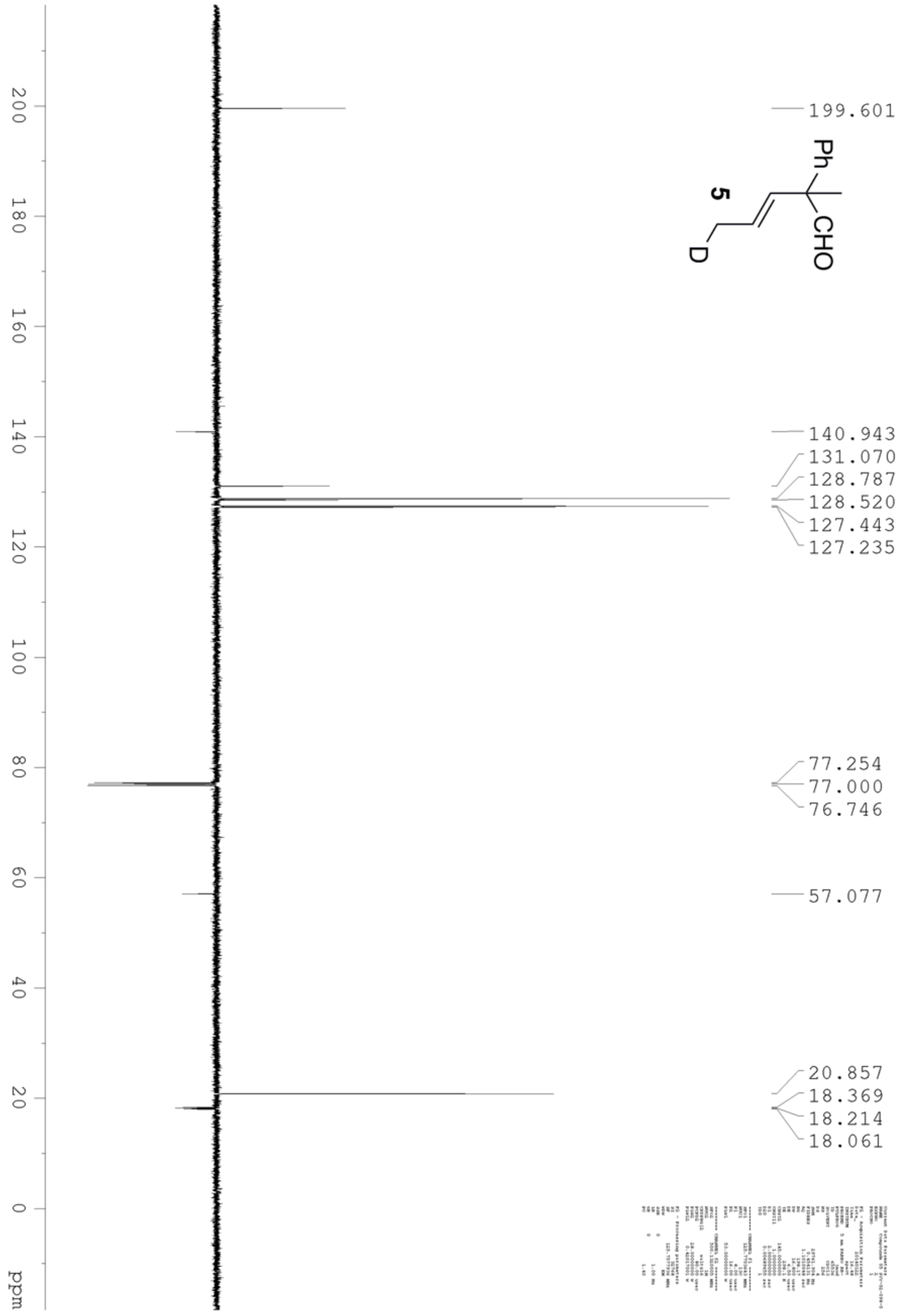
199.601
140.943
131.070
128.787
128.520
127.443
127.235
77.254
77.000
76.746
57.077
20.857
18.369
18.214
18.061
Ph
CHO
D
5
ppm

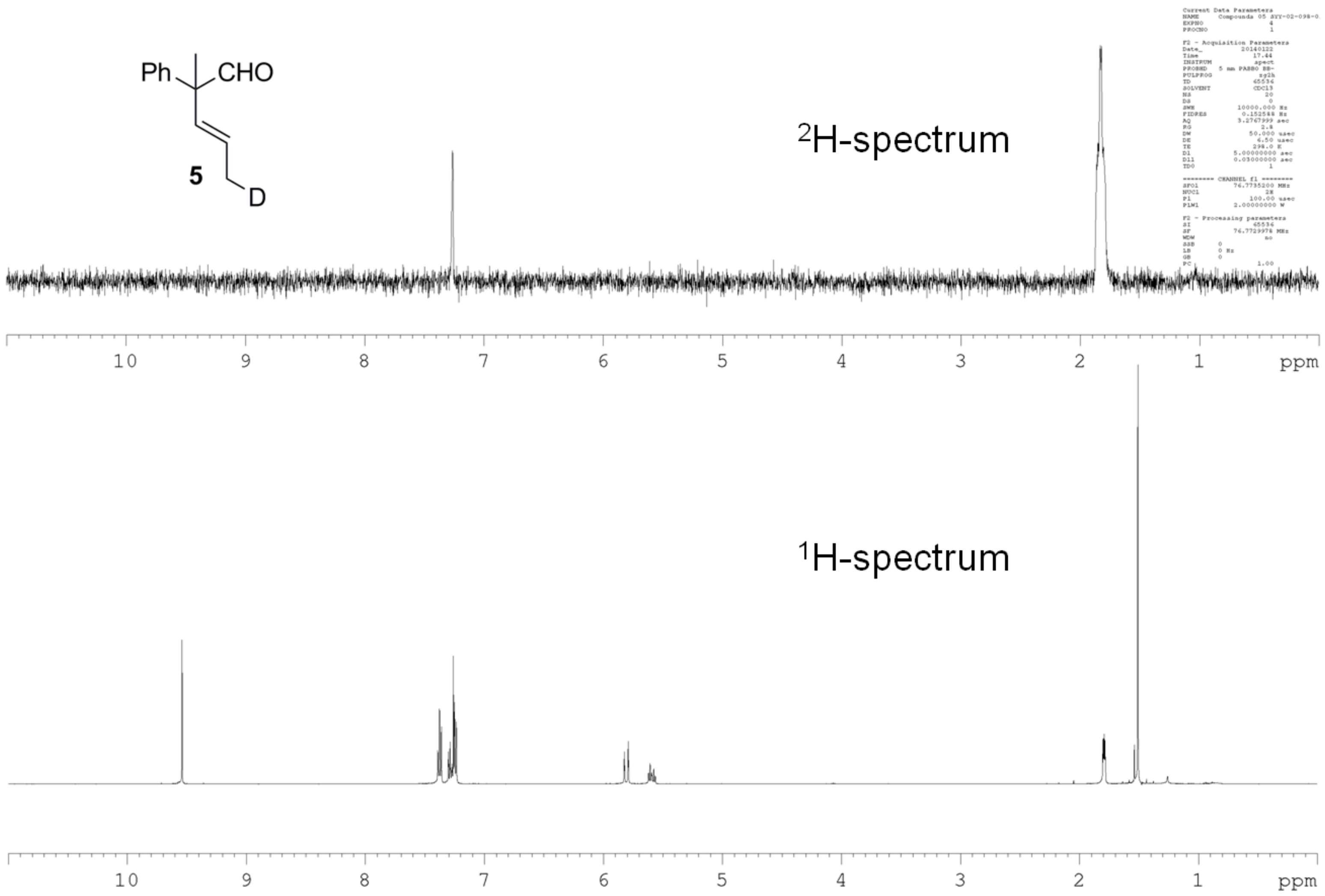
Ph
CHO
5
D
2H-spectrum
1H-spectrum
10 9 8 7 6 5 4 3 2 1 ppm
10 9 8 7 6 5 4 3 2 1 ppm

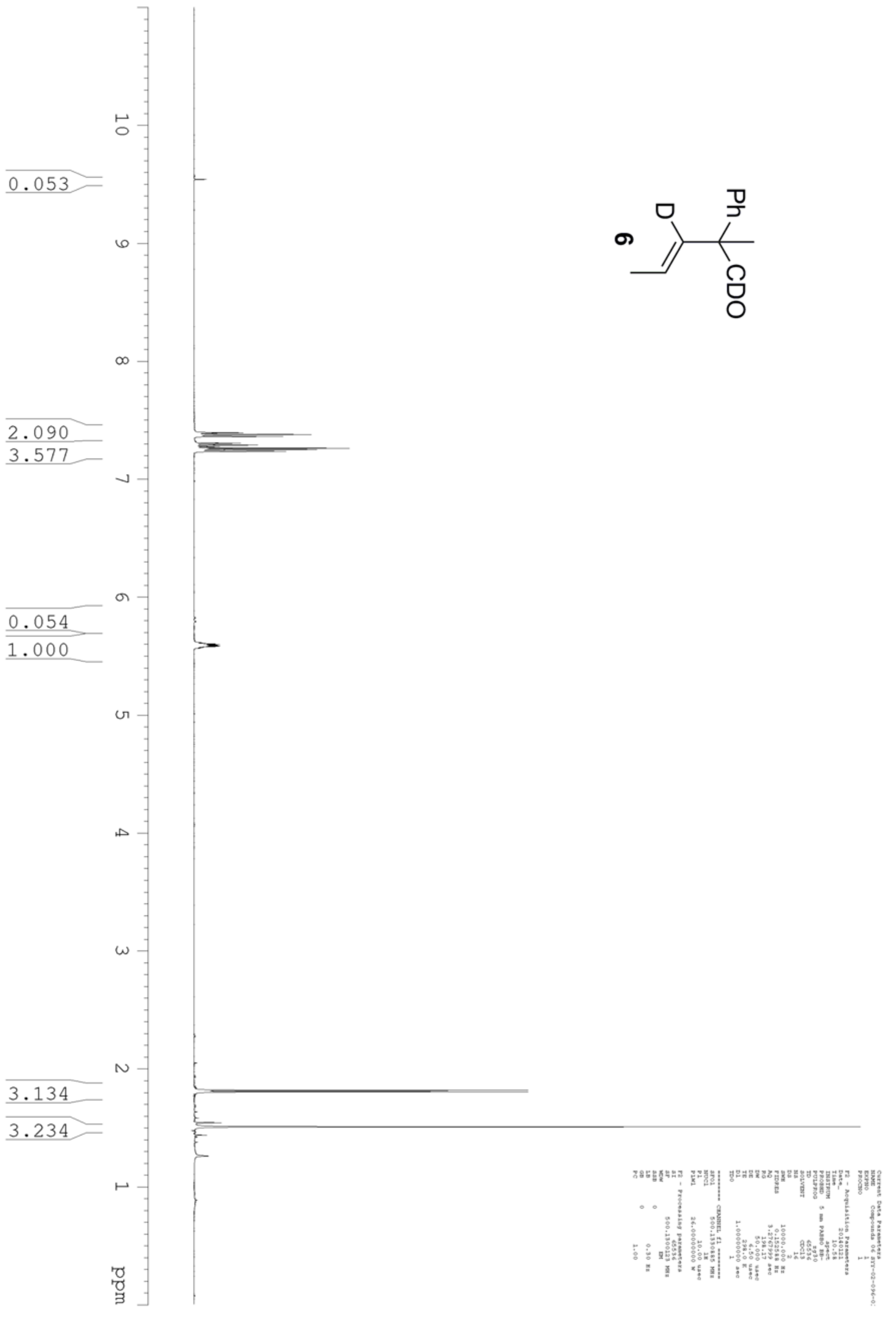
Ph
CDO
D
6
0.053
2.090
3.577
0.054
1.000
3.134
3.234
10
9
8
7
6
5
4
3
2
1
ppm

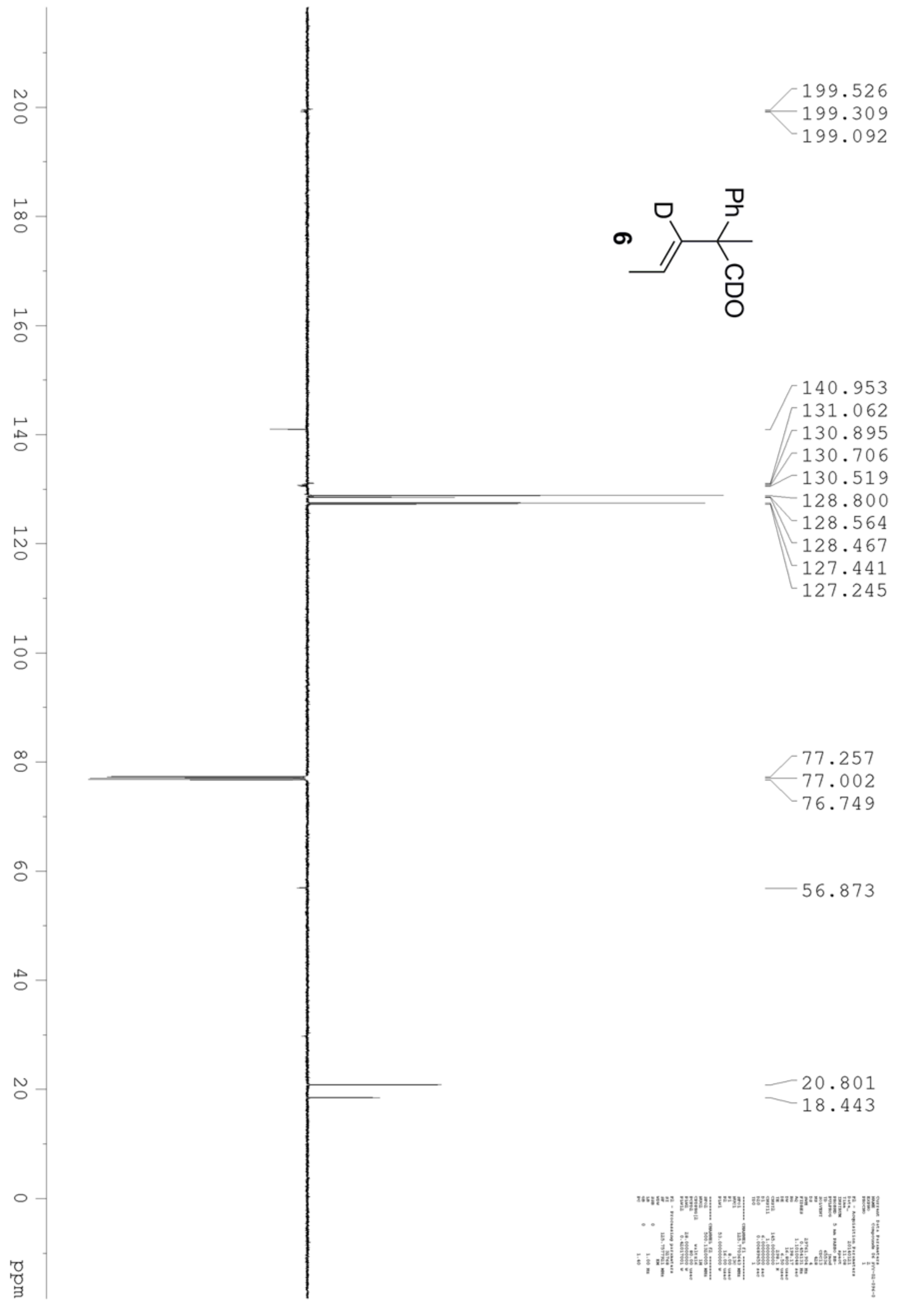

199.526
199.309
199.092
Ph
D
CDO
6
140.953
131.062
130.895
130.706
130.519
128.800
128.564
128.467
127.441
127.245
77.257
77.002
76.749
56.873
20.801
18.443
200
180
160
140
120
100
80
60
40
20
0
ppm

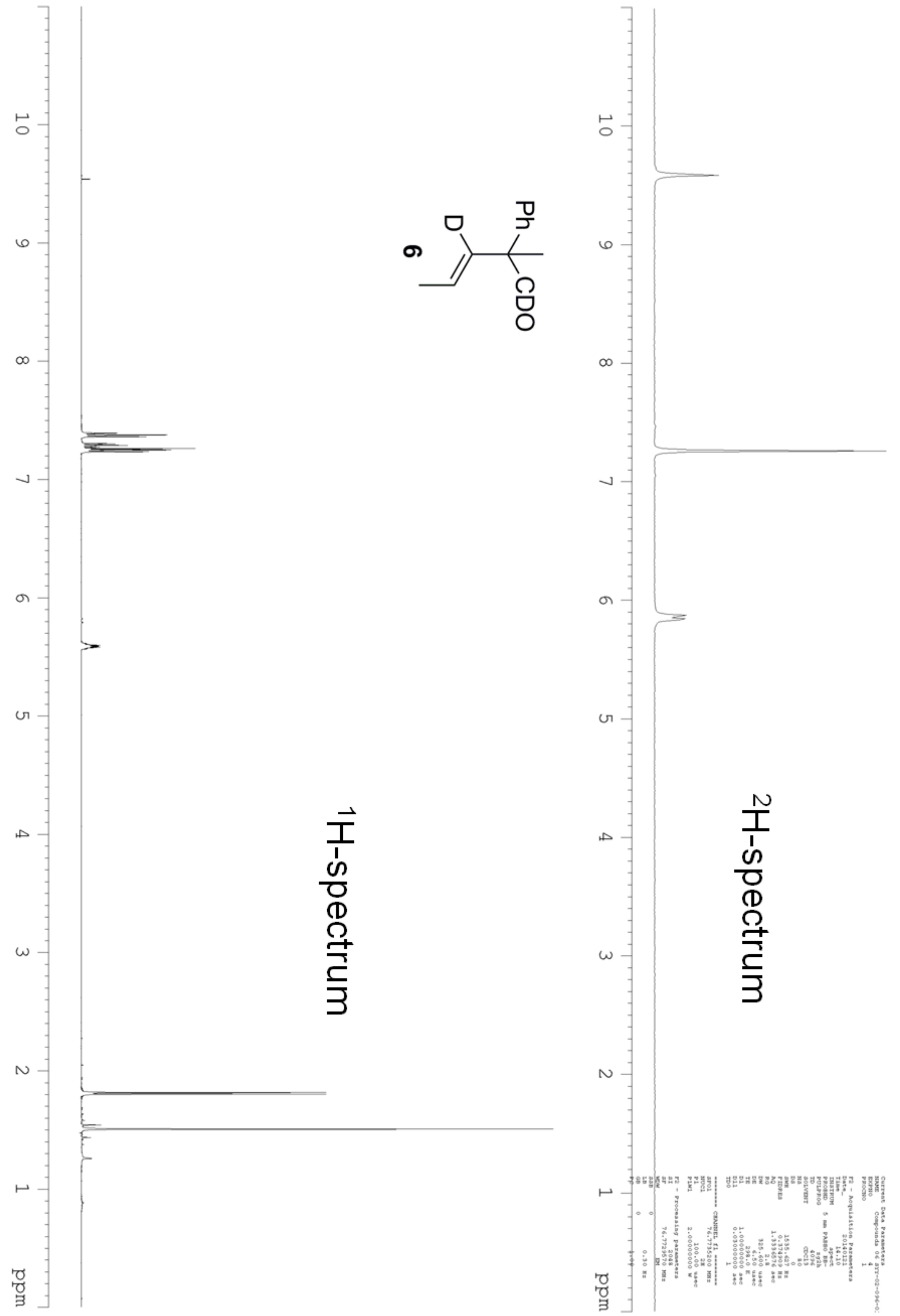

Ph
CDO
D
6
²H-spectrum
¹H-spectrum
10
9
8
7
6
5
4
3
2
1
ppm

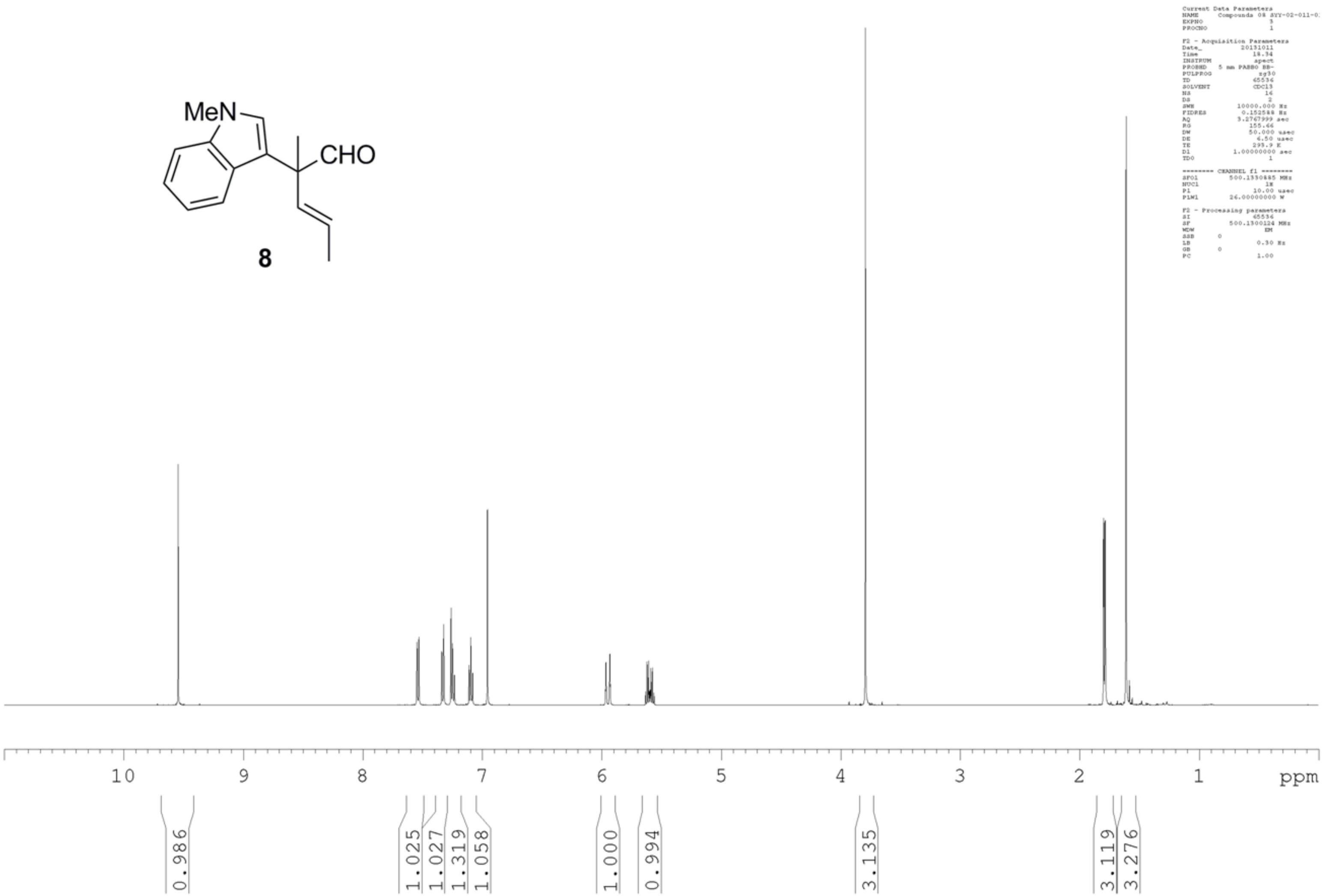
MeN
CHO
8
Current Data Parameters
NAME Compounds 08 SYY-02-011-0
EXPNO 3
PROCNO 1
F2 - Acquisition Parameters
Date_ 20131011
Time 18.34
INSTRUM spect
PROBHD 5 mm PABBO BB-
PULPROG zg30
TD 65536
SOLVENT CDCl3
NS 16
DS 2
SWH 10000.000 Hz
FIDRES 0.152588 Hz
AQ 3.2767999 sec
RG 155.46
DW 50.000 usec
DE 6.50 usec
TE 293.9 K
D1 1.00000000 sec
TD0 1
======== CHANNEL f1 ========
SFO1 500.1330885 MHz
NUC1 1H
P1 10.00 usec
PLW1 26.00000000 W
F2 - Processing parameters
SI 65536
SF 500.1300124 MHz
WDW EM
SSB 0
LB 0.30 Hz
GB 0
PC 1.00
10
9
8
7
6
5
4
3
2
1
ppm
0.986
1.025
1.027
1.319
1.058
1.000
0.994
3.135
3.119
3.276

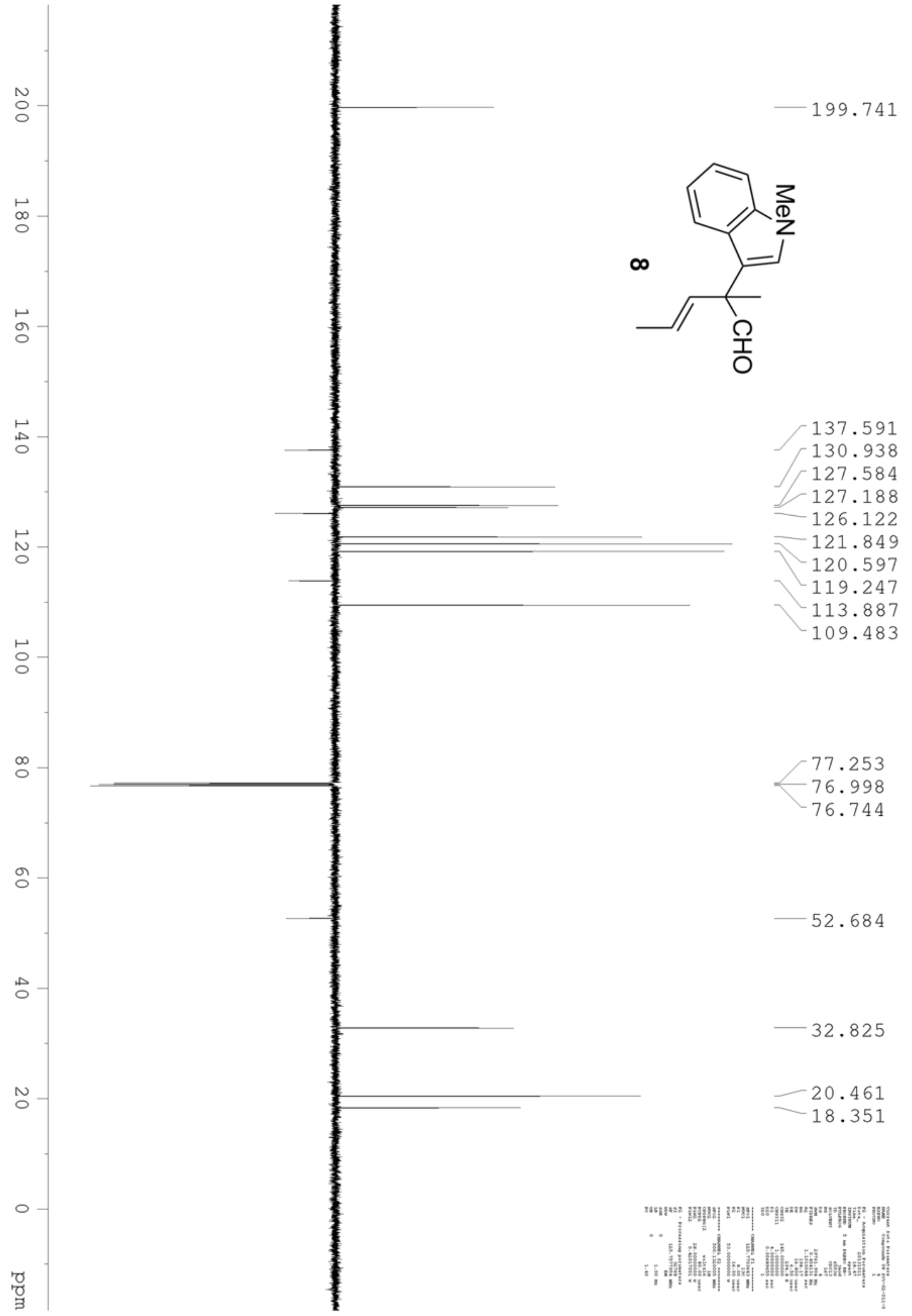
199.741
137.591
130.938
127.584
127.188
126.122
121.849
120.597
119.247
113.887
109.483
77.253
76.998
76.744
52.684
32.825
20.461
18.351
MeN
CHO
8
ppm
200
180
160
140
120
100
80
60
40
20
0

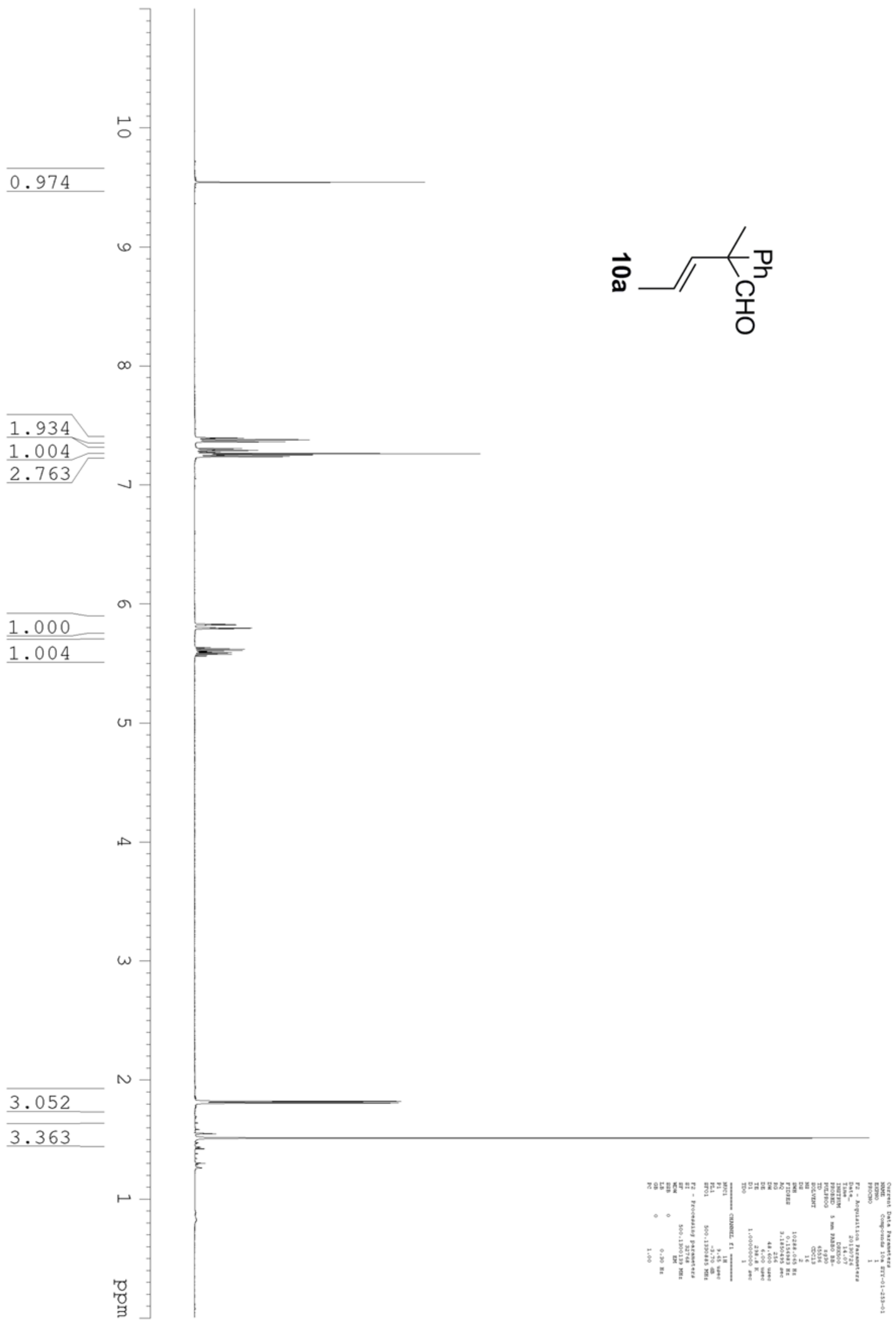
Ph
CHO
10a
0.974
1.934
1.004
2.763
1.000
1.004
3.052
3.363
ppm

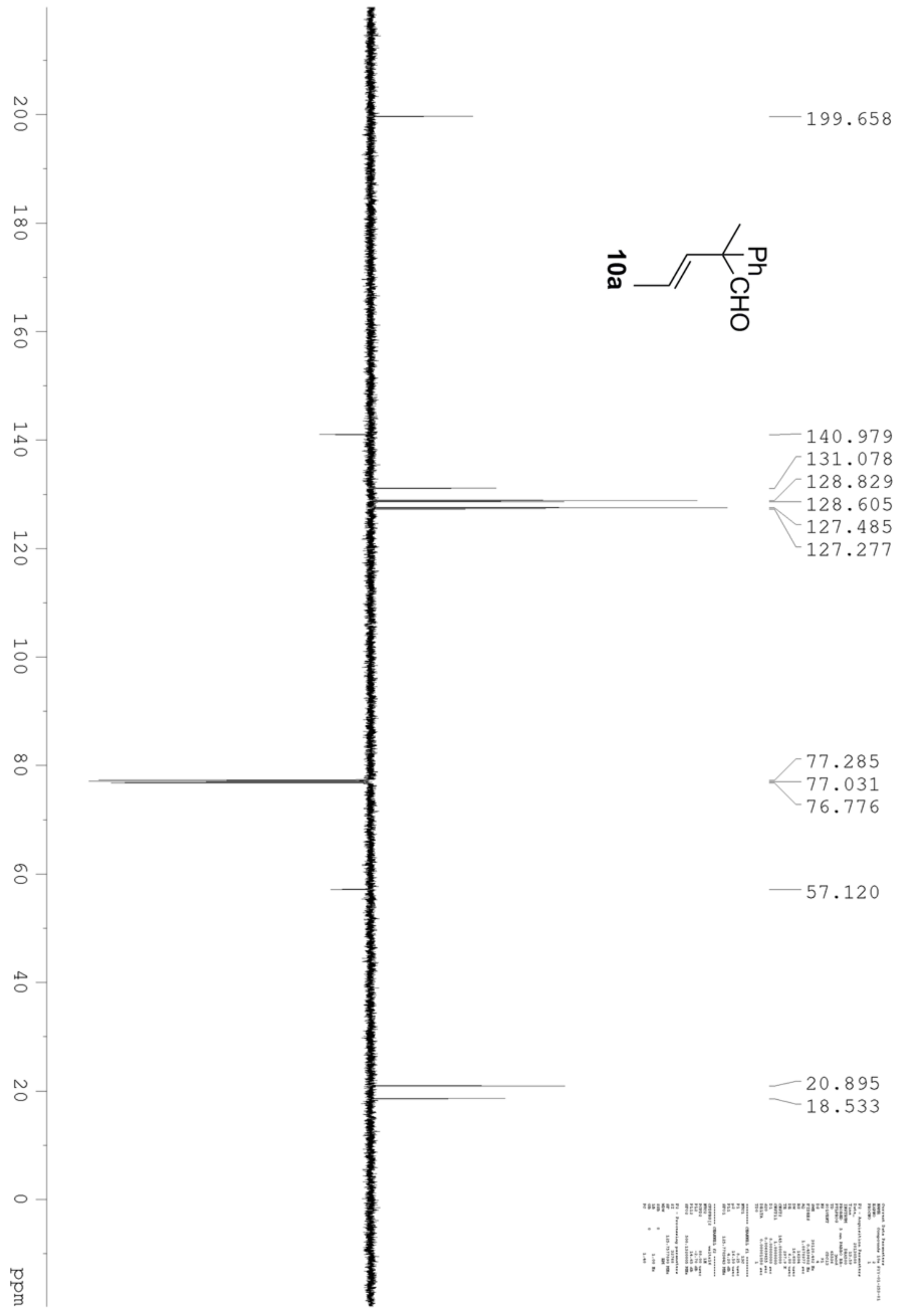
199.658
140.979
131.078
128.829
128.605
127.485
127.277
77.285
77.031
76.776
57.120
20.895
18.533
Ph
CHO
10a
200
180
160
140
120
100
80
60
40
20
0
ppm

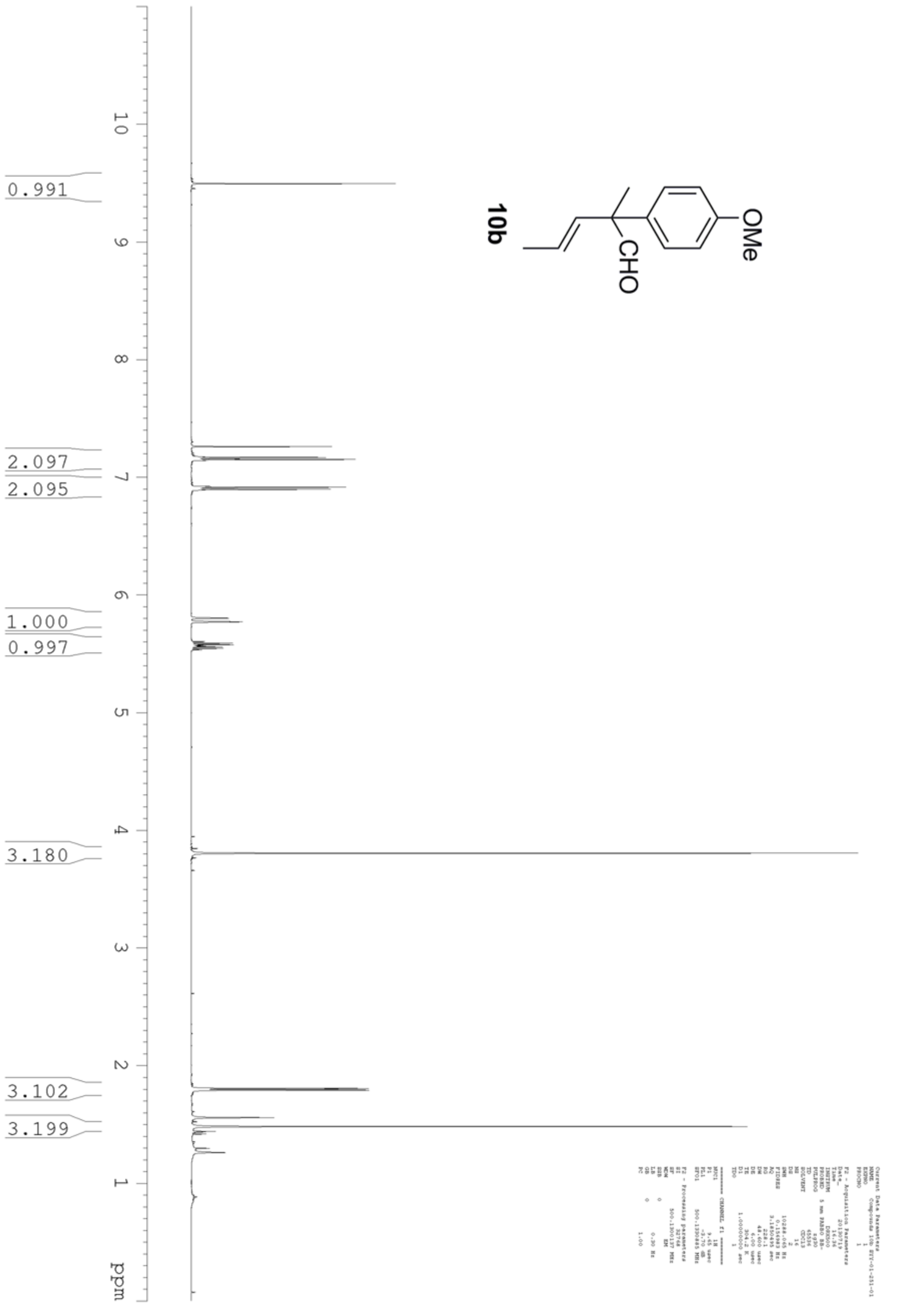
10b
OMe
CHO
0.991
2.097
2.095
1.000
0.997
3.180
3.102
3.199
10
9
8
7
6
5
4
3
2
1
ppm

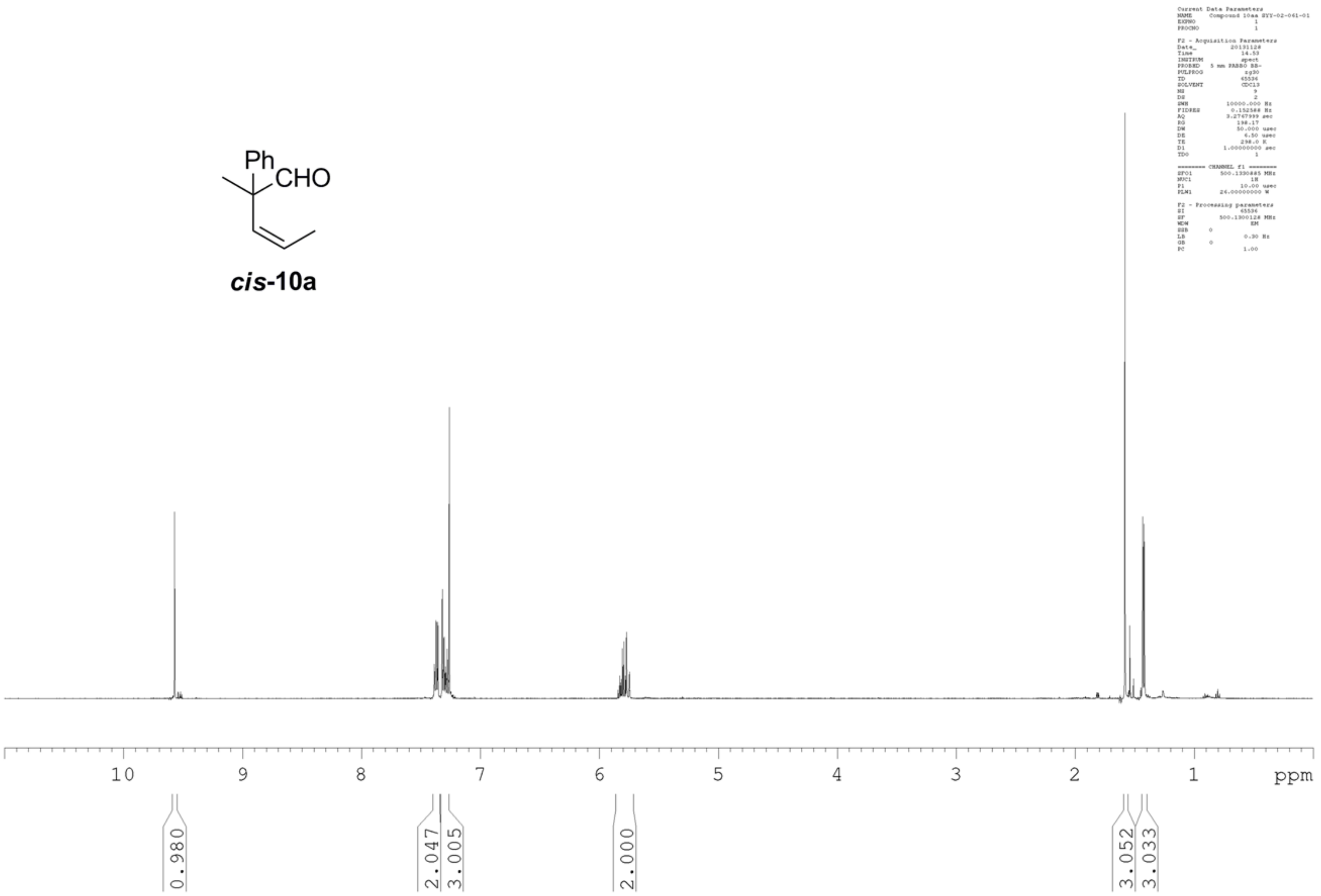

cis-**10a**

Current Data Parameters
NAME Compound 10aa SYY-02-061-01
EXPNO 1
PROCNO 1
F2 - Acquisition Parameters
Date_ 20131128
Time 14.53
INSTRUM spect
PROBHD 5 mm PABBO BB-
PULPROG zg30
TD 65536
SOLVENT CDCl3
NS 9
DS 2
SWH 10000.000 Hz
FIDRES 0.152588 Hz
AQ 3.2767999 sec
RG 198.17
DW 50.000 usec
DE 6.50 usec
TE 298.0 K
D1 1.00000000 sec
TD0 1
======== CHANNEL f1 ========
SFO1 500.1330885 MHz
NUC1 1H
P1 10.00 usec
PLW1 26.00000000 W
F2 - Processing parameters
SI 65536
SF 500.1300128 MHz
WDW EM
SSB 0
LB 0.30 Hz
GB 0
PC 1.00
10
9
8
7
6
5
4
3
2
1
ppm
0.980
2.047
3.005
2.000
3.052
3.033

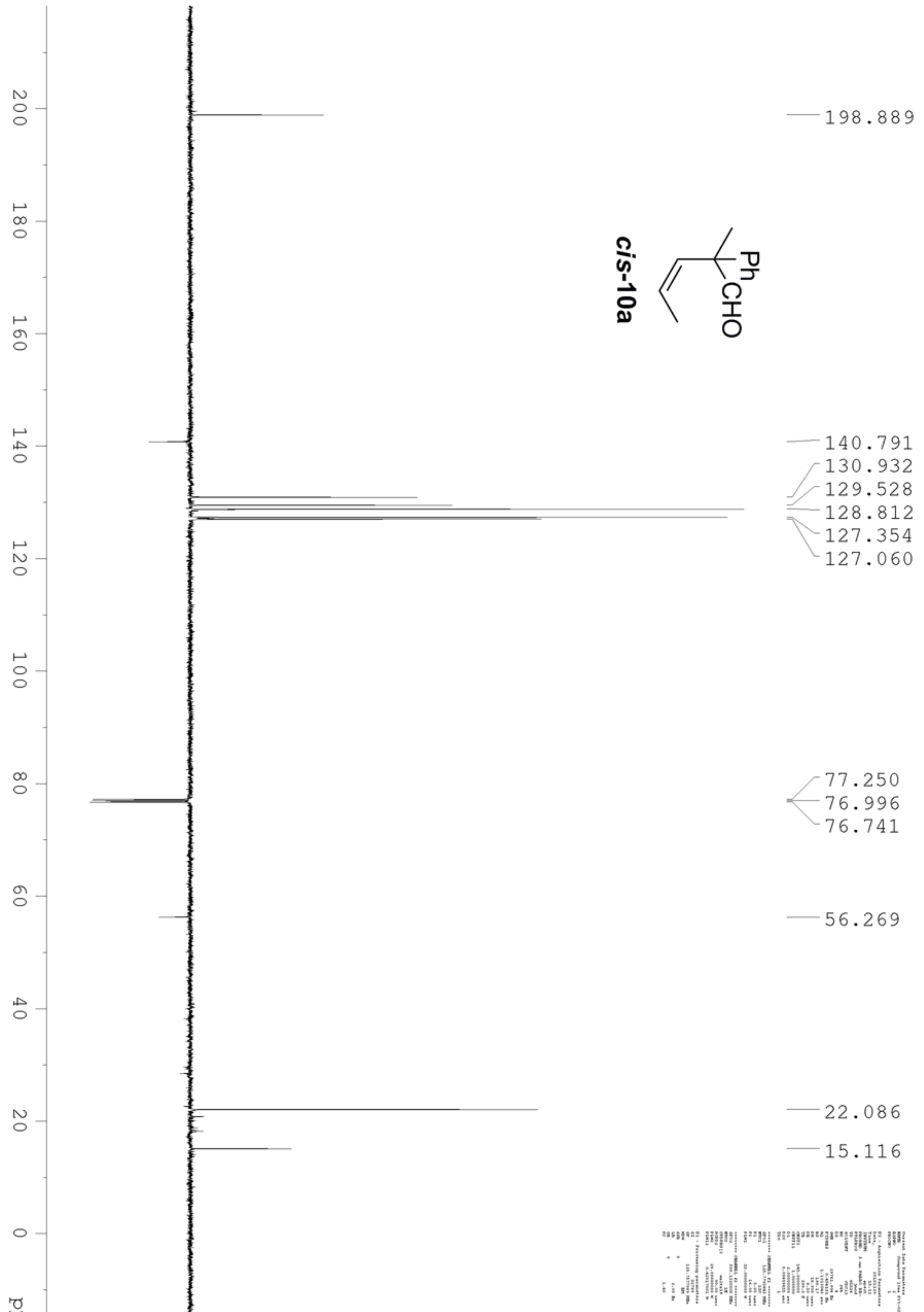
cis-10a
198.889
140.791
130.932
129.528
128.812
127.354
127.060
77.250
76.996
76.741
56.269
22.086
15.116
ppm

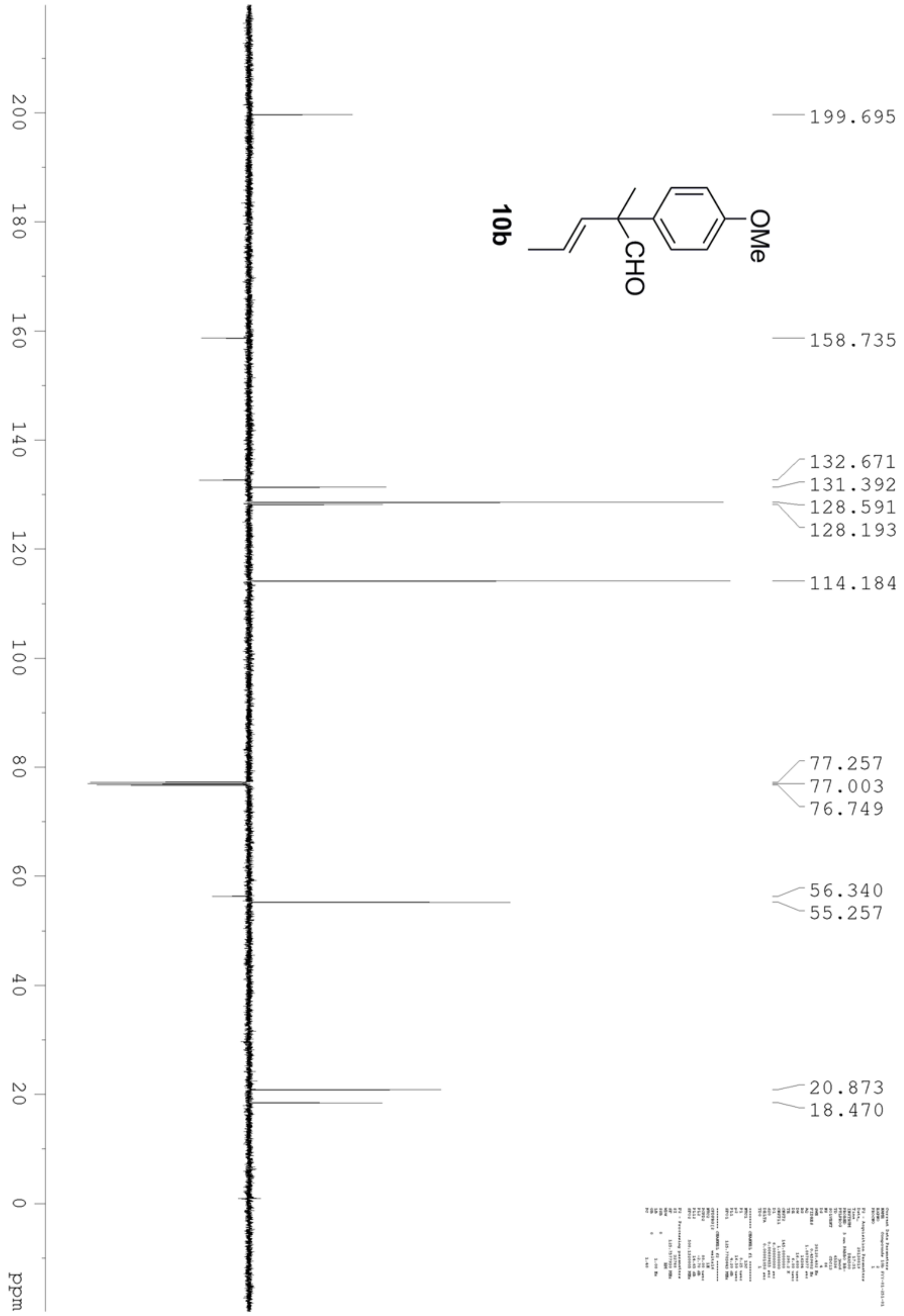

10b
CHO
OMe
199.695
158.735
132.671
131.392
128.591
128.193
114.184
77.257
77.003
76.749
56.340
55.257
20.873
18.470
200
180
160
140
120
100
80
60
40
20
0
ppm

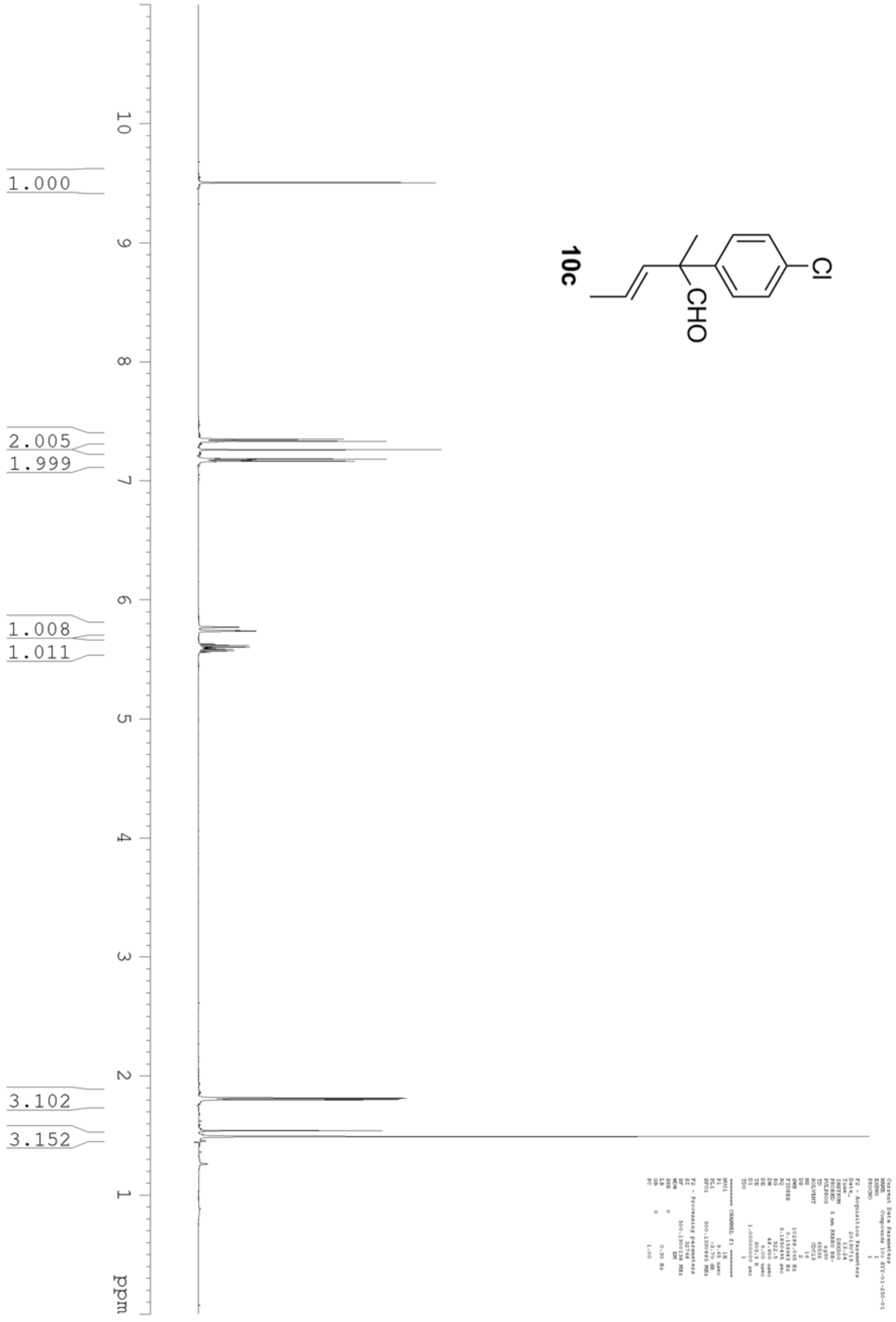
10c
CHO
Cl
1.000
2.005
1.999
1.008
1.011
3.102
3.152
10
9
8
7
6
5
4
3
2
1
ppm

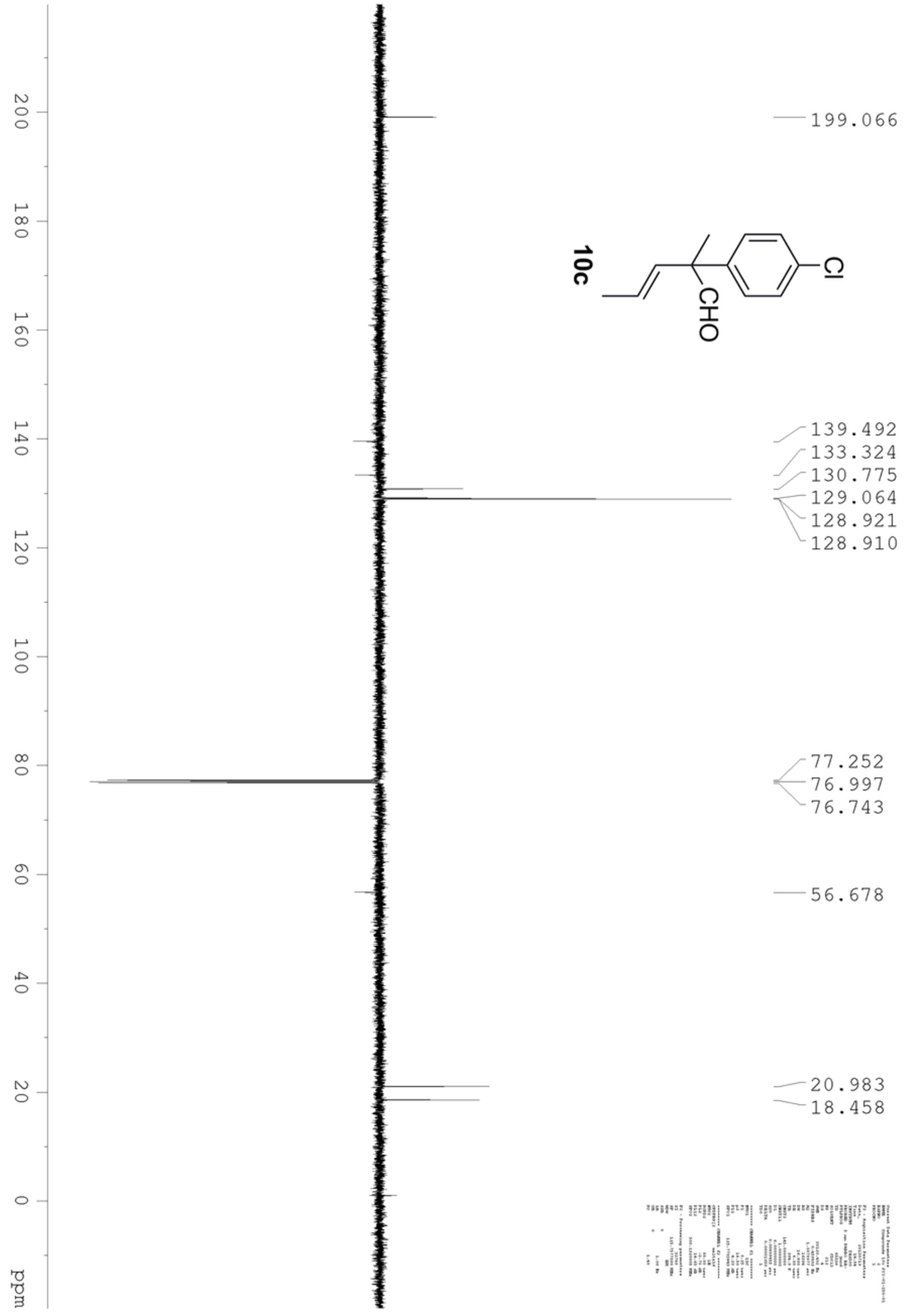

199.066
10c
CHO
Cl
139.492
133.324
130.775
129.064
128.921
128.910
77.252
76.997
76.743
56.678
20.983
18.458
ppm

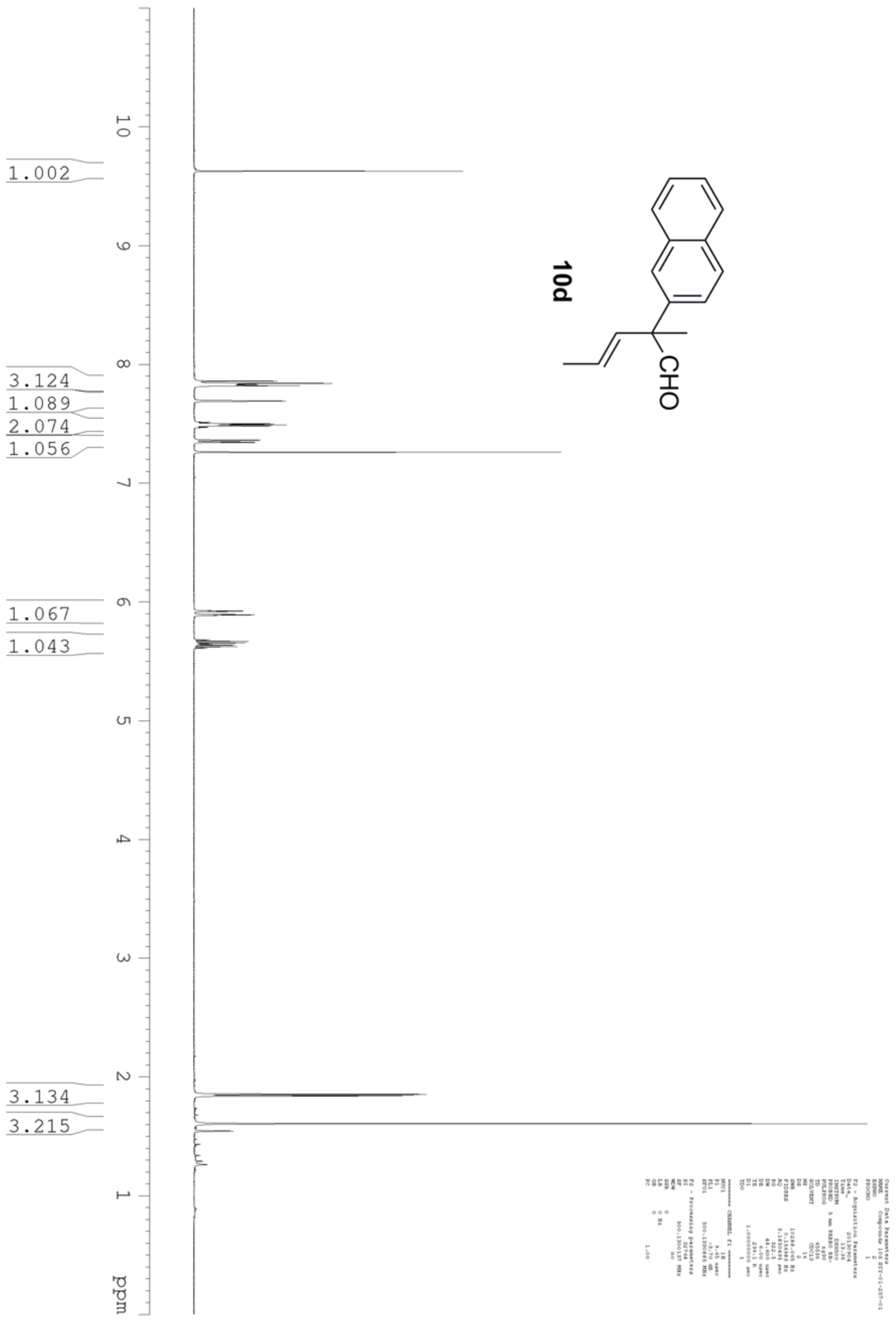
10d
CHO
1.002
3.124
1.089
2.074
1.056
1.067
1.043
3.134
3.215
10
9
8
7
6
5
4
3
2
1
ppm

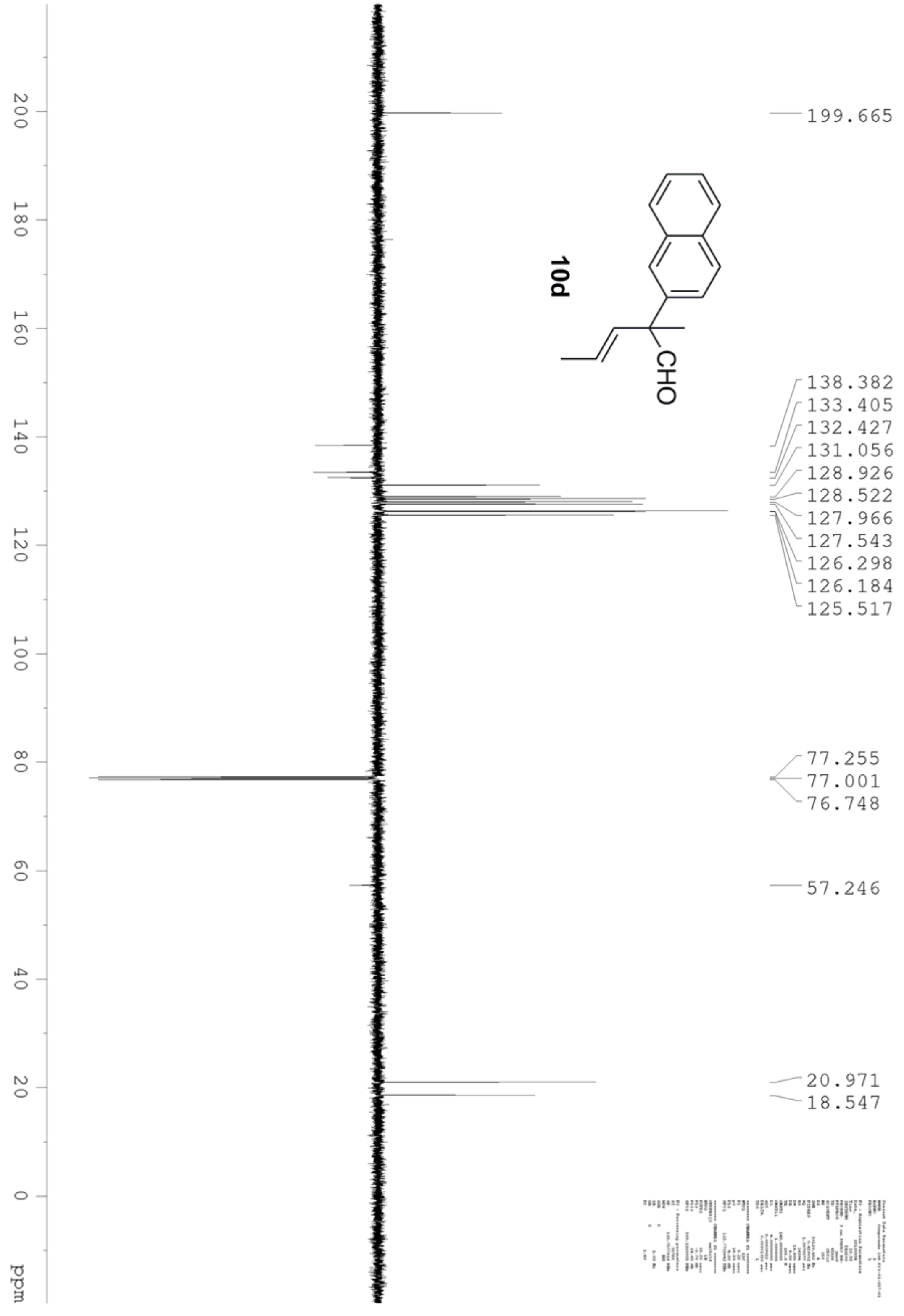
199.665
10d
CHO
138.382
133.405
132.427
131.056
128.926
128.522
127.966
127.543
126.298
126.184
125.517
77.255
77.001
76.748
57.246
20.971
18.547
200
180
160
140
120
100
80
60
40
20
0
ppm

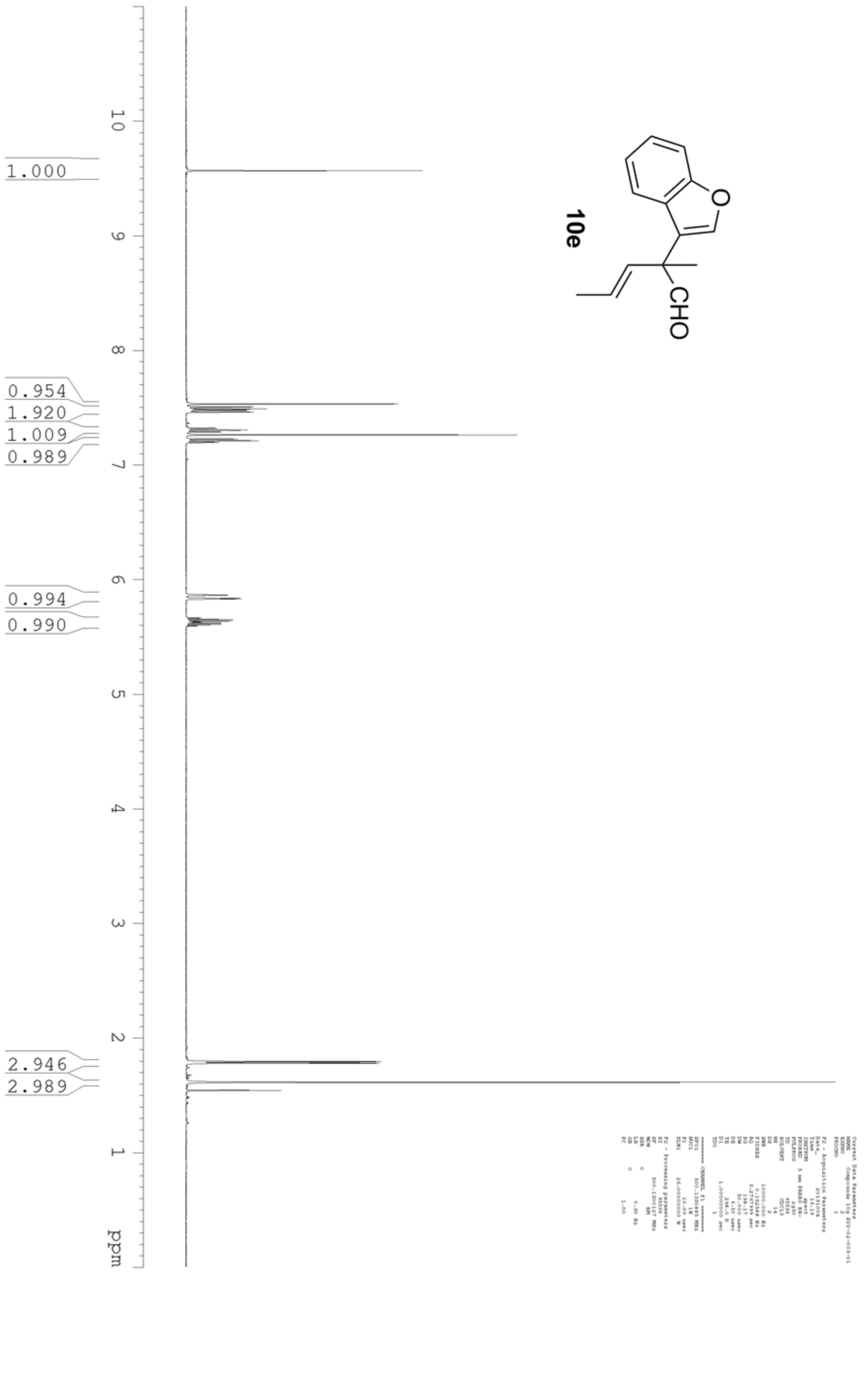

10e
ppm
1.000
0.954
1.920
1.009
0.989
0.994
0.990
2.946
2.989

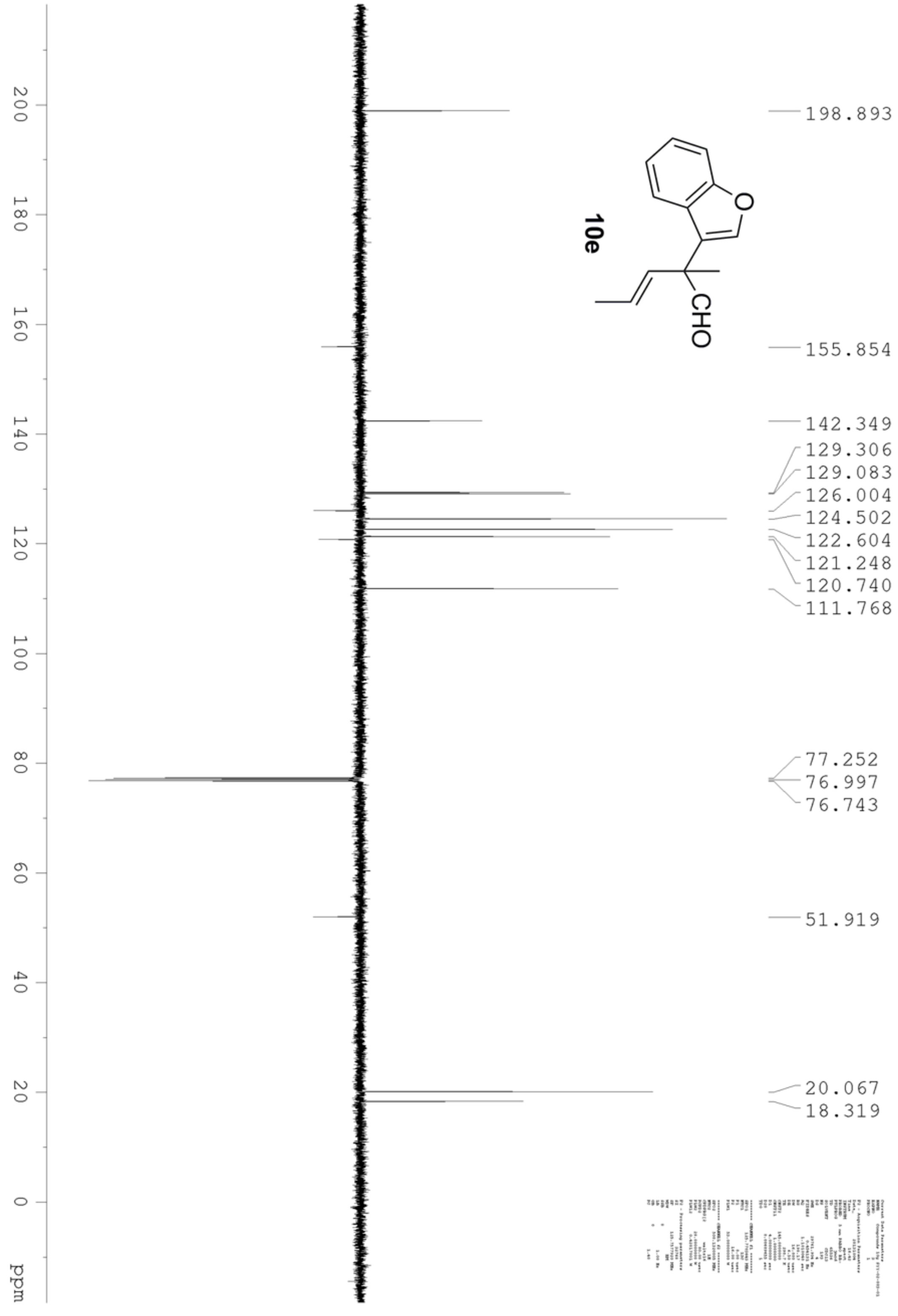

198.893
10e
O
CHO
155.854
142.349
129.306
129.083
126.004
124.502
122.604
121.248
120.740
111.768
77.252
76.997
76.743
51.919
20.067
18.319
200
180
160
140
120
100
80
60
40
20
0
ppm

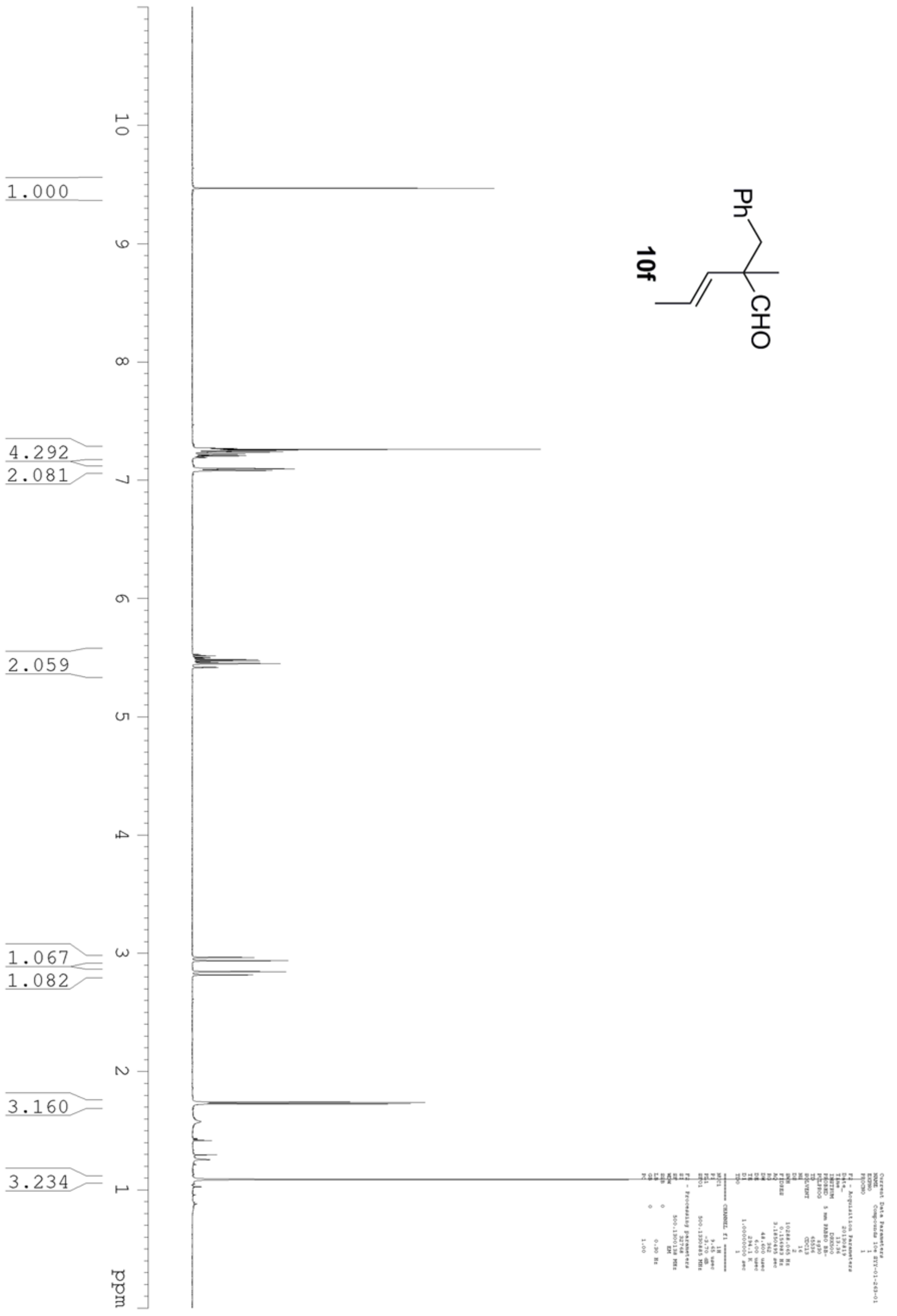

10f
CHO
Ph
1.000
4.292
2.081
2.059
1.067
1.082
3.160
3.234
10
9
8
7
6
5
4
3
2
1
ppm

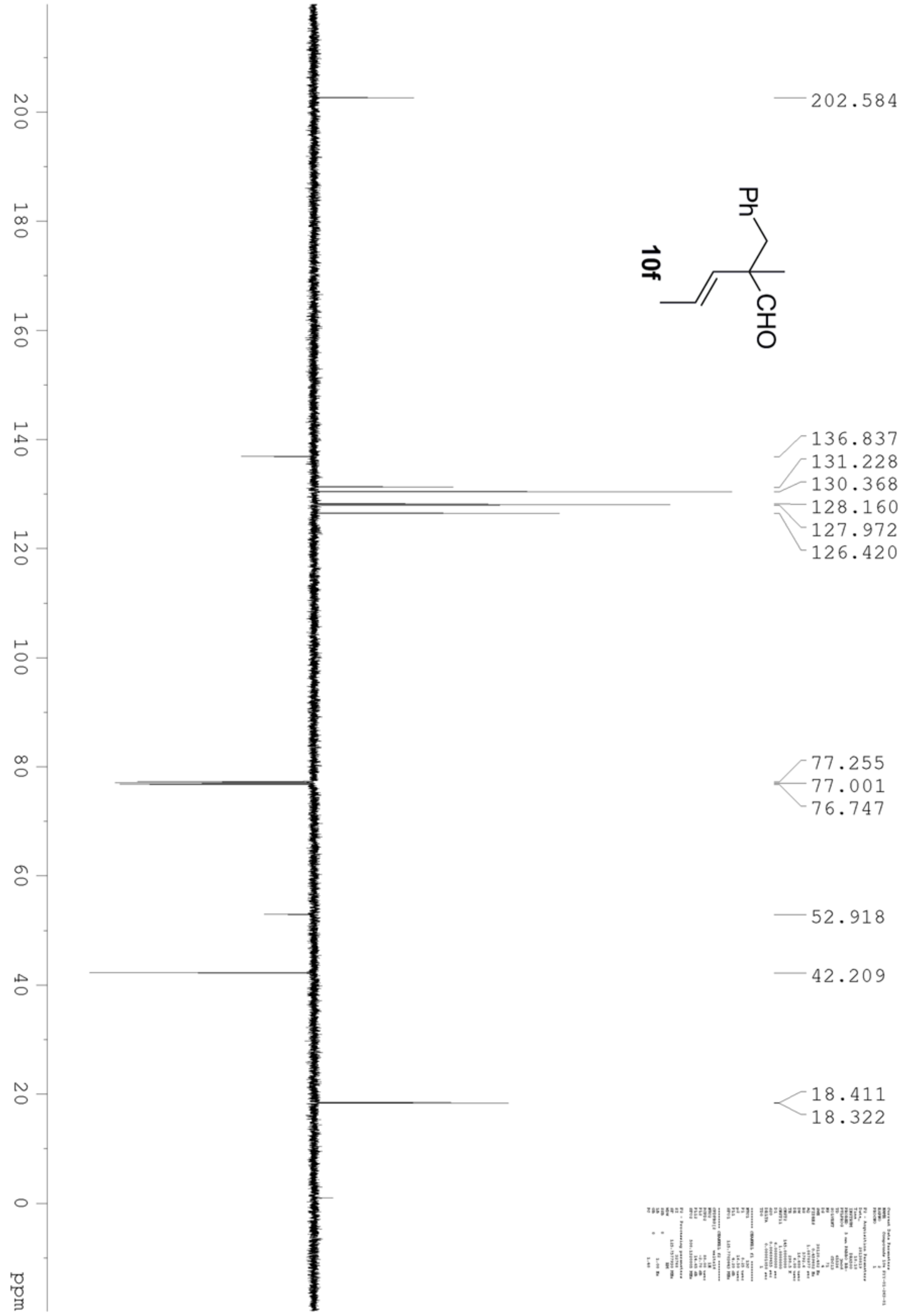

202.584
Ph
CHO
10f
136.837
131.228
130.368
128.160
127.972
126.420
77.255
77.001
76.747
52.918
42.209
18.411
18.322
200
180
160
140
120
100
80
60
40
20
0
ppm

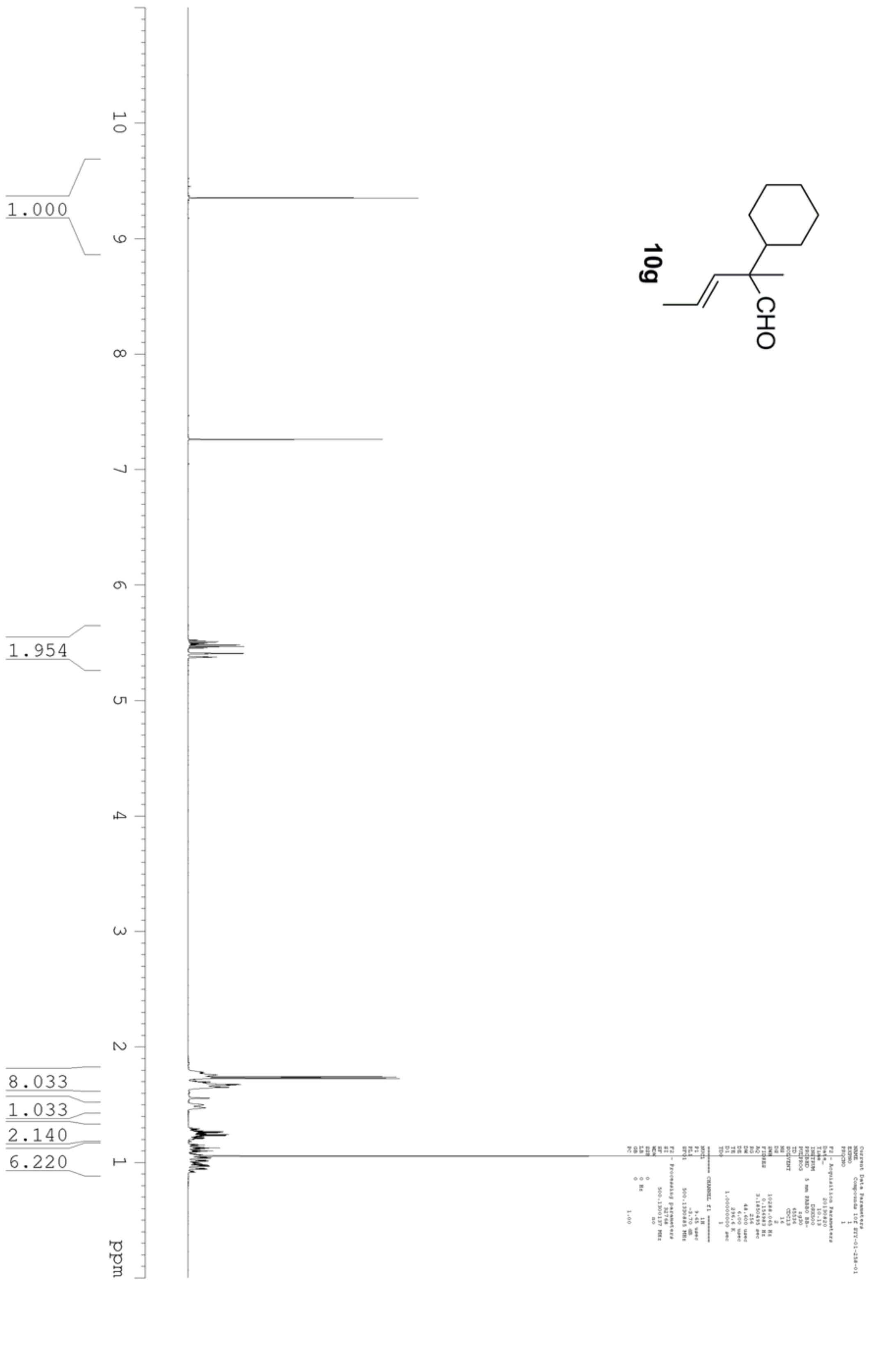
10g
CHO
1.000
1.954
8.033
1.033
2.140
6.220
10
9
8
7
6
5
4
3
2
1
ppm

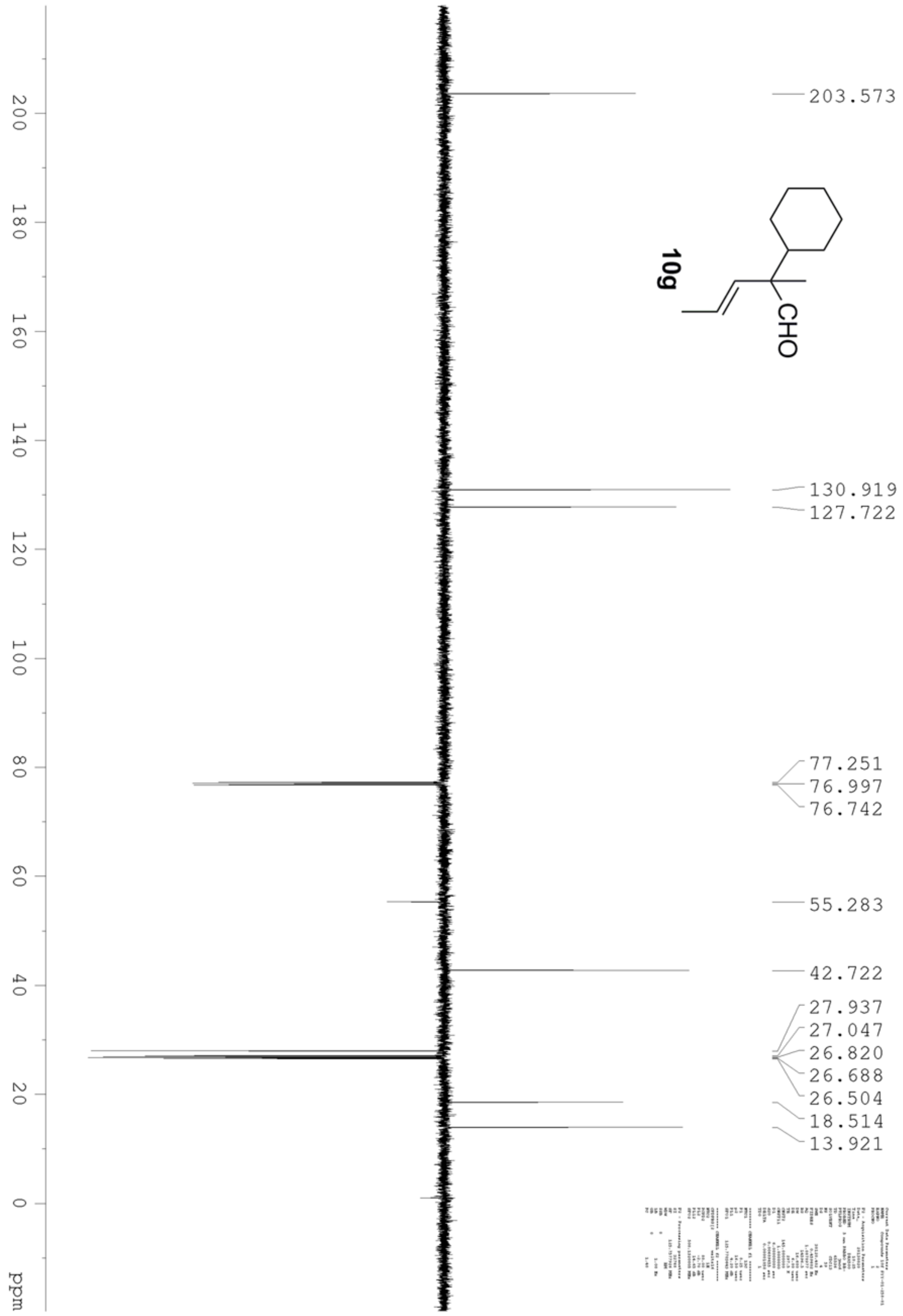

203.573
10g
CHO
130.919
127.722
77.251
76.997
76.742
55.283
42.722
27.937
27.047
26.820
26.688
26.504
18.514
13.921
ppm

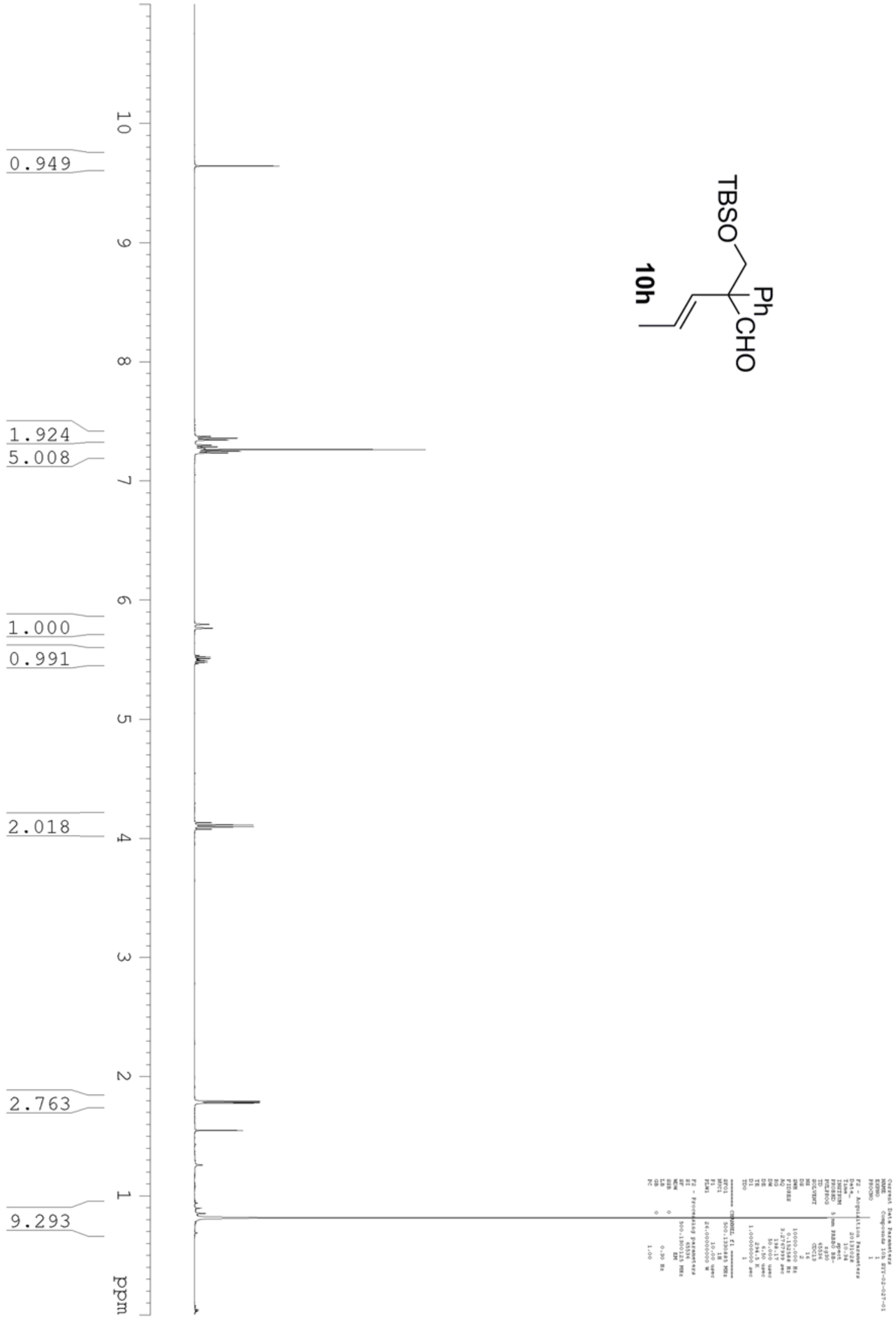
TBSO
Ph
CHO
10h
0.949
1.924
5.008
1.000
0.991
2.018
2.763
9.293
ppm

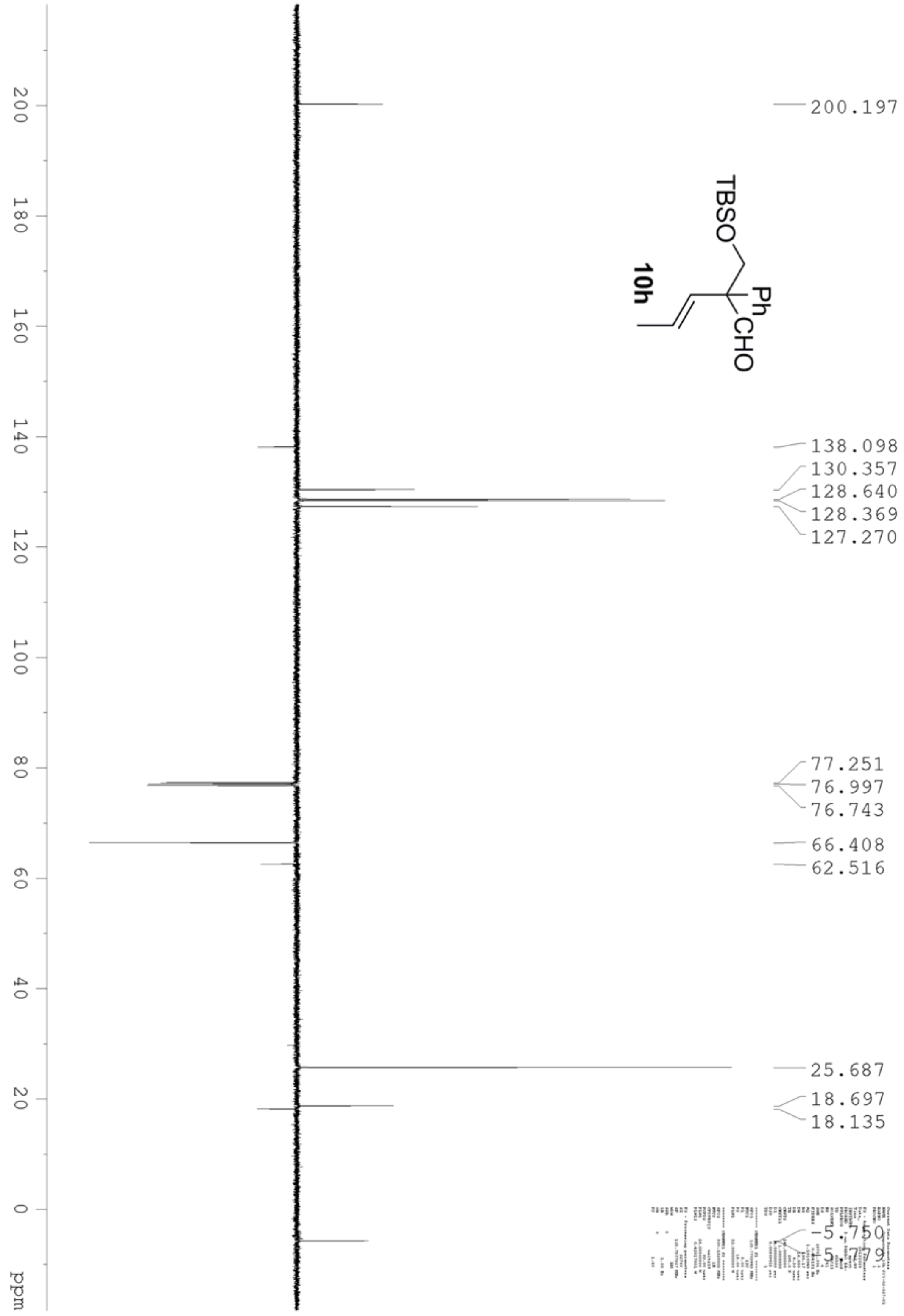

200.197
TBSO
Ph
CHO
10h
138.098
130.357
128.640
128.369
127.270
77.251
76.997
76.743
66.408
62.516
25.687
18.697
18.135
-5.750
ppm

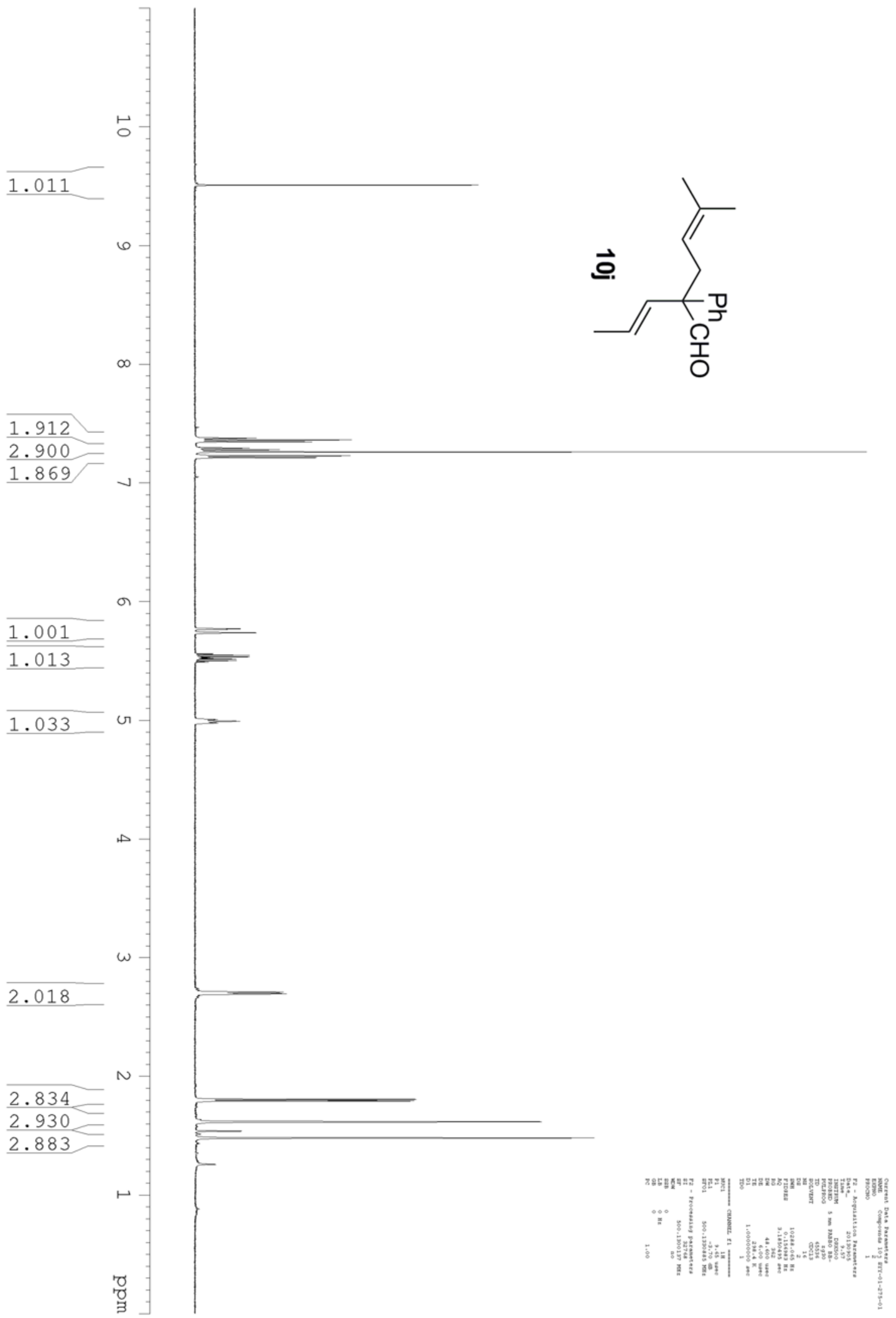
10j
Ph
CHO
1.011
1.912
2.900
1.869
1.001
1.013
1.033
2.018
2.834
2.930
2.883
ppm

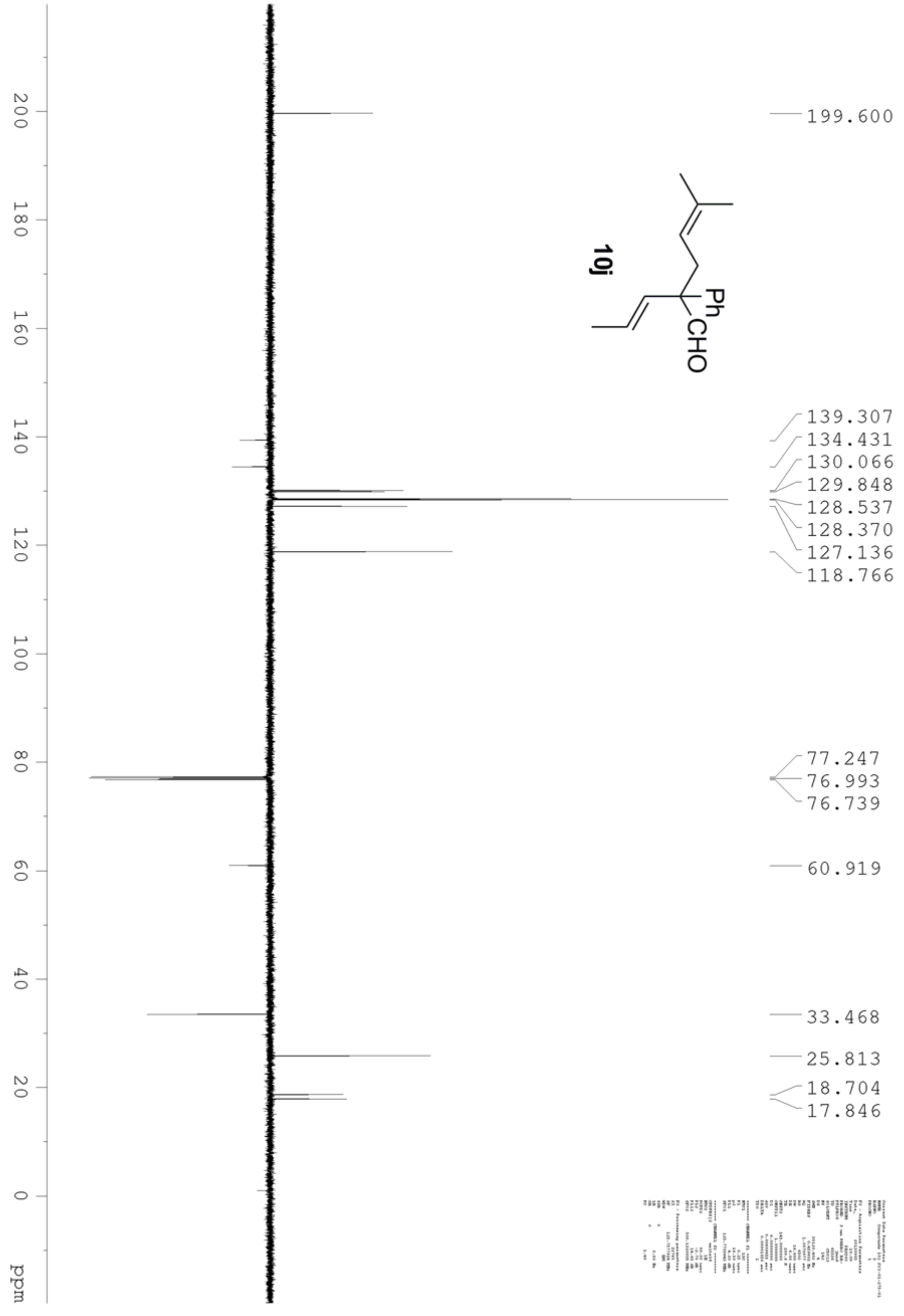
199.600
139.307
134.431
130.066
129.848
128.537
128.370
127.136
118.766
77.247
76.993
76.739
60.919
33.468
25.813
18.704
17.846
10j
Ph
CHO
200
180
160
140
120
100
80
60
40
20
0
ppm

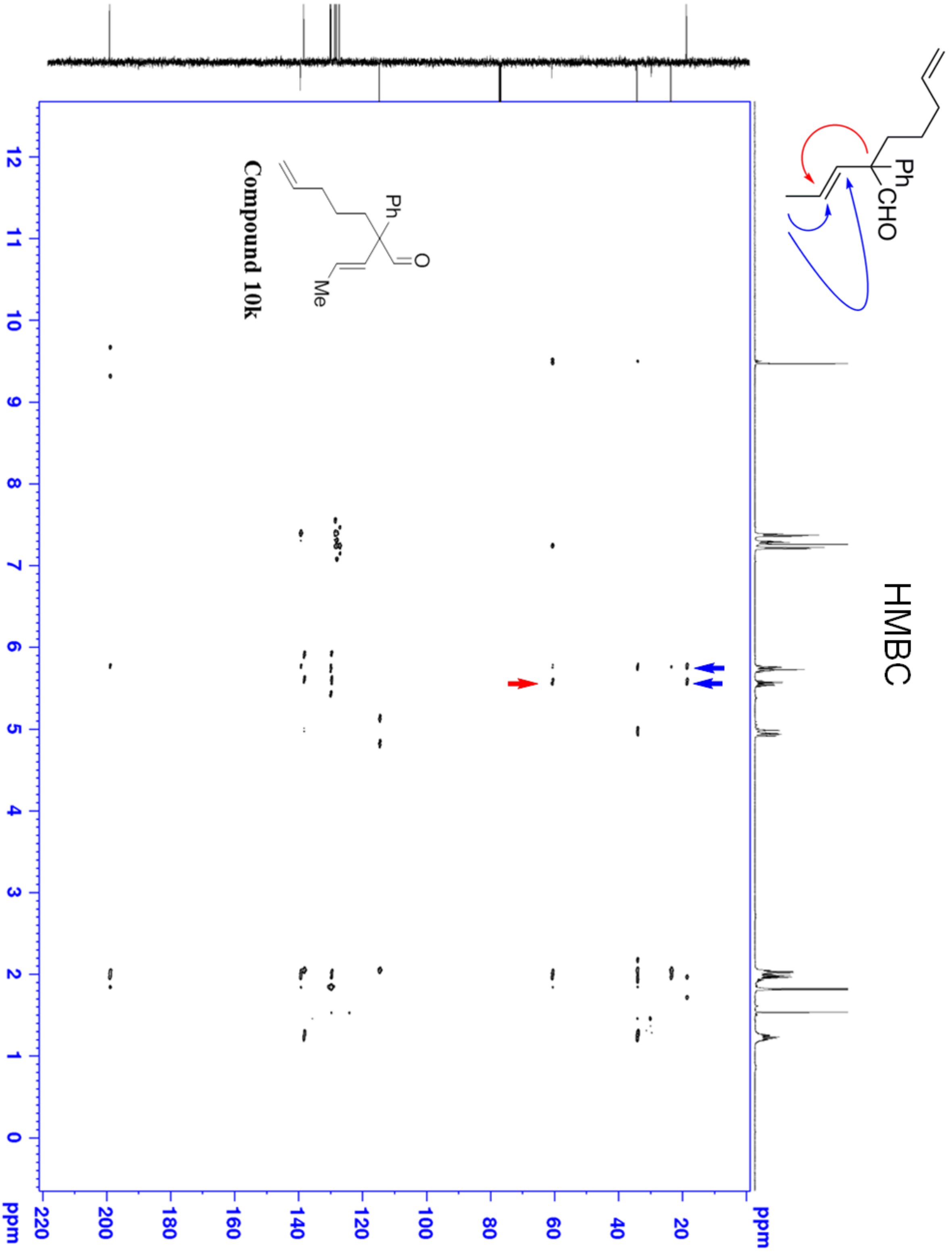

HMBC
Ph
CHO
Ph
O
Me
Compound 10k
ppm
ppm

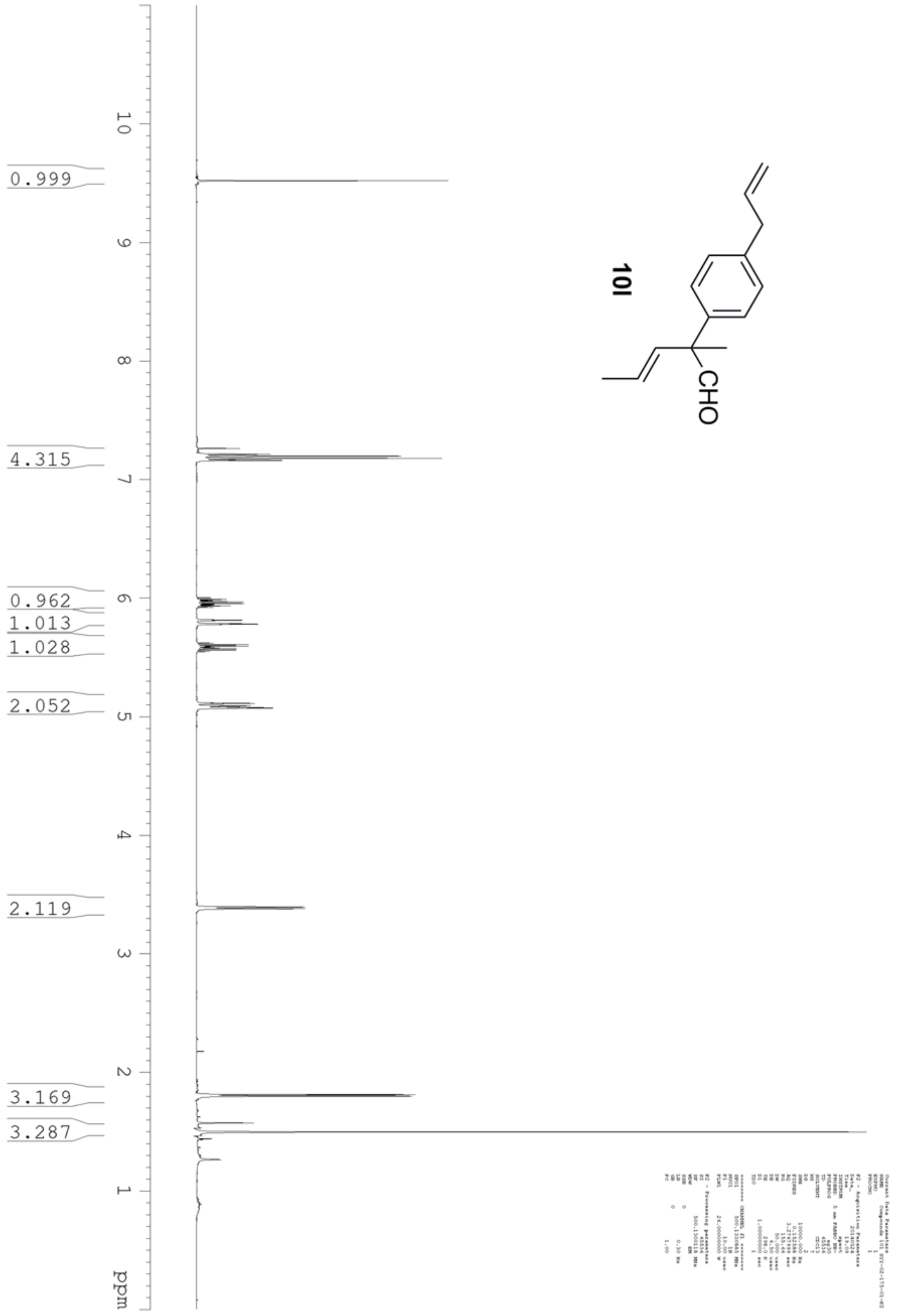

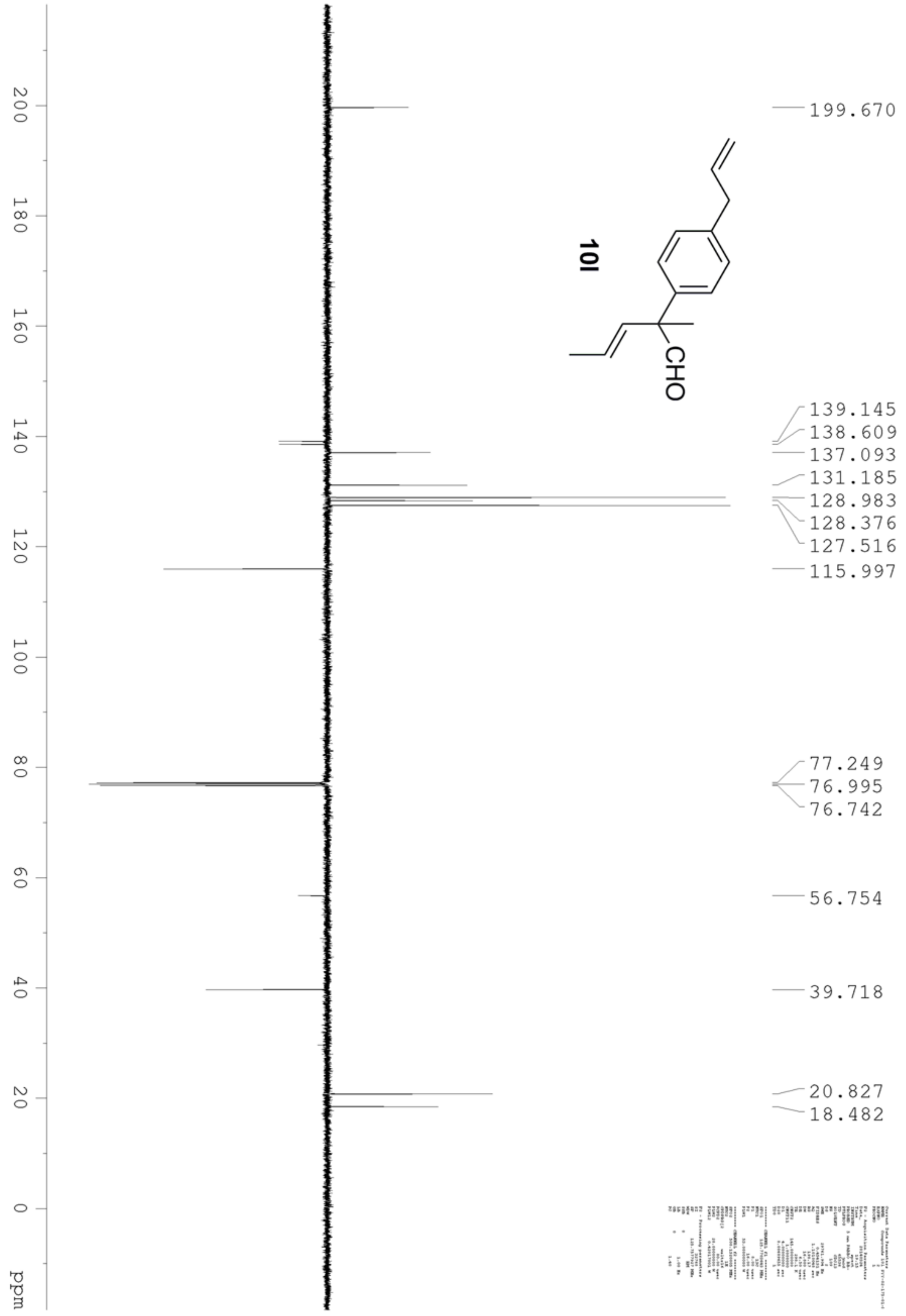
10l
CHO
199.670
139.145
138.609
137.093
131.185
128.983
128.376
127.516
115.997
77.249
76.995
76.742
56.754
39.718
20.827
18.482
200
180
160
140
120
100
80
60
40
20
0
ppm

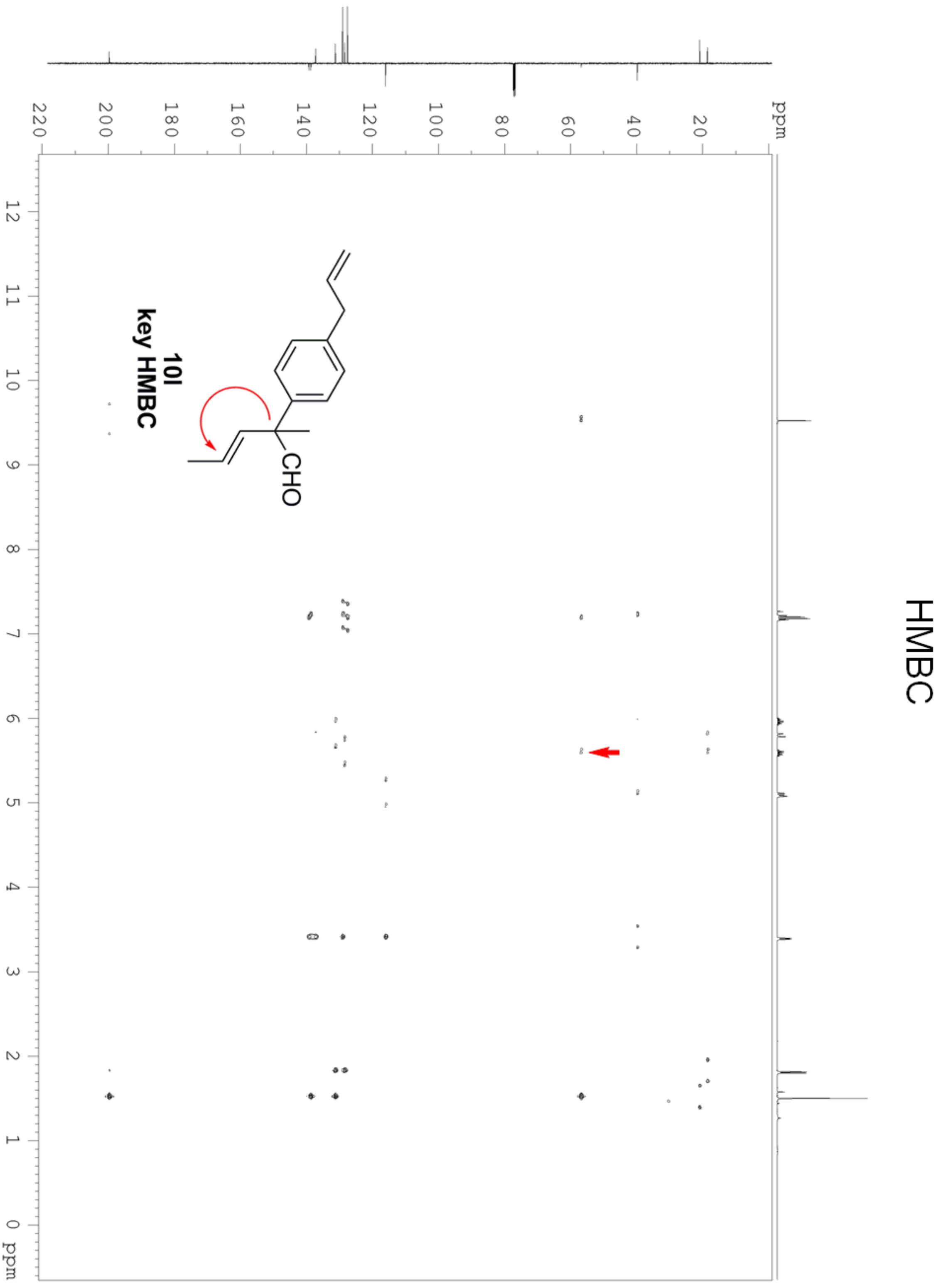
HMBC
CHO
10l
key HMBC
ppm

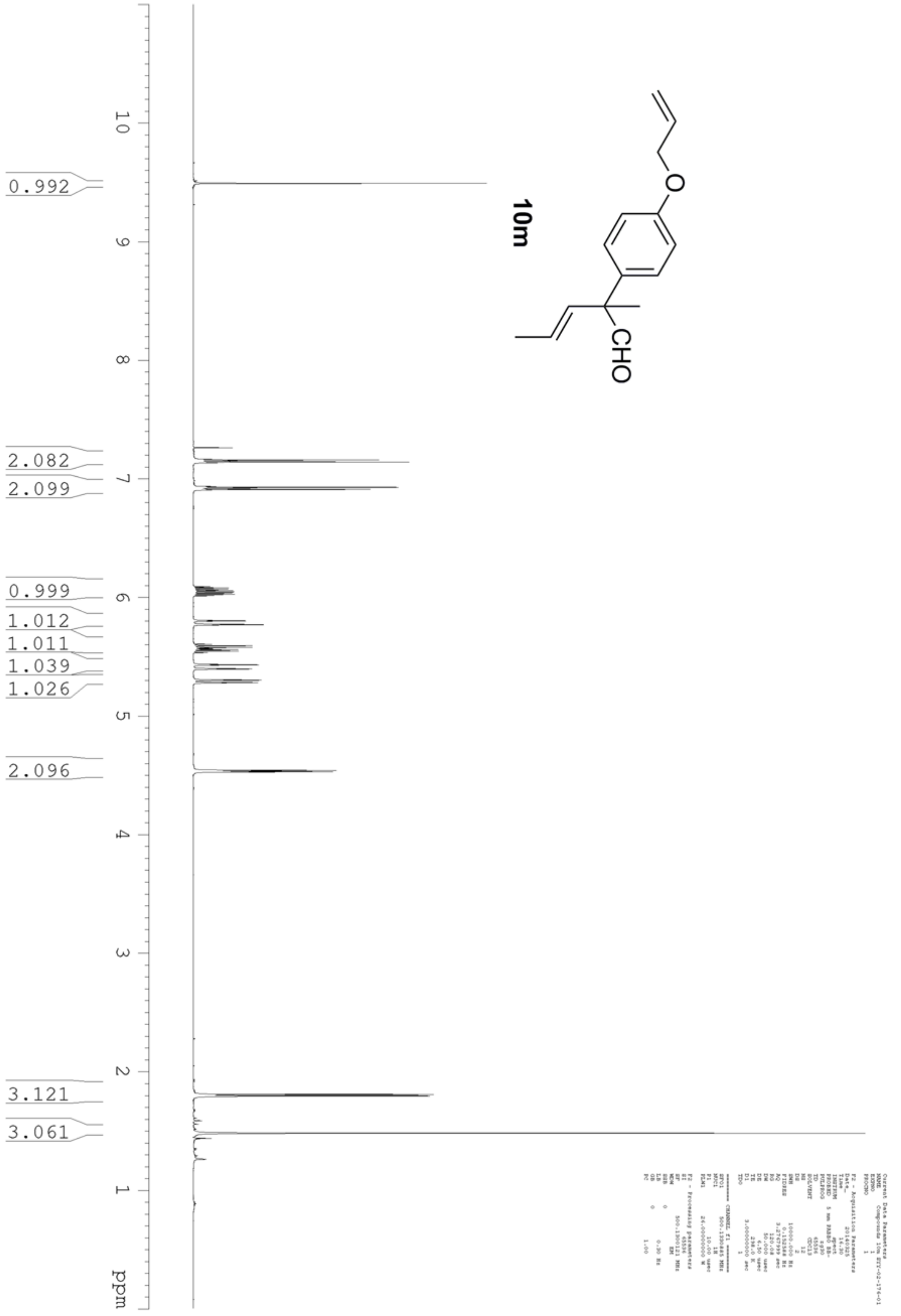

10m
CHO
O
0.992
2.082
2.099
0.999
1.012
1.011
1.039
1.026
2.096
3.121
3.061
10
9
8
7
6
5
4
3
2
1
ppm

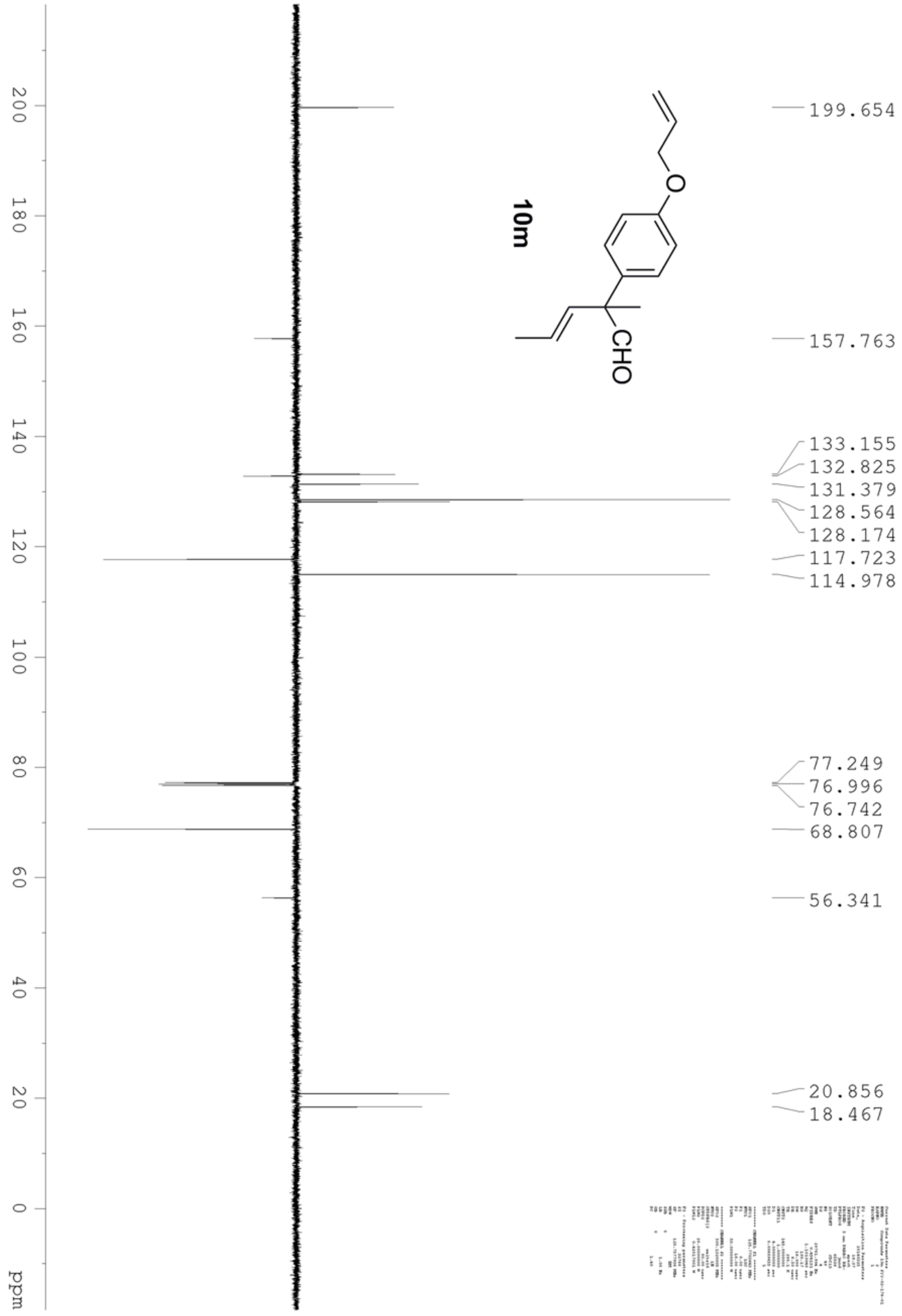
10m
O
CHO
199.654
157.763
133.155
132.825
131.379
128.564
128.174
117.723
114.978
77.249
76.996
76.742
68.807
56.341
20.856
18.467
200
180
160
140
120
100
80
60
40
20
0
ppm

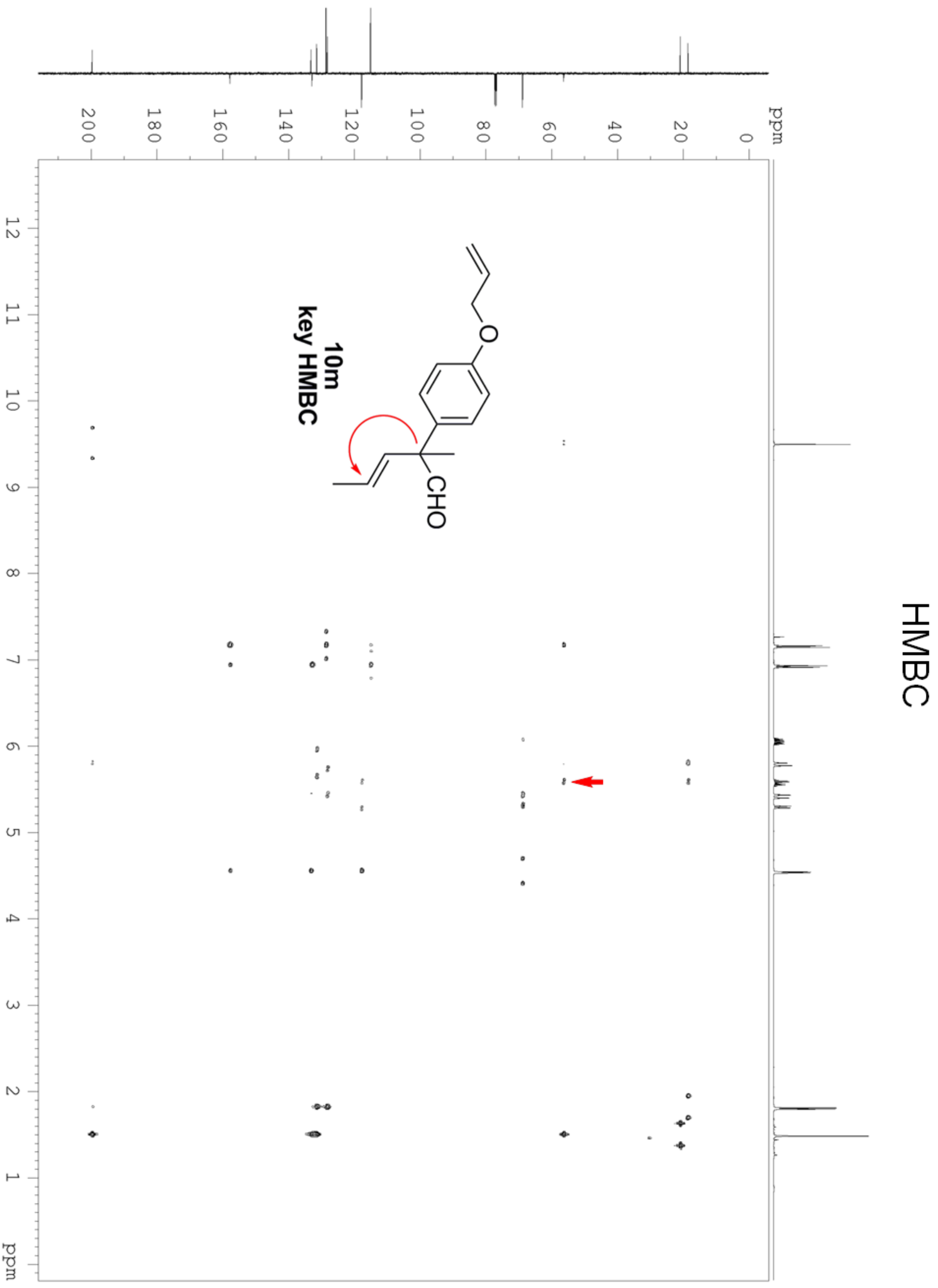

HMBC
10m
key HMBC
CHO
O
ppm

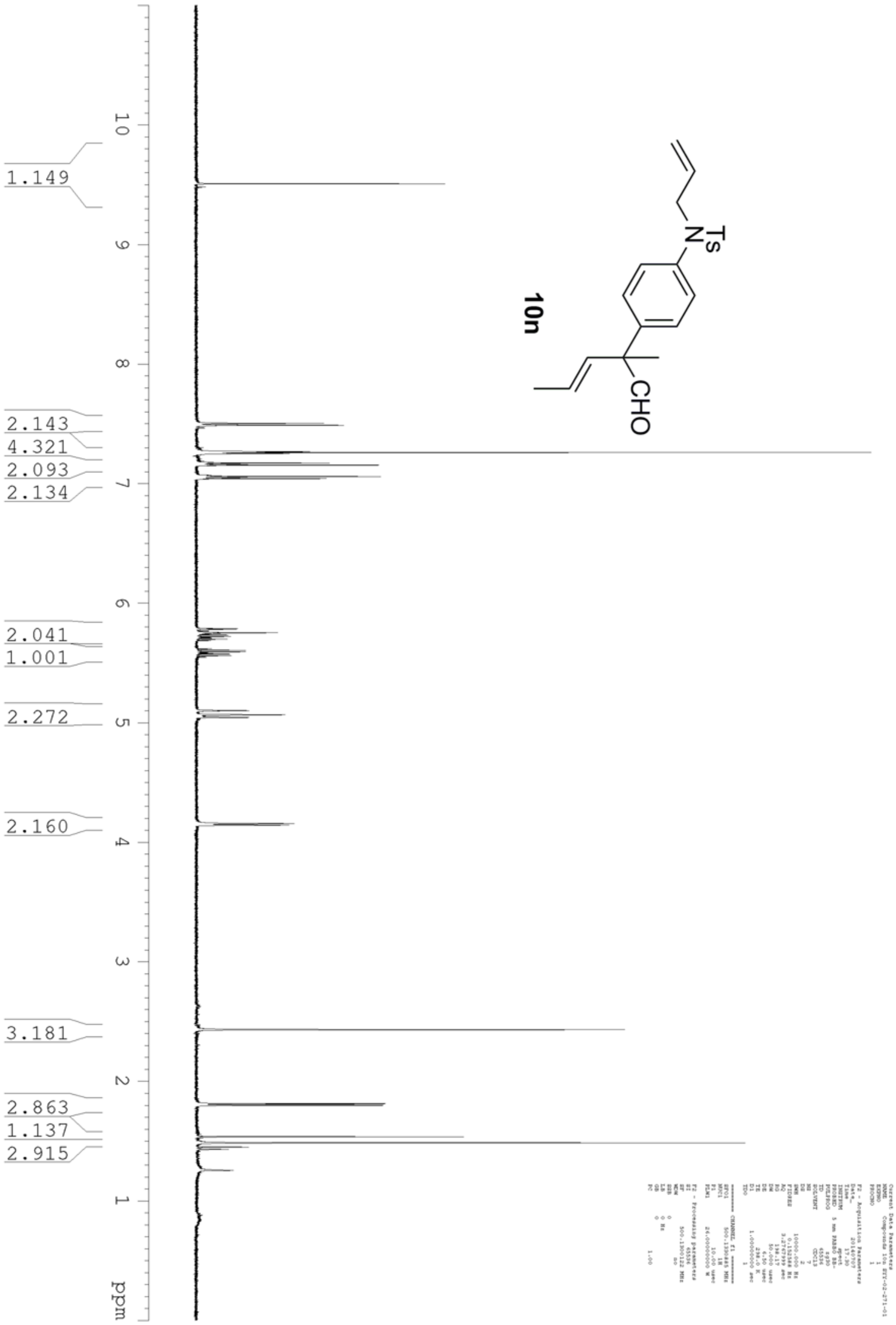
10n
CHO
Ts
N
1.149
2.143
4.321
2.093
2.134
2.041
1.001
2.272
2.160
3.181
2.863
1.137
2.915
10
9
8
7
6
5
4
3
2
1
ppm

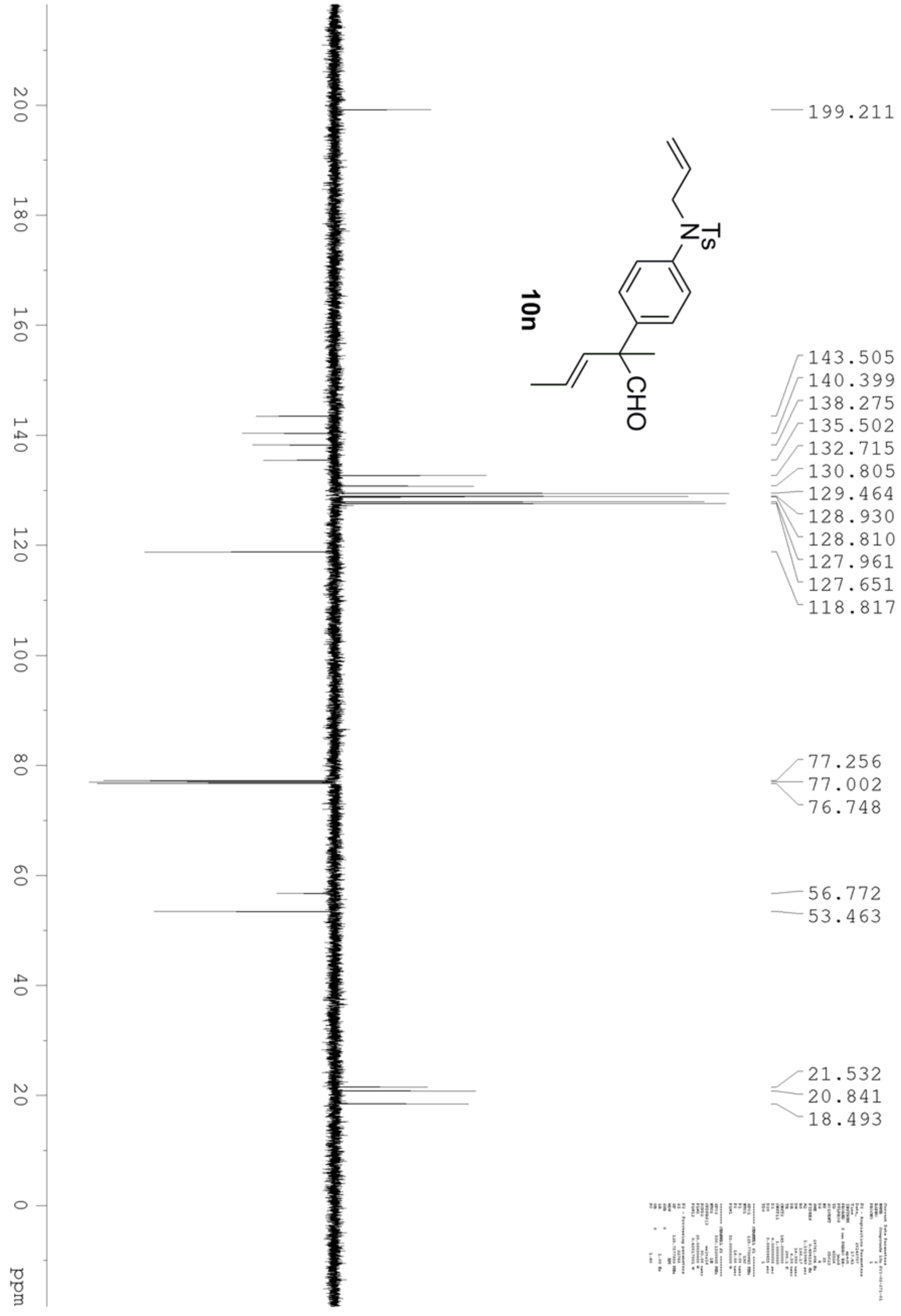
10n
Ts
N
CHO
199.211
143.505
140.399
138.275
135.502
132.715
130.805
129.464
128.930
128.810
127.961
127.651
118.817
77.256
77.002
76.748
56.772
53.463
21.532
20.841
18.493
200
180
160
140
120
100
80
60
40
20
0
ppm

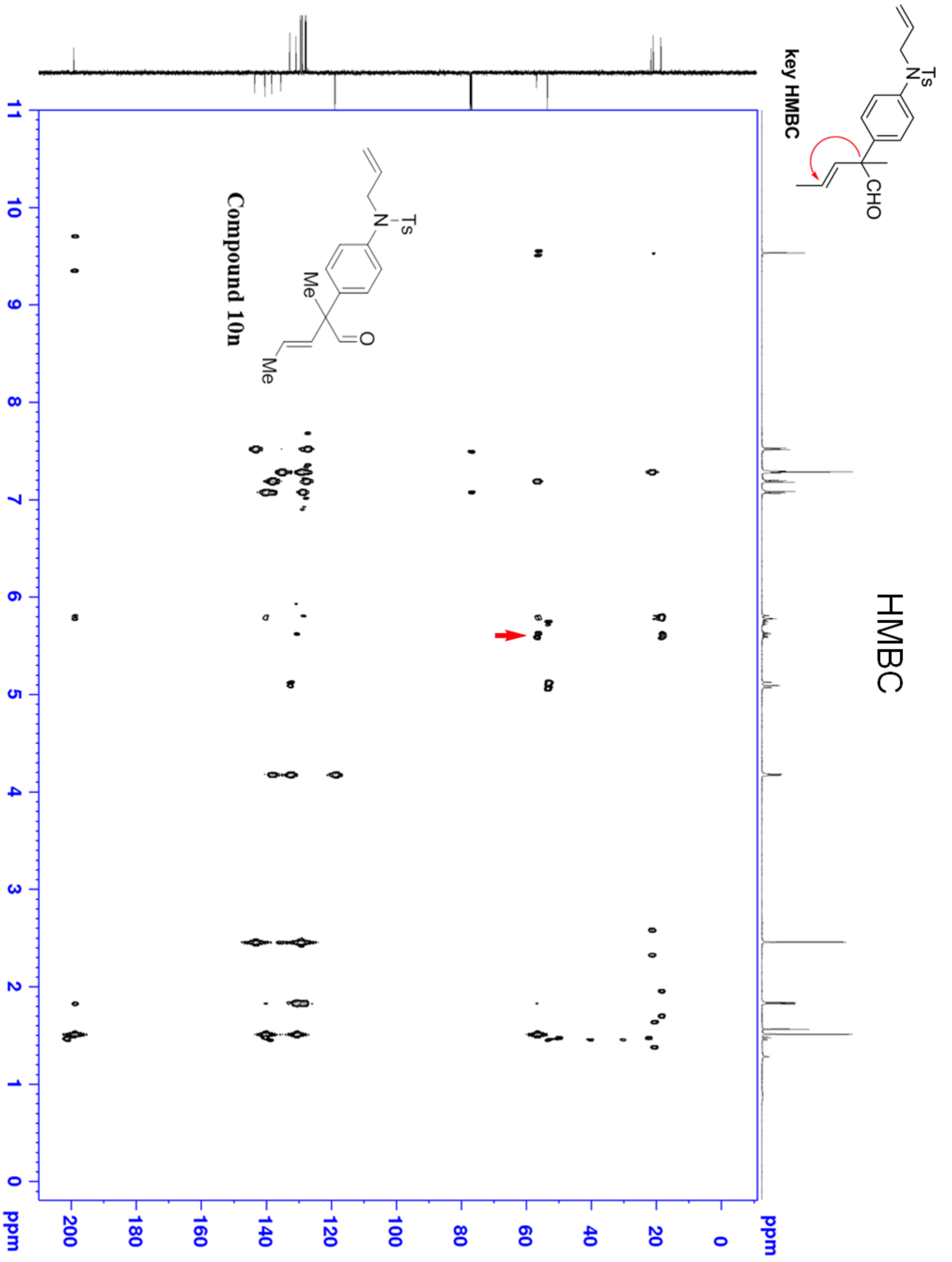
HMBC
key HMBC
CHO
Ts
N
Me
O
Compound 10n
ppm

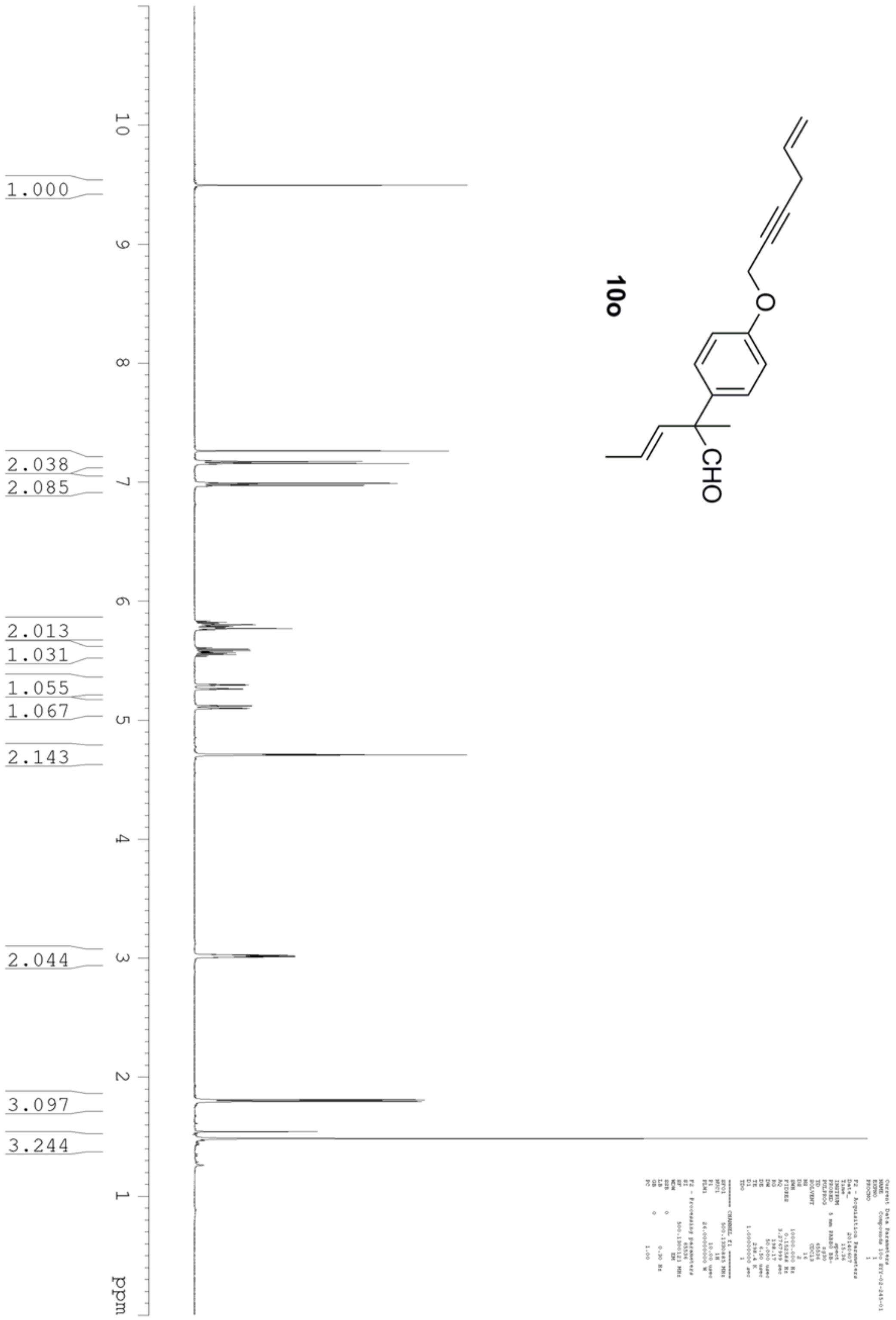
10o
CHO
O
1.000
2.038
2.085
2.013
1.031
1.055
1.067
2.143
2.044
3.097
3.244
10
9
8
7
6
5
4
3
2
1
ppm

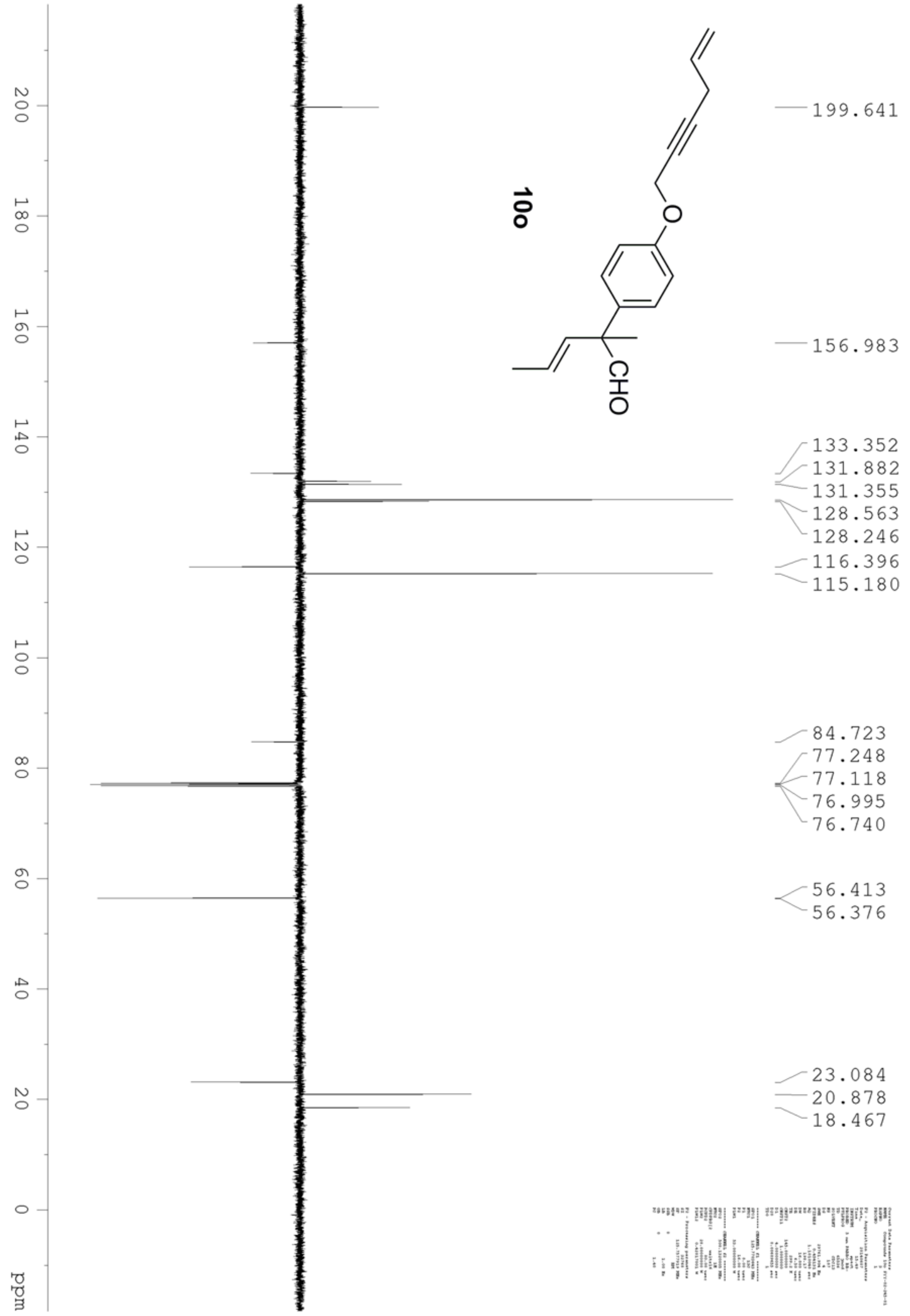
10o
O
CHO
199.641
156.983
133.352
131.882
131.355
128.563
128.246
116.396
115.180
84.723
77.248
77.118
76.995
76.740
56.413
56.376
23.084
20.878
18.467
200
180
160
140
120
100
80
60
40
20
0
ppm

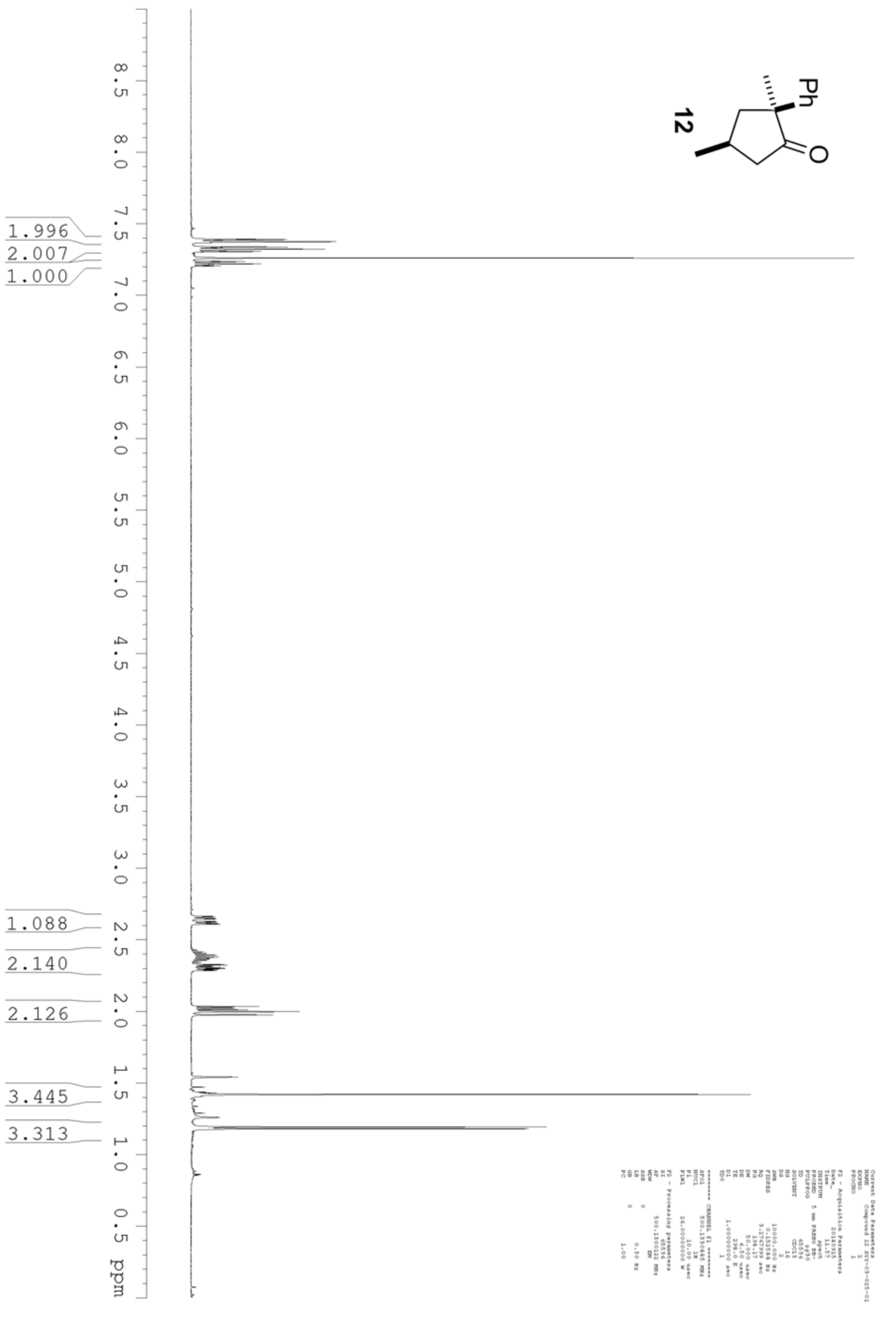
Ph
O
12
8.5 8.0 7.5 7.0 6.5 6.0 5.5 5.0 4.5 4.0 3.5 3.0 2.5 2.0 1.5 1.0 0.5 ppm
1.996
2.007
1.000
1.088
2.140
2.126
3.445
3.313

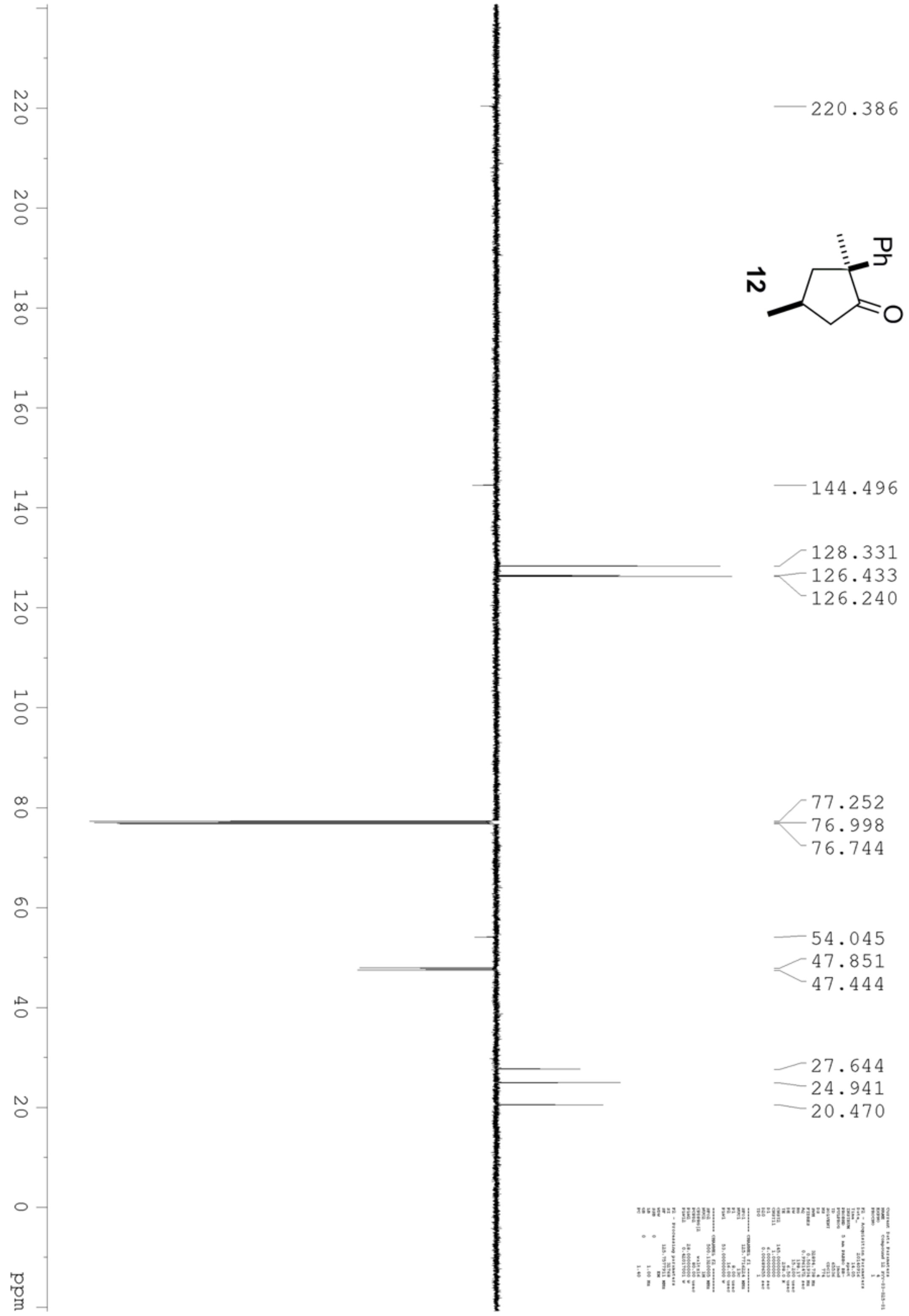

220.386
144.496
128.331
126.433
126.240
77.252
76.998
76.744
54.045
47.851
47.444
27.644
24.941
20.470
Ph
O
12
ppm

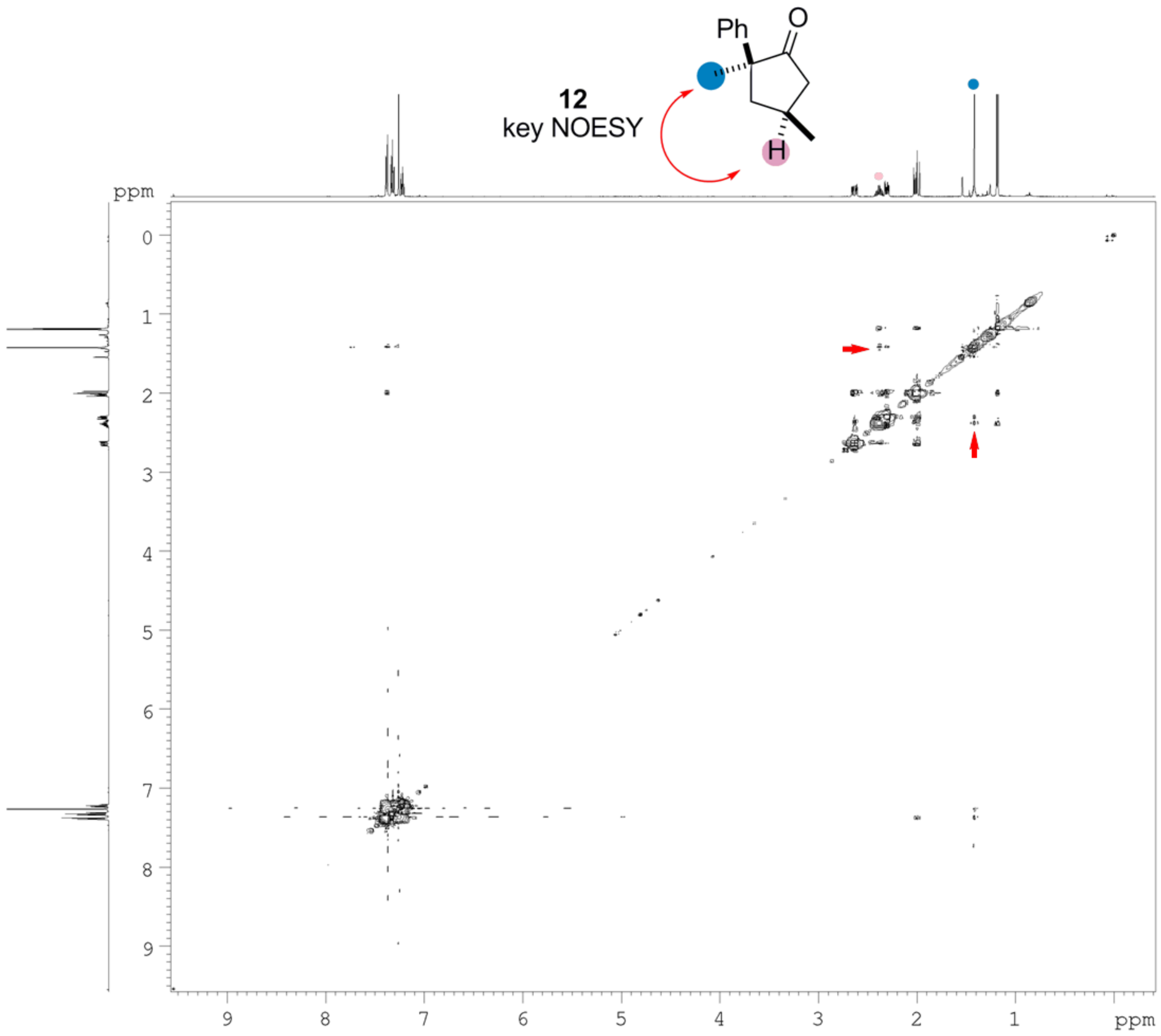

Current Data Parameters
NAME Compound 12 SYY-03-025-01
EXPNO 3
PROCNO 1

F2 - Acquisition Parameters
Date_ 20140915
Time 14.02
INSTRUM spect
PROBHD 5 mm PABBO BB-
PULPROG noesygpph
TD 2048
SOLVENT CDCl3
NS 4
DS 16
SWH 5000.000 Hz
FIDRES 2.441406 Hz
AQ 0.2048000 sec
RG 198.17
DW 100.000 usec
DE 6.50 usec
TE 298.0 K
D0 0.00008727 sec
D1 2.00000000 sec
D8 0.80000001 sec
D16 0.00020000 sec
IN0 0.00020000 sec

======== CHANNEL f1 ========
SFO1 500.1323023 MHz
NUC1 1H
P1 10.00 usec
P2 20.00 usec
PLW1 26.00000000 W

====== GRADIENT CHANNEL =====
GPNAM[1] SMSQ10.100
GPZ1 40.00 %
P16 1000.00 usec

F1 - Acquisition parameters
TD 256
SFO1 500.1323 MHz
FIDRES 19.531250 Hz
SW 9.997 ppm
FnMODE TPPI

F2 - Processing parameters
SI 1024
SF 500.1300133 MHz
WDW QSINE
SSB 2
LB 0 Hz
GB 0
PC 1.00

F1 - Processing parameters
SI 1024
MC2 TPPI
SF 500.1300133 MHz
WDW QSINE
SSB 2
LB 0 Hz
GB 0

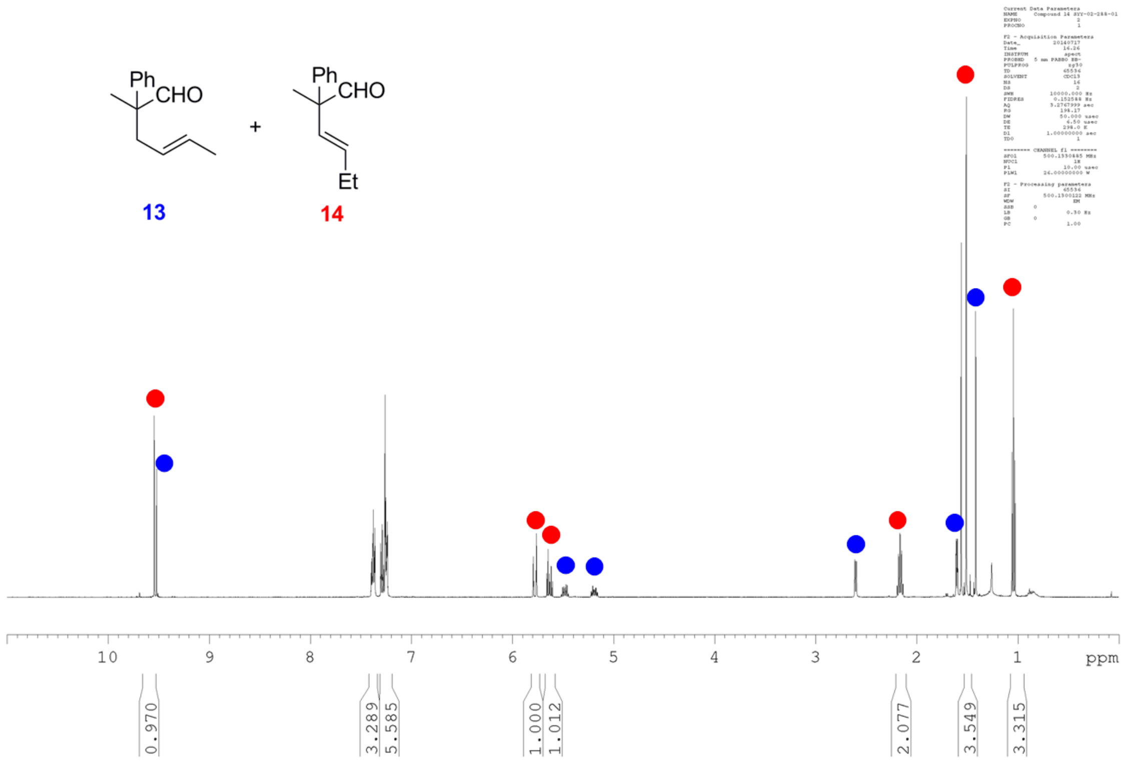

13
14

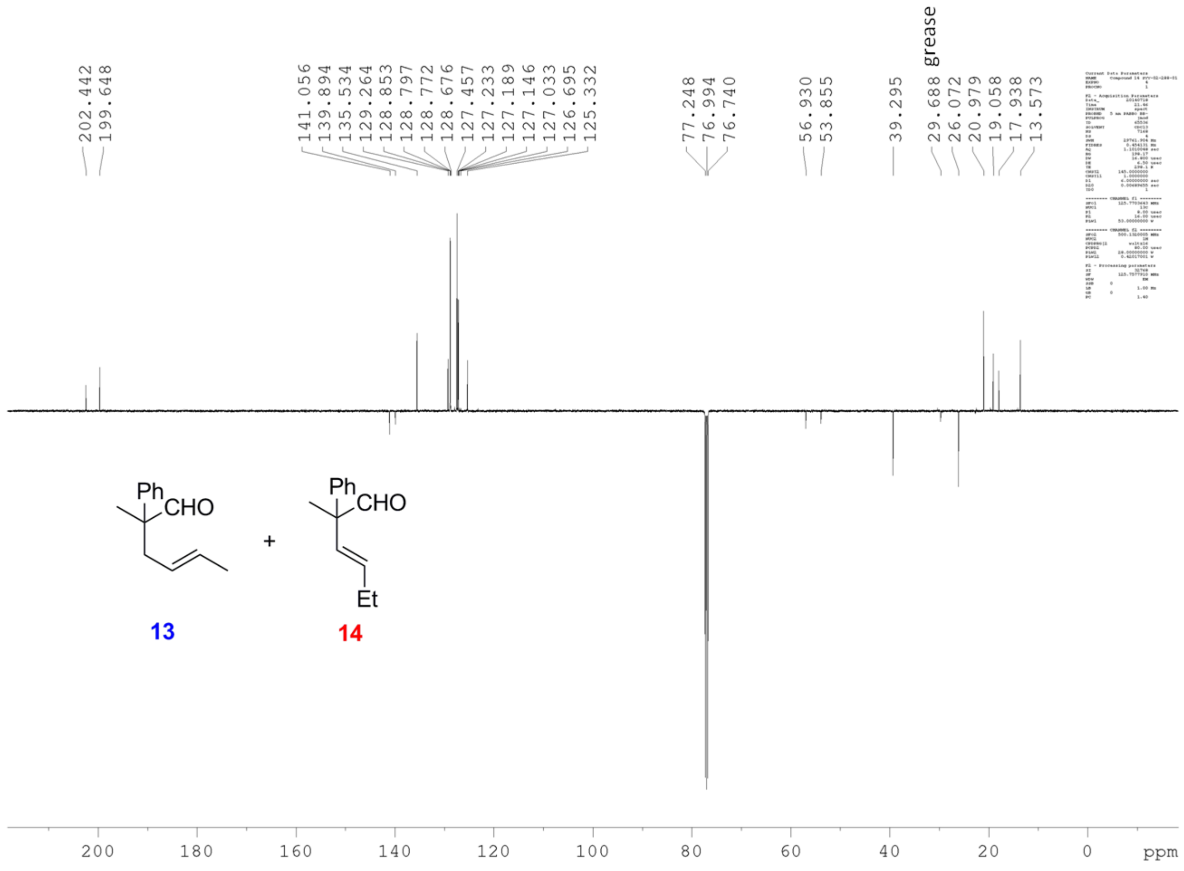

grease
13
14

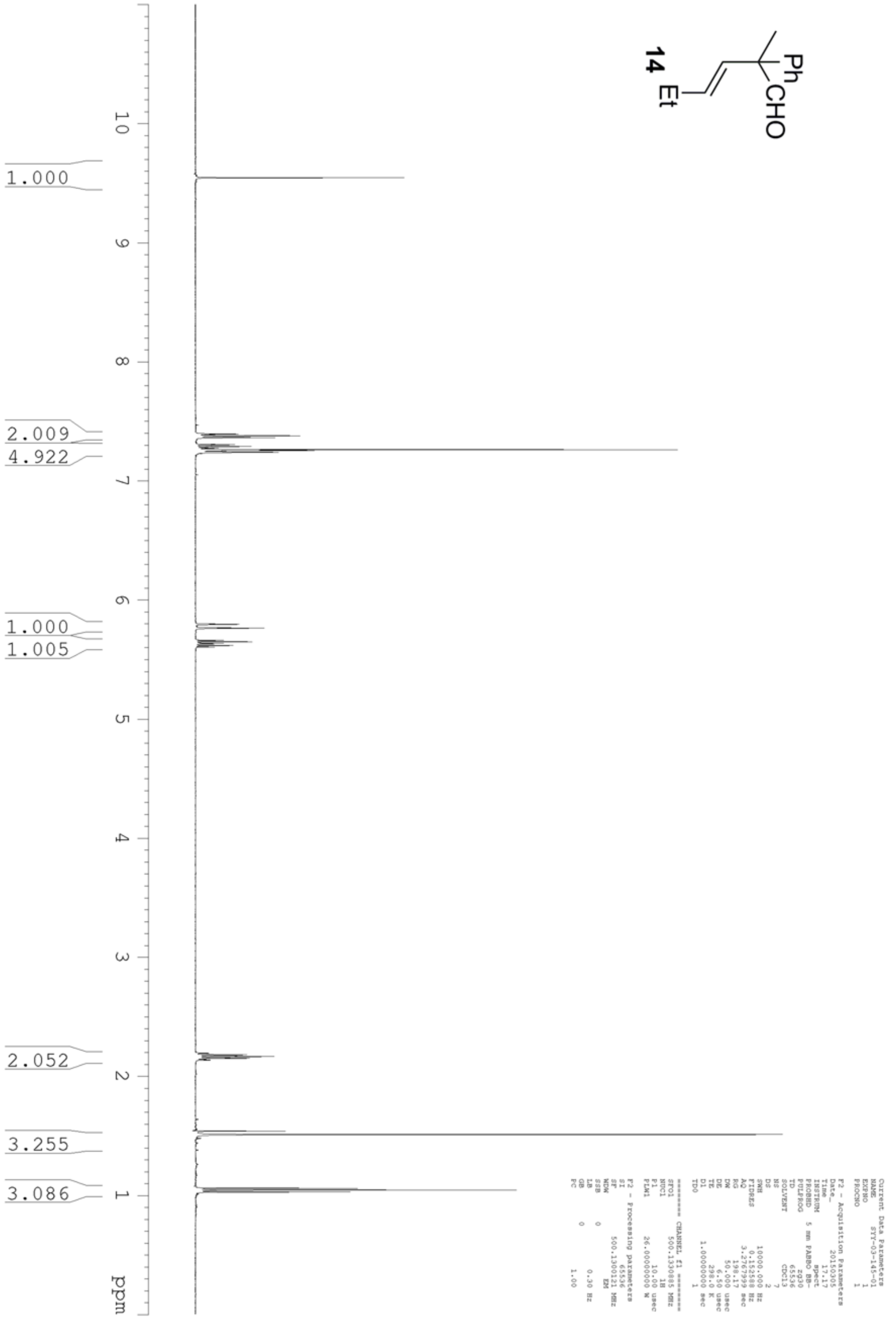
Ph
CHO
Et
14
1.000
2.009
4.922
1.000
1.005
2.052
3.255
3.086
ppm

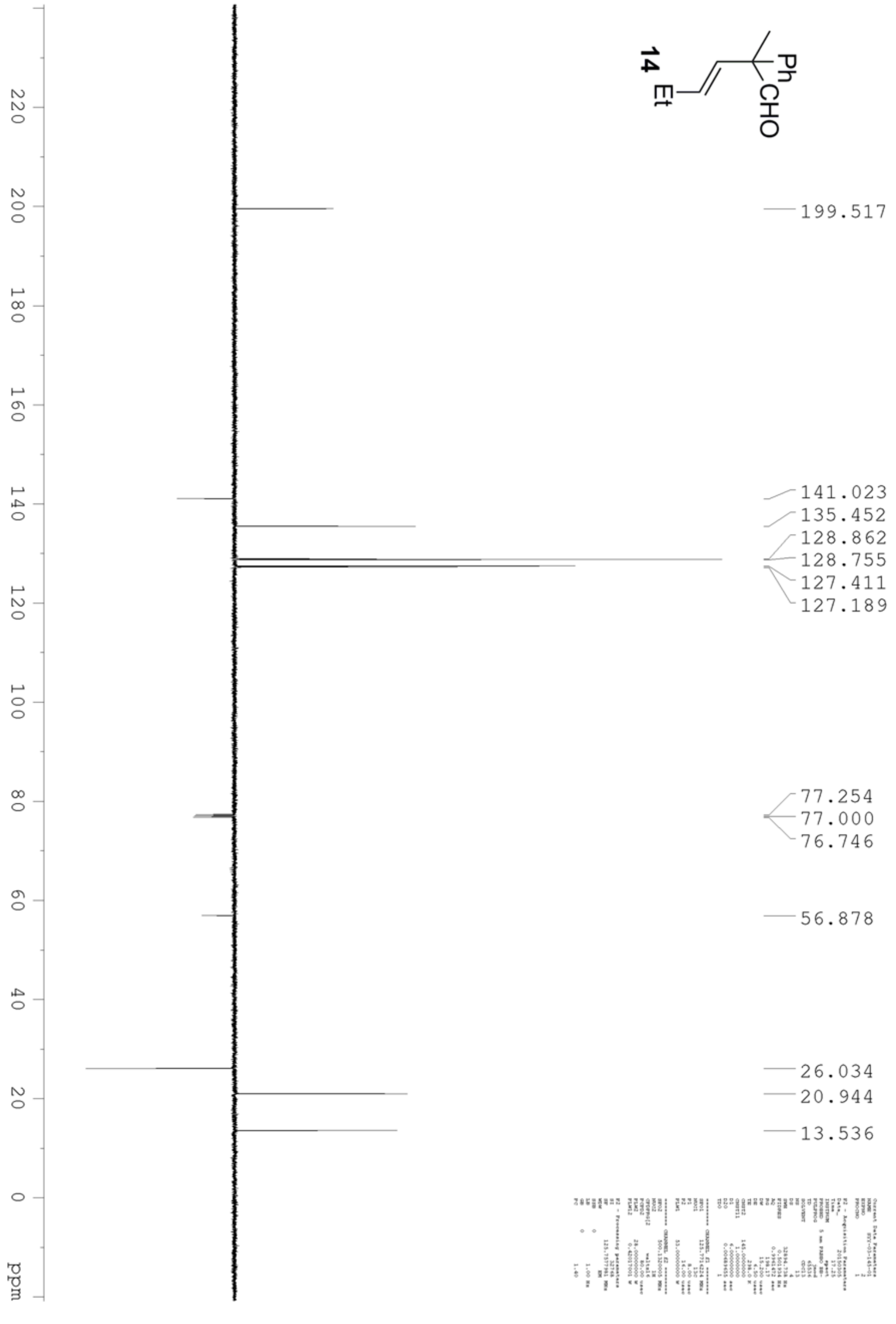

Ph
CHO
14
Et
199.517
141.023
135.452
128.862
128.755
127.411
127.189
77.254
77.000
76.746
56.878
26.034
20.944
13.536
220
200
180
160
140
120
100
80
60
40
20
0
ppm

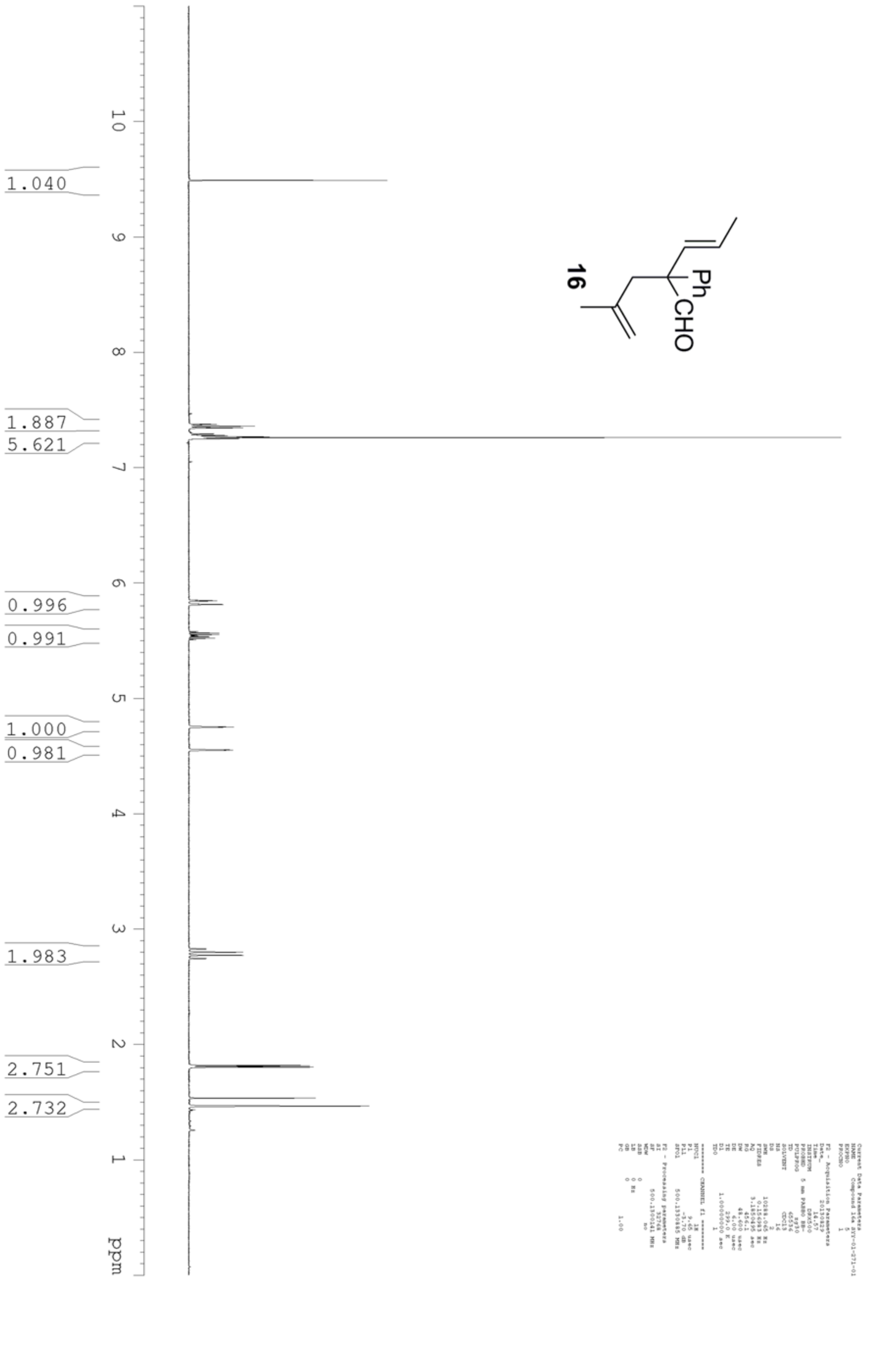

CHO
Ph
16
1.040
1.887
5.621
0.996
0.991
1.000
0.981
1.983
2.751
2.732
10
9
8
7
6
5
4
3
2
1
ppm

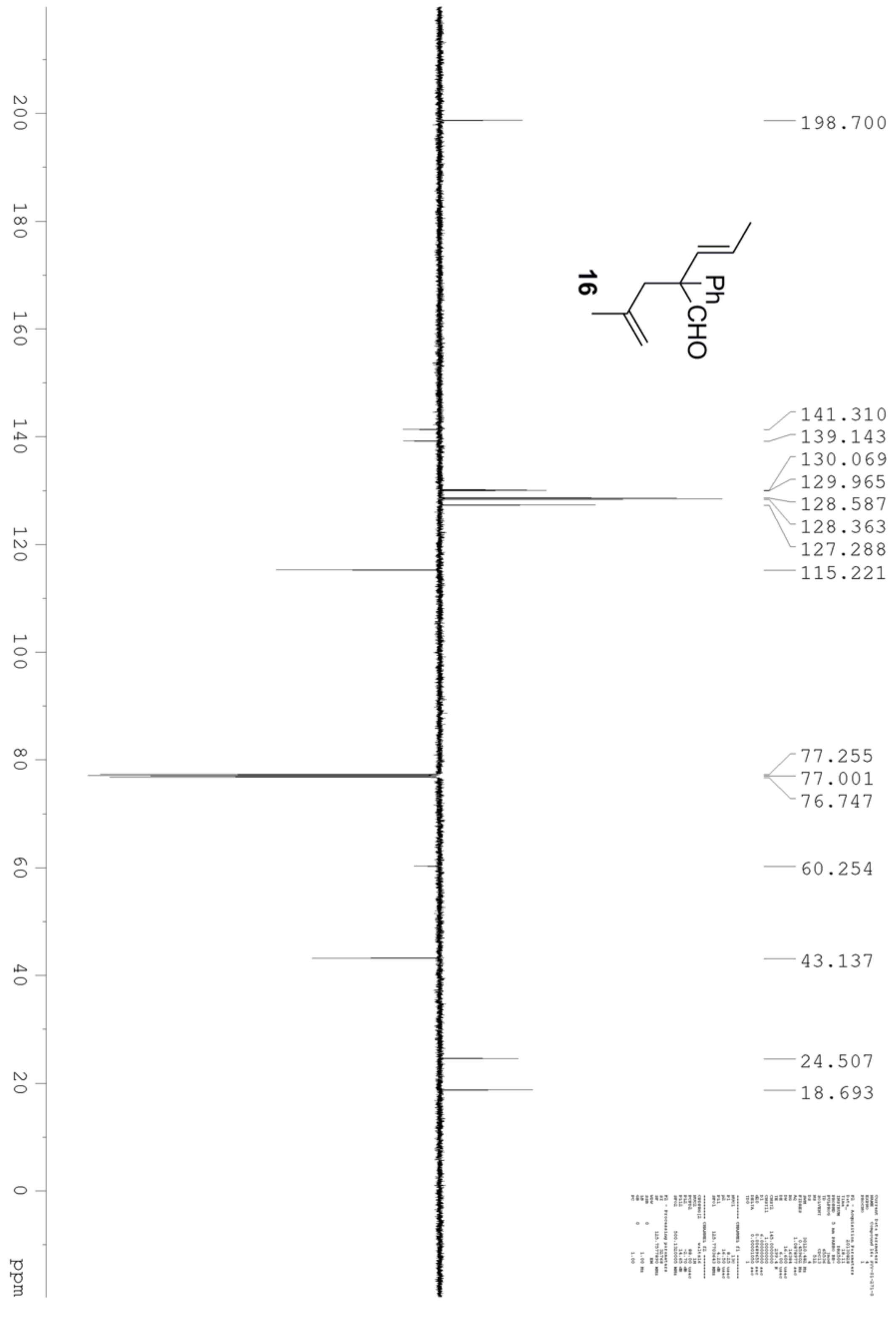

198.700
141.310
139.143
130.069
129.965
128.587
128.363
127.288
115.221
77.255
77.001
76.747
60.254
43.137
24.507
18.693
Ph
CHO
16
200
180
160
140
120
100
80
60
40
20
0
ppm

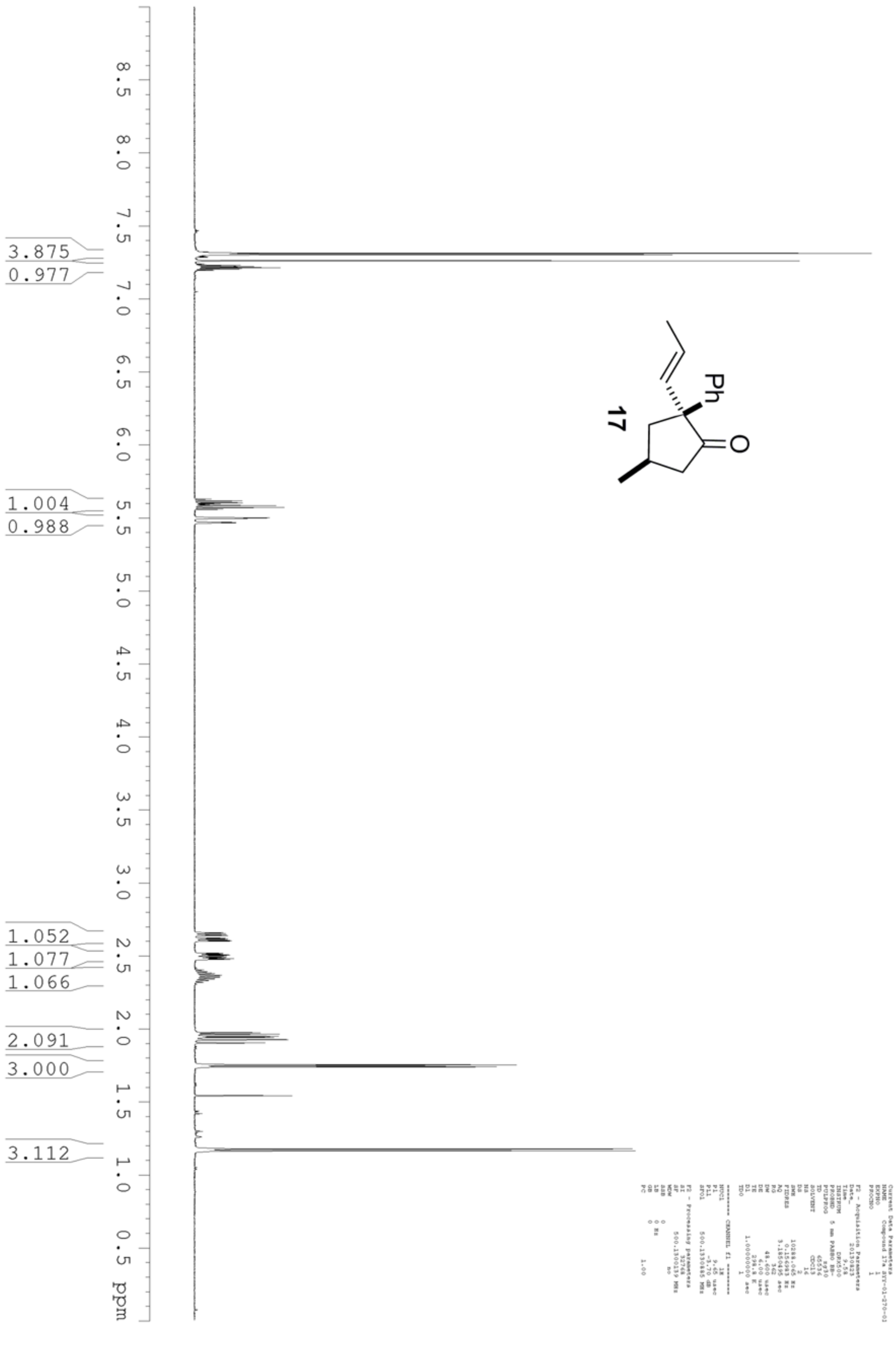
Ph
O
17
3.875
0.977
1.004
0.988
1.052
1.077
1.066
2.091
3.000
3.112
8.5
8.0
7.5
7.0
6.5
6.0
5.5
5.0
4.5
4.0
3.5
3.0
2.5
2.0
1.5
1.0
0.5 ppm

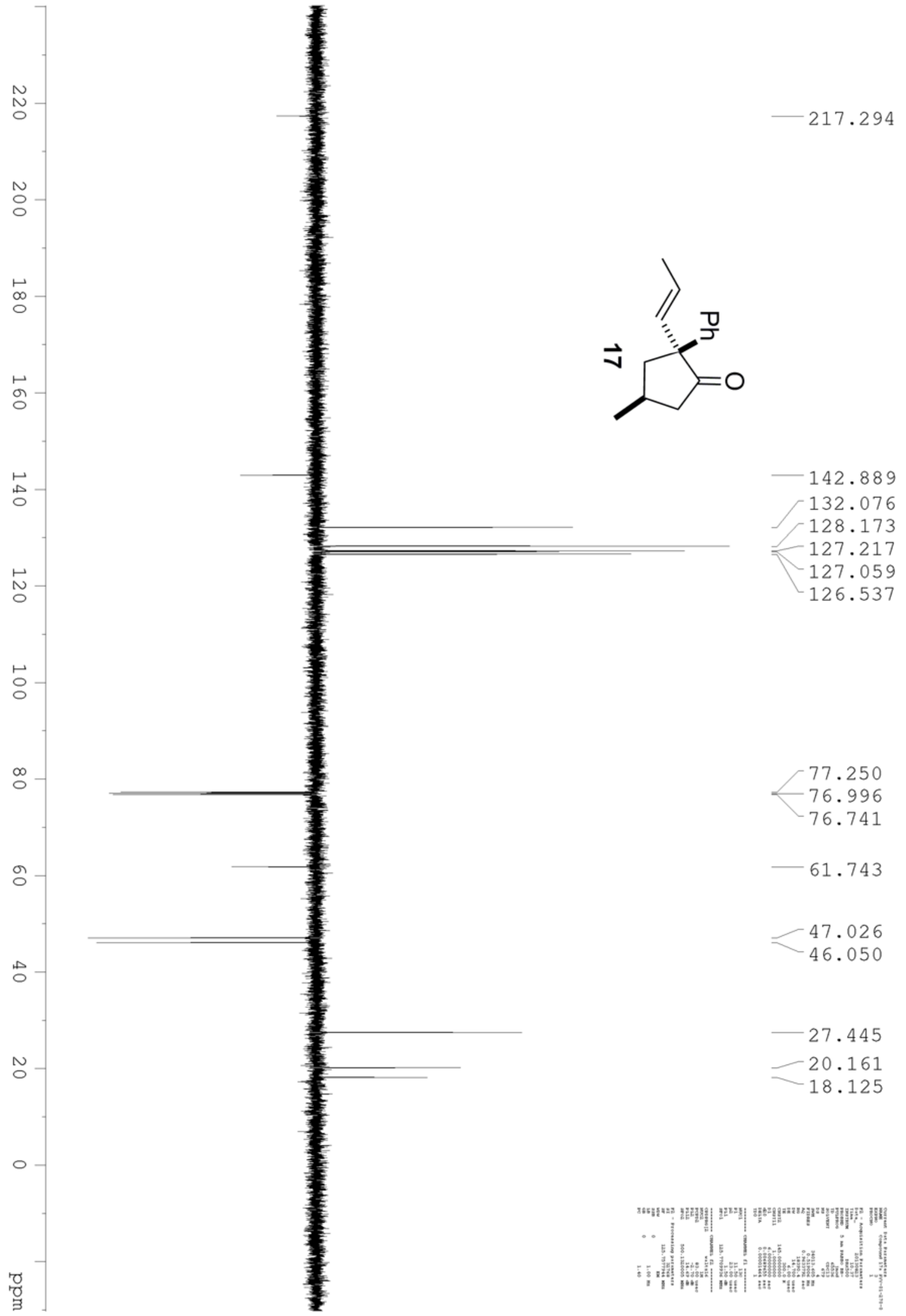

217.294
142.889
132.076
128.173
127.217
127.059
126.537
77.250
76.996
76.741
61.743
47.026
46.050
27.445
20.161
18.125
Ph
O
17
ppm

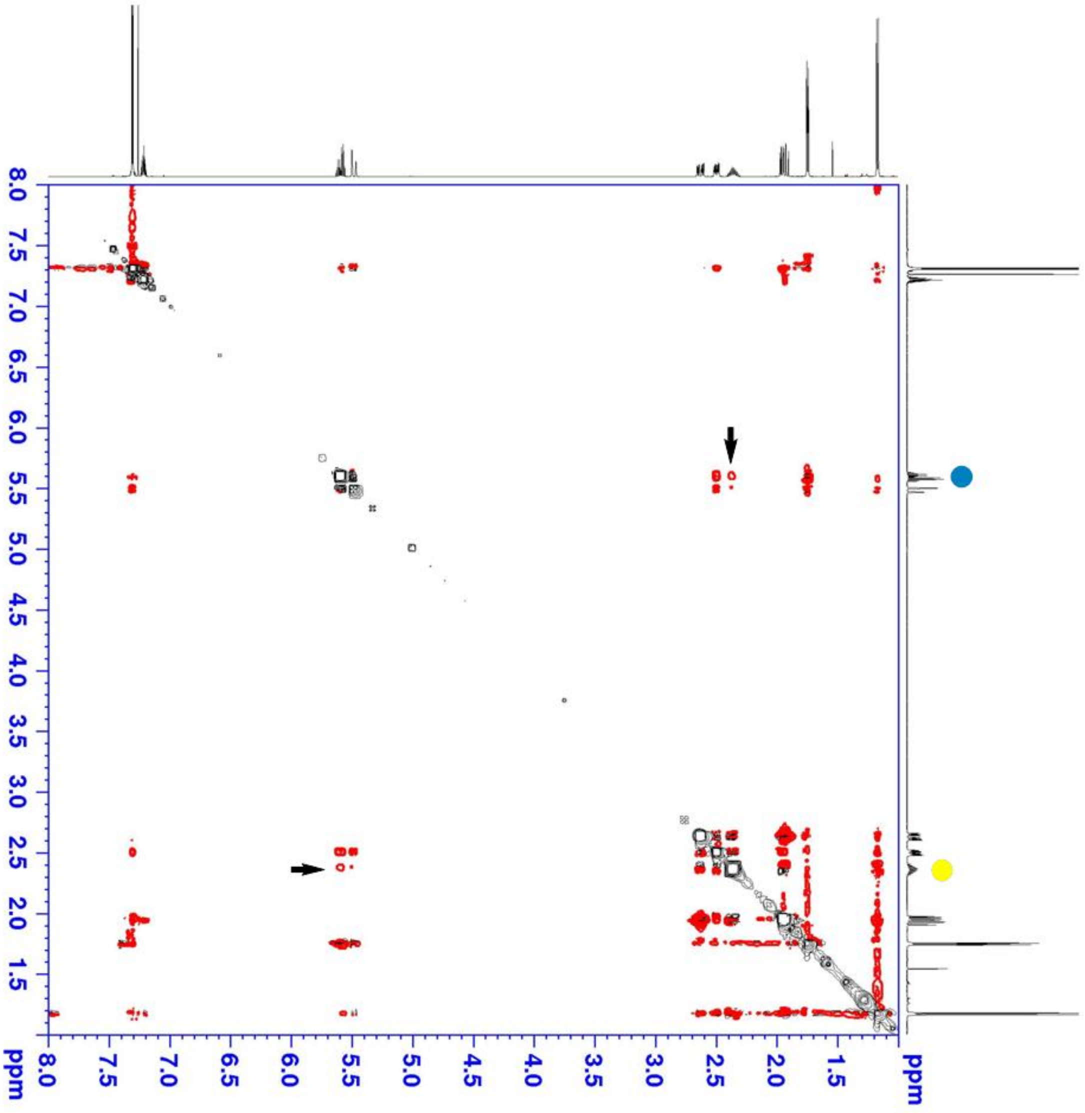

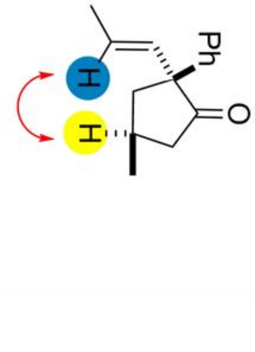

17
key NOESY

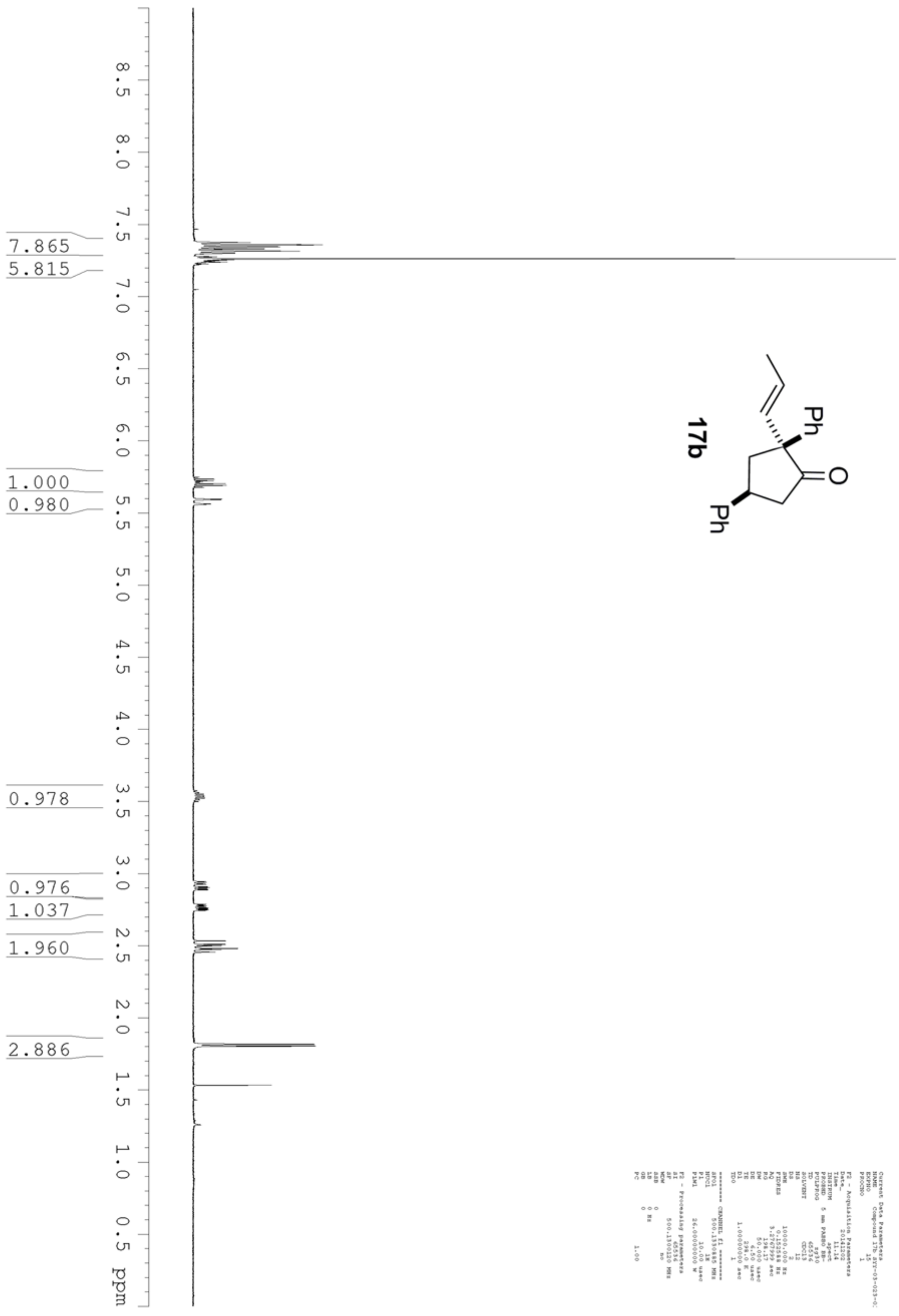

7.865
5.815
1.000
0.980
0.978
0.976
1.037
1.960
2.886
8.5
8.0
7.5
7.0
6.5
6.0
5.5
5.0
4.5
4.0
3.5
3.0
2.5
2.0
1.5
1.0
0.5 ppm
17b
Ph
O
Ph

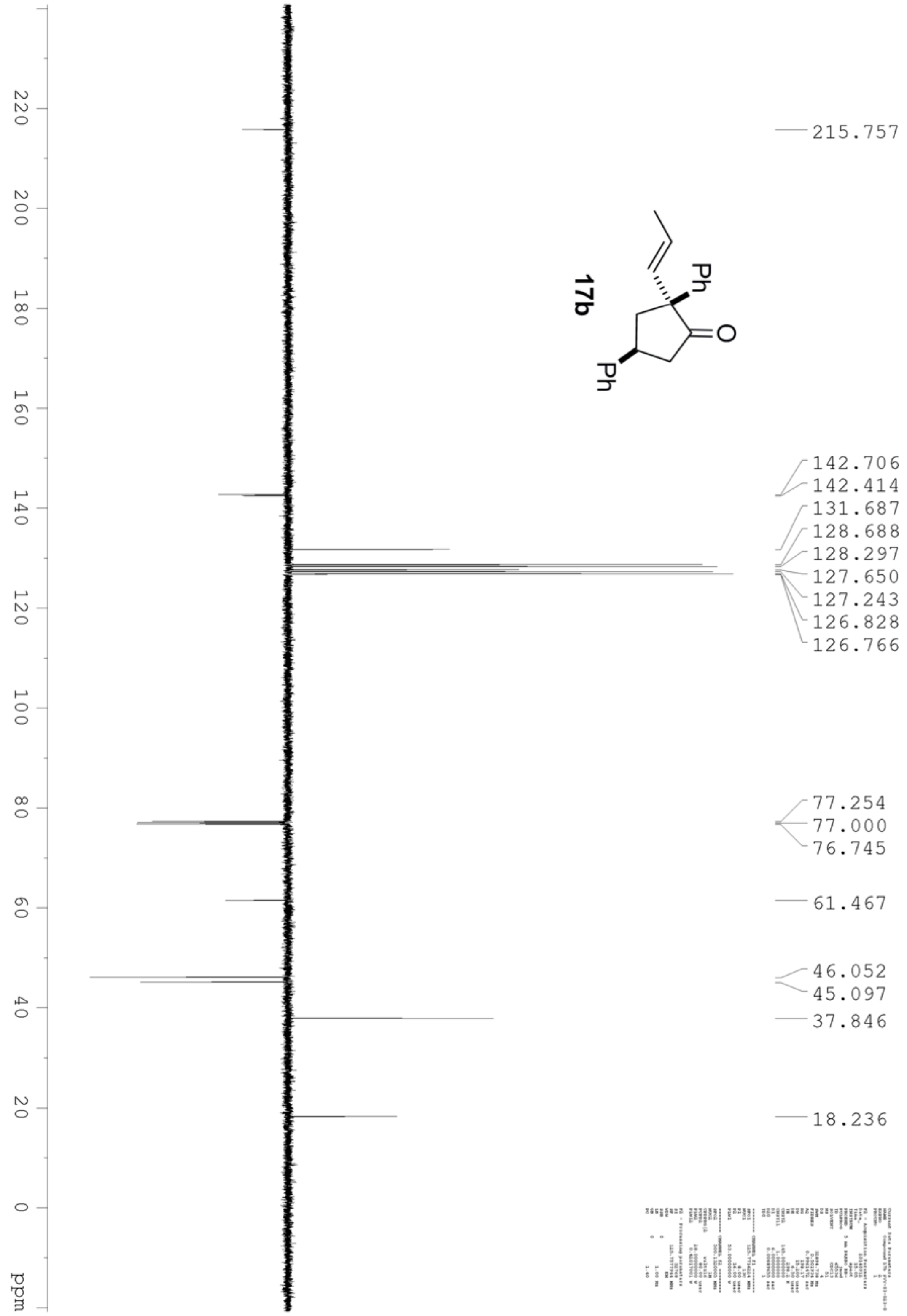
17b
Ph
O
Ph
215.757
142.706
142.414
131.687
128.688
128.297
127.650
127.243
126.828
126.766
77.254
77.000
76.745
61.467
46.052
45.097
37.846
18.236
ppm

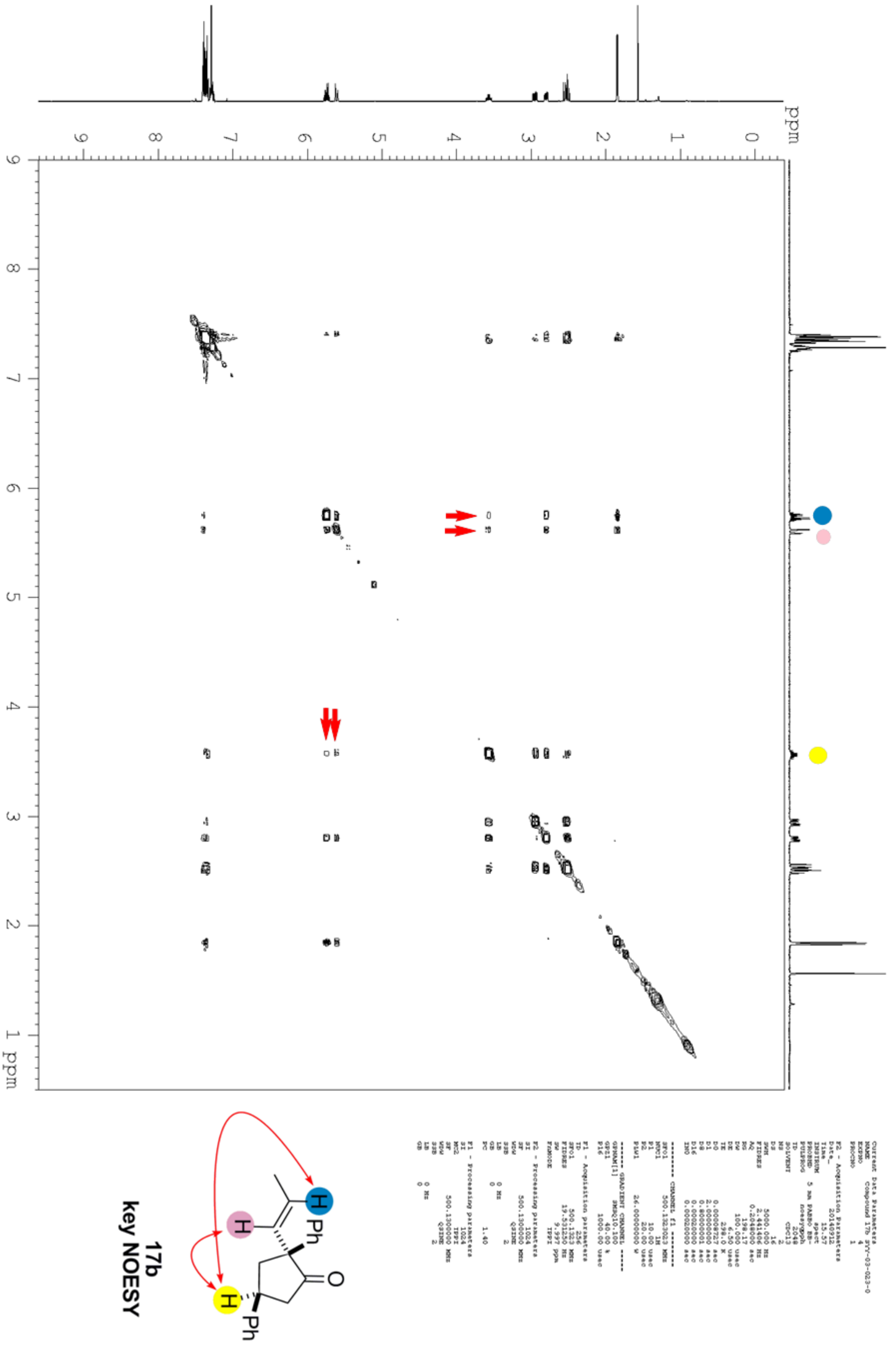
17b
key NOESY
Ph
Ph
O
H
H
H

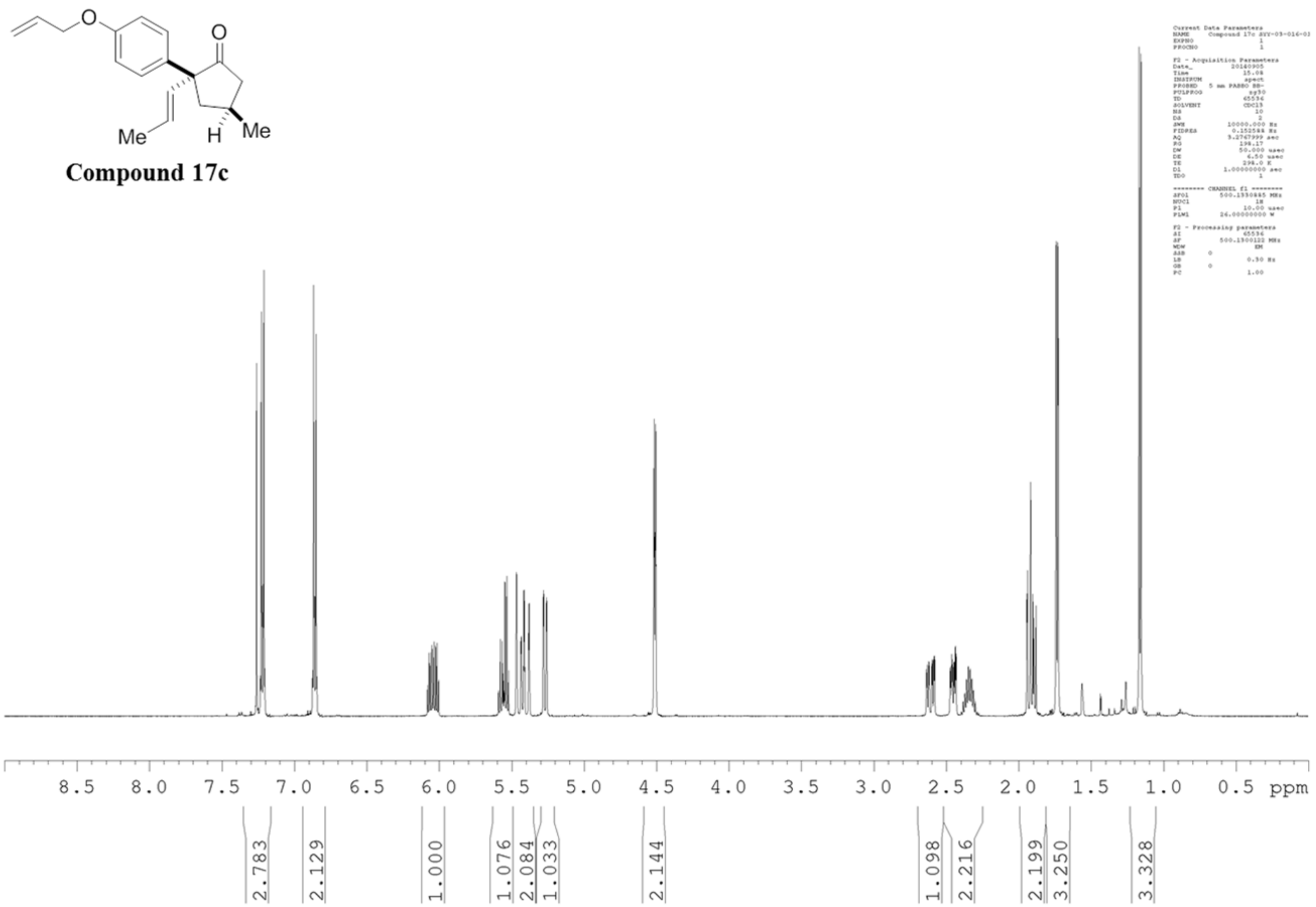

Compound 17c

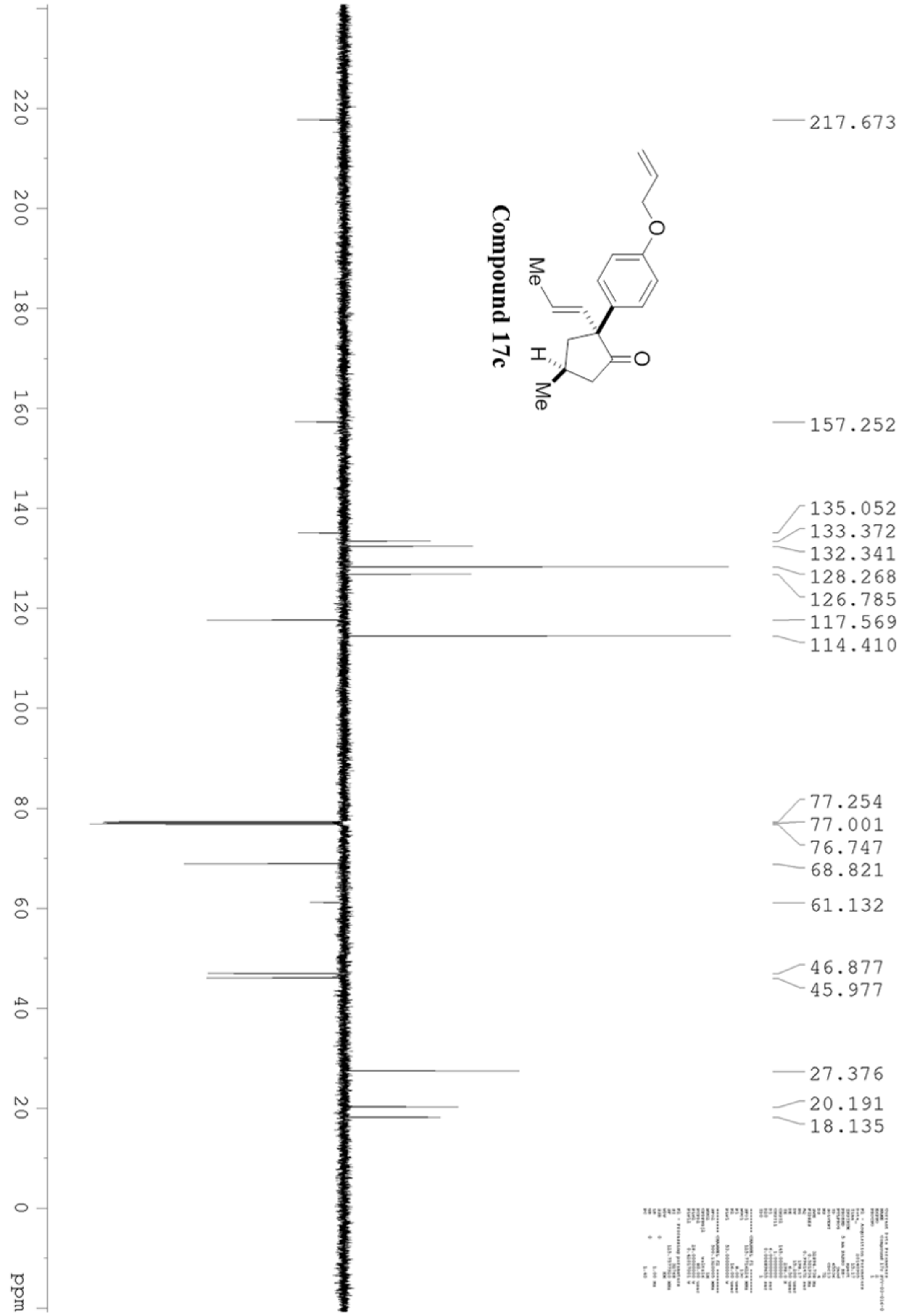
Compound 17c
217.673
157.252
135.052
133.372
132.341
128.268
126.785
117.569
114.410
77.254
77.001
76.747
68.821
61.132
46.877
45.977
27.376
20.191
18.135
ppm

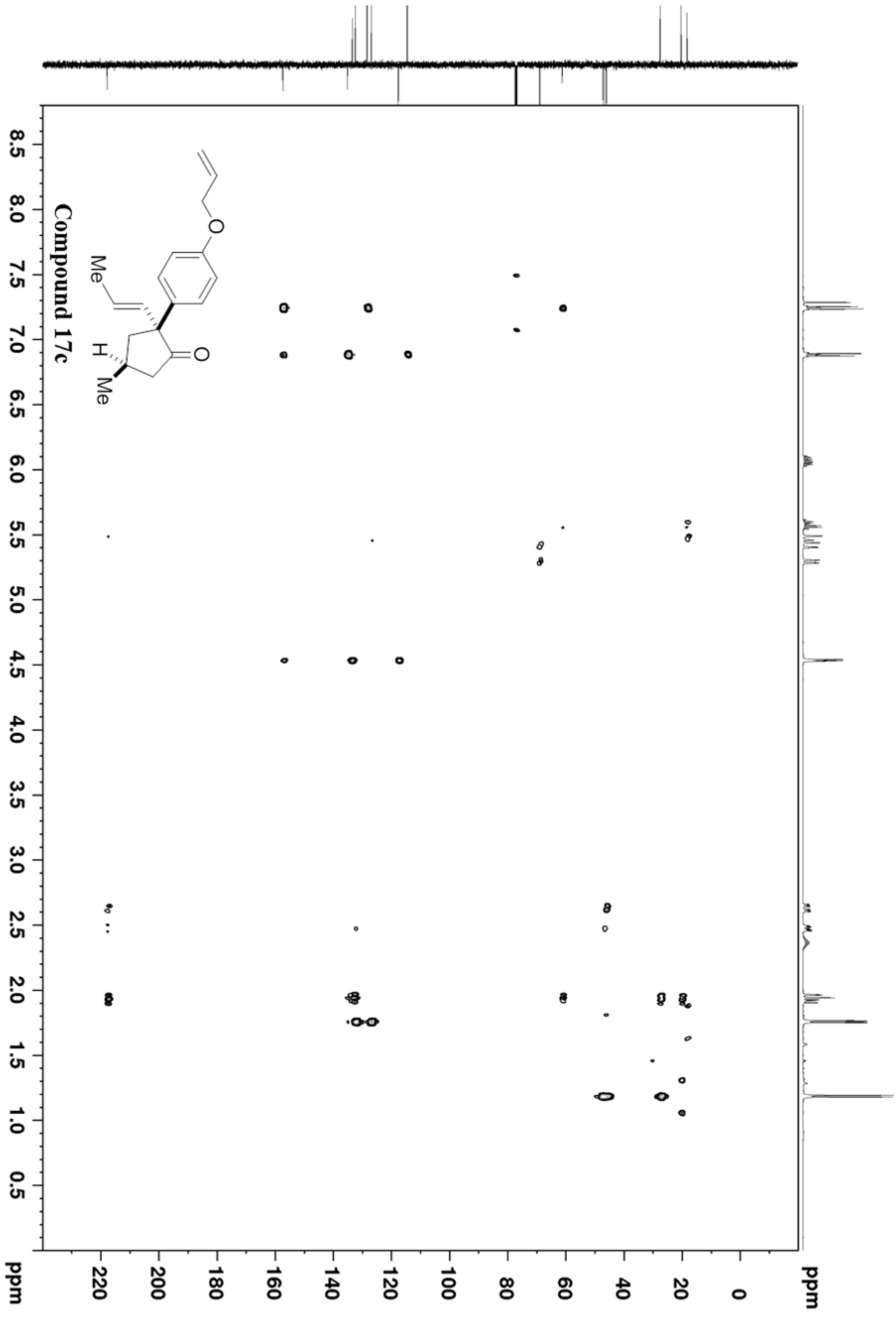
Compound 17c

HMBC

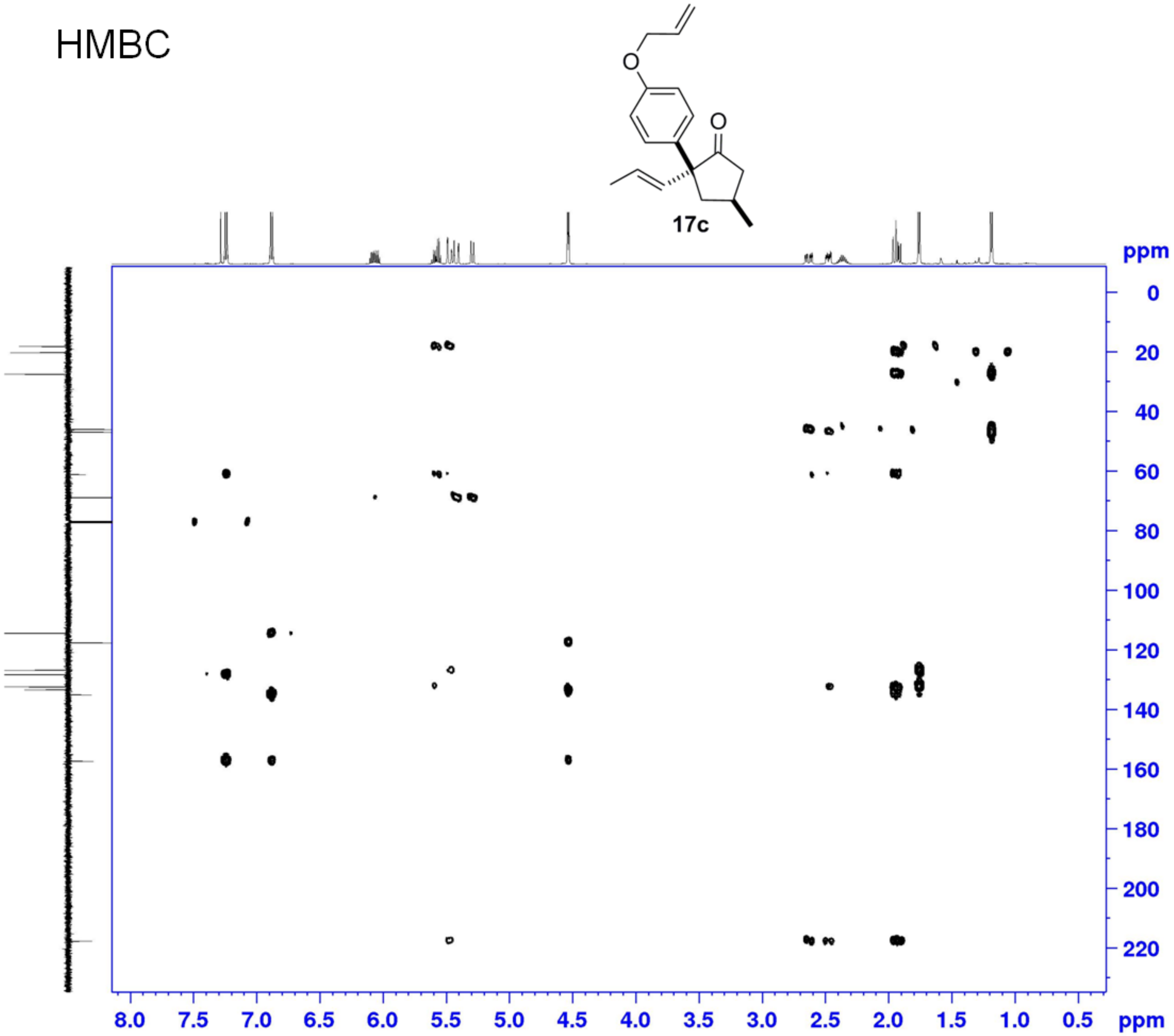

17c
ppm
0
20
40
60
80
100
120
140
160
180
200
220
8.0
7.5
7.0
6.5
6.0
5.5
5.0
4.5
4.0
3.5
3.0
2.5
2.0
1.5
1.0
0.5
ppm

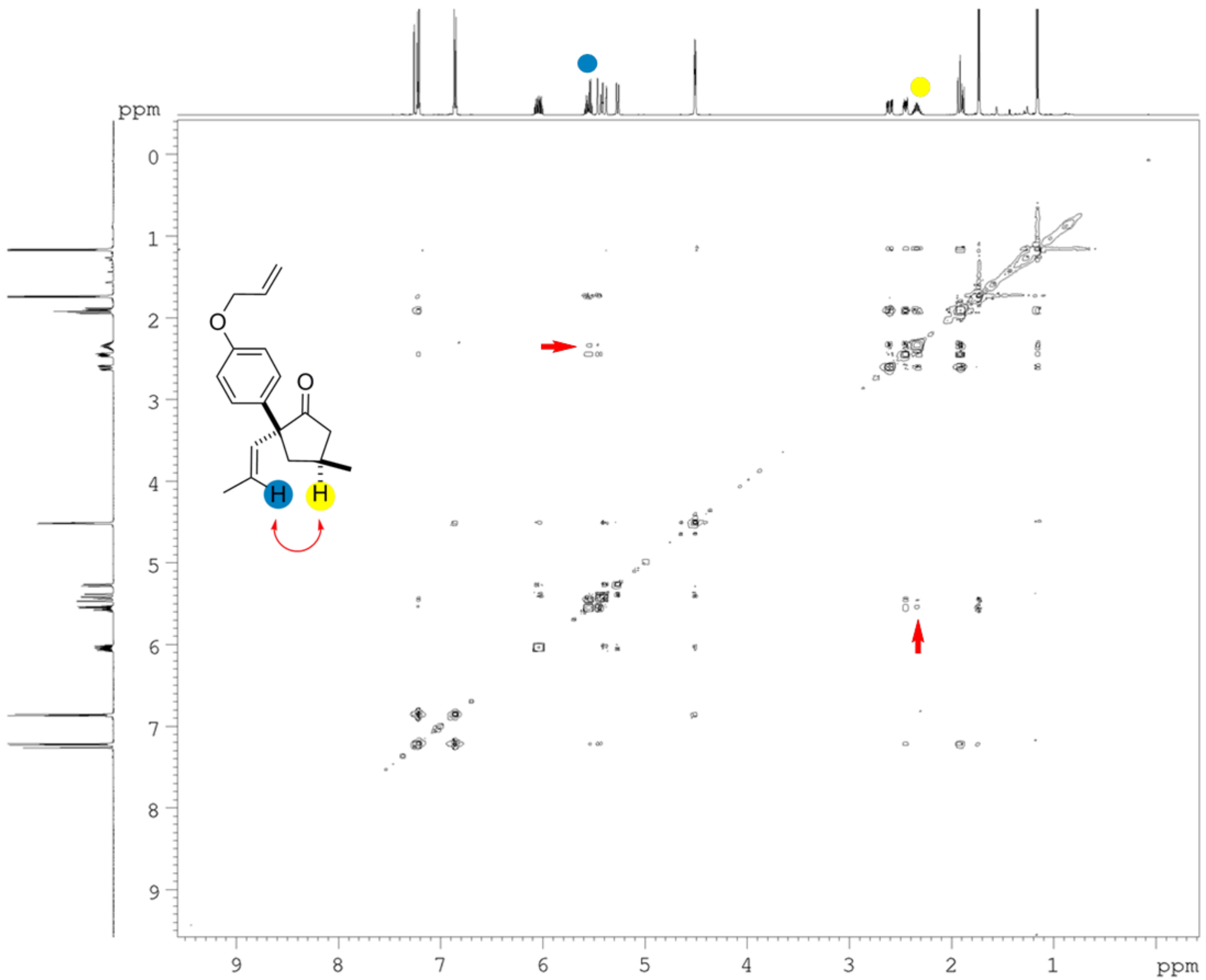

Current Data Parameters
NAME Compound 17c SYY-03-016-01
EXPNO 9
PROCNO 1

F2 - Acquisition Parameters
Date_ 20140908
Time 16.20
INSTRUM spect
PROBHD 5 mm PABBO BB-
PULPROG noesygpph
TD 2048
SOLVENT CDCl3
NS 4
DS 16
SWH 5000.000 Hz
FIDRES 2.441406 Hz
AQ 0.2048000 sec
RG 198.17
DW 100.000 usec
DE 6.50 usec
TE 298.0 K
D0 0.00008727 sec
D1 2.00000000 sec
D8 0.60000002 sec
D16 0.00020000 sec
IN0 0.00020000 sec

======== CHANNEL f1 ========
SFO1 500.1323023 MHz
NUC1 1H
P1 10.00 usec
P2 20.00 usec
PLW1 26.00000000 W

====== GRADIENT CHANNEL =====
GPNAM[1] SMSQ10.100
GPZ1 40.00 %
P16 1000.00 usec

F1 - Acquisition parameters
TD 256
SFO1 500.1323 MHz
FIDRES 19.531250 Hz
SW 9.997 ppm
FnMODE TPPI

F2 - Processing parameters
SI 1024
SF 500.1300130 MHz
WDW QSINE
SSB 2
LB 0 Hz
GB 0
PC 1.00

F1 - Processing parameters
SI 1024
MC2 TPPI
SF 500.1300119 MHz
WDW QSINE
SSB 2
LB 0 Hz
GB 0

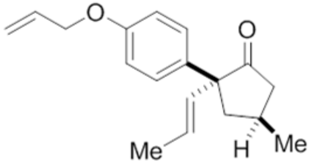

Compound 17c

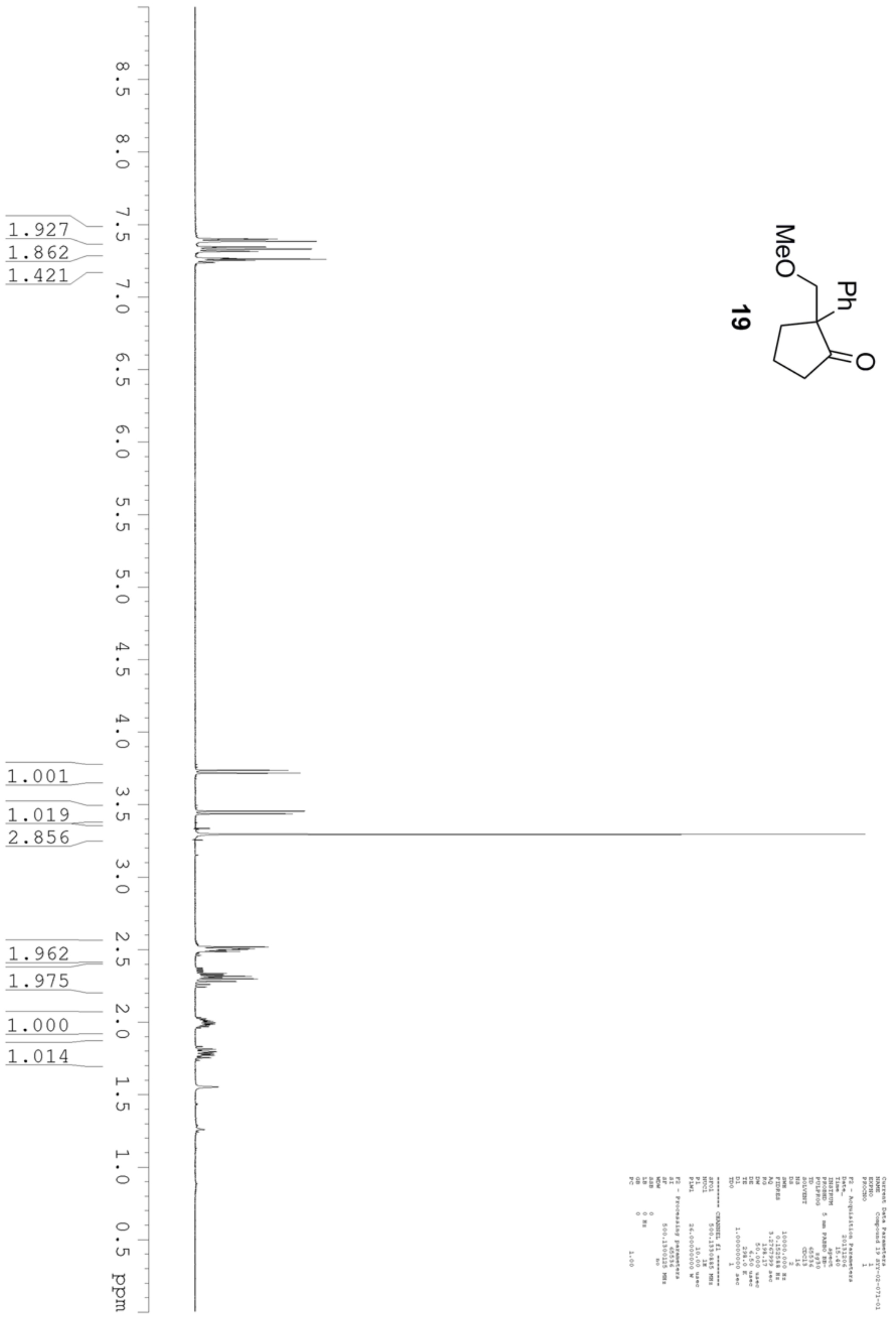
MeO
Ph
O
19
8.5
8.0
7.5
7.0
6.5
6.0
5.5
5.0
4.5
4.0
3.5
3.0
2.5
2.0
1.5
1.0
0.5 ppm
1.927
1.862
1.421
1.001
1.019
2.856
1.962
1.975
1.000
1.014

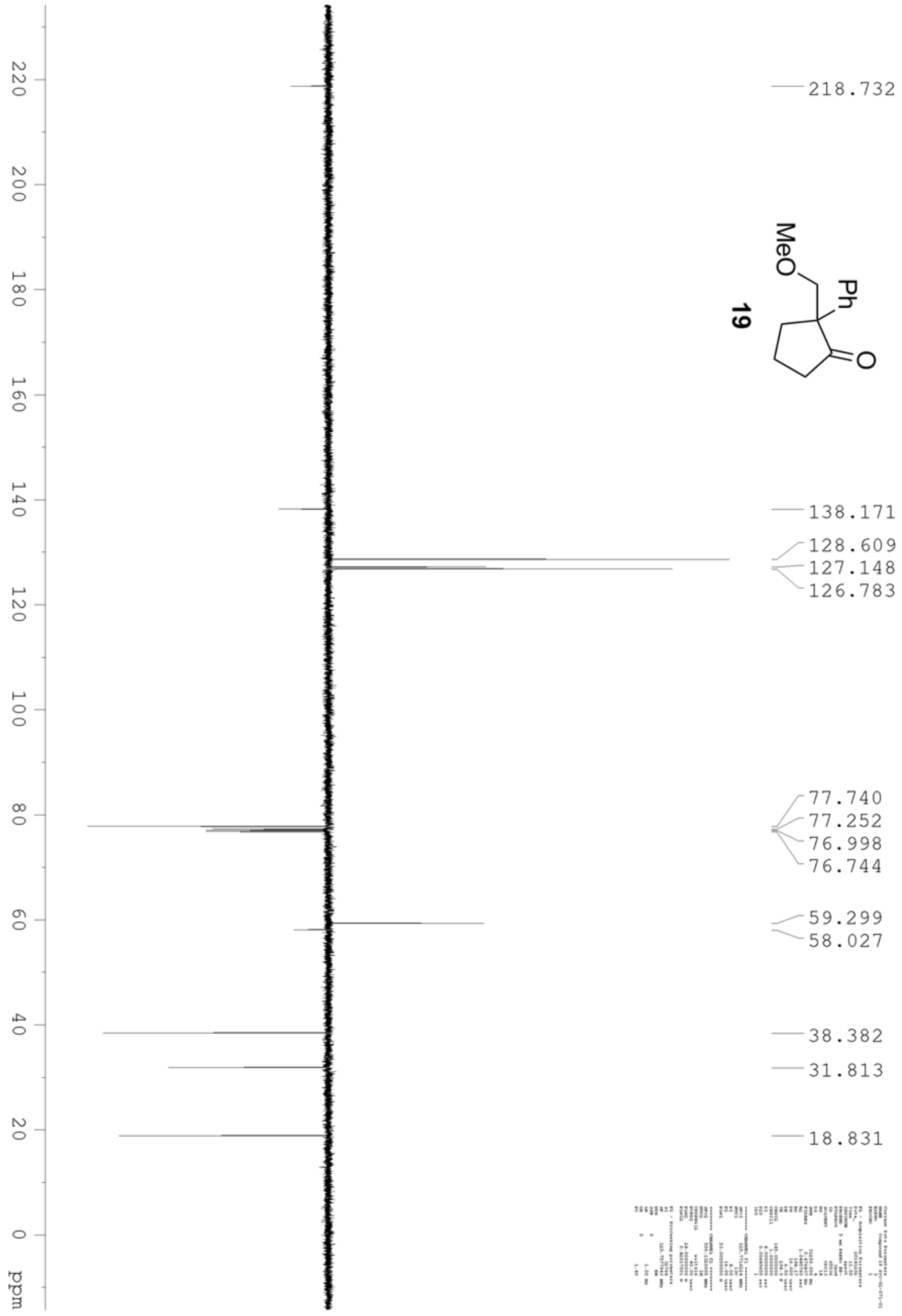

19
218.732
138.171
128.609
127.148
126.783
77.740
77.252
76.998
76.744
59.299
58.027
38.382
31.813
18.831
ppm

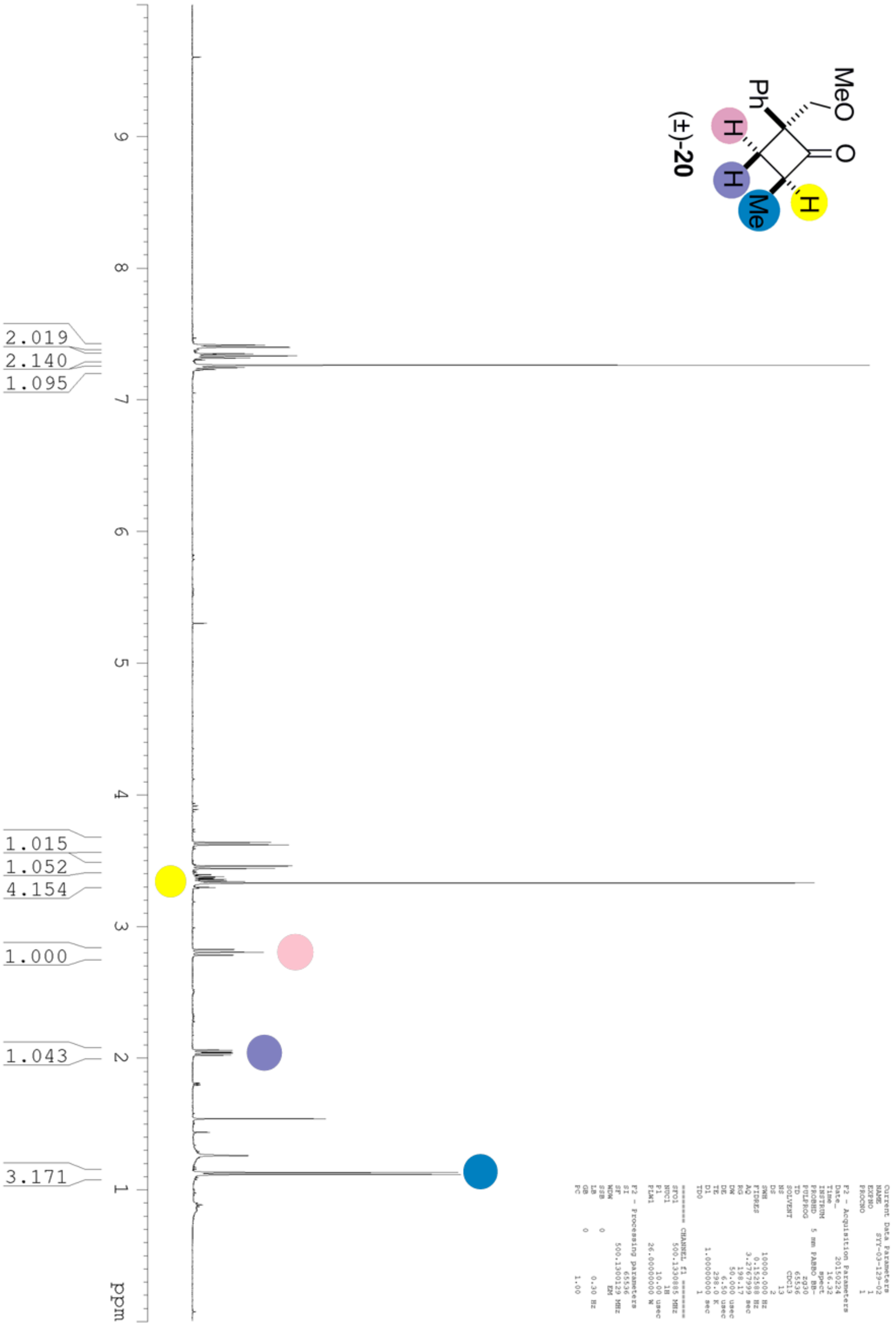
MeO
O
Ph
H
H
H
Me
(±)-20
2.019
2.140
1.095
1.015
1.052
4.154
1.000
1.043
3.171
ppm

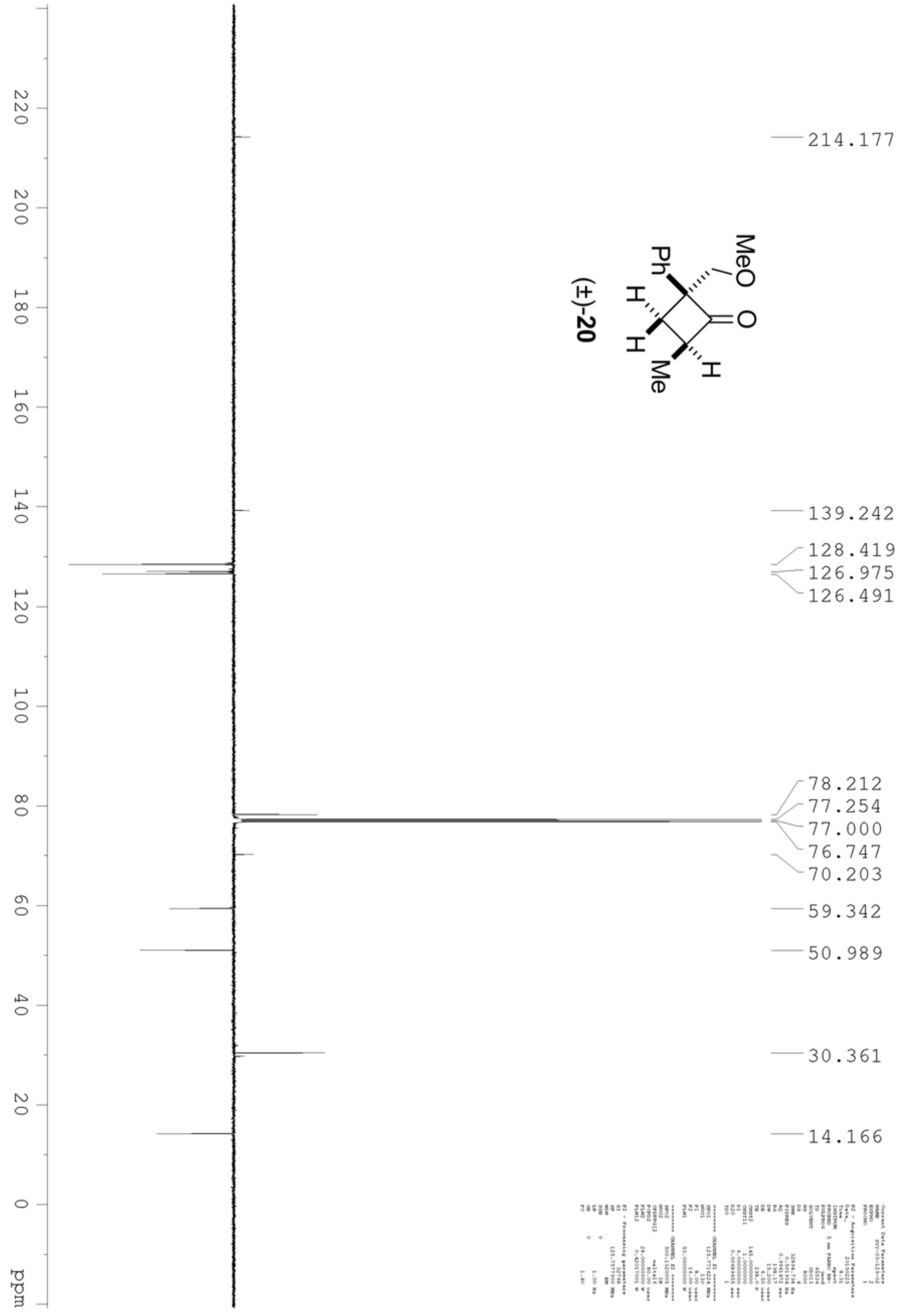

214.177
MeO
O
H
Ph
H
H
Me
(±)-20
139.242
128.419
126.975
126.491
78.212
77.254
77.000
76.747
70.203
59.342
50.989
30.361
14.166
220
200
180
160
140
120
100
80
60
40
20
0
ppm

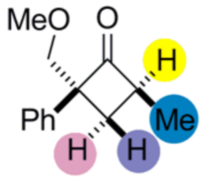

(±)-**20**

COSY

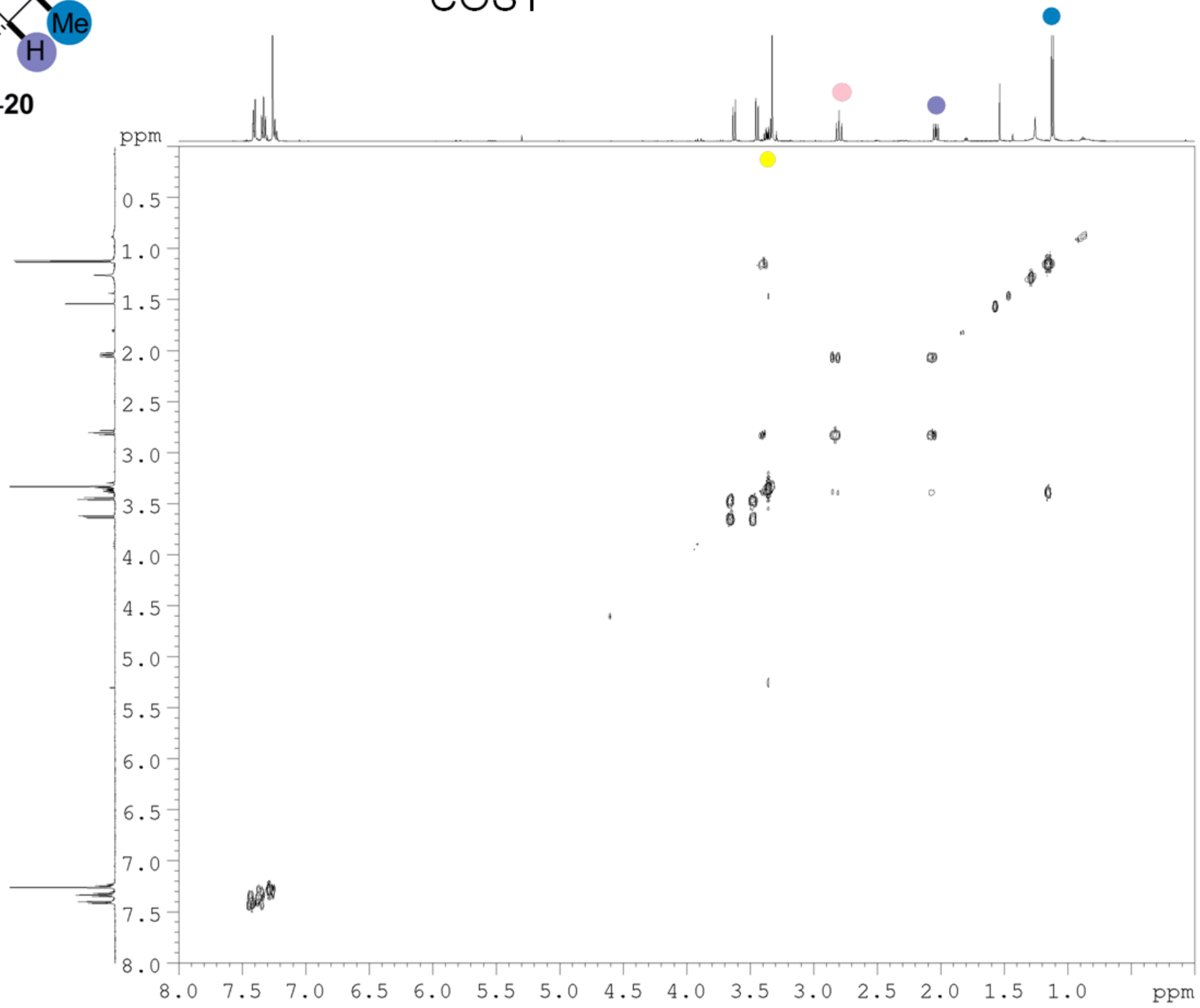


```
Current Data Parameters
NAME       SYY-03-129-02
EXPNO                  3
PROCNO                 1

F2 - Acquisition Parameters
Date_           20150225
Time               17.49
INSTRUM            spect
PROBHD   5 mm PABBO BB-
PULPROG         cosygpqf
TD                  2048
SOLVENT            CDCl3
NS                     1
DS                     8
SWH             5000.000 Hz
FIDRES          2.441406 Hz
AQ             0.2048000 sec
RG                198.17
DW               100.000 usec
DE                  6.50 usec
TE                 298.0 K
D0            0.00000300 sec
D1            1.50000000 sec
D13           0.00000400 sec
D16           0.00020000 sec
IN0           0.00020000 sec

======== CHANNEL f1 ========
SFO1         500.1323026 MHz
NUC1                  1H
P0                 10.00 usec
P1                 10.00 usec
PLW1         26.00000000 W

====== GRADIENT CHANNEL =====
GPNAM[1]      SMSQ10.100
GPZ1               10.00 %
P16              1000.00 usec

F1 - Acquisition parameters
TD                   128
SFO1            500.1323 MHz
FIDRES         39.062500 Hz
SW                 9.997 ppm
FnMODE                QF

F2 - Processing parameters
SI                  1024
SF           500.1300000 MHz
WDW                 SINE
SSB      0
LB       0 Hz
GB       0
PC                  1.00

F1 - Processing parameters
SI                  1024
MC2                   QF
SF           500.1300000 MHz
WDW                 SINE
SSB      0
LB       0 Hz
GB       0
```

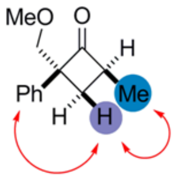
MeO
O
H
Ph
H
H
Me

(±)-**20**
key NOESY

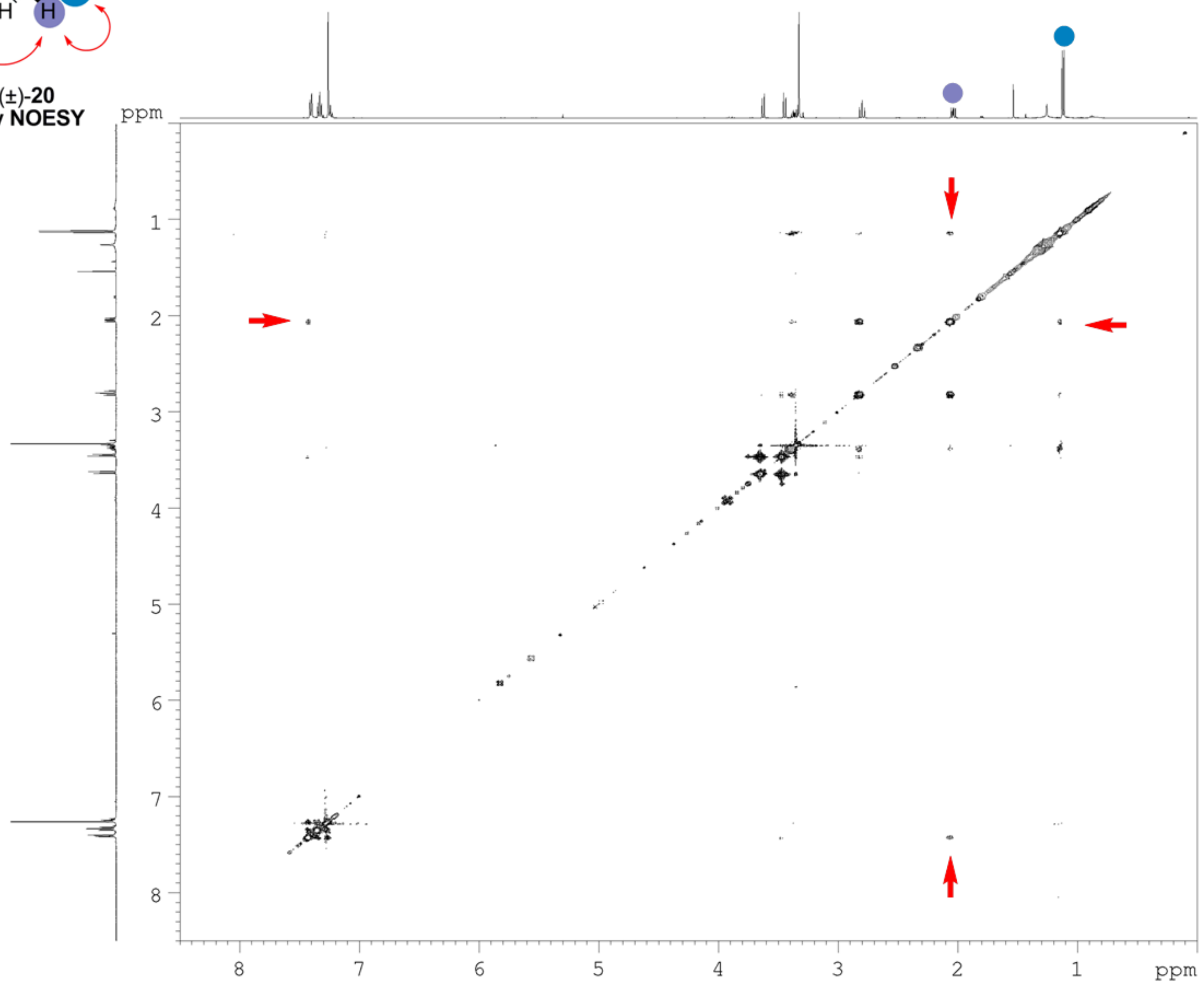
ppm
1
2
3
4
5
6
7
8
8
7
6
5
4
3
2
1
ppm

Current Data Parameters
NAME SYY-03-129-02
EXPNO 5
PROCNO 1

F2 - Acquisition Parameters
Date_ 20150225
Time 18.35
INSTRUM spect
PROBHD 5 mm PABBO BB-
PULPROG noesygpph
TD 2048
SOLVENT CDCl3
NS 4
DS 32
SWH 5000.000 Hz
FIDRES 2.441406 Hz
AQ 0.2048000 sec
RG 198.17
DW 100.000 usec
DE 6.50 usec
TE 298.0 K
D0 0.00008727 sec
D1 2.00000000 sec
D8 0.80000001 sec
D16 0.00020000 sec
IN0 0.00020000 sec

======== CHANNEL f1 ========
SFO1 500.1323023 MHz
NUC1 1H
P1 10.00 usec
P2 20.00 usec
PLW1 26.00000000 W

====== GRADIENT CHANNEL =====
GPNAM[1] SMSQ10.100
GPZ1 40.00 %
P16 1000.00 usec

F1 - Acquisition parameters
TD 4268
SFO1 500.1323 MHz
FIDRES 1.171509 Hz
SW 9.997 ppm
FnMODE TPPI

F2 - Processing parameters
SI 1024
SF 500.1300000 MHz
WDW QSINE
SSB 2
LB 0 Hz
GB 0
PC 1.00

F1 - Processing parameters
SI 1024
MC2 TPPI
SF 500.1300000 MHz
WDW QSINE
SSB 2
LB 0 Hz
GB 0

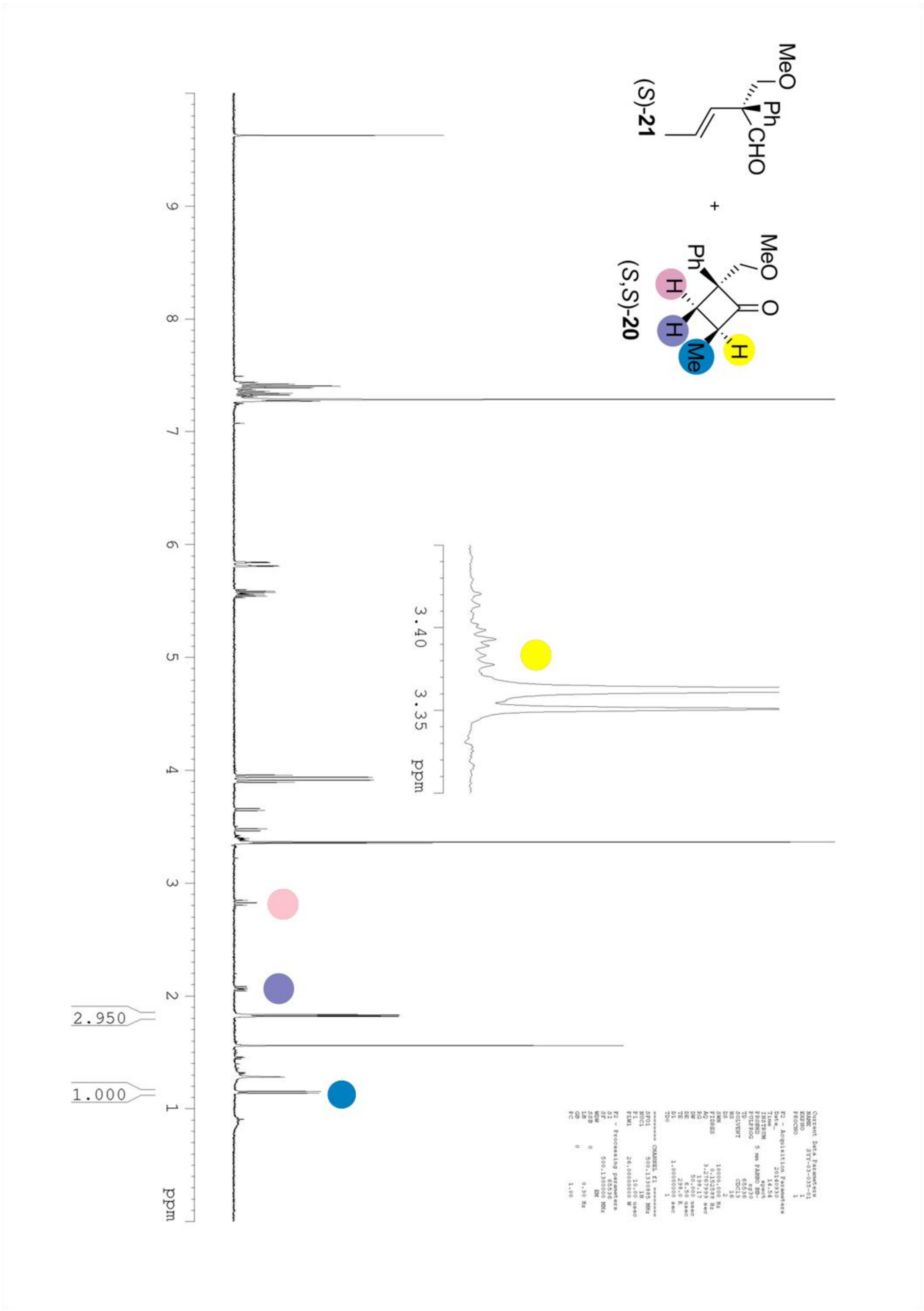
MeO
Ph
CHO
(S)-21
+
MeO
O
Ph
H
Me
(S,S)-20
3.40
3.35
ppm
2.950
1.000
ppm

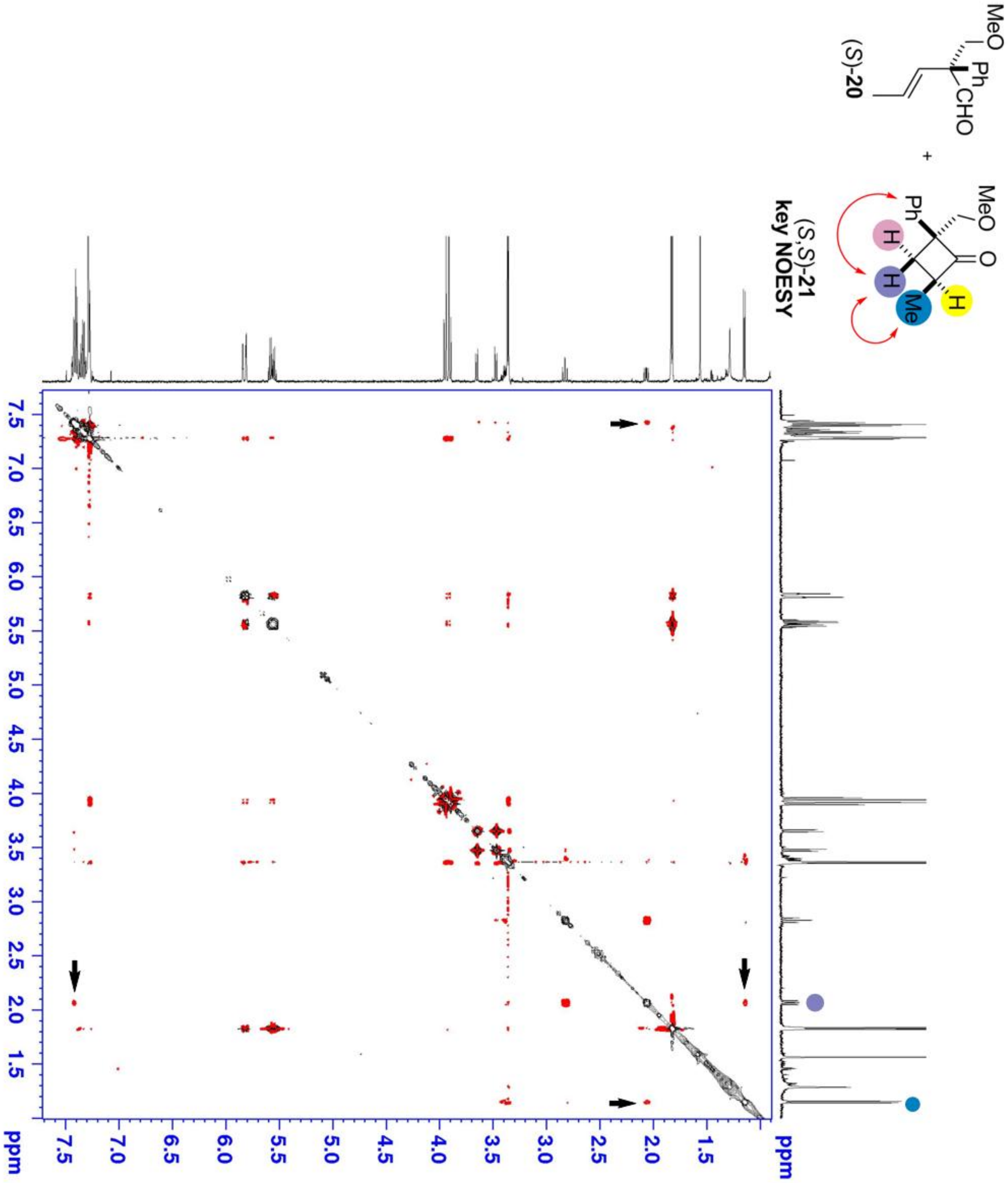

MeO
Ph
CHO
(S)-20
+
MeO
O
Ph
H
H
Me
H
(S,S)-21
key NOESY
ppm
ppm

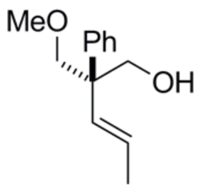
MeO
Ph
OH

(*R*)–**22**

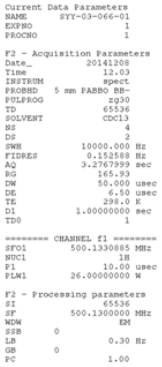
Current Data Parameters
NAME SYY-03-066-01
EXPNO 1
PROCNO 1
F2 - Acquisition Parameters
Date_ 20141208
Time 12.03
INSTRUM spect
PROBHD 5 mm PABBO BB-
PULPROG zg30
TD 65536
SOLVENT CDCl3
NS 4
DS 2
SWH 10000.000 Hz
FIDRES 0.152588 Hz
AQ 3.2767999 sec
RG 165.93
DW 50.000 usec
DE 6.50 usec
TE 298.0 K
D1 1.00000000 sec
TD0 1
======== CHANNEL f1 ========
SFO1 500.1330885 MHz
NUC1 1H
P1 10.00 usec
PLW1 26.00000000 W
F2 - Processing parameters
SI 65536
SF 500.1300000 MHz
WDW EM
SSB 0
LB 0.30 Hz
GB 0
PC 1.00

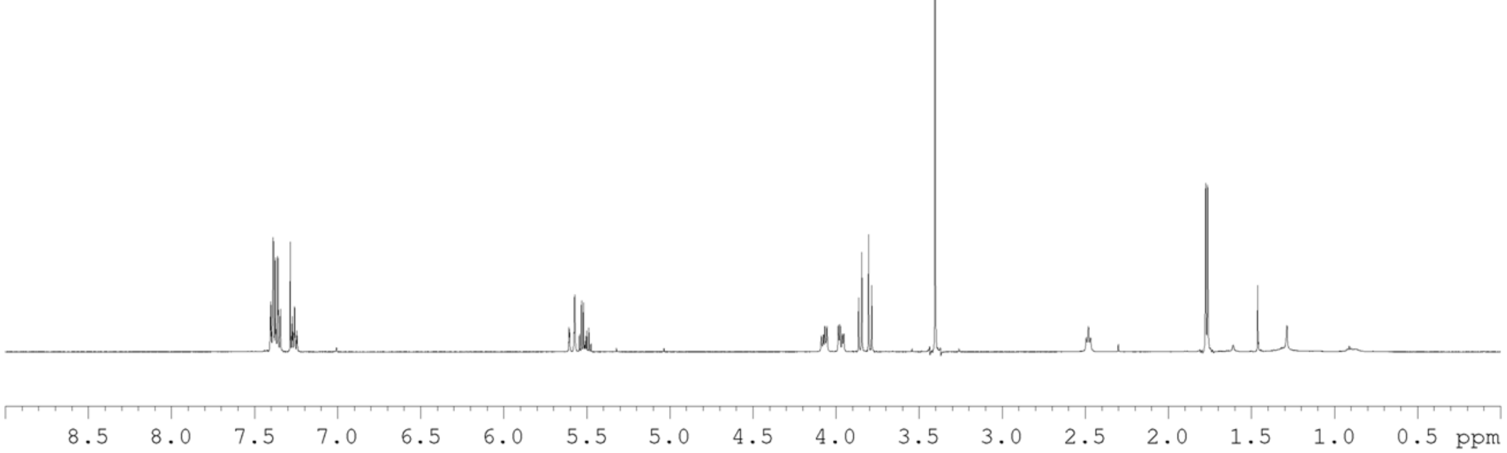
8.5
8.0
7.5
7.0
6.5
6.0
5.5
5.0
4.5
4.0
3.5
3.0
2.5
2.0
1.5
1.0
0.5
ppm

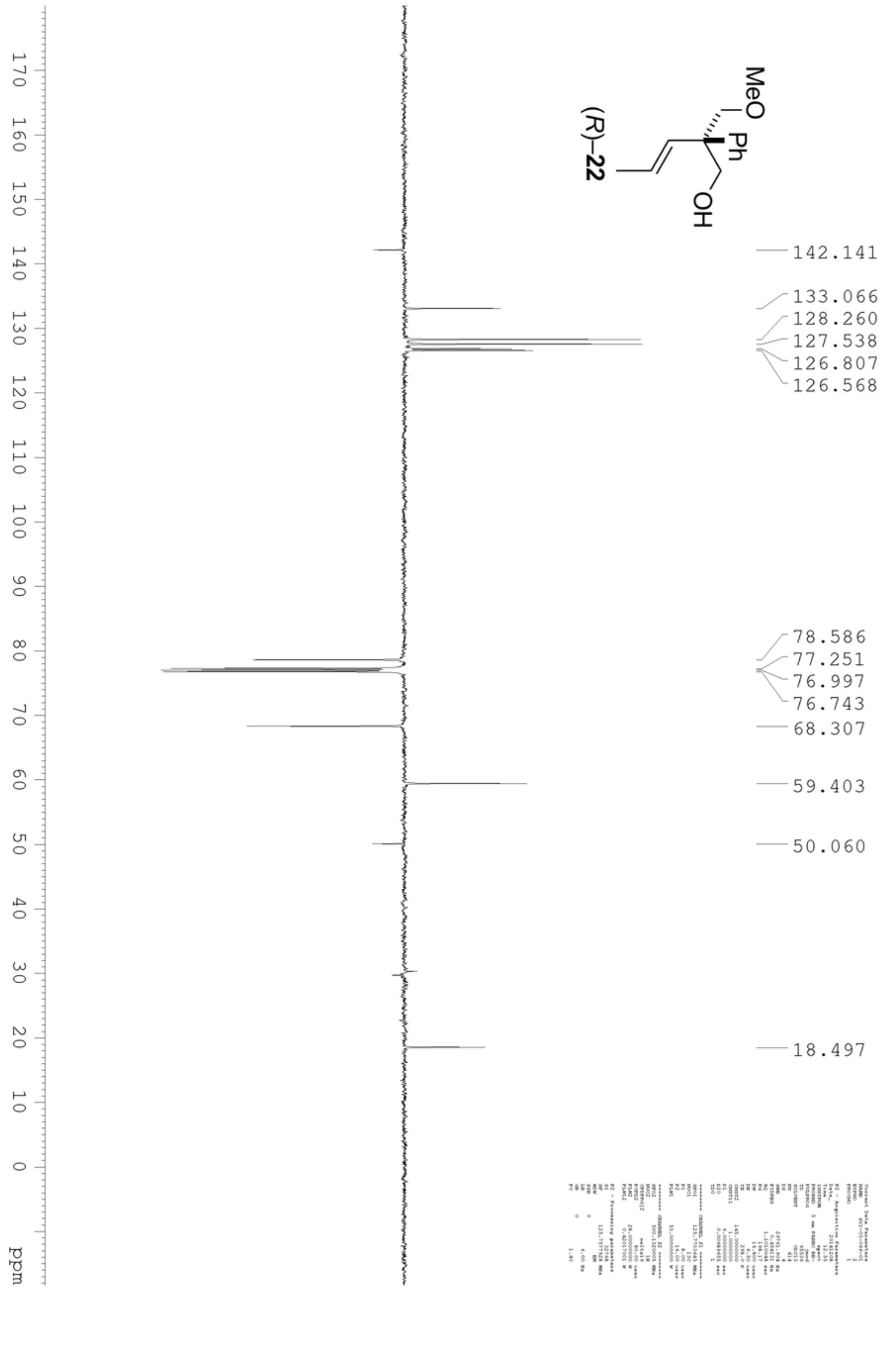

MeO
Ph
OH
(R)–22
142.141
133.066
128.260
127.538
126.807
126.568
78.586
77.251
76.997
76.743
68.307
59.403
50.060
18.497
170
160
150
140
130
120
110
100
90
80
70
60
50
40
30
20
10
0
ppm

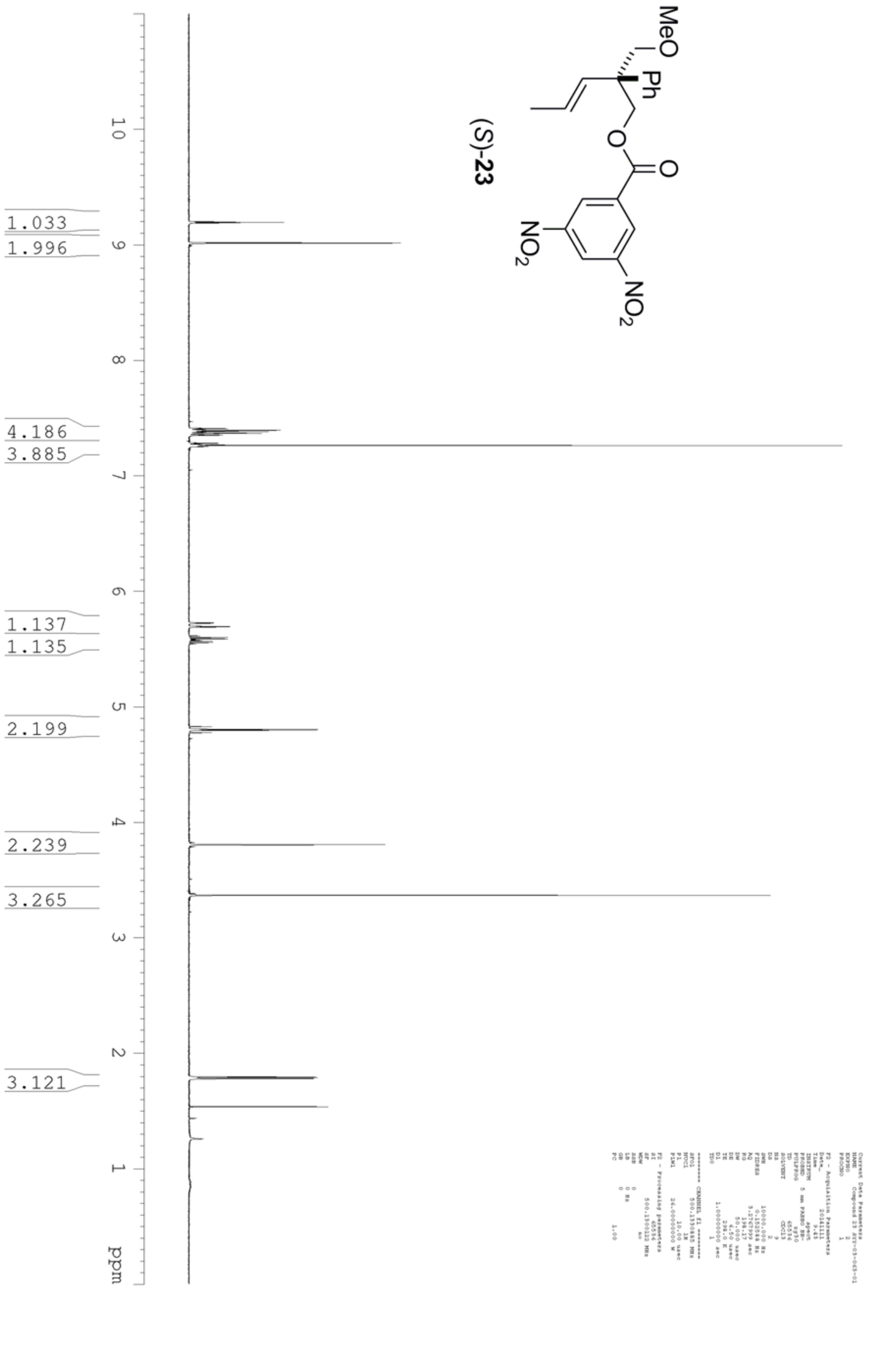
MeO
Ph
NO2
NO2
(S)-23
1.033
1.996
4.186
3.885
1.137
1.135
2.199
2.239
3.265
3.121
10
9
8
7
6
5
4
3
2
1
ppm

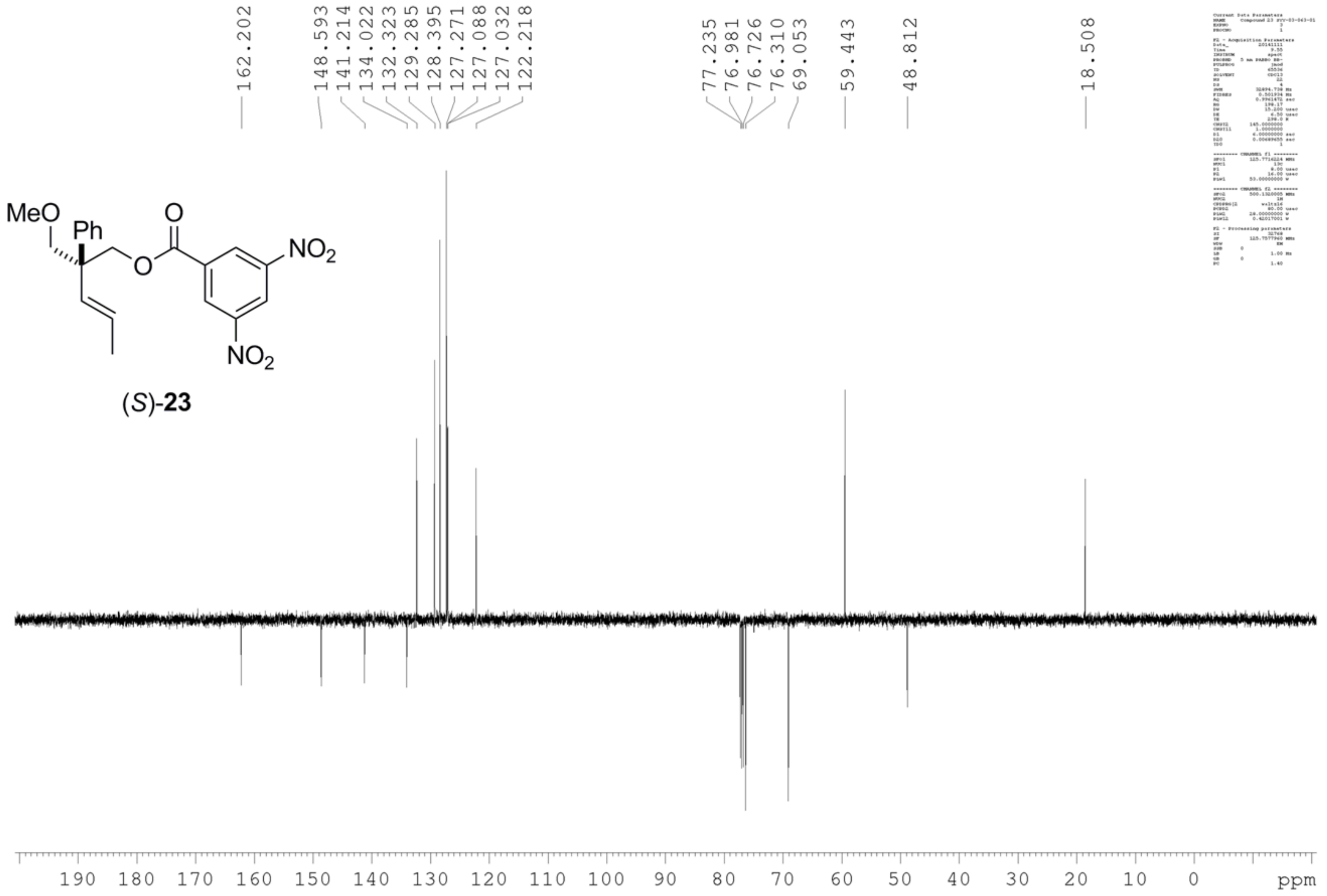
MeO
Ph
O
NO_2
NO_2
162.202
148.593
141.214
134.022
132.323
129.285
128.395
127.271
127.088
127.032
122.218
77.235
76.981
76.726
76.310
69.053
59.443
48.812
18.508
190
180
170
160
150
140
130
120
110
100
90
80
70
60
50
40
30
20
10
0
ppm
(*S*)-**23**

Data File C:\CHEM32\2\DATA\SYIP\SYY-02-070-01.D
Sample Name: SYY-02-070-01

===

Acq. Operator : Stephanie Yip
Acq. Instrument : Instrument 2 Location : Vial 62
Injection Date : 1/24/2014 3:04:09 PM
Inj Volume : 10 µl
Acq. Method : C:\CHEM32\2\METHODS\SYY CYCLISATION METHOD.M
Last changed : 1/24/2014 3:23:00 PM by Stephanie Yip
(modified after loading)
Analysis Method : C:\CHEM32\2\METHODS\CR AJC ON.M
Last changed : 1/24/2014 2:29:55 PM by Chris Riley
(modified after loading)
Sample Info : AD-H 99:1 hex:IPA 1ml/min
cyclopentanone racemic

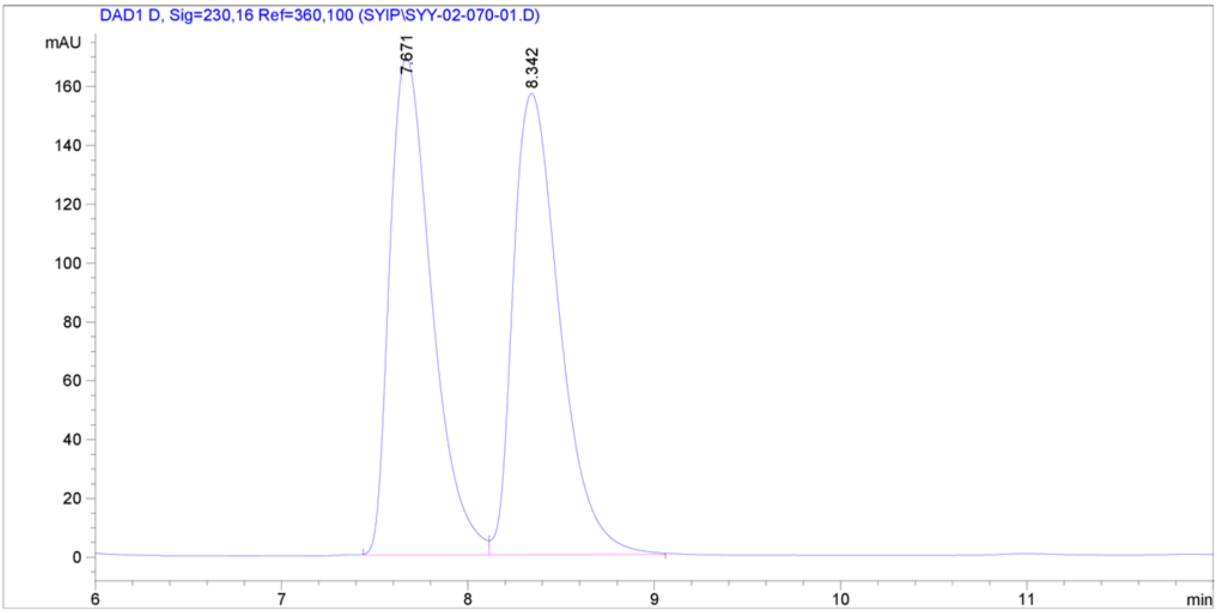

===
Area Percent Report
===

Sorted By : Signal
Multiplier : 1.0000
Dilution : 1.0000
Use Multiplier & Dilution Factor with ISTDs

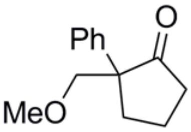

19

Signal 1: DAD1 D, Sig=230,16 Ref=360,100

Peak #	RetTime [min]	Type	Width [min]	Area [mAU*s]	Height [mAU]	Area %
1	7.671	BV	0.2449	2649.61450	169.00085	49.4277
2	8.342	VB	0.2697	2710.96729	156.82320	50.5723

Data File C:\CHEM32\2\DATA\SYIP\SYY-03-035-01 F2.D
Sample Name: SYY-03-035-01 F2

===

Acq. Operator : Stephanie Yip
Acq. Instrument : Instrument 2 Location : Vial 71
Injection Date : 1/24/2014 3:12:05 PM
Inj Volume : 10 µl
Acq. Method : C:\CHEM32\2\METHODS\SYY CYCLISATION METHOD.M
Last changed : 1/24/2014 3:12:10 PM by Stephanie Yip
(modified after loading)
Analysis Method : C:\CHEM32\2\METHODS\SMK AJC OXIDATION PAO.M
Last changed : 1/24/2014 3:23:44 PM by Stephanie Yip
(modified after loading)
Sample Info : 99% hexane, 1% ipa, 1ml/min, AD-H column
rxn at RT, F2.

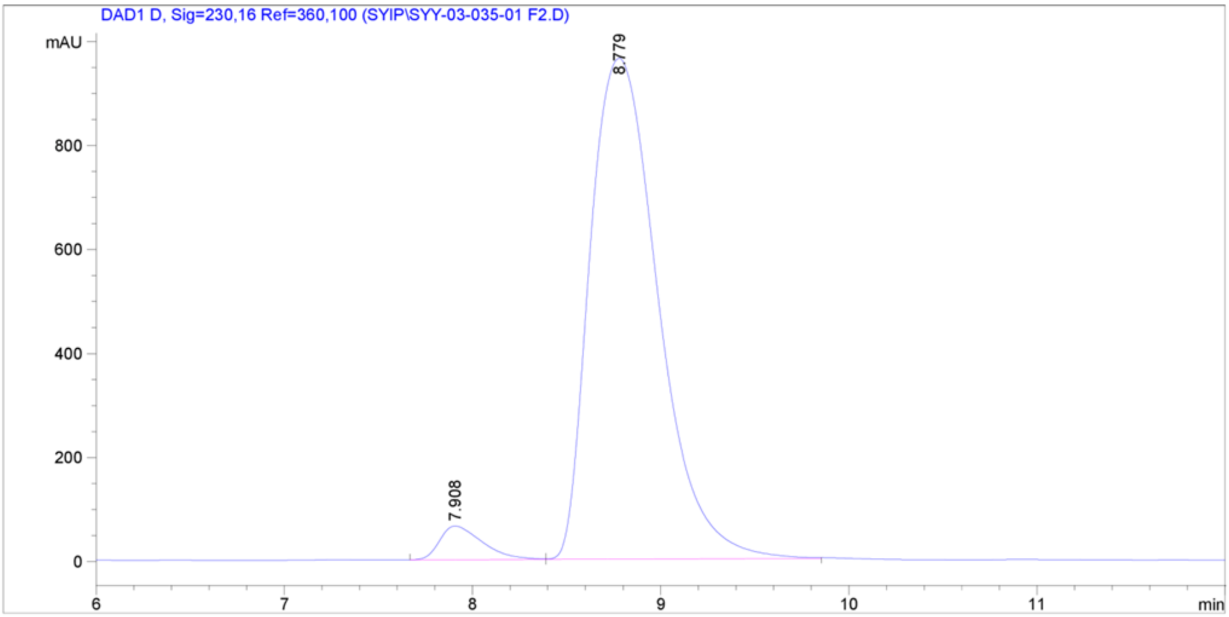

===
Area Percent Report
===

Sorted By : Signal
Multiplier : 1.0000
Dilution : 1.0000
Use Multiplier & Dilution Factor with ISTDs

Signal 1: DAD1 D, Sig=230,16 Ref=360,100

Peak #	RetTime [min]	Type	Width [min]	Area [mAU*s]	Height [mAU]	Area %
1	7.908	BV	0.2427	1015.39496	64.84653	4.0284
2	8.779	VB	0.3990	2.41904e4	963.25464	95.9716

Totals : 2.52058e4 1028.10117

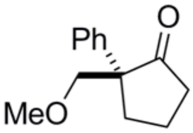

(*R*)-**19**

Data File C:\CHEM32\2\DATA\SYIP\SYY-03-062-01.D
Sample Name: SYY-03-062-01

===

Acq. Operator : Stephanie Yip
Acq. Instrument : Instrument 2 Location : Vial 12
Injection Date : 1/24/2014 2:57:00 PM
Inj Volume : 20 µl
Acq. Method : C:\CHEM32\2\METHODS\SMK AJC OXIDATION PAO.M
Last changed : 1/24/2014 3:15:19 PM by Stephanie Yip
(modified after loading)
Analysis Method : C:\CHEM32\2\METHODS\SMK AJC OXIDATION PAO.M
Last changed : 1/24/2014 2:50:09 PM by Stephanie Yip
(modified after loading)
Sample Info : 98:2% Hex:IPA, 1mL/min, AD-H.
Racemic alcohol

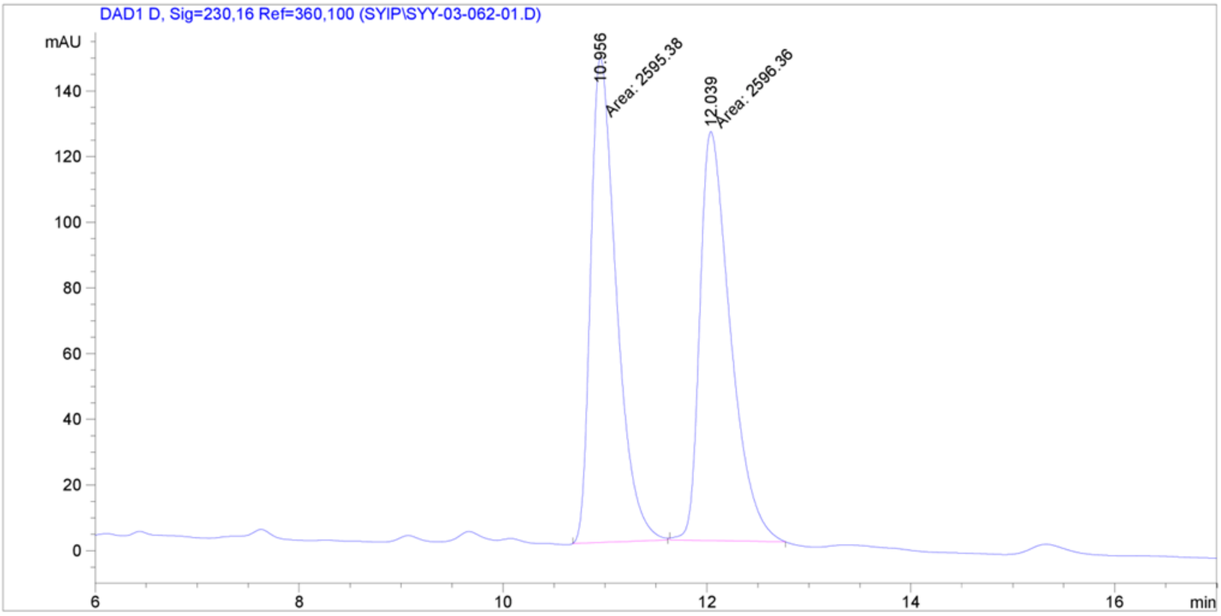

===
Area Percent Report
===

Sorted By : Signal
Multiplier : 1.0000
Dilution : 1.0000
Use Multiplier & Dilution Factor with ISTDs

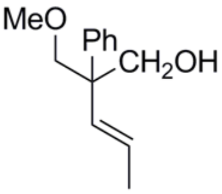

(±)-**22**

Signal 1: DAD1 D, Sig=230,16 Ref=360,100

Peak #	RetTime [min]	Type	Width [min]	Area [mAU*s]	Height [mAU]	Area %
1	10.956	MM	0.2927	2595.37598	147.77237	49.9905
2	12.039	MM	0.3472	2596.36255	124.61640	50.0095

Totals : 5191.73853 272.38877

Data File C:\CHEM32\2\DATA\SYIP\SYY-03-058-01.D
Sample Name: SYY-03-058-01

===

Acq. Operator : Stephanie Yip
Acq. Instrument : Instrument 2 Location : Vial 11
Injection Date : 1/24/2014 2:28:41 PM
Inj Volume : 20 µl
Acq. Method : C:\CHEM32\2\METHODS\SMK AJC OXIDATION PAO.M
Last changed : 1/24/2014 3:15:19 PM by Stephanie Yip
(modified after loading)
Analysis Method : C:\CHEM32\2\METHODS\CR AJC ON.M
Last changed : 1/24/2014 3:16:11 PM by Chris Riley
(modified after loading)
Sample Info : 98:2% Hex:IPA, 1mL/min, AD-H.
enantiopure alcohol

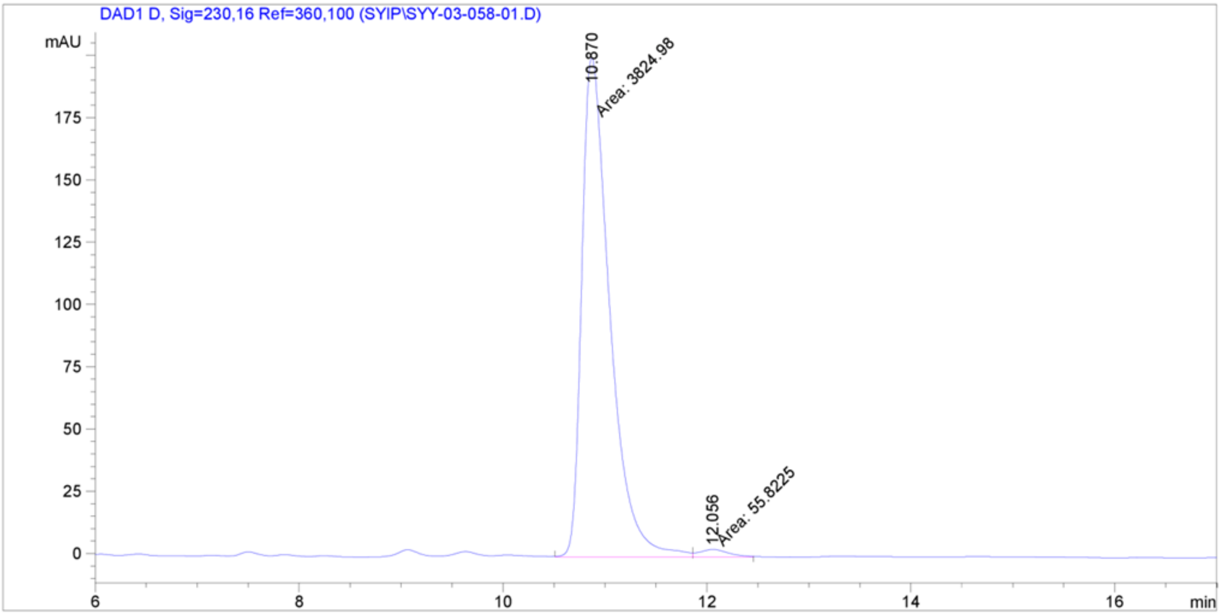

===

Area Percent Report

===

Sorted By : Signal
Multiplier : 1.0000
Dilution : 1.0000
Use Multiplier & Dilution Factor with ISTDs

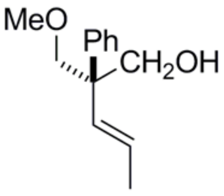

(*R*)-**22**

Signal 1: DAD1 D, Sig=230,16 Ref=360,100

Peak #	RetTime [min]	Type	Width [min]	Area [mAU*s]	Height [mAU]	Area %
1	10.870	MF	0.3180	3824.98169	200.44376	98.5616
2	12.056	FM	0.3191	55.82255	2.91560	1.4384

Totals : 3880.80424 203.35935

Data File C:\CHEM32\2\DATA\SYIP\SYY-03-150-01 OJ-H2.D
Sample Name: SYY-03-150-01 OJ-H2

===
Acq. Operator : Steph Yip
Acq. Instrument : Instrument 2 Location : Vial 1
Injection Date : 1/23/2015 2:04:08 PM
Inj Volume : 2 µl
Acq. Method : C:\CHEM32\2\METHODS\CR AJC ON.M
Last changed : 1/23/2015 2:34:56 PM by Steph Yip
(modified after loading)
Analysis Method : C:\CHEM32\2\METHODS\CR AJC ON.M
Last changed : 1/23/2015 2:22:30 PM by Steph Yip
(modified after loading)
Sample Info : OJ-H 94:6 HEX:IPA, 1mL/min.
chiral alcohol

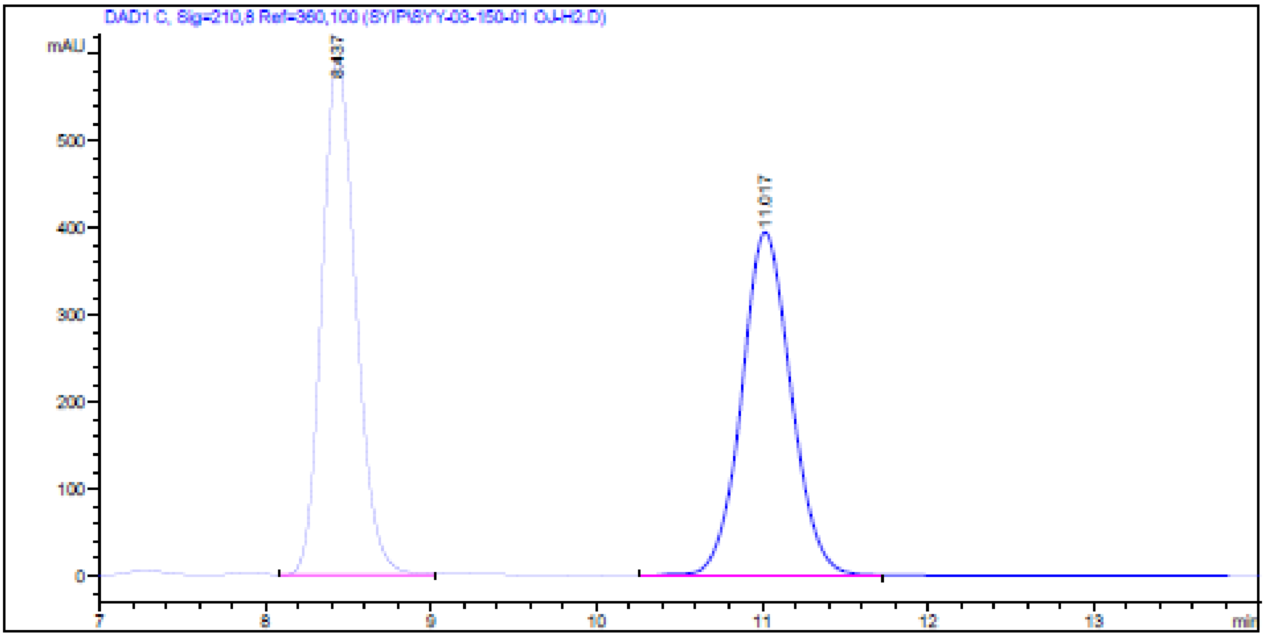

===
Area Percent Report
===

Sorted By : Signal
Multiplier : 1.0000
Dilution : 1.0000
Use Multiplier & Dilution Factor with ISTDs

Signal 1: DAD1 C, Sig=210,8 Ref=360,100

Peak #	RetTime [min]	Type	Width [min]	Area [mAU*s]	Height [mAU]	Area %
1	8.437	VB	0.2230	8474.80469	592.20422	50.8495
2	11.017	BB	0.3180	8191.63086	394.87689	49.1505

Instrument 2 1/23/2015 2:22:50 PM Steph Yip Page 1 of 2

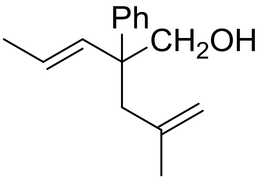

SI-3

Data File C:\CHEM32\2\DATA\SYIP\SYY-03-150-01 OJ-H2.D
Sample Name: SYY-03-150-01 OJ-H2

Totals : 1.66664e4 987.08112

===

*** End of Report ***

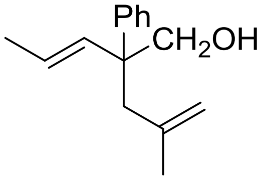

SI-3

Data File C:\CHEM32\2\DATA\SYIP\SYY-03-151-01 OJ-H.D
Sample Name: SYY-03-151-01 OJ-H

===
Acq. Operator   : Steph Yip
Acq. Instrument : Instrument 2   Location : Vial 2
Injection Date : 1/23/2015 2:16:23 PM
                                              Inj Volume : 2 µl
Acq. Method   : C:\CHEM32\2\METHODS\CR AJC ON.M
Last changed   : 1/23/2015 2:31:26 PM by Steph Yip
                  (modified after loading)
Analysis Method : C:\CHEM32\2\METHODS\CR AJC ON.M
Last changed   : 1/23/2015 2:22:30 PM by Steph Yip
                  (modified after loading)
Sample Info   : OJ-H 94:6 HEX:IPA, 1mL/min.
                  chiral alcohol

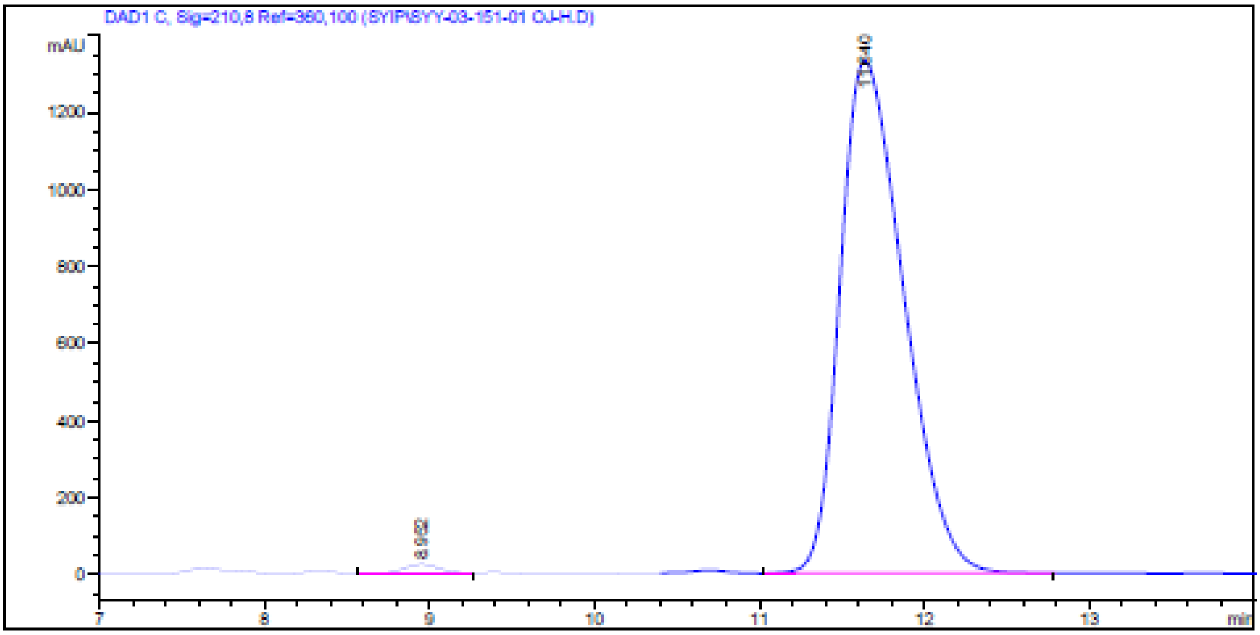

===
Area Percent Report
===

Sorted By   :   Signal
Multiplier   :   1.0000
Dilution   :   1.0000
Use Multiplier & Dilution Factor with ISTDs

Signal 1: DAD1 C, Sig=210,8 Ref=360,100

Peak #	RetTime [min]	Type	Width [min]	Area [mAU*s]	Height [mAU]	Area %
1	8.952	VV	0.2395	372.46994	23.93700	1.0323
2	11.640	VB	0.4187	3.57087e4	1332.38318	98.9677

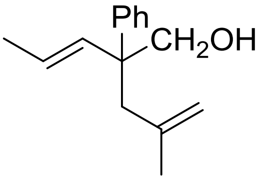

SI-3

Data File C:\CHEM32\2\DATA\SYIP\SYY-03-151-01 OJ-H.D
Sample Name: SYY-03-151-01 OJ-H

Totals : 3.60812e4 1356.32018

===
*** End of Report ***

Instrument 2 1/23/2015 2:24:14 PM Steph Yip Page 2 of 2

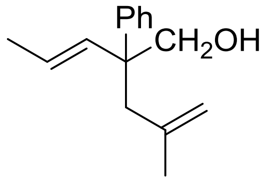

SI-3

Data File C:\CHEM32\2\DATA\SYIP\SYY-01-270-01 OJ-H.D
Sample Name: SYY-01-270-01 OJ-H

===
Acq. Operator : Steph Yip
Acq. Instrument : Instrument 2 Location : Vial 1
Injection Date : 1/23/2015 2:25:50 PM
Inj Volume : 5 µl
Acq. Method : C:\CHEM32\2\METHODS\SYY CYCLISATION METHOD.M
Last changed : 1/23/2015 2:50:03 PM by Steph Yip
(modified after loading)
Analysis Method : C:\CHEM32\2\METHODS\CR AJC ON.M
Last changed : 1/23/2015 2:25:57 PM by Steph Yip
(modified after loading)
Sample Info : OJ-H 98:2 HEX:IPA, 1mL/min.
racemic product

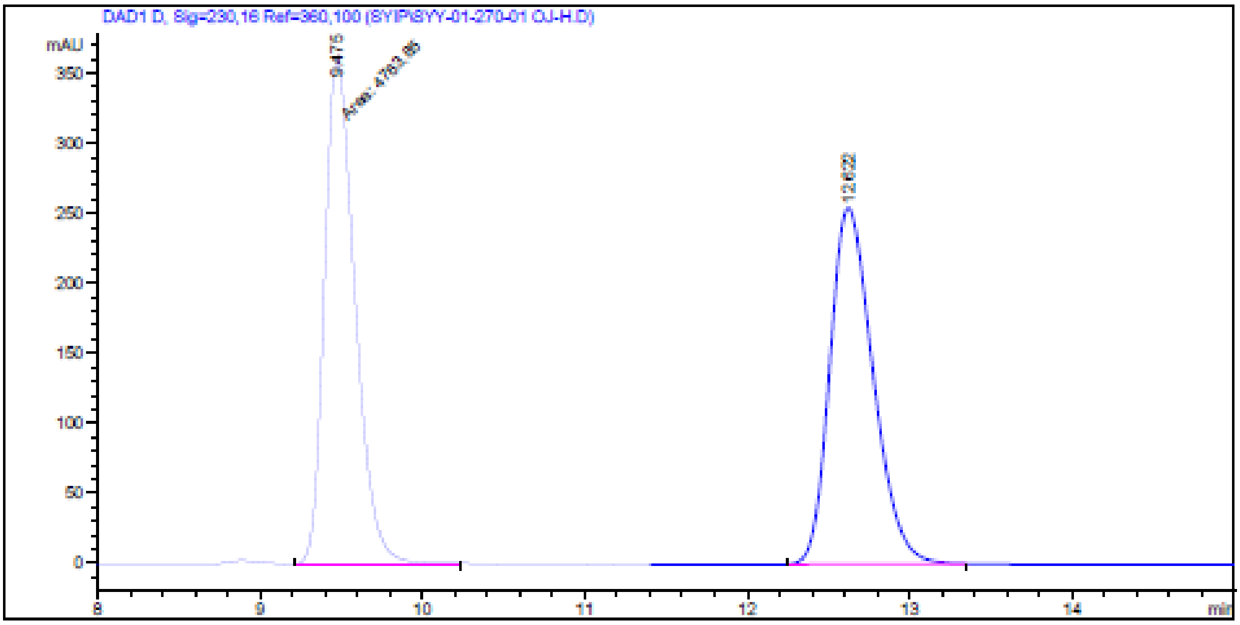

===
Area Percent Report
===

Sorted By : Signal
Multiplier : 1.0000
Dilution : 1.0000
Use Multiplier & Dilution Factor with ISTDs

Signal 1: DAD1 D, Sig=230,16 Ref=360,100

Peak #	RetTime [min]	Type	Width [min]	Area [mAU*s]	Height [mAU]	Area %
1	9.475	MM	0.2200	4763.84961	360.94324	50.2368
2	12.622	BB	0.2864	4718.93506	254.45750	49.7632

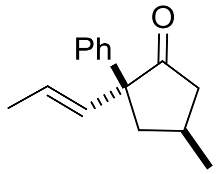

(±)-**17**
relative configuration only

Data File C:\CHEM32\2\DATA\SYIP\SYY-01-270-01 OJ-H.D
Sample Name: SYY-01-270-01 OJ-H

Totals : 9482.78467 615.40074

*** End of Report ***

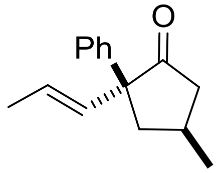

(±)-**17**
relative configuration only

```
Data File C:\CHEM32\2\DATA\SYIP\SYY-03-151-02 OJ-H.D
Sample Name: SYY-03-151-02 OJ-H

    =====================================================================
    Acq. Operator   : Steph Yip
    Acq. Instrument : Instrument 2                  Location : Vial 2
    Injection Date  : 1/23/2015 2:08:29 PM
                                                 Inj Volume : 5 µl
    Acq. Method     : C:\CHEM32\2\METHODS\SYY CYCLISATION METHOD.M
    Last changed    : 1/23/2015 2:02:31 PM by Steph Yip
                      (modified after loading)
    Analysis Method : C:\CHEM32\2\METHODS\CR AJC ON.M
    Last changed    : 1/23/2015 2:25:57 PM by Steph Yip
                      (modified after loading)
    Sample Info     : OJ-H 98:2 HEX:IPA, 1mL/min.
                      chiral product
```

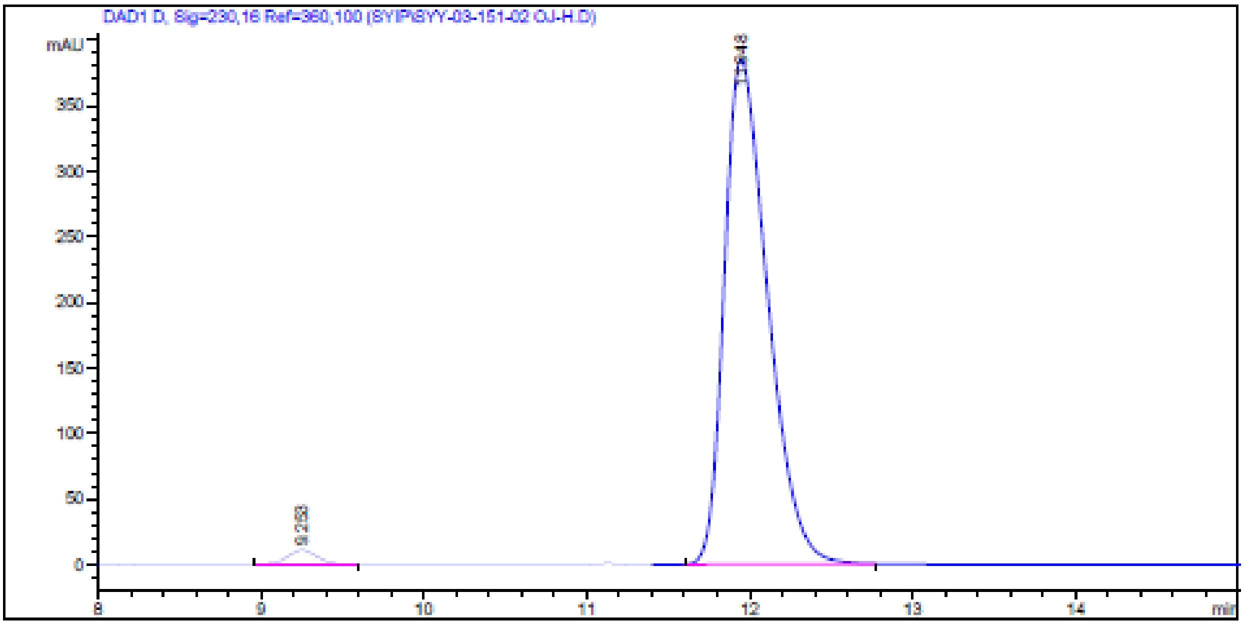


```
    =====================================================================
                         Area Percent Report
    =====================================================================

    Sorted By             :      Signal
    Multiplier            :      1.0000
    Dilution              :      1.0000
    Use Multiplier & Dilution Factor with ISTDs


    Signal 1: DAD1 D, Sig=230,16 Ref=360,100
```

Peak #	RetTime [min]	Type	Width [min]	Area [mAU*s]	Height [mAU]	Area %
1	9.253	BB	0.1989	141.26372	10.92173	1.9429
2	11.948	BB	0.2842	7129.33105	384.79364	98.0571

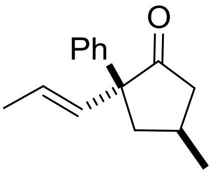

(–)-**17**

relative configuration only

Data File C:\CHEM32\2\DATA\SYIP\SYY-03-151-02 OJ-H.D
Sample Name: SYY-03-151-02 OJ-H

Totals : 7270.59477 395.71537

*** End of Report ***

Instrument 2 1/23/2015 2:28:05 PM Steph Yip

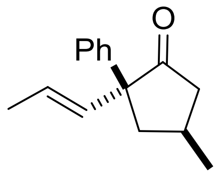

(–)-**17**
relative configuration only

Table SI-2 Crystal data and structure refinement for (*S*)-23

Empirical formula	$C_{20}H_{20}N_2O_7$		
Formula weight	400.38		
Temperature	150 °K		
Wavelength	1.54178		
Bond precision	C–C = 0.0033 Å		
Unit cell dimensions	a = 13.1111(4) Å	α = 90.00°	
	b = 8.4907(3) Å	β = 105.729°	
	c = 17.8251(5) Å	γ = 90.00°	
Volume	1910.03(10) $Å^3$		
Z	4		
Space group	P 21 (calculated)		P 1 21 1 (reported)
Hall group	P 2yb		
Density	1.392 mg/mm^3		
Absorption coefficient	0.897 mm^{-1}		
F(000)	840		
F(000’)	842.95 (calculated)		
h, k, l max	15, 10, 21		
Nref	7006 [3758] (calculated)		6959 (reported)
Tmin, Tmax	0.798, 0.836 (calculated)		0.676, 0.753 (reported)
Tmin’	0.764 (calculated)		
R (reflections)	0.0318 (6686)		
wR2 (reflections)	0.0864 (6959)		
S	1.038		
Npar	527		
Data completeness	1.85/0.99		
Theta (max)	68.281		

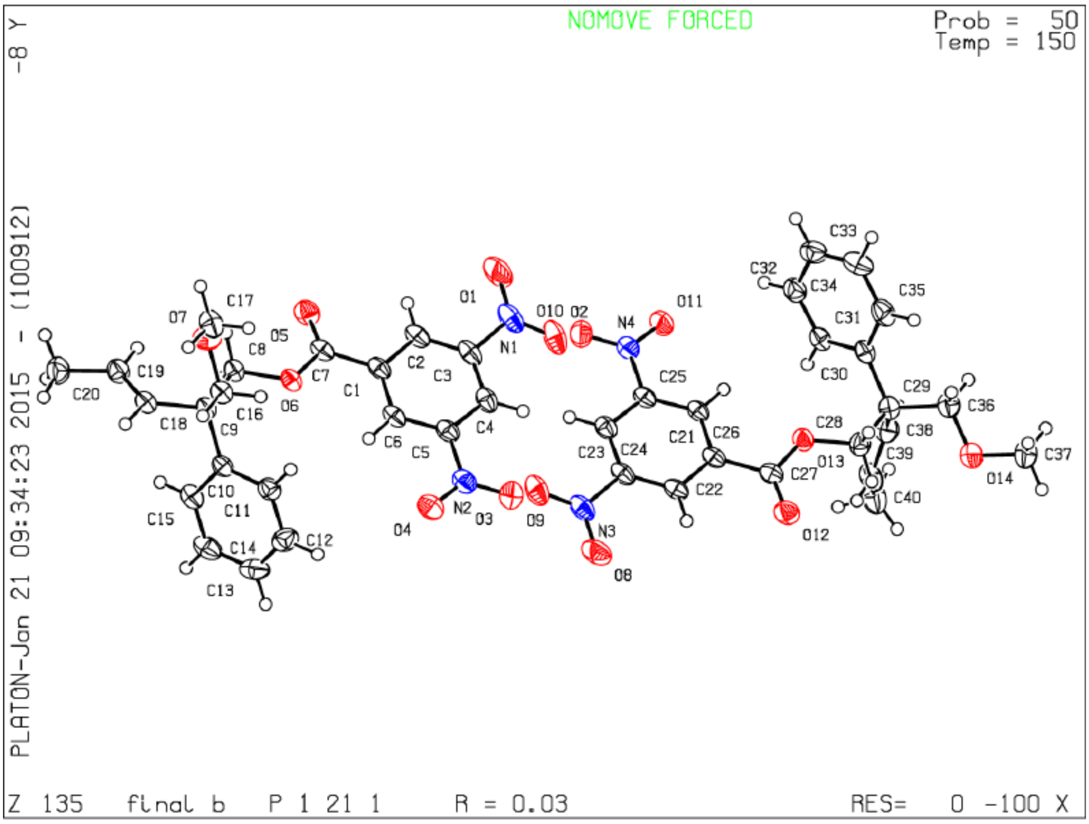

Supplement: Supplementary file 1 — miscellaneous_information [file anie0054-6870-sd1.pdf]
